# Supplementary material for: Building Up a Piperazine Ring from a Primary Amino Group via Catalytic Reductive Cyclization of Dioximes
Source: Int J Mol Sci. 2023 Jul 22;24(14):11794. doi: 10.3390/ijms241411794 (PMC10380651; doi:10.3390/ijms241411794)
Supplement: Supplementary file 1 [file ijms-24-11794-s001.zip › ijms-2515767-supplementary.pdf]

Supplementary material

Building up a piperazine ring from a primary amino group via catalytic reductive cyclization of dioximes

Evgeny V. Pospelov,[a] Alexey Yu. Sukhorukov[a,]*

[a] N. D. Zelinsky Institute of Organic Chemistry, 119991, Leninsky prospect, 47, Moscow, Russia

*Corresponding author: sukhorukov@ioc.ac.ru

Table of contents

1. General experimental S2

2. List of used ene-nitrosoacetals **3** S3

3. Experimental procedures and product characterization data S4

4. Stereochemistry elucidation in 2,6-disubstituted piperazines **1** S44

5. Assignment of *E*/*Z*-configuration and determination of isomer ratio in dioximes **2** S47

6. Copies of NMR spectra S52

5. References S208

1. General experimental

All the reactions were carried out in oven-dried (150 °C) glassware. NMR spectra were recorded at room temperature with peaks of residual solvents as internal standards. Multiplicities are indicated by s (singlet), d (doublet), t (triplet), q (quartet), m (multiplet), and br (broad). For ^{13}C spectra of *Boc*-**1c**, *EtCO*-**1j**, **2e**, **4d** apodization with exponential multiplication (3 Hz) was used. ^{1}H-^{15}N HMBC spectra was recorded using CH_3NO_2 as a relative compound. HRMS was measured on an electrospray ionization (ESI) instrument with a time-of-flight (TOF) detector. Column chromatography was performed using silica gel 40–60 μm 60A with petroleum ether–ethyl acetate mixtures as eluents. Analytical thin layer chromatography was performed on silica gel plates with QF-254. Visualization was accomplished with UV light and or with a solution of ninhydrin/CH_3CO_2H in ethanol.

CH_2Cl_2 and Et_3N were distilled from CaH_2, and DMF was distilled from CaH_2 under reduced pressure. Et_2O was distilled from $LiAlH_4$. Petroleum ether, methanol, ethanol, $CHCl_3$ and ethyl acetate were distilled without drying agents.

Raney nickel (Ra-Ni, ca. 50 % slurry in water), 5%-Pd/C, n-butylamine, benzylamine, tert-butylamine, cyclopentylamine, allylamine, α-phenylethylamine, propionic anhydride, dmap, glycine ethyl ester hydrochloride and L-leucine ethyl ester hydrochloride were commercial grade and were used as received.

Ene-nitrosoacetals **3a** [1], **3b** [1], **3c** [2], **3d** [3], **3e** [4], **3f** [5] were prepared in one step by silylation of the corresponding aliphatic nitro compounds in accordance with literature procedures.

Configurations of oximino-groups (*E*, *Z*) in designation of isomers of bis-oximes **2** and mono-oximes **4** are given in the same order they appear in IUPAC names.

2. List of used ene-nitrosoacetals 3

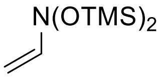

3a

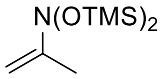

3b

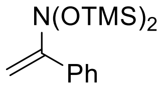

3c

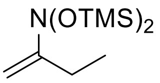

3d

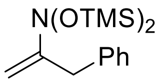

3e

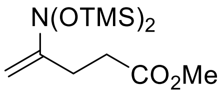

3f

3. Experimental procedures and product characterization data

3.1 Synthesis of α-amino acid ethyl esters

Synthesis of ethyl glycinate

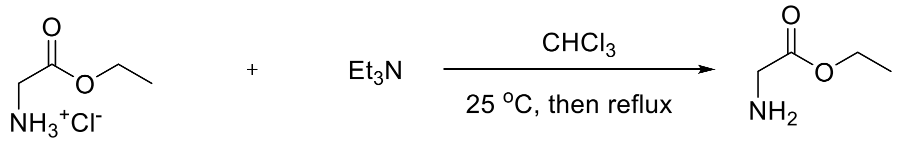

Et_3N (2.69 ml, 19.4 mmol) was added dropwise to a stirred solution of glycine ethyl ester hydrochloride (2.71 g, 19.4 mmol) in $CHCl_3$ (10 ml). The reaction mixture was stirred at room temperature for 17 hours and then heated to reflux at 1 hour. The resulting mixture was concentrated in vacuo. The precipitate was washed with Et_2O (3x25 ml) and resulting mixture was concentrated in vacuo to give 1180 mg (59 %) of pure ethyl glycinate as colorless oil. ^1H NMR spectrum is in agreement with previously published data [6].

Synthesis of ethyl L-leucinate

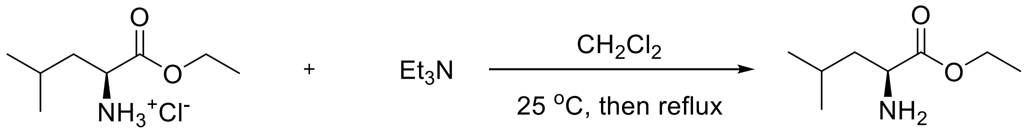

Et_3N (1.74 ml, 12.6 mmol) was added dropwise to a stirred solution of L-leucine ethyl ester hydrochloride (2.45 g, 12.6 mmol) in CH_2Cl_2 (7 ml). The reaction mixture was stirred at room temperature for 20 hours and then heated to reflux at 1 hour. The resulting mixture was concentrated in vacuo. The precipitate was washed with Et_2O (3x25 ml) and resulting mixture was concentrated in vacuo to give 1884 mg (94 %) of pure ethyl L-leucinate as colorless oil. ^1H NMR spectrum is in agreement with previously published data [7].

3.2 Synthesis and characterization of piperazines 1a–1r and product 5

General procedure for the synthesis of piperazines with 5%-Pd/C catalyst (Method A1). To a solution of dioxime **2** (1 eqv) in methanol (0.1 M) was added 5%-Pd/C catalyst (50 mg per 0.5 mmol of **2**). The vial was placed in a steel autoclave which was flushed and filled with hydrogen to a pressure of ca. 40 bar. Hydrogenation was conducted at this pressure and 50 °C for 6 hours with vigorous stirring. Then, the autoclave was cooled to rt and slowly depressurized, the catalyst was filtered off, and the solution was concentrated under reduced pressure. The residue was subjected to column chromatography on silica gel (eluent EtOAc : MeOH = 3 : 1).

General procedure for the synthesis of piperazines with 5%-Pd/C catalyst (Method A2). To a solution of dioxime **2** (1 eqv) and Boc_2O (3 eqv) in methanol (0.1 M of **2**) was added 5%-Pd/C catalyst (50 mg per 0.5 mmol of **2**). The vial was placed in a steel autoclave which was flushed and filled with hydrogen to a pressure of ca. 40 bar. Hydrogenation was conducted at this pressure and 50 °C for 6 hours with vigorous stirring. Then, the autoclave was cooled to rt and slowly depressurized, the catalyst was filtered off, and the solution was concentrated under reduced pressure. The residue was subjected to column chromatography on silica gel (eluent PE : EtOAc = 5 : 1).

General procedure for the synthesis of piperazines with Raney nickel catalyst (Method B1). A suspension of Ra-Ni (ca. 50 mg per 0.5 mmol of **2**) in methanol (1 ml) was added in a vial containing a solution of dioxime **2** (1 eqv) in methanol (0.1 M). The vial was placed in a steel autoclave which was flushed and filled with hydrogen to a pressure of ca. 40 bar. Hydrogenation was conducted at this pressure and 50 °C for 6 hours with vigorous stirring. Then, the autoclave was cooled to rt and slowly depressurized, the catalyst was filtered off, and the solution was concentrated under reduced pressure. The residue was subjected to column

chromatography on silica gel (eluent PE : EtOAc = 5 : 1 → 3 : 1 → 1 : 1 → EtOAc).

General procedure for the synthesis of piperazines with Raney nickel catalyst (Method B2). To a solution of dioxime **2** (1 eqv) and Boc_2O (3 eqv) in methanol (0.1 M of **2**) was added a suspension of Ra-Ni (ca. 50 mg per 0.5 mmol of **2**) in methanol (1 ml). The vial was placed in a steel autoclave which was flushed and filled with hydrogen to a pressure of ca. 40 bar. Hydrogenation was conducted at this pressure and 50 °C for 6 hours with vigorous stirring. Then, the autoclave was cooled to rt and slowly depressurized, the catalyst was filtered off, and the solution was concentrated under reduced pressure. The residue was subjected to column chromatography on silica gel (eluent PE : EtOAc = 5 : 1).

General procedure for the synthesis of piperazines 1i–1o with Raney nickel catalyst (Method B3). A suspension of Ra-Ni (ca. 50 mg per 0.5 mmol of **2**) in methanol (1 ml) was placed in a vial containing a solution of dioxime **2** (1 eqv) in methanol (0.1 M). The vial was placed in a steel autoclave which was flushed and filled with hydrogen to a pressure of ca. 40 bar. Hydrogenation was conducted at this pressure and 50 °C for 6 hours with vigorous stirring. Then, the autoclave was cooled to rt and slowly depressurized, the catalyst was filtered off, and the solution was concentrated under reduced pressure. The residue was dissolved in dichloromethane (3 mL per 0.5 mmol of **2**) and propionic anhydride (3 eqv), triethylamine (3 eqv) and dmap (1 eqv) were added to the solution. The mixture was placed in a refrigerator (about 0 ^{o}C) for 12 hours. Then the solution was concentrated under reduced pressure and the residue was subjected to column chromatography on silica gel (eluent PE : EtOAc = 5 : 1 → 3 : 1 → 1 : 1).

1-butylpiperazine (1a). The compound was prepared according to a general

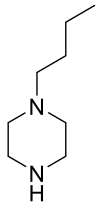

1a

procedure from dioxime **2a** (90 mg, 0.481 mmol). Yield: 30 mg (44 %, method A1). Yield: 0 mg (0 %, method B1). Yield: 0 mg (0 %, method B2). R_f = 0.3 (EtOAc-MeOH, 3:1). Colorless oil. ^{1}H NMR (300 MHz, $\mathrm{CDCl_3}$) δ 3.22 – 3.12 (m, 2H, $\mathrm{C}\mathit{H}_2$), 2.92 – 2.82 (m, 2H, $\mathrm{C}\mathit{H}_2$), 2.82-2.73 (m, 2H, $\mathrm{C}\mathit{H}_2$), 2.32 (t, J = 8.0 Hz, 2H, $\mathrm{C}\mathit{H}_2\mathrm{CH_2CH_2CH_3}$), 2.26 – 2.12 (m, 2H, $\mathrm{C}\mathit{H}_2$), 1.52 – 1.37 (m, 2H, $\mathrm{CH_2C}\mathit{H}_2\mathrm{CH_2CH_3}$), 1.37 – 1.23 (m, 2H, $\mathrm{CH_2CH_2C}\mathit{H}_2\mathrm{CH_3}$), 0.89 (t, J = 7.3 Hz, 3H, $\mathrm{CH_2CH_2CH_2C}\mathit{H}_3$), NH proton not observed. ^{13}C{^{1}H} NMR (75 MHz, DEPT135, $\mathrm{CDCl_3}$) δ 57.9 ($\mathit{C}\mathrm{H_2CH_2CH_2CH_3}$), 57.5 ($\mathit{C}\mathrm{H_2NBu}$), 51.9 ($\mathit{C}\mathrm{H_2NH}$), 29.2 ($\mathrm{CH_2}\mathit{C}\mathrm{H_2CH_2CH_3}$), 20.8 ($\mathrm{CH_2CH_2}\mathit{C}\mathrm{H_2CH_3}$), 14.1 ($\mathrm{CH_2CH_2CH_2}\mathit{C}\mathrm{H_3}$). ^{1}H NMR (300 MHz, DMSO-d_6) δ 7.96 (br s, 1H, $\mathrm{N}\mathit{H}$), 3.04 – 2.88 (m, 2H, $\mathrm{C}\mathit{H}_2$), 2.85 – 2.66 (m, 2H, $\mathrm{C}\mathit{H}_2$), 2.49-2.41 (m, 2H, $\mathrm{C}\mathit{H}_2$), 2.26 (t, J = 7.0 Hz, 2H, $\mathrm{C}\mathit{H}_2\mathrm{CH_2CH_2CH_3}$), 2.15 – 1.99 (m, 2H, $\mathrm{C}\mathit{H}_2$), 1.45 – 1.33 (m, 2H, $\mathrm{CH_2C}\mathit{H}_2\mathrm{CH_2CH_3}$), 1.33 – 1.19 (m, 2H, $\mathrm{CH_2CH_2C}\mathit{H}_2\mathrm{CH_3}$), 0.87 (t, J = 7.2 Hz, 3H, $\mathrm{CH_2CH_2CH_2C}\mathit{H}_3$). HRMS (ESI): m/z calcd. for $[\mathrm{C_8H_{19}N_2}]^+$ 143.1543, found 143.1544 $[\mathrm{M + H}]^+$.

Tert-butyl 4-butylpiperazine-1-carboxylate (*Boc*-1a). The compound was prepared according to a general procedure (method A2) from dioxime **2a** (90 mg,

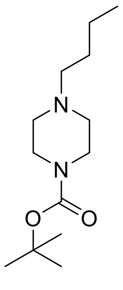

Boc-**1a**

0.481 mmol). Yield: 86 mg (74 %). R_f = 0.8 (PE-EtOAc, 1:1). Colorless oil. ^{1}H NMR (300 MHz, $\mathrm{CDCl_3}$) δ 3.53-3.41 (m, 4H, $\mathrm{CH_2}$), 2.45-2.39 (m, 4H, $\mathrm{CH_2}$), 2.35 (t, J = 8.0 Hz, 2H, $\mathrm{C}\mathit{H}_2\mathrm{CH_2CH_2CH_3}$), 1.56 – 1.47 (m, 2H, $\mathrm{CH_2C}\mathit{H}_2\mathrm{CH_2CH_3}$), 1.45 (s, 9H, $\mathrm{C}\mathit{H}_3$-C), 1.39 – 1.25 (m, 2H, $\mathrm{CH_2CH_2C}\mathit{H}_2\mathrm{CH_3}$), 0.91 (t, J = 7.3 Hz, 3H, $\mathrm{CH_2CH_2CH_2C}\mathit{H}_3$). ^{13}C{^{1}H} NMR (75 MHz, DEPT135, $\mathrm{CDCl_3}$) δ 154.8 (C=O), 79.8 (C), 58.6 ($\mathit{C}\mathrm{H_2CH_2CH_2CH_3}$), 53.1 ($\mathit{C}\mathrm{H_2NBu}$), 42.9 ($\mathit{C}\mathrm{H_2NBoc}$), 28.8 ($\mathrm{CH_2}\mathit{C}\mathrm{H_2CH_2CH_3}$), 28.6 ($\mathit{C}\mathrm{H_3}$-C), 20.8 ($\mathrm{CH_2CH_2}\mathit{C}\mathrm{H_2CH_3}$), 14.1 ($\mathrm{CH_2CH_2CH_2}\mathit{C}\mathrm{H_3}$). HRMS (ESI): m/z calcd. for $[\mathrm{C_{13}H_{27}N_2O_2}]^+$ 243.2067, found 243.2066 $[\mathrm{M + H}]^+$.

Tert-butyl 4-(tert-butyl)piperazine-1-carboxylate (*Boc*-1b). The compound was prepared according to a general procedure (method A2) from dioxime **2b** (80 mg, 0.428 mmol). Yield: 44 mg (42 %). R_f = 0.5 (PE-EtOAc, 1:1). White solid. ^{1}H NMR (300 MHz, $CDCl_3$) δ 3.65 – 3.18 (m, 4H, C*H*$_2$NBoc), 2.68 – 2.18 (m, 4H, C*H*$_2$NC), 1.43 (s, 9H, C*H*$_3$CO), 1.04 (s, 9H, C*H*$_3$CN). ^{13}C{^{1}H} NMR (75 MHz, DEPT135, $CDCl_3$) δ 154.8 (C=O), 79.5 (C-O), 54.1 (*C*-CH_3), 45.8 (*C*H_2NC), 44.2 (*C*H_2NBoc), 28.6 (*C*H_3CO), 26.0 (*C*H_3CN). M.P. = 50-53 °C. ^{1}H NMR spectrum is in agreement with previously published data [8].

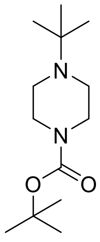

Boc-**1b**

Tert-butyl 4-cyclopentylpiperazine-1-carboxylate (*Boc*-1c). The compound was prepared according to a general procedure (method A2) from dioxime **2c** (90 mg, 0.452 mmol). Yield: 49 mg (43 %). R_f = 0.6 (PE-EtOAc, 1:1). Colorless oil. ^{1}H NMR (300 MHz, $CDCl_3$) δ 3.44 (m, 4H, CH_2), 2.48 (m, 1H, CH), 2.43 (m, 4H, CH_2), 1.84 (m, 2H, C*H*$_2$CH), 1.76-1.61 (m, 2H, C*H*$_2$CH), 1.61-1.48 (m, 2H, C*H*$_2$$CH_2$CH), 1.45 (s, 9H, CH_3), 1.40 – 1.33 (m, 2H, C*H*$_2$$CH_2$CH). ^{13}C{^{1}H} NMR (75 MHz, DEPT135, $CDCl_3$) δ ^{13}C NMR (75 MHz, $CDCl_3$) δ 154.9 (C=O), 79.7 (C), 67.6 (CH), 52.2 (*C*H_2N-Cyp), 43.6 (*C*H_2NBoc), 30.5 (CH_2*C*H_2CH), 28.6 (*C*H_3), 24.2 (*C*$H_2$$CH_2$CH). ^{15}N NMR (300 MHz, HMBC, $CDCl_3$) δ -295.8 (*N*-CH), -321.8 (*N*-Boc) (relative to nitromethane). HRMS (ESI): m/z calcd. for $[C_{14}H_{27}N_2O_2]^+$ 255.2067, found 255.2061 $[M + H]^+$.

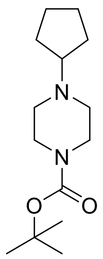

Boc-**1c**

Tert-butyl 4-propylpiperazine-1-carboxylate (*Boc*-1d'). The compound was prepared according to a general procedure (method A2) from dioxime **2d** (50 mg, 0.292 mmol). Yield: 45 mg (68 %). R_f = 0.75 (PE-EtOAc, 1:1). Colorless oil. ^{1}H NMR spectrum is in agreement with previously published data [9].

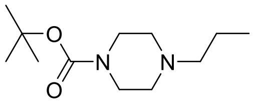

Boc-**1d'**

Di-tert-butyl piperazine-1,4-dicarboxylate (***Boc*-1e'**). The compound was prepared according to a general procedure (method A2) from dioxime **2e** (100 mg, 0.453 mmol). Yield: 75 mg (58 %). R_f = 0.85 (PE-EtOAc, 1:1). White solid. ^{1}H NMR (300 MHz, $CDCl_3$) δ 3.35 (s, 8H, CH_2), 1.43 (s, 18H, CH_3). $^{13}C\{^1H\}$ NMR (75 MHz, DEPT135, $CDCl_3$) δ 154.8 (2 C=O), 80.1 (2 C), 43.6 (4 CH_2), 28.5 (6 CH_3). HRMS (ESI): m/z calcd. for $[C_{14}H_{27}N_2O_4]^+$ 287.1965, found 287.1958 $[M + H]^+$.

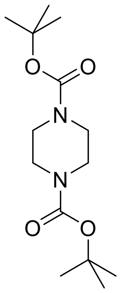

Boc-**1e'**

Tert-butyl (*S*)-4-(1-phenylethyl)piperazine-1-carboxylate (***Boc*-1f**). The compound was prepared according to a general procedure (method B2) from dioxime **2f** (90 mg, 0.383 mmol). Yield: 53 mg (48 %). Colorless oil. R_f = 0.8 (PE-EtOAc, 1:1). $[\alpha]_D$ = –30.3 (c=0.25, MeOH, 26 ^{o}C). ^{1}H NMR spectrum is in agreement with previously published data [8].

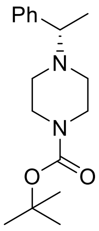

Boc-**1f**

Tert-butyl 4-(2-ethoxy-2-oxoethyl)piperazine-1-carboxylate (***Boc*-1g**). The compound was prepared according to a general procedure (method A2) from dioxime **2g** (55 mg, 0.253 mmol). Yield: 45 mg (65 %). R_f = 0.8 (PE-EtOAc, 1:1). Colorless oil. ^{1}H NMR spectrum is in agreement with previously published data [10].

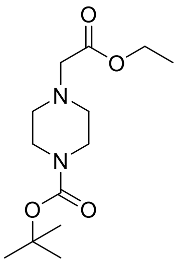

Boc-**1g**

Tert-butyl (S)-4-(1-ethoxy-4-methyl-1-oxopentan-2-yl)piperazine-1-carboxylate (*Boc*-1h). The compound was prepared according to a general procedure (method A2) from dioxime **2h** (100 mg, 0.366 mmol). Yield: 55 mg (46 %). R_f = 0.7 (PE-EtOAc, 1:1). $[\alpha]_D = -18.6$ (c=1, MeOH, 26 °C). Colorless oil. ^{1}H NMR (300 MHz, $CDCl_3$) δ 4.17 (q, J = 7.1 Hz, 2H, CH_2CH_3), 3.43 (m, 4H, CH_2NBoc), 3.31 (m, 1H, C*H*N), 2.63 (m, 4H, *C*H_2NCH), 1.62 (m, 3H, CHCH_2CH+C*H*CH_3), 1.45 (s, 9H, CH_3-C), 1.28 (t, J = 7.1 Hz, 3H, CH_2CH_3), 0.92 (d, J = 6.2 Hz, 3H, CH_3CH), 0.90 (d, J = 6.2 Hz, 3H, CH_3CH). ^{13}C{^{1}H} NMR (75 MHz, DEPT135, $CDCl_3$) δ 171.9 (O-*C*=O), 154.7 (N-*C*=O), 79.6 (*C*), 65.6 (*C*HN), 60.3 (*C*H_2CH_3), 49.1 (*C*H_2NCH), 44.1 (*C*H_2NBoc), 37.9 (CH*C*H_2CH), 28.4 (*C*H_3-C), 25.0 (CH_3*C*H), 22.5 (*C*H_3CH), 14.4 (CH_2*C*H_3). HRMS (ESI): m/z calcd. for $[C_{17}H_{33}N_2O_4]^+$ 329.2435, found 329.2428 $[M + H]^+$.

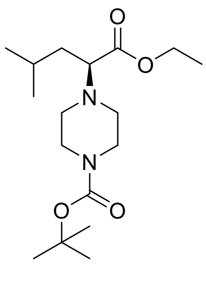

Boc-**1h**

1-(4-butyl-2,6-dimethylpiperazin-1-yl)propan-1-one (*EtCO*-1i). The compound was prepared according to a general procedure (method B3) from dioxime **2i** (90 mg, 0.420 mmol). Mixture of stereoisomers, which were separated by column chromatography. *Cis* : *trans* = 6.1:1. Yield: 50 mg (53 %). ***Cis*-isomer**: Colorless oil. R_f = 0.5 (PE-EtOAc, 1:1). ^{1}H NMR (300 MHz, $CDCl_3$) δ 4.76-3.72 (m, 2H, C*H*CH_3), 2.67 (d, J = 11.3 Hz, 2H, CH_2CH), 2.33 (q, J = 7.4 Hz, 2H, COCH_2CH_3), 2.29 (t, J = 6.9 Hz, 2H, CH_2CH_2CH_2CH_3), 2.04 (dd, J = 11.3, 4.4 Hz, 2H, CH_2CH), 1.52 – 1.38 (m, 4H, CH_2CH_2CH_2CH_3 + CH_2CH_2CH_2CH_3), 1.33 (d, J = 6.8 Hz, 6H, CH_3CH), 1.15 (t, J = 7.4 Hz, 3H, COCH_2CH_3), 0.92 (t, J = 7.1 Hz, 3H, CH_2CH_2CH_2CH_3). ^{13}C{^{1}H} NMR (75 MHz, DEPT135, $CDCl_3$) δ 172.7 (C=O), 58.2 (*C*H_2CH), 57.9 (*C*H_2CH_2CH_2CH_3), 49.1 (*C*HCH_3), 45.4 (*C*HCH_3), 29.1 (CH_2*C*H_2CH_2CH_3), 26.4 (CO*C*H_2CH_3), 21.4 (*C*H_3CH), 21.1 (*C*H_3CH), 20.5 (CH_2CH_2*C*H_2CH_3), 14.1

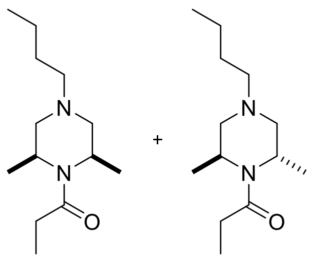

EtCO-**1i**

(CH$_2$CH$_2$CH$_2$*C*H$_3$), 9.8 (*C*H$_3$CH$_2$CO). HRMS (ESI): m/z calcd. for $[C_{13}H_{27}N_2O]^+$ 227.2118, found 227.2109 $[M + H]^+$.

***Trans*-isomer**: Colorless oil. R_f = 0.3 (PE-EtOAc, 1:1). ^{1}H NMR (300 MHz, CDCl$_3$) δ 3.91 (m, 2H, C*H*CH$_3$), 2.67 (d, J = 11.0 Hz, 2H, C*H*$_2$CH), 2.50 – 2.20 (m, 6H, C*H*$_2$CH+COC*H*$_2$CH$_3$+C*H*$_2$CH$_2$CH$_2$CH$_3$), 1.52 – 1.39 (m, 2H, CH$_2$C*H*$_2$CH$_2$CH$_3$), 1.36 (d, J = 6.5 Hz, 6H, C*H*$_3$CH), 1.32 – 1.21 (m, 2H, CH$_2$CH$_2$C*H*$_2$CH$_3$), 1.13 (t, J = 7.4 Hz, 3H, COCH$_2$C*H*$_3$), 0.91 (t, J = 7.2 Hz, 3H, CH$_2$CH$_2$CH$_2$C*H*$_3$). ^{13}C{^{1}H} NMR (75 MHz, DEPT135, CDCl$_3$) δ 175.7 (C=O), 58.5 (*C*H$_2$CH$_2$CH$_2$CH$_3$), 58.0 (*C*H$_2$CH), 49.1 (*C*HCH$_3$), 29.4 (CH$_2$*C*H$_2$CH$_2$CH$_3$), 27.9 (CO*C*H$_2$CH$_3$), 20.6 (CH$_2$CH$_2$*C*H$_2$CH$_3$), 20.1 (*C*H$_3$CH), 14.2 (CH$_2$CH$_2$CH$_2$*C*H$_3$), 9.9 (*C*H$_3$CH$_2$CO). HRMS (ESI): m/z calcd. for $[C_{13}H_{27}N_2O]^+$ 227.2118, found 227.2113 $[M + H]^+$.

1-(4-cyclopentyl-2,6-dimethylpiperazin-1-yl)propan-1-one (*EtCO*-1j). The compound was prepared according to a general procedure (method B3) from dioxime **2j** (90 mg, 0.442 mmol). Mixture of stereoisomers, which were separated by column chromatography. *Cis* : *trans* = 2.8:1. Yield: 72 mg (69 %). ***Cis*-isomer**: Colorless oil. R_f = 0.6 (PE-EtOAc, 1:1). ^{1}H NMR (300 MHz, HSQC, CDCl$_3$) δ 4.69 – 3.85 (m, 2H, C*H*CH$_3$), 2.77 (d, J = 11.2 Hz, 2H, NC*H*$_2$CHN), 2.47 (ddd, J = 15.4, 8.4, 7.0 Hz, 1H, CH$_2$C*H*CH$_2$), 2.34 (q, J = 7.3 Hz, 2H, C*H*$_2$CH$_3$), 2.07 (dd, J = 11.2, 4.3 Hz, 2H, NC*H*$_2$CHN), 1.88 – 1.75 (m, 2H, C*H*$_2$CHC*H*$_2$), 1.68 (m, 2H, C*H*$_2$CH$_2$CH), 1.56 (m, 2H, C*H*$_2$CH$_2$CH), 1.48 – 1.37 (m, 2H, C*H*$_2$CHC*H*$_2$), 1.33 (d, J = 6.8 Hz, 6H, C*H*$_3$CH), 1.16 (t, J = 7.3 Hz, 3H, CH$_2$C*H*$_3$). ^{13}C{^{1}H} NMR (75 MHz, DEPT135, CDCl$_3$) δ 172.7 (C=O), 66.5 (*C*HN), 57.1 (N*C*H$_2$CHN), 48.8 (*C*HCH$_3$), 46.1 (*C*HCH$_3$), 30.8 (CH$_2$*C*H$_2$CH), 26.3 (CO*C*H$_2$CH$_3$), 24.3 (*C*H$_2$CH$_2$CH), 21.2 (*C*H$_3$CH), 9.8 (*C*H$_3$CH$_2$). HRMS (ESI): m/z calcd. for $[C_{14}H_{27}N_2O]^+$ 239.2118, found 239.2117 $[M + H]^+$.

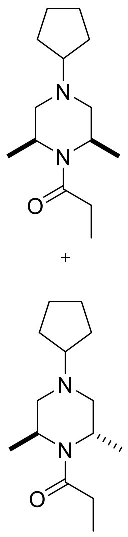

EtCO-**1j**

***Trans*-isomer**: Colorless oil. R_f = 0.3 (PE-EtOAc, 1:1). ^{1}H NMR (300 MHz, HSQC, $CDCl_3$) δ 3.88 (m, 2H, C*H*CH_3), 2.70 (d, J = 11.3 Hz, 2H, NC*H*$_2$CHN), 2.58 (ddd, J = 15.7, 8.6, 6.9 Hz, 1H, CH_2C*H*CH_2), 2.49 – 2.23 (m, 4H, NC*H*$_2$CHN+C*H*$_2$$CH_3$), 1.88 – 1.44 (m, 8H, CH_2C*H*$_2$CH+C*H*$_2$$CH_2$CH), 1.34 (d, J = 6.5 Hz, 6H, CHC*H*$_3$), 1.11 (t, J = 7.4 Hz, 3H, CH_2C*H*$_3$). ^{13}C{^{1}H} NMR (75 MHz, DEPT135, $CDCl_3$) δ 175.7 (C=O), 67.2 (CH_2*C*HCH_2), 56.8 (N*C*H_2CHN), 49.1 (*C*HCH_3), 30.7 (CH_2*C*H_2CH), 30.6 (CH_2*C*H_2CH), 27.8 (CO*C*$H_2$$CH_3$), 24.1 (*C*$H_2$$CH_2$CH), 20.2 (*C*$H_3$CH), 9.8 (*C*$H_3$$CH_2$CO). HRMS (ESI): m/z calcd. for $[C_{14}H_{27}N_2O]^+$ 239.2118, found 239.2115 $[M + H]^+$.

1-(2,6-dimethyl-4-((S)-1-phenylethyl)piperazin-1-yl)propan-1-one (*EtCO*-1k).

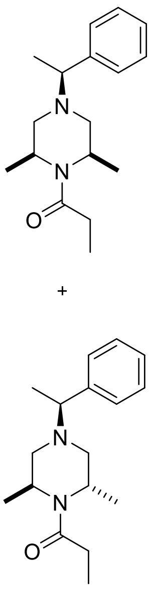

EtCO-**1k**

The compound was prepared according to a general procedure (method B3) from dioxime **2k** (80 mg, 0.302 mmol). Inseparable mixture of stereoisomers. *Cis* : *trans* = 3:1. Yield: 51 mg (62 %). R_f = 0.7 (PE-EtOAc, 1:1). Colorless oil. $[\alpha]_D$ = –12.9 (c=0.07, MeOH, 25 ^{o}C). ***Cis*-isomer** (characterized in mixture with *trans*-isomer): ^{1}H NMR (300 MHz, $CDCl_3$) δ 7.45 – 7.17 (m, 5H, Ph), 4.62 – 3.78 (m, 2H, C*H*CH_3), 3.38 (q, J = 6.7 Hz, 1H, C*H*Ph), 2.89 (m, 1H, C*H*$_2$CH), 2.59 (m, 1H, C*H*$_2$CH), 2.41 – 2.22 (q, J = 7.5 Hz, 2H, CH_3C*H*$_2$CO), 2.16 (dd, J = 11.1, 4.4 Hz, 1H, C*H*$_2$CH), 2.05 (dd, J = 11.4, 4.3 Hz, 1H, C*H*$_2$CH), 1.43 – 1.29 (m, 6H, 2CHC*H*$_3$), 1.29 (d, J = 6.8 Hz, 3H, C*H*$_3$CHPh), 1.15 (t, J = 7.5 Hz, 3H, C*H*$_3$$CH_2$CO). ^{13}C{^{1}H} NMR (75 MHz, DEPT135, $CDCl_3$) δ 172.5 (C=O), 144.4 (i-Ph), 128.3 (m-Ph), 127.5 (o-Ph), 127.1 (p-Ph), 64.1 (*C*HPh), 56.1 (*C*H_2CH), 54.6 (*C*H_2CH), 49.2 (CH_2*C*HCH_3), 46.8 (CH_2*C*HCH_3), 26.3 (CO*C*$H_2$$CH_3$), 21.3 (*C*$H_3CHCH_2$), 21.0 (*C*$H_3CHCH_2$), 19.8 (*C*$H_3$CHPh), 9.7 (COC$H_2$*C*$H_3$).

***Trans*-isomer** (characterized in mixture with *cis*-isomer): ^{1}H NMR (300 MHz, $CDCl_3$) δ 7.45 – 7.17 (m, 5H, Ph), 3.88 (m, 2H, C*H*CH_3), 3.55 (q, J = 6.7 Hz, 1H, C*H*Ph), 2.72 (m, 2H, C*H*$_2$CH), 2.41 – 2.22 (q, J = 7.5 Hz, 2H, CH_3C*H*$_2$CO), 2.31

(m, 2H, CH_2CH), 1.43 – 1.29 (m, 9H, 2CH_3CH+CH_3CHPh), 1.15 (t, J = 7.5 Hz, 3H, CH_3CH_2CO). ^{13}C{^{1}H} NMR (75 MHz, DEPT135, $CDCl_3$) δ 175.9 (C=O), 144.3 (i-Ph), 128.4 (m-Ph), 127.5 (o-Ph), 127.0 (p-Ph), 64.3 (*C*HPh), 55.7 (*C*H_2CH), 54.6 (*C*H_2CH), 49.3 (CH_2*C*HCH_3), 27.9 (CO*C*H_2CH_3), 20.2 (*C*H_3CHCH_2), 19.9 (*C*H_3CHCH_2), 19.5 (*C*H_3CHPh), 9.7 (COCH_2*C*H_3).

HRMS (ESI): m/z calcd. for $[C_{17}H_{27}N_2O]^+$ 275.2118, found 275.2113 $[M + H]^+$.

Ethyl 2-(3,5-dimethyl-4-propionylpiperazin-1-yl)acetate (*EtCO*-1l). The compound was prepared according to a general procedure (method B3) from dioxime **2l** (70 mg, 0.285 mmol). Inseparable mixture of stereoisomers. *Cis* : *trans* = 5.1 : 1. Yield: 50 mg (69 %). R_f = 0.7 (PE-EtOAc, 1:1). Colorless oil. ***Cis*-isomer** (characterized in mixture with *trans*-isomer): ^{1}H NMR (300 MHz, $CDCl_3$) δ 4.42-4.10 (m, 2H, C*H*CH_3), 4.18 (q, J = 7.1 Hz, 2H, COOCH_2CH_3), 3.22 (s, 2H, CH_2CO_2Et), 2.73 (d, J = 11.1 Hz, 2H, CHCH_2), 2.40-2.25 (m, 4H, CHCH_2+COCH_2CH_3), 1.38 (d, J = 6.7 Hz, 6H, CHCH_3), 1.28 (t, J = 7.1 Hz, 3H, COOCH_2CH_3), 1.16 (t, J = 7.4 Hz, 3H, COCH_2CH_3). ^{13}C{^{1}H} NMR (75 MHz, DEPT135, $CDCl_3$) δ 172.6 (C=O), 170.2 (*C*OOCH_2CH_3), 60.4 (COO*C*H_2CH_3), 59.4 (*C*H_2COOEt), 57.6 (*C*H_2CH), 49.0 (C*H*CH_3), 46.4 (C*H*CH_3), 26.2 (CO*C*H_2CH_3), 20.9 (*C*H_3CH), 19.9 (*C*H_3CH), 14.2 (COOCH_2CH_3), 9.6 (COCH_2*C*H_3).

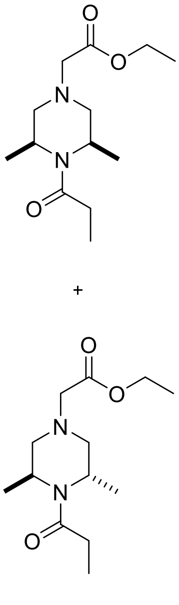

EtCO-**1l**

***Trans*-isomer** (characterized in mixture with *cis*-isomer): ^{1}H NMR (300 MHz, $CDCl_3$) δ 4.18 (q, J = 7.1 Hz, 2H, COOCH_2CH_3), 3.96 (dtd, J = 11.6, 6.5, 3.9 Hz, 2H, C*H*CH_3), 3.29 (d, J = 7.6 Hz, 2H, CH_2CO_2Et), 2.92 (dd, J = 11.5, 3.8 Hz, 2H, CHCH_2), 2.57 (dd, J = 11.5, 5.0 Hz, 2H, CHCH_2), 2.40-2.25 (m, 2H, COCH_2CH_3) 1.38 (d, J = 6.7 Hz, 6H, CHCH_3), 1.28 (t, J = 7.1 Hz, 3H, COOCH_2CH_3), 1.16 (t, J = 7.4 Hz, 3H, COCH_2CH_3). ^{13}C{^{1}H} NMR (75 MHz, DEPT135, $CDCl_3$) δ 172.6 (C=O), 170.4 (*C*OOCH_2CH_3), 60.4 (COO*C*H_2CH_3), 59.3 (*C*H_2COOEt), 56.9

(CH_2CH), 49.0 (C*H*CH_3), 27.6 (CO*C*H_2CH_3), 20.9 (*C*H_3CH), 14.2 ($COOCH_2C$*H_3*), 9.6 ($COCH_2$*C*H_3).

HRMS (ESI): m/z calcd. for $[C_{13}H_{25}N_2O_3]^+$ 257.1860, found 257.1852 $[M + H]^+$.

1-(4-benzyl-2,6-dimethylpiperazin-1-yl)propan-1-one (*EtCO*-1m). The compound was prepared according to a general procedure (method B3) from dioxime **2m** (100 mg, 0.4 mmol). Obtained as a sole *cis*-isomer. Yield: 74 mg (71 %). R_f = 0.6 (PE-EtOAc, 1:1). Colorless oil. ^{1}H NMR (300 MHz, $CDCl_3$) δ 7.44 – 7.16 (m, 5H, Ph), 4.56 (m, 1H, C*H*CH_3), 3.91 (qd, J = 6.4, 4.2 Hz, 1H, C*H*CH_3), 3.50 (s, 2H, CH_2Ph), 2.68 (d, J = 11.3 Hz, 2H, C*H*$_2$CH), 2.35 (m, 2H, C*H*$_2CH_3$), 2.14 (dd, J = 11.3, 4.2 Hz, 2H, C*H*$_2$CH), 1.39 (d, J = 6.4 Hz, 6H, CHC*H*$_3$), 1.16 (t, J = 7.5 Hz, 3H, CH_2C*H*$_3$). $^{13}C\{^1H\}$ NMR (75 MHz, DEPT135, $CDCl_3$) δ 172.6 (C=O), 138.5 (i-Ph), 128.6 (m-Ph), 128.3 (o-Ph), 127.1 (p-Ph), 62.7 (*C*H_2Ph), 57.9 (*C*H_2CH), 48.2 (CH_3*C*H), 44.9 (CH_3*C*H), 26.3 (CO*C*H_2CH_3), 21.3 (*C*H_3CH), 19.8 (*C*H_3CH), 9.7 (CH_2*C*H_3). HRMS (ESI): m/z calcd. for $[C_{16}H_{25}N_2O]^+$ 261.1961, found 261.1958 $[M + H]^+$.

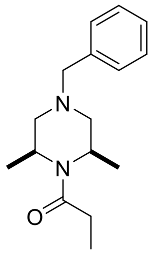

EtCO-**1m**

Tert-butyl (3S,5R)-3,5-dimethylpiperazine-1-carboxylate (1n). The compound was prepared according to a general procedure (method B1) from dioxime **2n** (110 mg, 0.423 mmol). Obtained as a sole *cis*-isomer. Yield: 37 mg (46 %). R_f = 0.4 (PE-EtOAc, 1:1). Colorless oil. ^{1}H NMR spectrum is in agreement with previously published data [11].

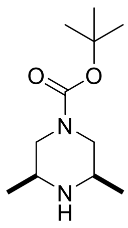

1n

(3S,5R)-1-butyl-3,5-diphenylpiperazine (1o). The compound was prepared according to a general procedure (method B1) from dioxime **2o** (90 mg, 0.296 mmol). Obtained as a sole *cis*-isomer.Yield: 53 mg (61 %). R_f = 0.3 (PE-EtOAc, 1:1). Colorless oil. ^{1}H NMR (300 MHz, $CDCl_3$) δ 7.62 – 7.24 (m, 10H, Ph), 4.31 – 4.19 (m, 2H, C*H*), 3.08 (dd, J = 11.3, 2.6 Hz, 2H, $CHCH_2$), 2.59 – 2.45 (m, 2H, $CH_2CH_2CH_2CH_3$), 2.16 (t, J = 10.8 Hz, 2H, $CHCH_2$), 1.58 (m, 2H, $CH_2CH_2CH_2CH_3$), 1.35 (m, 2H, $CH_2CH_2CH_2CH_3$), 0.93 (t, J = 7.3 Hz, 3H, $CH_2CH_2CH_2CH_3$) (N-H-proton was not observed). $^{13}C\{^1H\}$ NMR (75 MHz, DEPT135, $CDCl_3$) δ 142.3 (p-Ph), 128.4 (m-Ph), 127.7 (i-Ph), 127.2 (o-Ph), 60.7 ($CHCH_2$), 59.9 (*C*H), 58.4 ($CH_2CH_2CH_2CH_3$), 28.4 ($CH_2CH_2CH_2CH_3$), 20.7 ($CH_2CH_2CH_2CH_3$), 14.0 ($CH_2CH_2CH_2CH_3$). HRMS (ESI): m/z calcd. for $[C_{20}H_{27}N_2]^+$ 295.2169, found 295.2161 $[M + H]^+$.

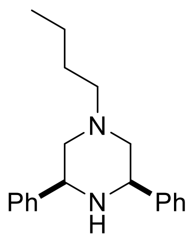

1o

Tert-butyl 4-butyl-2-methylpiperazine-1-carboxylate (*Boc*-1p). The compound was prepared according to a general procedure (method A2) from dioxime **2p** (48 mg, 0.239 mmol). Yield: 32 mg (53 %). R_f = 0.75 (PE-EtOAc, 1:1). Colorless oil. ^{1}H NMR (300 MHz, $CDCl_3$) δ 4.19 (s, 1H, C*H*), 3.79 (m, 1H, CH_2NBoc), 3.11 (m, 1H, CH_2NBoc), 2.79 (m, 1H, CH_2NBu), 2.67 (m, 1H, CH_2CH), 2.44 – 2.16 (m, 2H, $CH_2CH_2CH_2CH_3$), 2.09 (m, 1H, CH_2CH), 1.95 (m, 1H, CH_2NBu), 1.45 (s, 9H, CH_3C), 1.43 (m, 2H, $CH_2CH_2CH_2CH_3$), 1.28 – 1.18 (m, 2H, $CH_2CH_2CH_2CH_3$), 1.14 (d, J = 6.7 Hz, 3H, CH_3CH), 0.81 (t, J = 7.2 Hz, 3H, $CH_2CH_2CH_2CH_3$). $^{13}C\{^1H\}$ NMR (75 MHz, DEPT135, $CDCl_3$) δ 150.8 (C=O), 79.4 (C), 58.3 ($CH_2CH_2CH_2CH_3$), 57.5 (CH_2CH), 53.6 (CH_2NBu), 47.0 (*C*H), 39.0 (CH_2NBoc), 28.9 ($CH_2CH_2CH_2CH_3$), 28.5 (CH_3C), 20.6 ($CH_2CH_2CH_2CH_3$), 16.1 (CH_3CH), 14.0 ($CH_2CH_2CH_2CH_3$). HRMS (ESI): m/z calcd. for $[C_{14}H_{29}N_2O_2]^+$ 257.2224, found 257.2218 $[M + H]^+$.

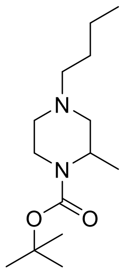

Boc-**1p**

Tert-butyl 4-butyl-2-ethylpiperazine-1-carboxylate (*Boc*-1q). The compound was prepared according to a general procedure (method A2) from dioxime **2q** (50 mg, 0.233 mmol). Yield: 45 mg (71 %). R_f = 0.75 (PE-EtOAc, 1:1). Colorless oil. ^{1}H NMR (300 MHz, $CDCl_3$) δ 3.99 (m, 1H, C*H*CH_2CH_3), 3.91 (m, 1H, CH_2NBoc), 3.11 (m, 1H, CH_2NBoc), 2.83 (m, 1H, CH_2NBu), 2.49 – 2.25 (m, 2H, CH_2CH), 2.06 (m, 1H, CH_2NBu), 1.94 – 1.78 (m, 3H, CH_2C$H_2$$CH_2CH_3$ + CHC$H_2$$CH_3$), 1.78 – 1.62 (m, 3H, CH_2CH_2C$H_2$$CH_3$ + CHC$H_2$$CH_3$), 1.46 (s, 9H, C$H_3$C), 1.35 (m, 2H, CH_2CH_2C$H_2$$CH_3$), 0.94 (t, J = 7.3 Hz, 3H, $CH_2CH_2CH_2$CH_3), 0.88 (t, J = 7.3 Hz, 3H, $CHCH_2$CH_3). ^{13}C{^{1}H} NMR (75 MHz, DEPT135, $CDCl_3$) δ 155.4 (C=O), 79.5 (C), 58.3 (*C*$H_2CH_2CH_2CH_3$), 55.0 (*C*H_2CH), 53.6 (*C*H_2NBu), 52.6 (*C*HCH_2CH_3), 39.0 (*C*H_2NBoc), 28.4 (*C*H_3C), 23.0 (CH_2*C*$H_2CH_2CH_3$), 22.1 (CH*C*H_2CH_3), 20.5 (CH_2CH_2*C*H_2CH_3), 14.0 ($CH_2CH_2CH_2$*C*H_3), 10.8 ($CHCH_2$*C*H_3). HRMS (ESI): m/z calcd. for $[C_{15}H_{31}N_2O_2]^+$ 271.2380, found 271.2372 $[M + H]^+$.

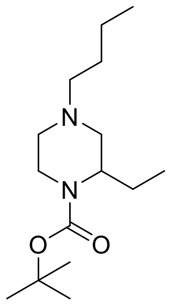

Boc-**1q**

Tert-butyl 2-benzyl-4-butylpiperazine-1-carboxylate (*Boc*-1r). The compound was prepared according to a general procedure (method A2) from dioxime **2r** (60 mg, 0.217 mmol). Yield: 54 mg (75 %). R_f = 0.8 (PE-EtOAc, 1:1). Colorless oil. ^{1}H NMR (300 MHz, $CDCl_3$) δ 7.45 – 7.16 (m, 5H, Ph), 4.21 (m, 1H, C*H*), 4.02 – 3.89 (m, 1H, CH_2NBoc), 3.22 (td, J = 12.9, 3.4 Hz, 1H, CH_2NBoc), 3.10 (dd, J = 13.0, 9.1 Hz, 1H, CH_2Ph), 2.88 (dd, J = 13.0, 6.9 Hz, 1H, CH_2Ph), 2.85 (m, 1H, CH_2NBu), 2.71 (d, J = 11.5 Hz, 1H, CH_2CH), 2.37 (m, 1H, C$H_2$$CH_2CH_2CH_3$), 2.22 (m, 1H, C$H_2$$CH_2CH_2CH_3$), 2.02 (dt, J = 12.5, 6.2 Hz, 1H, CH_2NBu), 1.95 – 1.90 (m, 1H, CH_2CH), 1.51 – 1.43 (m, 4H, CH_2C$H_2$$CH_2CH_3$ + CH_2CH_2C$H_2$$CH_3$), 1.41 (s, 9H, C$H_3$C), 0.95 (t, J = 7.1 Hz, 3H, $CH_2CH_2CH_2$CH_3). ^{13}C{^{1}H} NMR (75 MHz, DEPT135, $CDCl_3$) δ 154.7 (C=O), 139.5 (i-Ph), 129.5 (m-Ph), 128.4 (o-Ph), 126.1 (p-Ph), 79.5 (C), 58.3 (*C*$H_2CH_2CH_2CH_3$), 54.0 (*C*H_2CH), 53.6 (*C*H_2NBu), 53.5

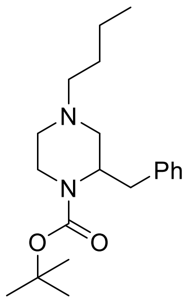

Boc-**1r**

(*C*H), 39.3 (*C*H_2NBoc), 36.2 (*C*H_2Ph), 28.9 (CH_2*C*$H_2CH_2CH_3$), 28.4 (*C*H_3C), 20.6 (CH_2CH_2*C*H_2CH_3), 14.0 ($CH_2CH_2CH_2$*C*H_3). HRMS (ESI): m/z calcd. for $[C_{20}H_{33}N_2O_2]^+$ 333.2537, found 333.2528 $[M + H]^+$.

Hexahydropyrrolo[1,2-a]pyrazin-6(2H)-one (5). To a solution of dioxime **2s** (50 mg, 0.163 mmol) in methanol (2 ml) was added 5%-Pd/C catalyst (50 mg). The vial was placed in a steel autoclave which was flushed and filled with hydrogen to a pressure of ca. 40 bar. Hydrogenation was conducted at this pressure and 50 °C for 5 hours with vigorous stirring. Then, the autoclave was cooled to rt and slowly depressurized, the catalyst was filtered off, and the solution was concentrated under reduced pressure. The residue was dissolved in toluene (2.5 ml), Et_3N (0.25 ml, 1.8 mmol) was added to the solution and left at 90 ^oC for 2.5 hours with vigorous stirring. The residue was subjected to a column chromatography on silica gel (eluent EtOAc : MeOH = 5 : 1 → 3 : 1 → 2 : 1 → 1 : 1) to give 11 mg (48 %) of product **5** as colorless oil. R_f = 0.3 (EtOAc-MeOH, 3:1). ^{1}H NMR spectrum is in agreement with previously published data [12].

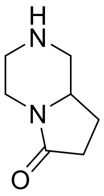

5

3.3 Synthesis and characterization of monooximes 4

To a solution of ene-nitrosoacetal **5** in dichloromethane (1 eqv, 1 M in CH_2Cl_2) was added an amine (10-14 eqv) and the mixture was stirred at room temperature for 24 hours. Then MeOH (5 ml) was added to the mixture and the mixture was stirred at room temperature for 8 hours. The reaction mixture was concentrated in vacuo and the resulting material was subjected to column chromatography (PE:EtOAc = 5:1 → 3:1 → 1:1).

2-(butylamino)acetaldehyde oxime (4a). The compound was prepared according to a general procedure from n-butylamine (4 ml, 40.5 mmol) and ene-nitrosoacetal **3a** (3 ml (1 M in CH_2Cl_2), 3 mmol). Yield: 273 mg (70 %). R_f = 0.13 (PE-EtOAc, 1:1). Colorless oil. Dynamic mixture of isomers, *E*-**4a** : *Z*-**4a** = 1.3 : 1. ^{1}H NMR (300 MHz, $CDCl_3$, *E*-isomer) δ 7.43 (t, J = 5.4 Hz, 1H, CH), 3.34 (d, J = 5.4 Hz, 2H, C*H*$_2$CH), 2.64 – 2.56 (m, 2H, C*H*$_2$CH$_2$CH$_2$CH$_3$), 1.52 – 1.38 (m, 2H, CH$_2$C*H*$_2$CH$_2$CH$_3$), 1.29 (h, J = 7.2 Hz, 2H, CH$_2$CH$_2$C*H*$_2$CH$_3$), 0.85 (t, J = 7.2 Hz, 3H, CH$_2$CH$_2$CH$_2$C*H*$_3$), N-OH and NH protons not observed. ^{13}C{^{1}H} NMR (75 MHz, DEPT135, $CDCl_3$, *E*-isomer) δ 148.5 (CH$_2$*C*H), 48.8 (*C*H$_2$CH$_2$CH$_2$CH$_3$), 48.0 (*C*H$_2$CH), 31.6 (CH$_2$*C*H$_2$CH$_2$CH$_3$), 20.4 (CH$_2$CH$_2$*C*H$_2$CH$_3$), 13.9 (CH$_2$CH$_2$CH$_2$*C*H$_3$).

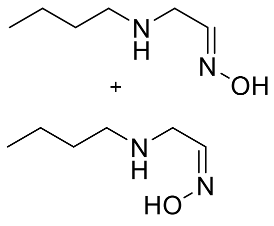

E,*Z*-**4a**

^{1}H NMR (300 MHz, $CDCl_3$, *Z*-isomer) δ 6.78 (t, J = 4.4 Hz, 1H, CH), 3.53 (d, J = 4.4 Hz, 2H, C*H*$_2$CH), 2.64 – 2.56 (m, 2H, C*H*$_2$CH$_2$CH$_2$CH$_3$), 1.52 – 1.38 (m, 2H, CH$_2$C*H*$_2$CH$_2$CH$_3$), 1.29 (h, J = 7.2 Hz, 2H, CH$_2$CH$_2$C*H*$_2$CH$_3$), 0.85 (t, J = 7.2 Hz, 3H, CH$_2$CH$_2$CH$_2$C*H*$_3$), N-OH and NH protons not observed. ^{13}C{^{1}H} NMR (75 MHz, DEPT135, $CDCl_3$, *Z*-isomer) δ 150.0 (CH$_2$*C*H), 49.3 (*C*H$_2$CH$_2$CH$_2$CH$_3$), 44.2 (*C*H$_2$CH), 31.6 (CH$_2$*C*H$_2$CH$_2$CH$_3$), 20.4 (CH$_2$CH$_2$*C*H$_2$CH$_3$), 13.9 (CH$_2$CH$_2$CH$_2$*C*H$_3$).

HRMS (ESI): m/z calcd. for $[C_6H_{15}N_2O]^+$ 131.1179, found 131.1181 $[M + H]^+$.

1-(butylamino)propan-2-one oxime (4b). The compound was prepared according to a general procedure from n-butylamine (1.2 ml, 12 mmol) and ene-nitrosoacetal **3b** (1 ml (1 M in CH_2Cl_2), 1 mmol). Yield: 70 mg (49 %). R_f = 0.11 (PE-EtOAc, 1:1). Colorless oil. Dynamic mixture of isomers, *E*-**4b** : *Z*-**4b** = 7.3 : 1. ^{1}H NMR (300 MHz, $CDCl_3$, *E*-isomer) δ 5.90 (br. s, 1H, NH), 3.30 (s, 2H, C*H*$_2$NH), 2.56 (t, J = 7.1 Hz, 2H,

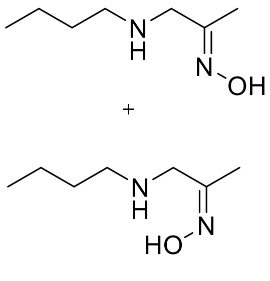

E,*Z*-**4b**

C*H*$_2$CH$_2$CH$_2$CH$_3$), 1.85 (s, 3H, CH$_3$), 1.45 (p, J = 7.1 Hz, 2H, CH$_2$C*H*$_2$CH$_2$CH$_3$), 1.29 (h, J = 7.2 Hz, 2H, CH$_2$CH$_2$C*H*$_2$CH$_3$), 0.86 (t, J = 7.2 Hz, 3H, CH$_2$CH$_2$CH$_2$C*H*$_3$), N-OH-proton not observed. ^{13}C{^{1}H} NMR (75 MHz, DEPT135, CDCl$_3$, *E*-isomer) δ 155.5 (C=N), 53.3 (*C*H$_2$NH), 48.9 (*C*H$_2$CH$_2$CH$_2$CH$_3$), 31.8 (CH$_2$*C*H$_2$CH$_2$CH$_3$), 20.5 (CH$_2$CH$_2$*C*H$_2$CH$_3$), 14.0 (CH$_2$CH$_2$CH$_2$*C*H$_3$), 12.6 (CH$_3$).

^{1}H NMR (300 MHz, CDCl$_3$, *Z*-isomer) δ 5.90 (br. s, 1H, NH), 3.47 (s, 2H, C*H*$_2$NH), 2.56 (t, J = 7.2 Hz, 2H, C*H*$_2$CH$_2$CH$_2$CH$_3$), 1.89 (s, 3H, CH$_3$), 1.45 (p, J = 7.1 Hz, 2H, CH$_2$C*H*$_2$CH$_2$CH$_3$), 1.29 (h, J = 7.2 Hz, 2H, CH$_2$CH$_2$C*H*$_2$CH$_3$), 0.86 (t, J = 7.2 Hz, 3H, CH$_2$CH$_2$CH$_2$C*H*$_3$), N-OH-proton not observed. ^{13}C{^{1}H} NMR (75 MHz, DEPT135, CDCl$_3$, *Z*-isomer) δ 156.2 (C=N), 49.3 (*C*H$_2$CH$_2$CH$_2$CH$_3$), 47.2 (*C*H$_2$NH), 31.8 (CH$_2$*C*H$_2$CH$_2$CH$_3$), 20.5 (CH$_2$CH$_2$*C*H$_2$CH$_3$), 19.6 (CH$_3$), 14.0 (CH$_2$CH$_2$CH$_2$*C*H$_3$).

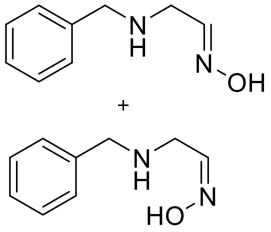

E,*Z*-**4c**

HRMS (ESI): m/z calcd. for [C$_7$H$_{17}$N$_2$O]$^+$ 145.1335, found 145.1336 [M + H]$^+$.

2-(benzylamino)acetaldehyde oxime (4c). The compound was prepared according to a general procedure from benzylamine (3.93 ml, 36 mmol) and ene-nitrosoacetal **3a** (3 ml (1 M in CH$_2$Cl$_2$), 3 mmol). Yield: 128 mg (26 %). 3.18 ml (81 %) of the initial benzylamine was recovered by vacuum distillation. R_f = 0.20 (PE-EtOAc, 1:1). Colorless oil. Dynamic mixture of isomers, *E*-**4c** : *Z*-**4c** = 1.9 : 1. ^{1}H NMR (300 MHz, CDCl$_3$, *E*-isomer) δ 7.53 (t, J = 5.3 Hz, 1H, CH), 7.33 (m, 5H, Ph), 3.84 (s, 2H, CH$_2$), 3.41 (d, J = 5.3 Hz, 2H, CHC*H*$_2$), N-OH and NH protons not observed. ^{13}C{^{1}H} NMR (75 MHz, DEPT135, CDCl$_3$, *E*-isomer) δ 148.9 (CH$_2$*C*H), 139.0 (i-Ph), 128.6 (o-Ph), 128.4 (m-Ph), 127.3 (p-Ph), 53.0 (CH$_2$), 47.3 (CH*C*H$_2$). ^{1}H NMR (300 MHz, CDCl$_3$, *Z*-isomer) δ 7.33 (m, 5H, Ph), 6.89 (t, J = 4.4 Hz, 1H, CH), 3.84 (s, 2H, CH$_2$), 3.61 (d, J = 4.4 Hz, 2H, CHC*H*$_2$), N-OH and NH protons not observed. ^{13}C{^{1}H} NMR (75 MHz, DEPT135, CDCl$_3$, *Z*-isomer) δ

150.4 (CH_2*C*H), 138.9 (i-Ph), 128.6 (o-Ph), 128.4 (m-Ph), 127.4 (p-Ph), 53.6 (CH_2), 43.7 (CH*C*H_2). HRMS (ESI): m/z calcd. for $[C_9H_{13}N_2O]^+$ 165.1022, found 165.1019 $[M + H]^+$.

3.4 Synthesis and characterization of dioximes 2

Synthesis of symmetrically substituted dioximes. Synthesis of dioximes 2a-m,o

To a solution of an amine (1 mmol) in CH_2Cl_2 (1 ml) was added dropwise a solution of ene-nitrosoacetal **5** (2.1 ml, 1M in CH_2Cl_2) and the mixture was left with vigorous stirring at room temperature for 24 hours. Then, MeOH (2 ml) was added to the reaction mixture, and the mixture was left with vigorous stirring for 8 hours. Then the reaction mixture was concentrated under reduced pressure, the residue was subjected to column chromatography.

Synthesis of unsymmetrically substituted dioximes. Synthesis of dioximes 2p-2s

To a solution of monooxime **4** (0.5 mmol) in CH_2Cl_2 (1 ml) was added dropwise a solution of ene-nitrosoacetal **5** (0.6 ml, 1M in CH_2Cl_2) and the mixture was left with vigorous stirring at room temperature for 24 hours. Then, MeOH (2 ml) was added to the reaction mixture, and the mixture was left with vigorous stirring for 8 hours. Then the reaction mixture was concentrated under reduced pressure, and the residue was subjected to column chromatography.

2,2'-(butylazanediyl)diacetaldehyde dioxime (**2a**). The compound was prepared according to a general procedure from ene-nitrosoacetal **3a** (2.1 ml (1 M in CH_2Cl_2), 2.1 mmol) and n-butylamine (0.1 ml, 1 mmol). Yield: 185 mg (99 %). R_f = 0.3 (PE-EtOAc, 1:1). Colorless oil. Dynamic mixture of isomers, *E,E*-**2a** : *E,Z*-**2a** : *Z,Z*-**2a** = 8.9 : 12 : 1.

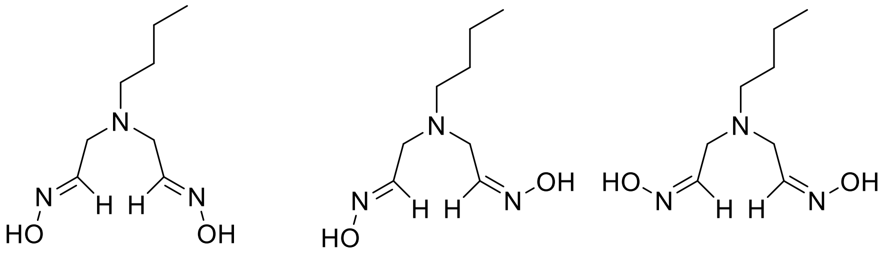

E,E-**2a** *E,Z*-**2a** *Z,Z*-**2a**

^{1}H NMR (300 MHz, HSQC, $CDCl_3$, *E,E*-isomer) δ 8.74 (s, 2H, 2 NOH), 7.48 (t, J = 6.1 Hz, 2H, 2 NC*H*), 3.28 (d, J = 6.1 Hz, 4H, 2 CH_2C=N), 2.66 – 2.50 (m, 2H, C$H_2CH_2CH_2CH_3$), 1.63 – 1.43 (m, 2H, CH_2C$H_2CH_2CH_3$), 1.34 (m, 2H, CH_2CH_2CH_2CH_3), 0.94 (t, J = 7.3 Hz, 3H, $CH_2CH_2CH_2$CH_3). ^{13}C{^{1}H} NMR (75 MHz, DEPT135, $CDCl_3$, *E,E*-isomer) δ 148.9 (2 H*C*=N), 54.1 (*C*$H_2CH_2CH_2CH_3$), 52.1 (2 *C*H_2C=N), 29.0 (CH_2*C*$H_2CH_2CH_3$), 20.4 (CH_2CH_2*C*H_2CH_3), 13.9 ($CH_2CH_2CH_2$*C*H_3). ^{1}H NMR (300 MHz, HSQC, $CDCl_3$, *E,Z*-isomer) δ 8.74 (s, 2H, 2 NOH), 7.49 (t, J = 4.4 Hz, 1H, NC*H*, *E*-fragment), 6.90 (t, J = 4.4 Hz, 1H, NC*H*, *Z*-fragment), 3.47 (d, J = 4.4 Hz, 2H, CH_2C=N, *Z*-fragment), 3.27 (d, J = 4.4 Hz, 2H, CH_2C=N, *E*-fragment), 2.66 – 2.50 (m, 2H, C$H_2CH_2CH_2CH_3$), 1.63 – 1.43 (m, 2H, CH_2C$H_2CH_2CH_3$), 1.34 (m, 2H, CH_2CH_2CH_2CH_3), 0.94 (t, J = 7.3 Hz, 3H, $CH_2CH_2CH_2$CH_3). ^{13}C{^{1}H} NMR (75 MHz, HSQC, DEPT135, $CDCl_3$, *E,Z*-isomer) δ 150.0 and 148.6 (2 H*C*=N), 54.9 (*C*$H_2CH_2CH_2CH_3$), 53.1 (*C*H_2C=N, *E*-fragment), 48.9 (*C*H_2C=N, *Z*-fragment), 29.0 (CH_2*C*$H_2CH_2CH_3$), 20.4 (CH_2CH_2*C*H_2CH_3), 13.9 ($CH_2CH_2CH_2$*C*H_3). ^{1}H NMR (300 MHz, $CDCl_3$, *Z,Z*-isomer) δ 8.73 (s, 2H, 2 NOH), 6.90 (t, J = 4.4 Hz, 2H, 2 NC*H*), 3.48 (d, J = 4.4 Hz, 4H, 2 CH_2C=N), 2.66 – 2.50 (m, 2H, C$H_2CH_2CH_2CH_3$), 1.63 – 1.43 (m, 2H, CH_2C$H_2CH_2CH_3$), 1.34 (m, 2H, CH_2CH_2CH_2CH_3), 0.94 (t, J = 7.3 Hz, 3H, $CH_2CH_2CH_2$CH_3). ^{13}C{^{1}H} NMR (75 MHz, HSQC, DEPT135, $CDCl_3$, *Z,Z*-isomer, characteristic signals) δ 148.9 (2 H*C*=N), 54.9 (*C*$H_2CH_2CH_2CH_3$), 49.4 (2 *C*H_2C=N). ^{15}N NMR (300 MHz, HMBC, $CDCl_3$, all isomers) δ -22.0 (C*N*H). ^{1}H NMR (300 MHz, DMSO-d_6, *E,E*-isomer) δ 10.69 (s, 2H, 2 NOH), 7.28 (t, J = 6.0 Hz, 2H, 2 NC*H*), 3.13 (d, J = 6.0 Hz, 4H, 2 CH_2C=N), 2.46 – 2.35 (m, 2H, C$H_2CH_2CH_2CH_3$), 1.45 – 1.32 (m, 2H, CH_2C$H_2CH_2CH_3$), 1.32-1.17 (m, 2H, CH_2CH_2CH_2CH_3), 0.86 (t, J = 7.3 Hz, 3H, $CH_2CH_2CH_2$CH_3). Characteristic

correlations in 2D NOESY: NOH/N=C*H*. ^{1}H NMR (300 MHz, DMSO-d_6, *E,Z*-isomer) δ 10.95 (s, 1H, NOH, Z-fragment), 10.70 (s, 1H, NOH, E-fragment), 7.29 (t, J = 6.0 Hz, 1H, NC*H*, E-fragment), 6.74 (t, J = 4.4 Hz, 1H, NC*H*, Z-fragment), 3.29 (d, J = 4.4 Hz, 2H, CH_2C=N, Z-fragment), 3.13 (d, J = 6.0 Hz, 2H, CH_2C=N, E-fragment), 2.46 – 2.35 (m, 2H, C$H_2CH_2CH_2CH_3$), 1.45 – 1.32 (m, 2H, $CH_2CH_2CH_2CH_3$), 1.32-1.17 (m, 2H, $CH_2CH_2CH_2CH_3$), 0.86 (t, J = 7.3 Hz, 3H, $CH_2CH_2CH_2CH_3$). ^{1}H NMR (300 MHz, DMSO-d_6, *Z,Z*-isomer) δ 10.96 (s, 2H, 2 NOH), 6.76 (t, J = 4.4 Hz, 2H, 2 NC*H*), 3.30 (d, J = 4.4 Hz, 4H, 2 CH_2C=N), 2.46 – 2.35 (m, 2H, C$H_2CH_2CH_2CH_3$), 1.45 – 1.32 (m, 2H, $CH_2CH_2CH_2CH_3$), 1.32-1.17 (m, 2H, $CH_2CH_2CH_2CH_3$), 0.86 (t, J = 7.3 Hz, 3H, $CH_2CH_2CH_2CH_3$). HRMS (ESI): m/z calcd. for $[C_8H_{18}N_3O_2]^+$ 188.1399, found 188.1394 $[M + H]^+$.

2,2'-(tert-butylazanediyl)diacetaldehyde dioxime (**2b**). The compound was prepared according to a general procedure from ene-nitrosoacetal **3a** (2.1 ml (1 M in CH_2Cl_2), 2.1 mmol) and tert-butylamine (0.106 ml, 1 mmol). Yield: 90 mg (48 %). R_f = 0.4 (PE-EtOAc, 1:1). Colorless oil. Dynamic mixture of isomers, *E,E*-**2b** : *E,Z*-**2b** : *Z,Z*-**2b** = 1 : 1.5 : 1.6.

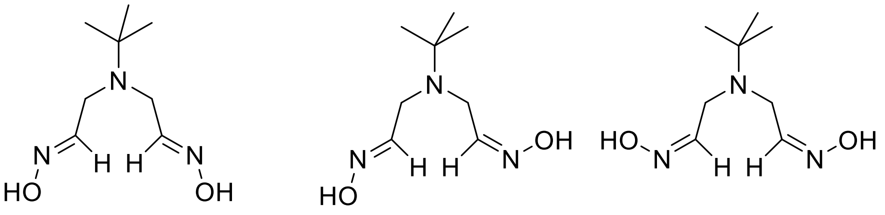

E,E-**2b** *E,Z*-**2b** *Z,Z*-**2b**

^{1}H NMR (300 MHz, $CDCl_3$, *E,E*-isomer) δ 7.36 (t, J = 6.1 Hz, 2H, 2 NC*H*), 3.34 (d, J = 6.1 Hz, 4H, 2 CH_2C=N), 1.12 (s, 9H, 3 CH_3), N-OH-protons not observed. ^{13}C{^{1}H} NMR (75 MHz, DEPT135, $CDCl_3$, *E,E*-isomer) δ 150.2 (2 H*C*=N), 55.9 (C), 47.8 (2 *C*H_2C=N), 27.5 (3 CH_3). ^{1}H NMR (300 MHz, $CDCl_3$, *E,Z*-isomer) δ 7.40 (t, J = 6.1 Hz, 1H, NC*H*, *E*-fragment), 6.80 (t, J = 4.3 Hz, 1H, NC*H*, *Z*-fragment), 3.51 (d, J = 4.3 Hz, 2H, CH_2C=N, *Z*-fragment), 3.31 (d, J = 6.1 Hz, 2H, CH_2C=N, *E*-fragment), 1.12 (s, 9H, 3 CH_3), N-OH-protons not observed. ^{13}C{^{1}H} NMR (75 MHz, DEPT135, $CDCl_3$, *E,Z*-isomer) δ 152.2 and 150.7 (2 H*C*=N), 55.4

(C), 49.2 (*C*H_2C=N, *E*-fragment), 44.5 (*C*H_2C=N, *Z*-fragment), 27.0 (3 CH_3). ^{1}H NMR (300 MHz, $CDCl_3$, *Z,Z*-isomer) δ 6.88 (t, J = 4.3 Hz, 2H, 2 NC*H*), 3.53 (d, J = 4.3 Hz, 4H, 2 C*H_2*C=N), 1.12 (s, 9H, 3 C*H_3*), N-OH-protons not observed. ^{13}C{^{1}H} NMR (75 MHz, DEPT135, $CDCl_3$, *Z,Z*-isomer, characteristic signals) δ 152.2 (2 H*C*=N), 55.7 (C), 45.6 (2 *C*H_2C=N), 26.8 (3 CH_3). HRMS (ESI): m/z calcd. for $[C_8H_{18}N_3O_2]^+$ 188.1394, found 188.1389 $[M + H]^+$.

2,2'-(cyclopentylazanediyl)diacetaldehyde dioxime (**2c**). The compound was prepared according to a general procedure from ene-nitrosoacetal **3a** (2.1 ml (1 M in CH_2Cl_2), 2.1 mmol) and cyclopentylamine (0.1 ml, 1 mmol). Yield: 144 mg (72 %). R_f = 0.3 (PE-EtOAc, 1:1). Colorless oil. Dynamic mixture of isomers, *E,E*-**2c** : *E,Z*-**2c** : *Z,Z*-**2c** = 3.4 : 3.8 : 1.

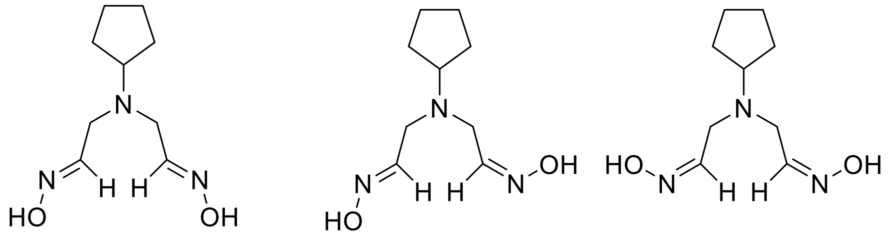

E,E-**2c** *E,Z*-**2c** *Z,Z*-**2c**

^{1}H NMR (300 MHz, $CDCl_3$, *E,E*-isomer) δ 10.14 (s, 2H, 2 NOH), 7.47 (t, J = 6.1 Hz, 2H, 2 NC*H*), 3.34 (d, J = 6.1 Hz, 4H, 2 C*H_2*C=N), 3.03 (m, 1H, CH_2CH_2C*H*), 1.96 – 1.76 (m, 2H, CH_2C*H_2*CH), 1.76 – 1.54 (m, 2H, CH_2C*H_2*CH), 1.60 – 1.33 (m, 4H, 2 C*H_2*CH_2CH). ^{13}C{^{1}H} NMR (75 MHz, DEPT135, $CDCl_3$, *E,E*-isomer) δ 148.5 (2 H*C*=N), 64.1 (CH_2CH_2*C*H), 50.6 (2 *C*H_2C=N), 30.4 (CH_2*C*H_2CH), 24.1 (*C*H_2CH_2CH). ^{1}H NMR (300 MHz, $CDCl_3$, *E,Z*-isomer) δ 10.14 (s, 2H, 2 NOH), 7.50 (t, J = 6.1 Hz, 1H, NC*H*, *E*-fragment), 6.92 (t, J = 4.4 Hz, 1H, NC*H*, *Z*-fragment), 3.53 (d, J = 4.4 Hz, 2H, C*H_2*C=N, *Z*-fragment), 3.34 (d, J = 6.1 Hz, 2H, C*H_2*C=N, *E*-fragment), 3.03 (m, 1H, CH_2CH_2C*H*), 1.96 – 1.76 (m, 2H, CH_2C*H_2*CH), 1.76 – 1.54 (m, 2H, CH_2C*H_2*CH), 1.60 – 1.33 (m, 4H, 2 C*H_2*CH_2CH). ^{13}C{^{1}H} NMR (75 MHz, DEPT135, $CDCl_3$, *E,Z*-isomer) δ 150.2 and 148.3 (2 H*C*=N), 64.9 (CH_2CH_2*C*H), 52.0 (*C*H_2C=N, *E*-fragment), 47.0 (*C*H_2C=N,

Z-fragment), 30.2 (CH_2*C*H_2CH), 24.1 (*C*H_2CH_2CH). ^{1}H NMR (300 MHz, $CDCl_3$, *Z,Z*-isomer) δ 10.14 (s, 2H, 2 NOH), 6.96 (t, J = 4.4 Hz, 2H, 2 NC*H*), 3.54 (d, J = 4.4 Hz, 4H, 2 C*H_2*C=N), 3.03 (m, 1H, CH_2CH_2C*H*), 1.96 – 1.76 (m, 2H, CH_2C*H_2*CH), 1.76 – 1.54 (m, 2H, CH_2C*H_2*CH), 1.60 – 1.33 (m, 4H, 2 C*H_2*CH_2CH). ^{13}C{^{1}H} NMR (75 MHz, DEPT135, $CDCl_3$, *Z,Z*-isomer) δ 149.8 (2 H*C*=N), 65.3 (CH_2CH_2*C*H), 47.9 (2 *C*H_2C=N), 30.1 (CH_2*C*H_2CH), 24.0 (*C*H_2CH_2CH). HRMS (ESI): m/z calcd. for $[C_9H_{18}N_3O_2]^+$ 200.1394, found 200.1392 $[M + H]^+$.

2,2'-(allylazanediyl)diacetaldehyde dioxime (**2d**). The compound was prepared according to a general procedure from ene-nitrosoacetal **3a** (2.1 ml (1 M in CH_2Cl_2), 2.1 mmol) and allylamine (0.075 ml, 1 mmol). Yield: 64 mg (37 %). R_f = 0.5 (PE-EtOAc, 1:1). Colorless oil. Dynamic mixture of isomers, *E,E*-**2d** : *E,Z*-**2d** : *Z,Z*-**2d** = 3.7 : 6.1 : 1.

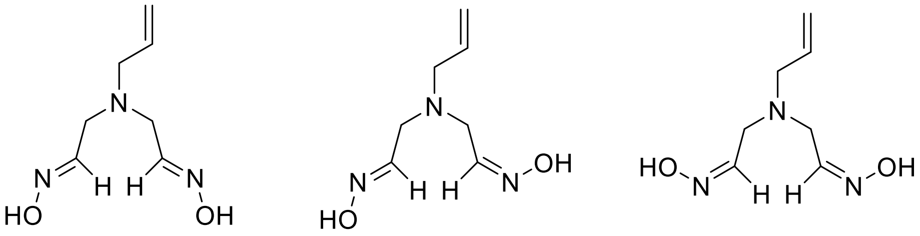

E,E-**2d** *E,Z*-**2d** *Z,Z*-**2d**

^{1}H NMR (300 MHz, $CDCl_3$, *E,E*-isomer) δ 8.73 (s, 2H, 2 NOH), 7.45 (t, J = 6.1 Hz, 2H, 2 NC*H*), 5.84 (m, 1H, NCH_2C*H*CH_2), 5.34 – 5.17 (m, 2H, NCH_2CHC*H_2*), 3.27 (d, J = 6.1 Hz, 4H, 2 C*H_2*C=N), 3.19 (d, J = 6.6 Hz, 2H, C*H_2*CHCH_2). ^{13}C{^{1}H} NMR (75 MHz, DEPT135, $CDCl_3$, *E,E*-isomer) δ 149.0 (2 H*C*=N), 134.4 (NCH_2*C*HCH_2), 119.2 (NCH_2CH*C*H_2), 57.6 (N*C*H_2CHCH_2), 51.9 (2 *C*H_2C=N). ^{1}H NMR (300 MHz, $CDCl_3$, *E,Z*-isomer) δ 8.73 (s, 2H, 2 NOH), 7.45 (t, J = 6.1 Hz, 1H, NC*H*, *E*-fragment), 6.87 (t, J = 6.1 Hz, 1H, NC*H*, *Z*-fragment), 5.84 (m, 1H, NCH_2C*H*CH_2), 5.34 – 5.17 (m, 2H, NCH_2CHC*H_2*), 3.47 (d, J = 6.1 Hz, 2H, C*H_2*C=N, *Z*-fragment), 3.27 (d, J = 6.1 Hz, 2H, C*H_2*C=N, *E*-fragment), 3.19 (d, J = 6.6 Hz, 2H, NC*H_2*CHCH_2). ^{13}C{^{1}H} NMR (75 MHz, DEPT135, $CDCl_3$, *E,Z*-

isomer) δ 151.0 and 148.7 (2 H*C*=N), 134.0 (NCH_2*C*HCH_2), 119.3 (NCH_2CH*C*H_2), 57.6 (N*C*H_2CHCH_2), 52.8 (*C*H_2C=N, *E*-fragment), 48.3 (*C*H_2C=N, *Z*-fragment). ^{1}H NMR (300 MHz, $CDCl_3$, *Z,Z*-isomer) δ 8.73 (s, 2H, 2 NOH), 7.56 (t, J = 4.4 Hz, 2H, 2 NC*H*), 5.84 (m, 1H, NCH_2C*H*CH_2), 4.68 – 4.61 (m, 2H, NCH_2CHC*H*$_2$), 4.28 (d, J = 4.4 Hz, 4H, 2 C*H*$_2$C=N), 3.19 (d, J = 6.6 Hz, 2H, NC*H*$_2CHCH_2$). $^{13}C\{^1H\}$ NMR (75 MHz, DEPT135, $CDCl_3$, *Z,Z*-isomer, characteristic signals) δ 148.9 (2 H*C*=N), 48.2 (2 *C*H_2C=N). HRMS (ESI): m/z calcd. for $[C_7H_{14}N_3O_2]^+$ 172.1081, found 172.1085 $[M + H]^+$.

2,2'-(benzylazanediyl)diacetaldehyde dioxime (**2e**). The compound was prepared according to a general procedure from ene-nitrosoacetal **3a** (1.93 ml (1 M in CH_2Cl_2), 1.93 mmol) and benzylamine (98 mg, 0.92 mmol). Yield: 143 mg (70 %). R_f = 0.50 (PE-EtOAc, 1:1). Colorless oil. Dynamic mixture of isomers, *E,E*-**2e** : *E,Z*-**2e** : *Z,Z*-**2e** = 3.8 : 7.3 : 1.

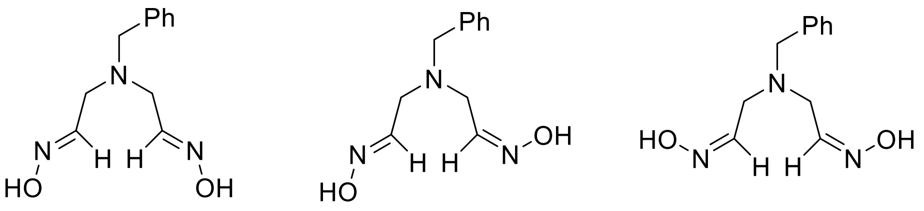

E,E-**2e** *E,Z*-**2e** *Z,Z*-**2e**

^{1}H NMR (300 MHz, $CDCl_3$, *E,E*-isomer) δ 8.87 (s, 2H, 2 NOH), 7.45 (t, J = 6.0 Hz, 2H, 2 NC*H*), 7.36 – 7.23 (m, 5H, Ph), 3.68 (s, 2H, C*H*$_2$Ph), 3.26 (d, J = 6.0 Hz, 4H, 2 C*H*$_2$C=N). $^{13}C\{^1H\}$ NMR (75 MHz, DEPT135, $CDCl_3$, *E,E*-isomer) δ 149.1 (2 *C*=N), 137.6 (i-Ph), 129.0 (o-Ph), 128.5 (m-Ph), 127.5 (p-Ph), 58.6 (*C*H_2Ph), 51.8 (2 *C*H_2C=N). ^{1}H NMR (300 MHz, $CDCl_3$, *E,Z*-isomer) δ 9.29 (s, 1H, NOH, *Z*-fragment), 8.87 (s, 1H, NOH, *E*-fragment), 7.46 (t, J = 6.0 Hz, 1H, NC*H*, *E*-fragment), 7.36 – 7.23 (m, 5H, Ph), 6.87 (t, J = 4.5 Hz, 1H, NC*H*, *Z*-fragment), 3.68 (s, 2H, C*H*$_2$Ph), 3.47 (d, J = 4.5 Hz, 2H, C*H*$_2$C=N, *Z*-fragment), 3.24 (d, J = 6.0 Hz, 2H, C*H*$_2$C=N, *E*-fragment). $^{13}C\{^1H\}$ NMR (75 MHz, DEPT135, $CDCl_3$, *E,Z*-isomer) δ 150.4 and 148.9 (2 *C*=N), 137.4 (i-Ph), 129.1 (o-Ph), 128.5 (m-Ph), 127.6 (p-Ph), 59.4 (*C*H_2Ph), 52.8 (*C*H_2C=N, *E*-fragment), 48.4 (*C*H_2C=N, *Z*-

fragment). ^{1}H NMR (300 MHz, $CDCl_3$, *Z,Z*-isomer) δ 9.29 (s, 2H, 2 NOH), 7.36 – 7.23 (m, 5H, Ph), 6.91 (t, J = 4.5 Hz, 2H, 2 NC*H*), 3.68 (s, 2H, CH_2Ph), 3.47 (d, J = 4.5 Hz, 4H, 2 CH_2C=N). ^{13}C{^{1}H} NMR (75 MHz, DEPT135, $CDCl_3$, *Z,Z*-isomer, characteristic signals) δ 150.1 (2 *C*=N), 137.3 (i-Ph), 129.2 (o-Ph), 128.6 (m-Ph), 127.7 (p-Ph), 59.8 (*C*H_2Ph), 49.0 (2 *C*H_2C=N). HRMS (ESI): m/z calcd. for $[C_{11}H_{16}N_3O_2]^+$ 222.1237, found 222.1241 $[M + H]^+$.

2,2'-(((S)-1-phenylethyl)azanediyl)diacetaldehyde dioxime (**2f**). The compound was prepared according to a general procedure from ene-nitrosoacetal **3a** (2.1 ml (1 M in CH_2Cl_2), 2.1 mmol) and α-phenylethylamine (0.127 ml, 1 mmol). Yield: 193 mg (82 %). R_f = 0.6 (PE-EtOAc, 1:1). Colorless oil. $[\alpha]_D$ = –40.9 (c=2, MeOH, 26 ^{o}C). Dynamic mixture of isomers, *E,E*-**2f** : *E,Z*-**2f** : *Z,Z*-**2f** = 3.2 : 3.8 : 1.

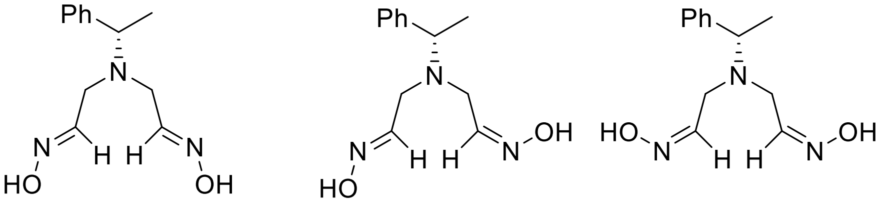

E,E-**2f** *E,Z*-**2f** *Z,Z*-**2f**

^{1}H NMR (300 MHz, $CDCl_3$, *E,E*-isomer) δ 9.79 (s, 2H, 2 NOH), 7.38-7.21 (m, 5H, Ph), 7.42 (t, J = 6.2 Hz, 2H, 2 NC*H*), 3.96-3.84 (m, 1H, PhC*H*CH_3), 3.38 – 3.15 (m, 4H, 2 CH_2C=N), 1.43 (d, J = 3.4 Hz, 3H, CH_3). ^{13}C{^{1}H} NMR (75 MHz, DEPT135, $CDCl_3$, *E,E*-isomer) δ 149.7 (2 H*C*=N), 142.6 (i-Ph), 127.7 and 127.5 (o-Ph and m-Ph), 125.9 (p-Ph), 60.2 (Ph*C*HCH_3), 49.0 (2 *C*H_2C=N), 19.8 (*C*H_3). ^{1}H NMR (300 MHz, $CDCl_3$, *E,Z*-isomer) δ 9.79 (s, 2H, 2 NOH), 7.44 (t, J = 6.2 Hz, 1H, NC*H*, *E*-fragment), 7.38-7.21 (m, 5H, Ph), 6.87 (t, J = 4.4 Hz, 1H, NC*H*, *Z*-fragment), 3.96-3.84 (m, 1H, PhC*H*CH_3), 3.63-3.40 (m, 2H, CH_2C=N, *Z*-fragment), 3.38 – 3.15 (m, 2H, CH_2C=N, *E*-fragment), 1.41 (d, J = 3.2 Hz, 3H, CH_3). ^{13}C{^{1}H} NMR (75 MHz, DEPT135, $CDCl_3$, *E,Z*-isomer) δ 151.2 and 149.4 (2 H*C*=N), 142.3 (i-Ph), 129.6 (p-Ph), 128.5 and 127.8 (o-Ph and m-Ph), 61.0 (Ph*C*HCH_3), 50.3 (*C*H_2C=N, *E*-fragment), 45.5 (*C*H_2C=N, *Z*-fragment), 17.8

(*C*H_3). ^{1}H NMR (300 MHz, $CDCl_3$, *Z,Z*-isomer) δ 9.79 (s, 2H, 2 NOH), 7.38-7.21 (m, 5H, Ph), 6.91 (t, J = 4.4 Hz, 2H, 2 NC*H*), 3.96-3.84 (m, 1H, PhC*H*CH_3), 3.63-3.40 (m, 4H, 2 C*H*$_2$C=N), 1.41 (d, J = 3.2 Hz, 3H, C*H*$_3$). $^{13}C\{^1H\}$ NMR (75 MHz, DEPT135, $CDCl_3$, *Z,Z*-isomer) δ 150.9 (2 H*C*=N), 136.5 (i-Ph), 128.4 and 127.4 (o-Ph and m-Ph), 127.5 (p-Ph), 61.4 (Ph*C*HCH_3), 46.4 (2 *C*H_2C=N), 18.0 (*C*H_3). HRMS (ESI): m/z calcd. for $[C_{12}H_{18}N_3O_2]^+$ 236.1394, found 236.1393 $[M + H]^+$.

Ethyl bis(2-(hydroxyimino)ethyl)glycinate (**2g**). The compound was prepared according to a general procedure from ene-nitrosoacetal **3a** (2.1 ml (1 M in CH_2Cl_2), 2.1 mmol) and ethyl glycinate (103 mg, 1 mmol). Yield: 72 mg (33 %). R_f = 0.4 (PE-EtOAc, 1:1). Colorless oil. Dynamic mixture of isomers, *E*,*E*-**2g** : *E*,*Z*-**2g** : *Z*,*Z*-**2g** = 6.6 : 6.9 : 1.

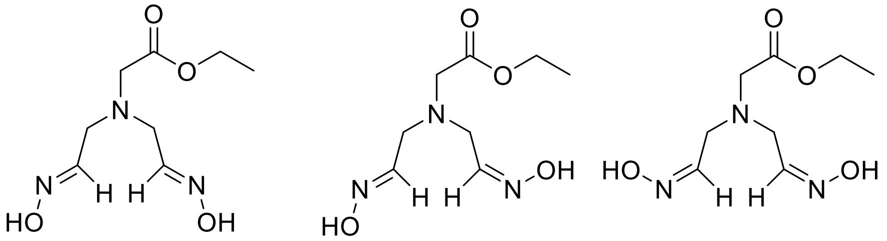

E,E-**2g** *E,Z*-**2g** *Z,Z*-**2g**

^{1}H NMR (300 MHz, DMSO-d6, *E,E*-isomer) δ 10.75 (s, 2H, 2 NOH), 7.27 (t, J = 6.1, 2H, 2 NC*H*), 4.07 (q, J = 7.1 Hz, 2H, C*H*$_2$$CH_3$), 3.40 (s, 2H, NC*H*$_2$C=O), 3.30 (d, J = 6.1 Hz, 4H, 2 C*H*$_2$C=N), 1.18 (t, J = 7.1 Hz, 3H, CH_2C*H*$_3$). $^{13}C\{^1H\}$ NMR (75 MHz, DEPT135, DMSO-d6, *E,E*-isomer) δ 170.5 (C=O), 147.1 (2 H*C*=N), 60.0 (*C*$H_2$$CH_3$), 53.7 (N*C*$H_2$C=O), 52.1 (2 *C*$H_2$C=N), 14.2 ($CH_2$*C*$H_3$). [1]H NMR (300 MHz, DMSO-d6, *E,Z*-isomer) δ 10.97 and 10.75 (s, 2H, 2 NOH), 7.28 (t, J = 6.1 Hz, 1H, NC*H*, *E*-fragment), 6.77 (t, J = 4.3 Hz, 1H, NC*H*, *Z*-fragment), 4.07 (q, J = 7.1 Hz, 2H, C*H*$_2$$CH_3$), 3.48 (d, J = 4.3 Hz, 2H, C*H*$_2$C=N, *Z*-fragment), 3.40 (s, 2H, NC*H*$_2$C=O), 3.37 (d, J = 6.1 Hz, 2H, C*H*$_2$C=N, *E*-fragment), 1.18 (t, J = 7.1 Hz, 3H, CH_2C*H*$_3$). $^{13}C\{^1H\}$ NMR (75 MHz, DEPT135, DMSO-d6, *E,Z*-isomer) δ 170.5 (C=O), 149.1 and 147.0 (2 H*C*=N), 60.0 (*C*$H_2$$CH_3$), 54.5 (N*C*$H_2$C=O), 52.6 (*C*$H_2$C=N, *E*-fragment), 48.4 (*C*H_2C=N, *Z*-fragment), 14.2 (CH_2*C*H_3). ^{1}H NMR

(300 MHz, DMSO-d6, *Z,Z*-isomer) δ 10.98 (s, 2H, 2NOH), 6.84 – 6.70 (t, J = 4.3 Hz, 2H, 2 NC*H*), 4.07 (q, J = 7.1 Hz, 2H, C*H*$_2$CH$_3$), 3.42 (d, J = 4.3 Hz, 4H, 2 C*H*$_2$C=N), 3.40 (s, 2H, NC*H*$_2$C=O), 1.18 (t, J = 7.1 Hz, 3H, CH$_2$C*H*$_3$). ^{13}C{^{1}H} NMR (75 MHz, DEPT135, DMSO-d6, *Z,Z*-isomer, characteristic signals) δ 170.5 (C=O), 149.0 (2 H*C*=N), 60.0 (*C*H$_2$CH$_3$), 48.8 (2 *C*H$_2$C=N), 14.2 (CH$_2$*C*H$_3$). HRMS (ESI): m/z calcd. for $[C_8H_{16}N_3O_4]^+$ 218.1135, found 218.1144 $[M + H]^+$.

Ethyl bis(2-(hydroxyimino)ethyl)-L-leucinate (**2h**). The compound was prepared according to a general procedure from ene-nitrosoacetal **3a** (2.1 ml (1 M in CH$_2$Cl$_2$), 2.1 mmol) and ethyl L-leucinate (159 mg, 1 mmol). Yield: 173 mg (63 %). R_f = 0.51 (PE-EtOAc, 1:1). Colorless oil. $[\alpha]_D$ = –25.2 (c=1, MeOH, 26 °C). Dynamic mixture of isomers, *E,E*-**2h** : *E,Z*-**2h** : *Z,Z*-**2h** = 2.1 : 2.7 : 1.

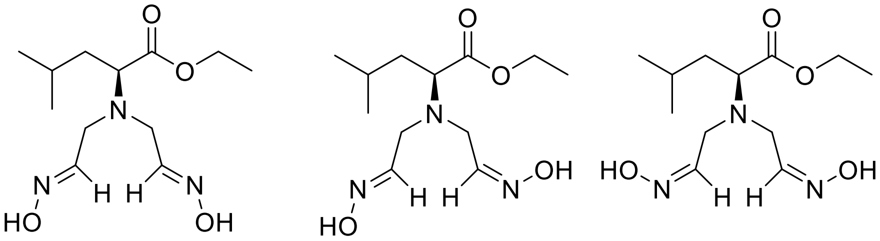

E,E-**2h** *E,Z*-**2h** *Z,Z*-**2h**

^{1}H NMR (300 MHz, CDCl$_3$, *E,E*-isomer) δ 9.50 (s, 2H, 2 NOH), 7.32 (t, J = 5.5 Hz, 2H, 2 NC*H*), 4.17 (qd, J = 7.1, 3.4 Hz, 2H, C*H*$_2$CH$_3$), 3.41 (d, 4H, 2 C*H*$_2$C=N), 1.68 (m, 1H, CH$_3$C*H*CH$_3$), 1.52 (m, 3H, CHC*H*$_2$CHCO and CHC*H*$_2$C*H*CO), 1.26 (t, J = 7.1 Hz, 3H, CH$_2$C*H*$_3$), 0.95 – 0.83 (m, 6H, CH(C*H*$_3$)$_2$). ^{13}C{^{1}H} NMR (75 MHz, DEPT135, CDCl$_3$, *E,E*-isomer) δ 173.5 (*C*=O), 149.4 (2 H*C*=N), 61.0 (*C*H$_2$CH$_3$), 59.3 (*C*HCO), 49.7 (2 *C*H$_2$CHNOH), 38.8 (CH*C*H$_2$CH), 24.9 (*C*H(CH$_3$)$_2$), 23.0 (CH(*C*H$_3$)$_2$), 14.3 (CH$_2$*C*H$_3$). ^{1}H NMR (300 MHz, CDCl$_3$, *E,Z*-isomer) δ 9.50 (s, 2H, 2 NOH), 7.42 (t, J = 5.5 Hz, 1H, NC*H*, *E*-fragment), 6.74 (t, J = 4.4 Hz, 1H, NC*H*, *Z*-fragment), 4.17 (qd, J = 7.1, 3.4 Hz, 2H, C*H*$_2$CH$_3$), 3.58 (d, J = 4.4 Hz, 2H, C*H*$_2$C=N, *Z*-fragment), 3.27 (d, J = 5.5 Hz, 2H, C*H*$_2$C=N, *E*-fragment), 1.68 (m, 1H, CH$_3$C*H*CH$_3$), 1.52 (m, 3H, CHC*H*$_2$CHCO and CHC*H*$_2$C*H*CO), 1.26 (t, J = 7.1 Hz, 3H, CH$_2$C*H*$_3$), 0.95 – 0.83 (m, 6H, CH(C*H*$_3$)$_2$).

$^{13}C\{^1H\}$ NMR (75 MHz, DEPT135, $CDCl_3$, *E,Z*-isomer) δ 173.3 (*C*=O), 151.5 and 149.1 (2 H*C*=N), 60.7 ($\mathit{C}H_2CH_3$), 59.3 (*C*HCO), 50.9 ($\mathit{C}H_2$CHNOH, *E*-fragment), 42.5 ($\mathit{C}H_2$CHNOH, *Z*-fragment), 38.8 (CH$\mathit{C}H_2$CH), 24.7 (*C*H($CH_3)_2$), 22.6 and 22.5 (CH($\mathit{C}H_3)_2$), 14.3 ($CH_2\mathit{C}H_3$). ^1H NMR (300 MHz, $CDCl_3$, *Z,Z*-isomer) δ 9.50 (s, 2H, 2 NOH), 6.8 (t, J = 4.4 Hz, 2H, 2 NC*H*), 4.17 (qd, J = 7.1, 3.4 Hz, 2H, $\mathit{CH_2}CH_3$), 3.64 (t, J = 4.4 Hz, 4H, 2 $C\mathit{H_2}$C=N), 1.68 (m, 1H, CH_3C*H*CH_3), 1.52 (m, 3H, CH$C\mathit{H_2}$CHCO and CH$C\mathit{H_2}$C*H*CO), 1.26 (t, J = 7.1 Hz, 3H, $CH_2C\mathit{H_3}$), 0.95 – 0.83 (m, 6H, CH($C\mathit{H_3})_2$). $^{13}C\{^1H\}$ NMR (75 MHz, DEPT135, $CDCl_3$, *Z,Z*-isomer) δ 173.2 (*C*=O), 151.2 (2 H*C*=N), 60.6 ($\mathit{C}H_2CH_3$), 59.9 (*C*HCO), 42.9 (2 $\mathit{C}H_2$CHNOH), 38.8 (CH$\mathit{C}H_2$CH), 24.6 (*C*H($CH_3)_2$), 22.6 and 22.5 (CH($\mathit{C}H_3)_2$), 14.4 ($CH_2\mathit{C}H_3$). HRMS (ESI): m/z calcd. for $[C_{12}H_{24}N_3O_4]^+$ 274.1761, found 274.1763 $[M + H]^+$.

Ethyl (2-(hydroxyimino)ethyl)-L-leucinate (4d). Monooxime was isolated from an experiment to produce dioxime **2h** from ene-nitrosoacetal **3a** (2.1 ml (1 M in CH_2Cl_2), 2.1 mmol) and ethyl L-leucinate (159 mg, 1 mmol) as an impurity. Yield: 100 mg (20 %). R_f = 0.51 (EtOAc-MeOH, 1:1). Colorless oil. Dynamic mixture of isomers, *E*-**4d** : *Z*-**4d** = 2.5 : 1. ***Cis*-isomer** ^1H NMR (300 MHz, $CDCl_3$) δ 7.10 (t, J = 4.4 Hz, 1H, HONC*H*), 4.26 (q, J = 7.1 Hz, 2H, $C\mathit{H_2}CH_3$), 3.64 (d, J = 4.4 Hz, 2H, $C\mathit{H_2}$CHNOH), 1.88 – 1.68 (m, 3H, CH$C\mathit{H_2}$CH and C*H*CO), 1.68 – 1.48 (m, 1H, CH_3C*H*CH_3), 1.32 (t, J = 7.1 Hz, 3H, $CH_2C\mathit{H_3}$), 0.96 (d, J = 7.6 Hz, 6H, CH($C\mathit{H_3})_2$). N-OH and NH protons not observed. $^{13}C\{^1H\}$ NMR (75 MHz, DEPT135, $CDCl_3$) δ 145.2 (H*C*=N), 62.3 ($\mathit{C}H_2CH_3$), 59.5 (*C*HCO), 44.2 ($\mathit{C}H_2$CHNOH), 40.1 (CH$\mathit{C}H_2$CH), 25.0 (*C*H($CH_3)_2$), 22.8 and 22.0 (CH($\mathit{C}H_3)_2$), 14.3 ($CH_2\mathit{C}H_3$). *C*=O atom not observed. ***Trans*-isomer** ^1H NMR (300 MHz, $CDCl_3$) δ 7.56 (t, J = 5.2 Hz, 1H, HONC*H*), 4.26 (q, J = 7.1 Hz, 2H, $C\mathit{H_2}CH_3$), 3.64 (d, J = 5.2 Hz, 2H, $C\mathit{H_2}$CHNOH), 1.88 – 1.68 (m, 3H, CH$C\mathit{H_2}$CH and

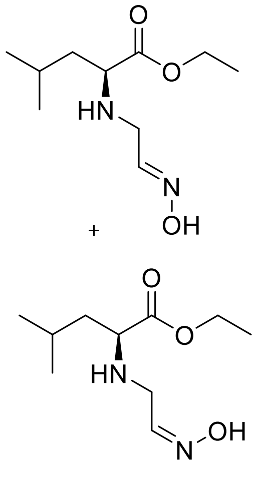

E,Z-**4d**

C*H*CO), 1.68 – 1.48 (m, 1H, CH_3CHCH_3), 1.32 (t, J = 7.1 Hz, 3H, CH_2CH_3), 0.96 (d, J = 7.6 Hz, 6H, $CH(CH_3)_2$). N-OH and NH protons not observed. $^{13}C\{^1H\}$ NMR (75 MHz, DEPT135, $CDCl_3$) δ 144.7 (H*C*=N), 62.2 (CH_2CH_3), 58.9 (*C*HCO), 45.5 (CH_2CHNOH), 40.0 ($CHCH_2CH$), 25.0 ($CH(CH_3)_2$), 22.9 and 22.0 ($CH(CH_3)_2$), 14.3 (CH_2CH_3). *C*=O atom not observed. HRMS (ESI): m/z calcd. for $[C_{10}H_{21}N_2O_3]^+$ 217.1547, found 217.1551 $[M + H]^+$.

1,1'-(butylazanediyl)bis(propan-2-one) dioxime (**2i**). The compound was prepared according to a general procedure from ene-nitrosoacetal **3b** (2.1 ml (1 M in CH_2Cl_2), 2.1 mmol) and n-butylamine (0.1 ml, 1 mmol). Yield: 197 mg (91 %). R_f = 0.54 (PE-EtOAc, 1:1). Colorless oil. Dynamic mixture of isomers, *E,E*-**2i** : *E,Z*-**2i** = 3.5 : 1.

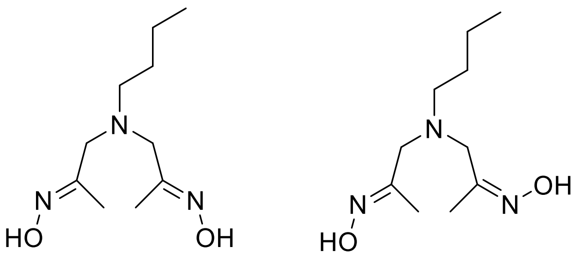

E,E-**2i** *E,Z*-**2i**

^1H NMR (300 MHz, $CDCl_3$, *E,E*-isomer) δ 9.62 (s, 2H, 2 NOH), 3.02 (s, 4H, 2 $CH_2C{=}N$), 2.36 (t, J = 7.1 Hz, 2H, $CH_2CH_2CH_2CH_3$), 1.90 (s, 6H, 2 $CH_3C{=}N$), 1.51 – 1.34 (m, 2H, $CH_2CH_2CH_2CH_3$), 1.34 – 1.19 (m, 2H, $CH_2CH_2CH_2CH_3$), 0.87 (t, J = 7.2 Hz, 3H, $CH_2CH_2CH_2CH_3$). $^{13}C\{^1H\}$ NMR (75 MHz, DEPT135, $CDCl_3$, *E,E*-isomer) δ 157.4 (2 *C*=N), 58.1 (2 $CH_2C{=}N$), 53.8 ($CH_2CH_2CH_2CH_3$), 51.5, 29.1 ($CH_2CH_2CH_2CH_3$), 20.5 ($CH_2CH_2CH_2CH_3$), 14.1 ($CH_2CH_2CH_2CH_3$), 12.6 (2 $CH_3C{=}N$). ^1H NMR (300 MHz, $CDCl_3$, *E,Z*-isomer) δ 9.62 (s, 2H, 2 NOH), 3.30 (s, 2H, $CH_2C{=}N$, *Z*-fragment), 3.05 (s, 2H, $CH_2C{=}N$, *E*-fragment), 2.36 (t, J = 7.1 Hz, 2H, $CH_2CH_2CH_2CH_3$), 1.92 (s, 3H, $CH_3C{=}N$, *Z*-fragment), 1.90 (s, 3H, $CH_3C{=}N$, *E*-fragment), 1.51 – 1.34 (m, 2H, $CH_2CH_2CH_2CH_3$), 1.34 – 1.19 (m, 2H, $CH_2CH_2CH_2CH_3$), 0.87 (t, J = 7.2 Hz, 3H, $CH_2CH_2CH_2CH_3$). $^{13}C\{^1H\}$ NMR (75 MHz, DEPT135, $CDCl_3$, *E,Z*-isomer) δ 157.1 and 156.9 (2 *C*=N), 58.8 ($CH_2C{=}N$,

E-fragment), 54.7 ($CH_2CH_2CH_2CH_3$), 51.5 (CH_2C=N, *Z*-fragment), 29.1 ($CH_2\mathit{C}H_2CH_2CH_3$), 20.6 ($CH_2CH_2\mathit{C}H_2CH_3$), 19.0 ($\mathit{C}H_3C$=N, *Z*-fragment), 14.1 ($CH_2CH_2CH_2\mathit{C}H_3$), 12.6 ($\mathit{C}H_3C$=N, *E*-fragment). HRMS (ESI): m/z calcd. for $[C_{10}H_{22}N_3O_2]^+$ 216.1707, found 216.1701 $[M + H]^+$.

1,1'-(cyclopentylazanediyl)bis(propan-2-one) dioxime (**2j**). The compound was prepared according to a general procedure from ene-nitrosoacetal **3b** (2.1 ml (1 M in CH_2Cl_2), 2.1 mmol) and cyclopentylamine (0.1 ml, 1 mmol). Yield: 190 mg (84 %). R_f = 0.42 (PE-EtOAc, 1:1). Colorless oil. Dynamic mixture of isomers, *E,E*-**2j** : *E,Z*-**2j** = 4.0 : 1.

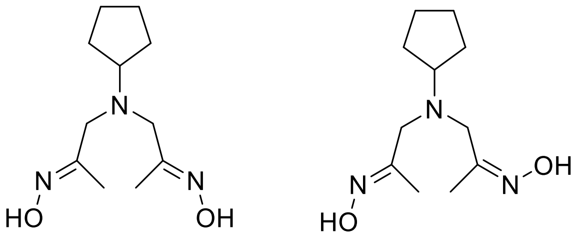

E,E-**2j** *E,Z*-**2j**

^{1}H NMR (300 MHz, $CDCl_3$, *E,E*-isomer) δ 9.46 (s, 2H, 2 NOH), 3.08 (s, 4H, 2 $C\mathit{H}_2C$=N), 3.13 – 3.00 (m, 1H, $CH_2CH_2C\mathit{H}$), 1.88 (s, 6H, 2 $C\mathit{H}_3C$=N), 1.83 – 1.67 (m, 2H, $CH_2C\mathit{H}_2CH$), 1.67 – 1.29 (m, 6H, $CH_2C\mathit{H}_2CH$ and $C\mathit{H}_2CH_2CH$). $^{13}C\{^1H\}$ NMR (75 MHz, DEPT135, $CDCl_3$, *E,E*-isomer) δ 157.5 (2 *C*=N), 63.6 ($CH_2CH_2\mathit{C}H$), 55.7 (2 $\mathit{C}H_2C$=N), 28.2 ($CH_2\mathit{C}H_2CH$), 24.1 ($\mathit{C}H_2CH_2CH$), 12.3 (2 $\mathit{C}H_3C$=N). ^{1}H NMR (300 MHz, $CDCl_3$, *E,Z*-isomer) δ 9.46 (s, 2H, 2 NOH), 3.37 (s, 2H, $C\mathit{H}_2C$=N, *Z*-fragment), 3.10 (s, 2H, $C\mathit{H}_2C$=N, *E*-fragment), 3.13 – 3.00 (m, 1H, $CH_2CH_2C\mathit{H}$), 1.92 (s, 3H, $C\mathit{H}_3C$=N, E-fragment), 1.88 (s, 3H, $C\mathit{H}_3C$=N, Z-fragment), 1.83 – 1.67 (m, 2H, $CH_2C\mathit{H}_2CH$), 1.67 – 1.29 (m, 6H, $CH_2C\mathit{H}_2CH$ and $C\mathit{H}_2CH_2CH$). $^{13}C\{^1H\}$ NMR (75 MHz, DEPT135, $CDCl_3$, *E,Z*-isomer) δ 157.6 and 157.2 (2 *C*=N), 64.5 ($CH_2CH_2\mathit{C}H$), 56.7 ($\mathit{C}H_2C$=N, *E*-fragment), 49.5 ($\mathit{C}H_2C$=N, *Z*-fragment), 28.2 ($CH_2\mathit{C}H_2CH$), 24.1 ($\mathit{C}H_2CH_2CH$), 19.3 ($\mathit{C}H_3C$=N, *Z*-fragment), 12.6 ($\mathit{C}H_3C$=N, *E*-fragment). ^{1}H NMR (300 MHz, NOESY, DMSO-d6, *E,E*-isomer) δ 10.44 (s, 2H, 2 NOH), 2.98 (s, 4H, 2 $C\mathit{H}_2C$=N), 3.10 – 2.91 (m, 1H,

CH_2CH_2CH), 1.73 (s, 6H, 2 CH_3C=N), 1.70 – 1.59 (m, 2H, CH_2CH_2CH), 1.59 – 1.28 (m, 6H, CH_2CH_2CH and CH_2CH_2CH). ^{1}H NMR (300 MHz, DMSO-d6, *E,Z*-isomer) δ 10.47 and 10.35 (s, 2H, 2 NOH), 3.22 (s, 2H, CH_2C=N, *Z*-fragment), 2.97 (s, 2H, CH_2C=N, *E*-fragment), 3.10 – 2.91 (m, 1H, CH_2CH_2CH), 1.78 (s, 3H, CH_3C=N, E-fragment), 1.76 (s, 3H, CH_3C=N, Z-fragment), 1.70 – 1.59 (m, 2H, CH_2CH_2CH), 1.59 – 1.28 (m, 6H, CH_2CH_2CH and CH_2CH_2CH). HRMS (ESI): m/z calcd. for $[C_{11}H_{22}N_3O_2]^+$ 228.1707, found 228.1715 $[M + H]^+$.

1,1'-(((S)-1-phenylethyl)azanediyl)bis(propan-2-one) dioxime (**2k**). The compound was prepared according to a general procedure from ene-nitrosoacetal **3b** (2.1 ml (1 M in CH_2Cl_2), 2.1 mmol) and α-phenylethylamine (0.127 ml, 1 mmol). Yield: 111 mg (42 %). R_f = 0.48 (PE-EtOAc, 1:1). Colorless oil. $[\alpha]_D$ = –26.7 (c=0.2, MeOH, 25 ^{o}C). Dynamic mixture of isomers, *E,E*-**2k** : *E,Z*-**2k** = 3.4 : 1.

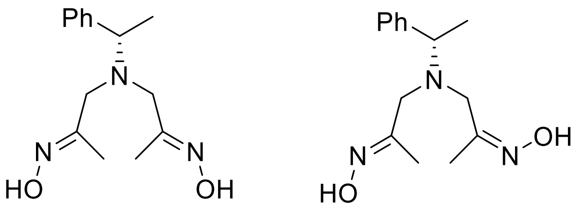

E,E-**2k** *E,Z*-**2k**

^{1}H NMR (300 MHz, $CDCl_3$, *E,E*-isomer) δ 9.39 (s, 2H, 2 NOH), 7.49 – 7.18 (m, 5H, Ph), 3.92 (q, J = 6.9 Hz, 1H, $PhCHCH_3$), 3.13 (d, J = 13.6 Hz, 2H, CH_2C=N), 2.99 (d, J = 13.5 Hz, 2H, CH_2C=N), 1.87 (s, 6H, 2 CH_3C=N), 1.38 (d, J = 6.9 Hz, 3H, $PhCHCH_3$). ^{13}C{^{1}H} NMR (75 MHz, DEPT135, $CDCl_3$, *E,E*-isomer) δ 157.4 (2 *C*=N), 142.1 (i-Ph), 128.2 (o-Ph and m-Ph), 127.2 (p-Ph), 58.2 ($PhCHCH_3$), 53.9 (2 CH_2C=N), 13.2 ($PhCHCH_3$), 12.4 (2 CH_3C=N). ^{1}H NMR (300 MHz, $CDCl_3$, *E,Z*-isomer) δ 9.39 (s, 2H, 2 NOH), 7.49 – 7.18 (m, 5H, Ph), 3.86 (q, J = 6.9 Hz, 1H, $PhCHCH_3$), 3.52 (d, J = 14.8 Hz, 2H, CH_2C=N, *Z*-fragment), 2.99 (d, J = 13.5 Hz, 2H, CH_2C=N, *E*-fragment), 1.91 (s, 3H, CH_3C=N), 1.90 (s, 3H, CH_3C=N), 1.41 (d, J = 6.9 Hz, 3H, $PhCHCH_3$). ^{13}C{^{1}H} NMR (75 MHz, DEPT135, $CDCl_3$, *E,Z*-isomer) δ 157.4 (2 *C*=N), 141.9 (i-Ph), 128.3 and 128.2 (o-

Ph and m-Ph), 127.3 (p-Ph), 59.6 (Ph*C*HCH$_3$), 54.9 (*C*H$_2$C=N, *E*-fragment), 47.1 (*C*H$_2$C=N, *Z*-fragment), 19.0 (*C*H$_3$C=N, *Z*-fragment), 14.1 (PhCH*C*H$_3$), 12.5 (*C*H$_3$C=N, *E*-fragment). HRMS (ESI): m/z calcd. for $[C_{14}H_{22}N_3O_2]^+$ 264.1707, found 264.1703 $[M + H]^+$.

ethyl bis(2-(hydroxyimino)propyl)glycinate (**2l**). The compound was prepared according to a general procedure from ene-nitrosoacetal **3b** (2.1 ml (1 M in CH_2Cl_2), 2.1 mmol) and ethyl glycinate (103 mg, 1 mmol). Yield: 72 mg (29 %). R_f = 0.67 (PE-EtOAc, 1:1). Colorless oil. Dynamic mixture of isomers, *E,E*-**2l** : *E,Z*-**2l** = 4.5 : 1.

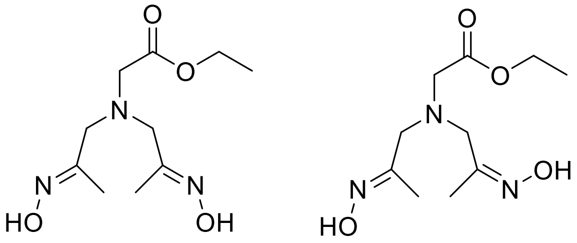

E,E-**2l** *E,Z*-**2l**

^{1}H NMR (300 MHz, $CDCl_3$, *E,E*-isomer) δ 9.52 (s, 2H, 2 NOH), 4.12 (q, J = 7.1 Hz, 2H, C*H*$_2$CH$_3$), 3.26 (s, 2H, C*H*$_2$C=O), 3.22 (s, 4H, 2 C*H*$_2$C=N), 1.91 (s, 6H, 2 C*H*$_3$C=N), 1.23 (t, J = 7.1 Hz, 3H, CH$_2$C*H*$_3$). $^{13}C\{^1H\}$ NMR (75 MHz, DEPT135, $CDCl_3$, *E,E*-isomer) δ 171.0 (*C*=O), 156.6 (2 *C*=N), 60.7 (*C*H$_2$CH$_3$), 57.9 (*C*H$_2$C=O), 54.5 (2 *C*H$_2$C=N), 14.3 (CH$_2$*C*H$_3$), 12.5 (2 *C*H$_3$C=N). ^{1}H NMR (300 MHz, $CDCl_3$, *E,Z*-isomer) δ 9.52 (s, 2H, 2 NOH), 4.12 (q, J = 7.1 Hz, 2H, C*H*$_2$CH$_3$), 3.47 (s, 2H, C*H*$_2$C=N, *Z*-fragment), 3.26 (s, 2H, C*H*$_2$C=O), 3.24 (s, 2H, C*H*$_2$C=N, *E*-fragment), 1.93 and 1.92 (s, 6H, C*H*$_3$C=N, *Z*-fragment and C*H*$_3$C=N, *E*-fragment), 1.23 (t, J = 7.1 Hz, 3H, CH$_2$C*H*$_3$). $^{13}C\{^1H\}$ NMR (75 MHz, DEPT135, $CDCl_3$, *E,Z*-isomer) δ 170.9 (*C*=O), 156.7 and 156.5 (2 *C*=N), 64.1 (*C*H$_2$CH$_3$), 58.8 (*C*H$_2$C=O), 55.3 (*C*H$_2$C=N, *E*-fragment), 50.3 (*C*H$_2$C=N, *Z*-fragment), 18.7 (*C*H$_3$C=N, *Z*-fragment), 14.3 (CH$_2$*C*H$_3$), 12.5 (*C*H$_3$C=N, *E*-fragment). HRMS (ESI): m/z calcd. for $[C_{10}H_{20}N_3O_4]^+$ 246.1448, found 246.1440 $[M + H]^+$.

1,1'-(benzylazanediyl)bis(propan-2-one) dioxime (**2m**). The compound was prepared according to a general procedure from ene-nitrosoacetal **3b** (2.1 ml (1 M in CH_2Cl_2), 2.1 mmol) and benzylamine (110 mg, 1 mmol). Yield: 200 mg (80 %). R_f = 0.61 (PE-EtOAc, 1:1). Colorless oil. Dynamic mixture of isomers, *E,E*-**2m** : *E,Z*-**2m** = 4.7 : 1. ^{1}H NMR spectrum is in agreement with previously published data [13].

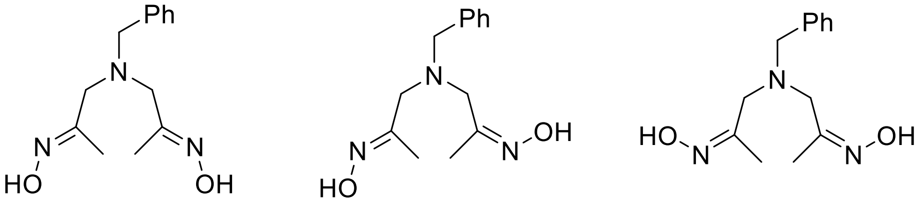

1,1'-(benzylazanediyl)bis(propan-2-one) dioxime. Pd/C (5%, 100 mg) was added to a solution of **2m** (620 mg, 2.49 mmol) in MeOH (10 mL) and the mixture was hydrogenated with H_2 (5 atm) in a steel autoclave at 45 °C for 2 h. The resulting solution was filtered through Celite and the filtrate was evaporated. The residue was washed with Et_2O and dried in vacuo to give 1,1'-(benzylazanediyl)bis(propan-2-one) dioxime as a white solid. Yield: 333 mg (84 %). Single *E,E*-isomer. ^{1}H NMR spectrum is in agreement with previously published data [13].

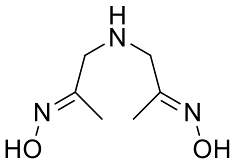

tert-butyl bis(2-(hydroxyimino)propyl)carbamate (**2n**). Et_3N (0.52 ml, 3.78 mmol) and Boc_2O (824 mg, 3.78 mmol) were added to a solution of 1,1'-(benzylazanediyl)bis(propan-2-one) dioxime (300 mg, 1.89 mmol) in methanol (3 ml). The mixture was stirred at room temperature for 19 hours. The resulting mixture was concentrated in vacuo and chromatographed to give **2n** (485 mg, 99

%) as colorless oil. R_f = 0.7 (PE-EtOAc, 1:1). Dynamic mixture of isomers, *E,E*-**2n** : *E,Z*-**2n** : *Z,Z*-**2n** = 22.2 : 9.6 : 1.

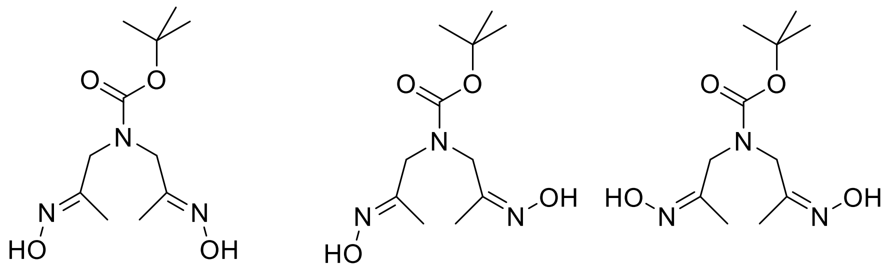

E,E-**2n** *E,Z*-**2n** *Z,Z*-**2n**

^{1}H NMR (300 MHz, DMSO-d6, *E,E*-isomer) δ 10.64 (s, 2H, 2 NOH), 3.79 (s, 4H, 2 CH_2C=N), 1.68 (s, 6H, 2 CH_3C=N), 1.39 (s, 9H, 3 CH_3). ^{13}C{^{1}H} NMR (75 MHz, DEPT135, DMSO-d6, *E,E*-isomer) δ 155.3 (*C*=O), 152.1 (2 *C*=N), 79.7 (*C*(CH_3)$_3$), 49.5 (2 *C*H_2C=N), 28.4 (3 *C*H_3), 11.9 (2 *C*H_3C=N). ^{1}H NMR (300 MHz, $CDCl_3$, *E,Z*-isomer) δ 10.71 (s, 1H, NOH), 10.49 (s, 1H, NOH), 3.96 (s, 2H, CH_2C=N, *Z*-fragment), 3.83 (s, 2H, CH_2C=N, *E*-fragment), 1.70 (s, 6H, 2 CH_3C=N), 1.39 (s, 9H, 3 CH_3). ^{13}C{^{1}H} NMR (75 MHz, DEPT135, DMSO-d6, *E,Z*-isomer) δ 155.3 (*C*=O), 152.1 (2 *C*=N), 79.8 (*C*(CH_3)$_3$), 49.9 (*C*H_2C=N, *E*-fragment), 44.4 (*C*H_2C=N, *Z*-fragment), 28.4 (3 *C*H_3), 17.0 (*C*H_3C=N, *Z*-fragment), 11.9 (*C*H_3C=N, *E*-fragment). ^{1}H NMR (300 MHz, DMSO-d6, *Z,Z*-isomer) δ 10.54 (s, 2H, 2 NOH), 4.01 (s, 4H, 2 CH_2C=N), 1.73 (s, 6H, 2 CH_3C=N), 1.39 (s, 9H, 3 CH_3). ^{13}C{^{1}H} NMR (75 MHz, DEPT135, DMSO-d6, *Z,Z*-isomer, characteristic signals) δ 155.3 (*C*=O), 152.1 (2 *C*=N), 80.0 (*C*(CH_3)$_3$), 28.4 (3 *C*H_3), 12.0 (2 *C*H_3C=N). HRMS (ESI): m/z calcd. for $[C_{11}H_{22}N_3O_4]^+$ 260.1605, found 260.1611 $[M + H]^+$.

2,2'-(butylazanediyl)bis(1-phenylethan-1-one) dioxime (**2o**). The compound was prepared according to a general procedure from ene-nitrosoacetal **3c** (2.1 ml (1 M in CH_2Cl_2), 2.1 mmol) and n-butylamine (0.1 ml, 1 mmol). Yield: 199 mg (59 %). R_f = 0.88 (PE-EtOAc, 1:1). Colorless oil. Dynamic mixture of isomers, *E*,*E*-**2o** : *E*,*Z*-**2o** : *Z*,*Z*-**2o** = 1 : 10 : 1.3.

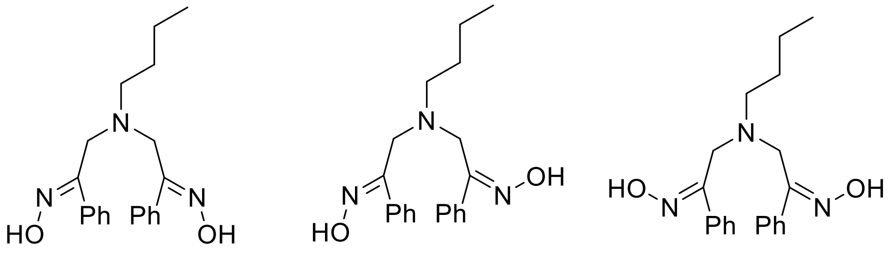

E,E-**2o** *E,Z*-**2o** *Z,Z*-**2o**

^{1}H NMR (300 MHz, COSY, HSQC, $CDCl_3$, *E,E*-isomer) δ 10.35 (s, 2H, 2 NOH), 7.71 – 7.11 (m, 10H, 2 Ph), 3.60 (s, 4H, 2 $C\mathit{H}_2C$=N), 2.57 (t, J = 7.7 Hz, 2H, $C\mathit{H}_2CH_2CH_2CH_3$), 1.54 – 1.39 (m, 2H, $CH_2C\mathit{H}_2CH_2CH_3$), 1.16 (h, J = 7.3 Hz, 2H, $CH_2CH_2C\mathit{H}_2CH_3$), 0.85 (t, J = 7.3 Hz, 3H, $CH_2CH_2CH_2C\mathit{H}_3$). $^{13}C\{^1H\}$ NMR (75 MHz, DEPT135, HSQC, $CDCl_3$, *E,E*-isomer) δ 155.3 (2 *C*=N), 132.2 (2 i-Ph), 128.9 (2 p-Ph), 128.5 (2 m-Ph), 125.9 (2 o-Ph), 57.5 ($\mathit{C}H_2CH_2CH_2CH_3$), 54.6 (2 $\mathit{C}H_2C$=N), 28.4 ($CH_2\mathit{C}H_2CH_2CH_3$), 20.3 ($CH_2CH_2\mathit{C}H_2CH_3$), 14.0 ($CH_2CH_2CH_2\mathit{C}H_3$). ^{1}H NMR (300 MHz, COSY, HSQC, $CDCl_3$, *E,Z*-isomer) δ 10.35 (s, 2H, 2 NOH), 7.71 – 7.11 (m, 10H, 2 Ph), 3.90 (s, 2H, $C\mathit{H}_2C$=N, *Z*-fragment), 3.65 (s, 2H, $C\mathit{H}_2C$=N, *E*-fragment), 2.57 (t, J = 7.7 Hz, 2H, $C\mathit{H}_2CH_2CH_2CH_3$), 1.54 – 1.39 (m, 2H, $CH_2C\mathit{H}_2CH_2CH_3$), 1.16 (h, J = 7.3 Hz, 2H, $CH_2CH_2C\mathit{H}_2CH_3$), 0.85 (t, J = 7.3 Hz, 3H, $CH_2CH_2CH_2C\mathit{H}_3$). $^{13}C\{^1H\}$ NMR (75 MHz, DEPT135, HSQC, $CDCl_3$, *E,Z*-isomer) δ 155.8 and 154.8 (2 *C*=N), 135.3 and 131.6 (2 i-Ph), 129.3 and 129.1 (2 p-Ph), 128.6 and 128.2 and 128.1 and 126.8 (2 o-Ph and 2 m-Ph), 57.9 ($\mathit{C}H_2CH_2CH_2CH_3$), 54.1 ($\mathit{C}H_2C$=N, *E*-fragment), 49.9 ($\mathit{C}H_2C$=N, *Z*-fragment), 28.4 ($CH_2\mathit{C}H_2CH_2CH_3$), 20.3 ($CH_2CH_2\mathit{C}H_2CH_3$), 14.0 ($CH_2CH_2CH_2\mathit{C}H_3$). ^{1}H NMR (300 MHz, COSY, HSQC, $CDCl_3$, *Z,Z*-isomer) δ 10.35 (s, 2H, 2 NOH), 7.71 – 7.11 (m, 10H, 2 Ph), 3.95 (s, 4H, 2 $C\mathit{H}_2C$=N), 2.57 (t, J = 7.7 Hz, 2H, $C\mathit{H}_2CH_2CH_2CH_3$), 1.54 – 1.39 (m, 2H, $CH_2C\mathit{H}_2CH_2CH_3$), 1.16 (h, J = 7.3 Hz, 2H, $CH_2CH_2C\mathit{H}_2CH_3$), 0.85 (t, J = 7.3 Hz, 3H, $CH_2CH_2CH_2C\mathit{H}_3$).

$^{13}C\{^1H\}$ NMR (75 MHz, DEPT135, HSQC, $CDCl_3$, *Z,Z*-isomer, characteristic signals) δ 155.7 (2 *C*=N), 135.0 (2 i-Ph), 129.6 (2 p-Ph), 128.4 (2 m-Ph), 127.9 (2 o-Ph), 57.9 ($\mathit{C}H_2CH_2CH_2CH_3$), 49.5 (2 $\mathit{C}H_2C$=N), 28.4 ($CH_2\mathit{C}H_2CH_2CH_3$), 19.8 ($CH_2CH_2\mathit{C}H_2CH_3$), 14.0 ($CH_2CH_2CH_2\mathit{C}H_3$). HRMS (ESI): m/z calcd. for $[C_{20}H_{26}N_3O_2]^+$ 340.2020, found 340.2020 $[M + H]^+$.

2-(butyl(2-(hydroxyimino)propyl)amino)acetaldehyde oxime (**2p**). The compound was prepared according to a general procedure from ene-nitrosoacetal **3b** (0.92 ml (1 M in CH_2Cl_2), 0.92 mmol) and monooxime **4a** (100 mg, 0.77 mmol). Yield: 48 mg (31 %). R_f = 0.47 (PE-EtOAc, 1:1). Colorless oil. Dynamic mixture of isomers, *E*,*E*-**2p** : *E*,*Z*-**2p** : *Z*,*E*-**2p** : *Z*,*Z*-**2p** = 6.4 : 2.6 : 4.1 : 1.

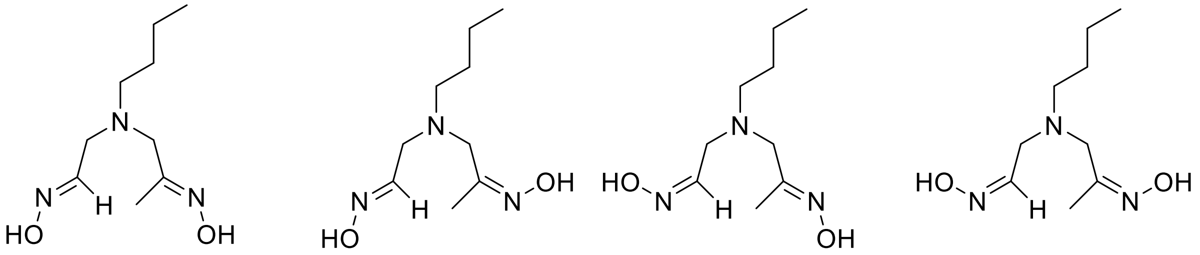

E,E-**2p** *E,Z*-**2p** *Z,E*-**2p** *Z,Z*-**2p**

^1H NMR (300 MHz, $CDCl_3$, *E,E*-isomer) δ 9.34 (s, 2H, 2 NOH), 7.41 (t, J = 6.1 Hz, 1H, C*H*N), 3.19 (d, J = 6.1 Hz, 2H, $C\mathit{H}_2C$=N, oxyiminoethyl-fragment), 3.11 (s, 2H, $C\mathit{H}_2C$=N, oxyiminopropyl-fragment), 2.52 – 2.44 (m, 2H, $C\mathit{H}_2CH_2CH_2CH_3$), 1.90 (s, 3H, $C\mathit{H}_3C$=N), 1.45 (m, 2H, $CH_2C\mathit{H}_2CH_2CH_3$), 1.38 – 1.21 (m, 2H, $CH_2CH_2C\mathit{H}_2CH_3$), 0.89 (t, J = 7.2 Hz, 3H, $CH_2CH_2CH_2C\mathit{H}_3$). $^{13}C\{^1H\}$ NMR (75 MHz, DEPT135, $CDCl_3$, *E,E*-isomer) δ 157.0 (Me-*C*=N), 149.3 (H-*C*=N), 58.1 ($CH_2CH_2CH_2CH_3$), 54.3 and 52.3 (2 $\mathit{C}H_2C$=N), 29.2 ($CH_2\mathit{C}H_2CH_2CH_3$), 20.5 ($CH_2CH_2\mathit{C}H_2CH_3$), 14.1 ($CH_2CH_2CH_2\mathit{C}H_3$), 12.7 ($\mathit{C}H_3C$=N). ^1H NMR (300 MHz, $CDCl_3$, *E,Z*-isomer) δ 9.34 (s, 2H, 2 NOH), 7.44 (t, J = 6.1 Hz, 1H, C*H*N), 3.19 (d, J = 6.1 Hz, 2H, $C\mathit{H}_2C$=N, oxyiminoethyl-fragment), 3.11 (s, 2H, $C\mathit{H}_2C$=N, oxyiminopropyl-fragment), 2.52 – 2.44 (m, 2H, $C\mathit{H}_2CH_2CH_2CH_3$), 1.92 (s, 3H, $C\mathit{H}_3C$=N), 1.45 (m, 2H, $CH_2C\mathit{H}_2CH_2CH_3$), 1.38 – 1.21 (m, 2H, $CH_2CH_2C\mathit{H}_2CH_3$), 0.89 (t, J = 7.2 Hz, 3H, $CH_2CH_2CH_2C\mathit{H}_3$).

$^{13}C\{^1H\}$ NMR (75 MHz, DEPT135, $CDCl_3$, *E,Z*-isomer) δ 156.6 (Me-*C*=N), 150.7 (H-*C*=N), 58.9 ($CH_2CH_2CH_2CH_3$), 55.1 and 49.1 (2 $\mathit{C}H_2C$=N), 29.2 ($CH_2\mathit{C}H_2CH_2CH_3$), 20.5 ($CH_2CH_2\mathit{C}H_2CH_3$), 14.1 ($CH_2CH_2CH_2\mathit{C}H_3$), 12.7 ($\mathit{C}H_3C$=N). ^1H NMR (300 MHz, $CDCl_3$, *Z,E*-isomer) δ 9.34 (s, 2H, 2 NOH), 6.82 (t, J = 4.4 Hz, 1H, C*H*N), 3.39 (d, J = 4.4 Hz, 2H, $C\mathit{H}_2C$=N, oxyiminoethyl-fragment), 3.11 (s, 2H, $C\mathit{H}_2C$=N, oxyiminopropyl-fragment), 2.52 – 2.44 (m, 2H, $C\mathit{H}_2CH_2CH_2CH_3$), 2.16 (s, 3H, $C\mathit{H}_3C$=N), 11.45 (m, 2H, $CH_2C\mathit{H}_2CH_2CH_3$), 1.38 – 1.21 (m, 2H, $CH_2CH_2C\mathit{H}_2CH_3$), 0.89 (t, J = 7.2 Hz, 3H, $CH_2CH_2CH_2C\mathit{H}_3$). $^{13}C\{^1H\}$ NMR (75 MHz, DEPT135, $CDCl_3$, *Z,E*-isomer, characteristic signals) δ 157.0 (Me-*C*=N), 148.7 (H-*C*=N), 28.7 ($CH_2\mathit{C}H_2CH_2CH_3$). ^1H NMR (300 MHz, $CDCl_3$, *Z,Z*-isomer) δ 9.34 (s, 2H, 2 NOH), 6.87 (t, J = 4.4 Hz, 1H, C*H*N), 3.38 (d, J = 4.4 Hz, 2H, $C\mathit{H}_2C$=N, oxyiminoethyl-fragment), 3.11 (s, 2H, $C\mathit{H}_2C$=N, oxyiminopropyl-fragment), 2.52 – 2.44 (m, 2H, $C\mathit{H}_2CH_2CH_2CH_3$), 1.90 (s, 3H, $C\mathit{H}_3C$=N), 1.45 (m, 2H, $CH_2C\mathit{H}_2CH_2CH_3$), 1.38 – 1.21 (m, 2H, $CH_2CH_2C\mathit{H}_2CH_3$), 0.89 (t, J = 7.2 Hz, 3H, $CH_2CH_2CH_2C\mathit{H}_3$). $^{13}C\{^1H\}$ NMR (75 MHz, DEPT135, $CDCl_3$, *Z,Z*-isomer, characteristic signals) δ 150.6 (H-*C*=N). HRMS (ESI): m/z calcd. for $[C_9H_{20}N_3O_2]^+$ 202.1550, found 202.1549 $[M + H]^+$.

2-(butyl(2-(hydroxyimino)butyl)amino)acetaldehyde oxime (**2q**). The compound was prepared according to a general procedure from ene-nitrosoacetal **3d** (0.92 ml (1 M in CH_2Cl_2), 0.92 mmol) and monooxime **4a** (100 mg, 0.77 mmol). Yield: 50 mg (30 %). R_f = 0.52 (PE-EtOAc, 1:1). Colorless oil. Dynamic mixture of isomers, *E,E*-**2q** : *E,Z*-**2q** : *Z,E*-**2q** : *Z,Z*-**2q** = 1.6 : 1.5 : 1.3 : 1.

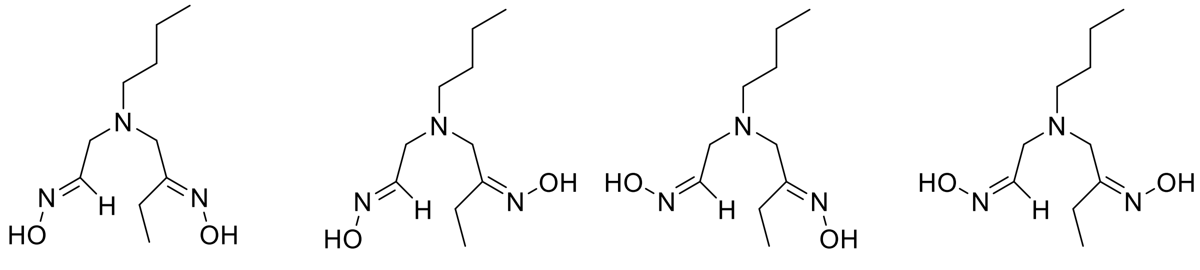

E,E-**2q** *E,Z*-**2q** *Z,E*-**2q** *Z,Z*-**2q**

^{1}H NMR (300 MHz, $CDCl_3$, *E,E*-isomer) δ 9.29 (s, 2H, 2 NOH), 7.43 (t, J = 6.1 Hz, 1H, C*H*N), 3.19 (d, J = 6.2 Hz, 2H, CH_2C=N, oxyiminoethyl-fragment), 3.11 (s, 2H, CH_2C=N, oxyiminobutyl-fragment), 2.55 – 2.25 (m, 4H, C$H_2$$CH_2CH_2CH_3$ and N=CC$H_2$$CH_3$), 1.54 – 1.41 (m, 2H, CH_2C$H_2$$CH_2CH_3$), 1.40 – 1.25 (m, 2H, CH_2CH_2C$H_2$$CH_3$), 1.10 (t, J = 7.6 Hz, 3H, N=CCH_2CH_3), 0.99 – 0.86 (m, 3H, $CH_2CH_2CH_2$CH_3). ^{13}C{^{1}H} NMR (75 MHz, DEPT135, HSQC, HMBC, $CDCl_3$, *E,E*-isomer) δ 161.0 (Et-*C*=N), 149.2 (*H*-C=N), 56.2 (*C*H_2C=N, oxyiminobutyl-fragment), 54.2 (*C*$H_2CH_2CH_2CH_3$), 52.1 (*C*H_2C=N, oxyiminoethyl-fragment), 29.2 (CH_2*C*$H_2CH_2CH_3$), 20.4 (CH_2CH_2*C*H_2CH_3), 19.6 (NC*C*H_2CH_3), 14.0 ($CH_2CH_2CH_2$*C*H_3), 10.0 (NCCH_2*C*H_3). ^{1}H NMR (300 MHz, $CDCl_3$, *E,Z*-isomer) δ 9.29 (s, 2H, 2 NOH), 7.46 (t, J = 6.1 Hz, 1H, C*H*N), 3.40 (s, 2H, CH_2C=N, oxyiminobutyl-fragment), 3.21 (d, J = 6.2 Hz, 2H, CH_2C=N, oxyiminoethyl-fragment), 2.55 – 2.25 (m, 4H, C$H_2$$CH_2CH_2CH_3$ and N=CC$H_2$$CH_3$), 1.54 – 1.41 (m, 2H, CH_2C$H_2$$CH_2CH_3$), 1.40 – 1.25 (m, 2H, CH_2CH_2C$H_2$$CH_3$), 1.10 (t, J = 7.6 Hz, 3H, N=CCH_2CH_3), 0.99 – 0.86 (m, 3H, $CH_2CH_2CH_2$CH_3). ^{13}C{^{1}H} NMR (75 MHz, DEPT135, HSQC, HMBC, $CDCl_3$, *E,Z*-isomer) δ 160.7 (Et-*C*=N), 148.3 (*H*-C=N), 57.1 (*C*H_2C=N, oxyiminobutyl-fragment), 55.1 (*C*$H_2CH_2CH_2CH_3$), 52.8 (*C*H_2C=N, oxyiminoethyl-fragment), 29.2 (CH_2*C*$H_2CH_2CH_3$), 20.5 (CH_2CH_2*C*H_2CH_3), 19.7 (NC*C*H_2CH_3), 14.0 ($CH_2CH_2CH_2$*C*H_3), 10.0 (NCCH_2*C*H_3). ^{1}H NMR (300 MHz, $CDCl_3$, *Z,E*-isomer) δ 9.29 (s, 2H, 2 NOH), 6.83 (t, J = 4.4 Hz, 1H, C*H*N), 3.41 (d, J = 4.4 Hz, 2H, CH_2C=N, oxyiminoethyl-fragment), 3.11 (s, 2H, CH_2C=N, oxyiminobutyl-fragment), 2.55 – 2.25 (m, 4H, C$H_2$$CH_2CH_2CH_3$ and N=CC$H_2$$CH_3$), 1.54 – 1.41 (m, 2H, CH_2C$H_2$$CH_2CH_3$), 1.40 – 1.25 (m, 2H, CH_2CH_2C$H_2$$CH_3$), 1.10 (t, J = 7.6 Hz, 3H, N=CCH_2CH_3), 0.99 – 0.86 (m, 3H, $CH_2CH_2CH_2$CH_3). ^{13}C{^{1}H} NMR (75 MHz, DEPT135, HSQC, HMBC, $CDCl_3$, *Z,E*-isomer, characteristic signals) δ 159.4 (Et-*C*=N), 150.7 (*H*-C=N), 56.9 (*C*H_2C=N, oxyiminobutyl-fragment), 54.8 (*C*$H_2CH_2CH_2CH_3$), 49.0 (*C*H_2C=N, oxyiminoethyl-fragment), 29.0 (CH_2*C*$H_2CH_2CH_3$), 26.6 (NC*C*H_2CH_3), 20.4 (CH_2CH_2*C*H_2CH_3), 14.0 ($CH_2CH_2CH_2$*C*H_3), 10.9 (NCCH_2*C*H_3). ^{1}H NMR (300 MHz, $CDCl_3$, *Z,Z*-isomer) δ 9.29 (s, 2H, 2 NOH), 6.88 (t, J = 4.4 Hz, 1H, C*H*N),

3.43 (d, J = 4.4 Hz, 2H, C*H*$_2$C=N, oxyiminoethyl-fragment), 3.40 (s, 2H, C*H*$_2$C=N, oxyiminobutyl-fragment), 2.55 – 2.25 (m, 4H, C*H*$_2$CH$_2$CH$_2$CH$_3$ and N=CC*H*$_2$CH$_3$), 1.54 – 1.41 (m, 2H, CH$_2$C*H*$_2$CH$_2$CH$_3$), 1.40 – 1.25 (m, 2H, CH$_2$CH$_2$C*H*$_2$CH$_3$), 1.10 (t, J = 7.6 Hz, 3H, N=CCH$_2$C*H*$_3$), 0.99 – 0.86 (m, 3H, CH$_2$CH$_2$CH$_2$C*H*$_3$). ^{13}C{^{1}H} NMR (75 MHz, DEPT135, HSQC, HMBC, CDCl$_3$, *Z,Z*-isomer, characteristic signals) δ 152.8 (Et-*C*=N), 150.3 (*H*-C=N), 57.1 (*C*H$_2$C=N, oxyiminobutyl-fragment), 54.2 (*C*H$_2$C=N, oxyiminoethyl-fragment), 28.7 (CH$_2$*C*H$_2$CH$_2$CH$_3$), 23.3 (CH$_2$CH$_2$*C*H$_2$CH$_3$), 13.9 (CH$_2$CH$_2$CH$_2$*C*H$_3$), 8.8. HRMS (ESI): m/z calcd. for $[C_{10}H_{22}N_3O_2]^+$ 216.1707, found 216.1709 $[M + H]^+$.

2-(butyl(2-(hydroxyimino)-3-phenylpropyl)amino)acetaldehyde oxime (**2r**). The compound was prepared according to a general procedure from ene-nitrosoacetal **3e** (0.92 ml (1 M in CH$_2$Cl$_2$), 0.92 mmol) and monooxime **4a** (100 mg, 0.77 mmol). Yield: 67 mg (32 %). R_f = 0.73 (PE-EtOAc, 1:1). Colorless oil. Dynamic mixture of isomers, *E,E*-**2r** : *E,Z*-**2r** : *Z,E*-**2r** : *Z,Z*-**2r** = 1.6 : 1.5 : 1.3 : 1.

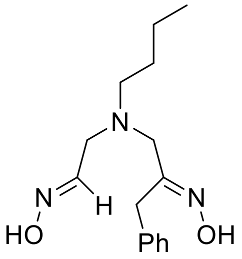

E,E-**2r**

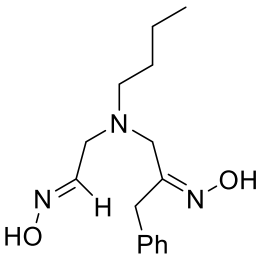

E,Z-**2r**

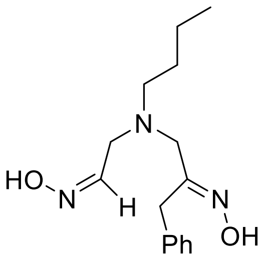

Z,E-**2r**

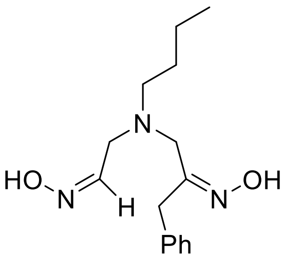

Z,Z-**2r**

^{1}H NMR (300 MHz, CDCl$_3$, *E,E*-isomer) δ 9.25 (s, 2H, 2 NOH), 7.37 (t, J = 6.4 Hz, 1H, C*H*N), 7.33 – 7.17 (m, 5H, Ph), 3.84 (s, 2H, C*H*$_2$Ph), 3.21 (d, J = 6.4 Hz, 2H, C*H*$_2$CHNOH), 3.09 (s, 2H, C*H*$_2$CNBn), 2.46 (m, 2H, C*H*$_2$CH$_2$CH$_2$CH3), 1.48 – 1.23 (m, 4H, CH$_2$C*H*$_2$CH$_2$CH$_3$ and CH$_2$CH$_2$C*H*$_2$CH$_3$), 0.96 – 0.86 (m, 3H, CH$_2$CH$_2$CH$_2$C*H*$_3$). ^{13}C{^{1}H} NMR (75 MHz, DEPT135, HSQC, HMBC, CDCl$_3$, *E,E*-isomer) δ 157.9 (Bn-*C*=N), 149.2 (H-*C*=N), 136.8 (i-Ph), 129.4 and 128.6 (o-Ph and m-Ph), 126.5 (p-Ph), 55.9 (*C*H$_2$Ph), 54.8 (*C*H$_2$CH$_2$CH$_2$CH$_3$), 54.3 (*C*H$_2$CNBn), 52.1 (*C*H$_2$CHNOH), 29.2 (CH$_2$*C*H$_2$CH$_2$CH$_3$), 20.5 (CH$_2$CH$_2$*C*H$_2$CH$_3$), 14.1 (CH$_2$CH$_2$CH$_2$*C*H$_3$). ^{1}H NMR (300 MHz, CDCl$_3$, *E,Z*-

isomer) δ 9.25 (s, 2H, 2 NOH), 7.35 (t, J = 6.4 Hz, 1H, C*H*N), 7.33 – 7.17 (m, 5H, Ph), 3.86 (s, 2H, C*H*$_2$Ph), 3.33 (s, 2H, C*H*$_2$CNBn), 3.16 (d, J = 6.4 Hz, 2H, C*H*$_2$CHNOH), 2.46 (m, 2H, C*H*$_2$CH$_2$CH$_2$CH3), 1.48 – 1.23 (m, 4H, CH$_2$C*H*$_2$CH$_2$CH$_3$ and CH$_2$CH$_2$C*H*$_2$CH$_3$), 0.96 – 0.86 (m, 3H, CH$_2$CH$_2$CH$_2$C*H*$_3$). ^{13}C{^{1}H} NMR (75 MHz, DEPT135, HSQC, HMBC, CDCl$_3$, *E,Z*-isomer) δ 158.1 (Me-*C*=N), 150.7 (H-*C*=N), 136.9 (i-Ph), 129.5 and 128.7 (o-Ph and m-Ph), 126.6 (p-Ph), 56.8 (*C*H$_2$Ph), 55.2 (*C*H$_2$CH$_2$CH$_2$CH$_3$), 52.7 and 49.0 (2 *C*H$_2$C=N), 31.9 (CH$_2$*C*H$_2$CH$_2$CH$_3$), 20.6 (CH$_2$CH$_2$*C*H$_2$CH$_3$), 14.0 (CH$_2$CH$_2$CH$_2$*C*H$_3$). ^{1}H NMR (300 MHz, CDCl$_3$, *Z,E*-isomer) δ 9.25 (s, 2H, 2 NOH), 7.33 – 7.17 (m, 5H, Ph), 6.75 (t, J = 4.5 Hz, 1H, C*H*N), 3.80 (s, 2H, C*H*$_2$Ph), 3.41 (d, J = 4.5 Hz, 2H, C*H*$_2$CHNOH), 3.08 (s, 2H, C*H*$_2$CNBn), 2.46 (m, 2H, C*H*$_2$CH$_2$CH$_2$CH3), 1.48 – 1.23 (m, 4H, CH$_2$C*H*$_2$CH$_2$CH$_3$ and CH$_2$CH$_2$C*H*$_2$CH$_3$), 0.96 – 0.86 (m, 3H, CH$_2$CH$_2$CH$_2$C*H*$_3$). ^{13}C{^{1}H} NMR (75 MHz, DEPT135, HSQC, HMBC, CDCl$_3$, *Z,E*-isomer, characteristic signals) δ 157.7 (Me-*C*=N), 148.3 (H-*C*=N), 136.7 (i-Ph), 129.3 and 128.7 (o-Ph and m-Ph), 126.8 (p-Ph), 54.7 (*C*H$_2$CH$_2$CH$_2$CH$_3$), 52.7 (*C*H$_2$CNBn), 47.3 (*C*H$_2$CHNOH), 32.0 (CH$_2$*C*H$_2$CH$_2$CH$_3$), 20.4 (CH$_2$CH$_2$*C*H$_2$CH$_3$), 14.0 (CH$_2$CH$_2$CH$_2$*C*H$_3$). ^{1}H NMR (300 MHz, CDCl$_3$, *Z,Z*-isomer) δ 9.25 (s, 2H, 2 NOH), 7.33 – 7.17 (m, 5H, Ph), 6.67 (t, J = 4.5 Hz, 1H, C*H*N), 3.60 (s, 4H, C*H*$_2$Ph and C*H*$_2$CNBn), 3.39 (d, J = 4.5 Hz, 2H, C*H*$_2$CHNOH), 2.46 (m, 2H, C*H*$_2$CH$_2$CH$_2$CH3), 1.48 – 1.23 (m, 4H, CH$_2$C*H*$_2$CH$_2$CH$_3$ and CH$_2$CH$_2$C*H*$_2$CH$_3$), 0.96 – 0.86 (m, 3H, CH$_2$CH$_2$CH$_2$C*H*$_3$). ^{13}C{^{1}H} NMR (75 MHz, DEPT135, HSQC, HMBC, CDCl$_3$, *Z,Z*-isomer, characteristic signals) δ 126.7 (p-Ph), 28.8 (CH$_2$*C*H$_2$CH$_2$CH$_3$), 14.0 (CH$_2$CH$_2$CH$_2$*C*H$_3$). HRMS (ESI): m/z calcd. for [$C_{15}H_{24}N_3O_2$]$^+$ 278.1863, found 278.1863 [M + H]$^+$.

Methyl 5-(benzyl(2-(hydroxyimino)ethyl)amino)-4-(hydroxyimino)pentanoate (2s). The compound was prepared according to a general procedure from ene-nitrosoacetal **3f** (0.88 ml (1 M in CH_2Cl_2), 0.88 mmol) and monooxime **4c** (120 mg, 0.73 mmol). Yield: 61 mg (27 %). R_f = 0.65 (PE-EtOAc, 1:1). Colorless oil. Dynamic mixture of isomers, *E,E*-**2s** : *E,Z*-**2s** : *Z,E*-**2s** : *Z,Z*-**2s** = 3.3 : 1.5 : 2.0 : 1.

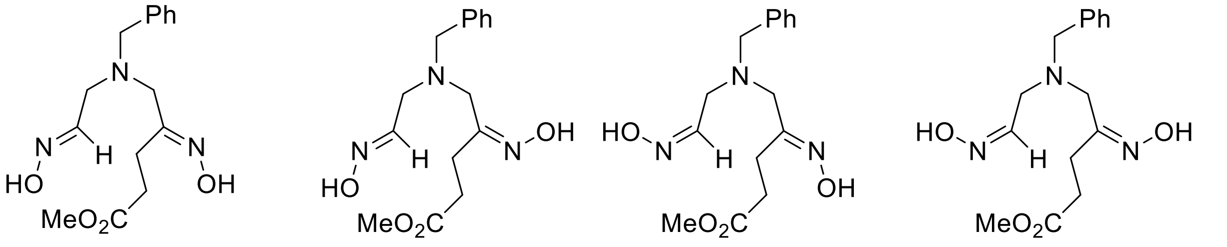

E,E-**2s** *E,Z*-**2s** *Z,E*-**2s** *Z,Z*-**2s**

^{1}H NMR (300 MHz, COSY, $CDCl_3$, *E,E*-isomer) δ 9.49 (s, 2H, 2 NOH), 7.46 (t, J = 6.1 Hz, 1H, C*H*N), 7.31 (m, 5H, Ph), 3.67 (s, 3H, CO_2CH_3), 3.62 (s, 2H, C*H*$_2$Ph), 3.20 (d, J = 6.1 Hz, 2H, C*H*$_2$CHNOH), 3.15 (s, 2H, C*H*$_2$CCH$_2$CH$_2$CO$_2$Me), 2.79 – 2.59 (m, 2H, C*H*$_2$CH$_2$CO$_2$Me), 2.59 – 2.44 (m, 2H, CH$_2$C*H*$_2$CO$_2$Me). ^{13}C{^{1}H} NMR (75 MHz, DEPT135, HSQC, $CDCl_3$, *E,E*-isomer) δ 173.5 (*C*=O), 158.0 (N=*C*CH$_2$CH$_2$CO$_2$Me), 148.9 (H-*C*=N), 138.1 (i-Ph), 129.1 and 128.5 (o-Ph and m-Ph), 127.5 (p-Ph), 58.8 (*C*H$_2$Ph), 56.3 (*C*H$_2$CHNOH), 56.3 (*C*H$_2$CCH$_2$CH$_2$CO$_2$Me), 51.9 (CO$_2$*C*H$_3$), 29.8 (CH$_2$*C*H$_2$CO$_2$Me), 22.2 (*C*H$_2$CH$_2$CO$_2$Me). ^{1}H NMR (300 MHz, COSY, $CDCl_3$, *E,Z*-isomer) δ 9.49 (s, 2H, 2 NOH), 7.48 (t, J = 6.1 Hz, 1H, C*H*N), 7.31 (m, 5H, Ph), 3.66 (s, 3H, CO$_2$C*H*$_3$), 3.62 (s, 2H, C*H*$_2$Ph), 3.40 (s, 2H, C*H*$_2$CCH$_2$CH$_2$CO$_2$Me), 3.20 (d, J = 6.1 Hz, 2H, C*H*$_2$CHNOH), 2.79 – 2.59 (m, 2H, C*H*$_2$CH$_2$CO$_2$Me), 2.59 – 2.44 (m, 2H, CH$_2$C*H*$_2$CO$_2$Me). ^{13}C{^{1}H} NMR (75 MHz, DEPT135, HSQC, $CDCl_3$, *E,Z*-isomer, characteristic signals) δ 173.6 (*C*=O), 148.7 (H-*C*=N), 137.9 (i-Ph), 129.2 and 128.6 (o-Ph and m-Ph), 127.6 (p-Ph), 59.5 (*C*H$_2$Ph), 52.7 (CH$_2$CHNOH), 51.9 (CO$_2$*C*H$_3$), 49.7 (*C*H$_2$CCH$_2$CH$_2$CO$_2$Me), 30.4 (CH$_2$*C*H$_2$CO$_2$Me), 27.7 (*C*H$_2$CH$_2$CO$_2$Me). ^{1}H NMR (300 MHz, COSY, $CDCl_3$, *Z,E*-isomer) δ 9.49 (s, 2H, 2 NOH), 7.31 (m, 5H, Ph), 6.87 (t, J = 4.4 Hz, 1H, C*H*N), 3.67 (s, 3H, CO$_2$C*H*$_3$), 3.62 (s, 2H, C*H*$_2$Ph), 3.42 (d, J = 4.4 Hz, 2H, C*H*$_2$CHNOH), 3.15 (s, 2H,

CH_2CCH_2CH_2CO_2Me), 2.79 – 2.59 (m, 2H, C*H*$_2$CH_2CO_2Me), 2.59 – 2.44 (m, 2H, CH_2C*H*$_2$CO_2Me). ^{13}C{^{1}H} NMR (75 MHz, DEPT135, HSQC, $CDCl_3$, *Z,E*-isomer) δ 173.4 (*C*=O), 157.9 (N=*C*CH_2CH_2CO_2Me), 150.6 (H-*C*=N), 138.0 (i-Ph), 129.1 and 128.6 (o-Ph and m-Ph), 127.5 (p-Ph), 59.6 (*C*H_2Ph), 57.2 (*C*H_2CCH_2CH_2CO_2Me), 51.9 (CO_2*C*H_3), 48.5 (CH_2CHNOH), 29.8 (CH_2*C*H_2CO_2Me), 22.2 (*C*H_2CH_2CO_2Me). ^{1}H NMR (300 MHz, COSY, $CDCl_3$, *Z,Z*-isomer) δ 9.49 (s, 2H, 2 NOH), 7.31 (m, 5H, Ph), 6.91 (t, J = 4.4 Hz, 1H, C*H*N), 3.67 (s, 3H, CO_2C*H*$_3$), 3.62 (s, 2H, C*H*$_2$Ph), 3.47 (s, 2H, C*H*$_2$CCH_2CH_2CO_2Me), 3.42 (d, J = 4.4 Hz, 2H, C*H*$_2$CHNOH), 2.79 – 2.59 (m, 2H, C*H*$_2$CH_2CO_2Me), 2.59 – 2.44 (m, 2H, CH_2C*H*$_2$CO_2Me). ^{13}C{^{1}H} NMR (75 MHz, DEPT135, HSQC, $CDCl_3$, *Z,Z*-isomer, characteristic signals) δ 173.1 (*C*=O), 150.3 (H-*C*=N), 138.3 (i-Ph), 61.4 (*C*H_2Ph), 51.9 (CO_2*C*H_3). HRMS (ESI): m/z calcd. for $[C_{15}H_{22}N_3O_4]^+$ 308.1605, found 308.1603 $[M + H]^+$.

4. Stereochemistry elucidation in 2,6-disubstituted piperazines 1

Hydrogenation of diketooximes **2i-o** afforded the corresponding 2,6-disubstituted piperazines **1i-o** (free or *N*-protected) predominantly as *cis*-isomers.

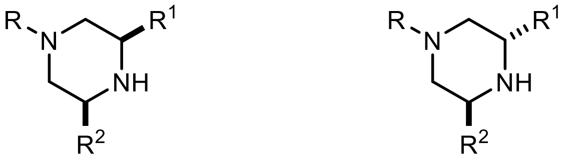

cis-**1**, ***major*** *trans*-**1**, *minor*

Unfortunately, 2D NOESY and $^3J_{H\text{-}H}$ coupling constants data, which are typically used to establish the relative position of substituents in six-membered rings, are not informative in the case of symmetrically 2,6-disubstituted piperazines. Thus, the assignment of stereochemistry in piperazines **1** is challenging and several considerations were taken into account:

1) Primarily, the *cis*-configuration of the obtained piperazines was confirmed by a comparison of the NMR spectra of with those previously reported for *cis*-**1n** and *trans*-**1o**. The NMR spectra of the obtained product **1n** matches with known *cis*-**1n** [11]. It is logical to assume that the major isomer in other 2,6-dimethyl-substituted piperazines (R^1, R^2 = Me, **1i-m**) is identical to *cis*-**1n**. The NMR spectra of the obtained product **1o** (R^1, R^2 = Ph) does not match with known *trans*-**1o** [8] suggesting that *cis*-**1o** was formed in the reaction.

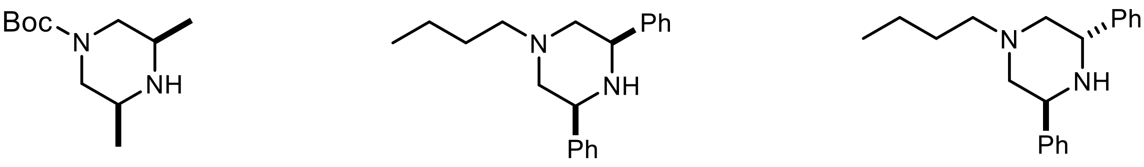

cis-**1n** — obtained, known

cis-**1o** — obtained

trans-**1o** — known

2) In the ^{1}H NMR spectra of the major isomer of EtC(O)-**1k** the CH_2 groups appear as four signals. This is consistent with the expected four diastereotopic hydrogens in the *cis*-isomer. On the contrary, in the minor isomer of EtC(O)-**1k** only two signals of CH_2 groups are observed. This is consistent with the *trans*-isomer, which has only two types of non-equivalent hydrogens.

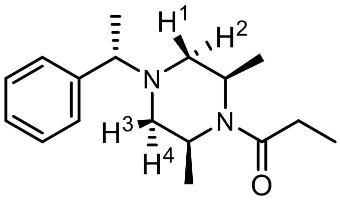

cis-EtCO-**1k**

$H^1 \neq H^2 \neq H^3 \neq H^4$

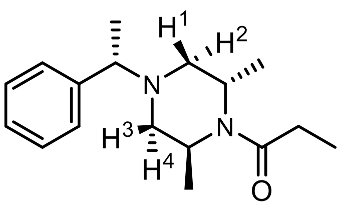

trans-EtCO-**1k**

$H^1 = H^4$, $H^2 = H^3$

3) The appearance of NMR spectra of *cis*- and *trans*-isomers of 2,6-disubstituted piperazines can provide indirect evidence for the assignment of stereochemistry. Thus, in the ^{1}H NMR of *cis*-isomers of propionyl-protected piperazines EtC(O)-**1i-m** the H-2 and H-6 hydrogens appear as two very broad multiplets at 3.7-4.7 ppm, while in the *trans*-isomers these protons appear as a sharp multiplet at ca. 4 ppm. Based on the literature [14, 15, 16] and our NMR data ($^3J_{H\text{-}H} < 5$ Hz for CH_2 protons of the piperazine ring), methyl groups in *cis*-2,6-dimethylpiperazine derivatives occupy axial positions. Hence, broadening of H-2 and H-6 signals in the *cis*-isomers is likely to be a consequence of the restricted interconversion of between amide *E*/*Z* conformers [17] due to a sterical hindrance created by two axial methyl groups. In the *trans*-2,6-dimethylpiperazine derivatives, the interconversion between amide *E*/*Z* conformers is expected to take place more easily. The observed spectral features are in line with previously reported NMR data for *N*-acyl derivatives of *cis*- and *trans*-2,6-dimethylpiperazine derivatives [18].

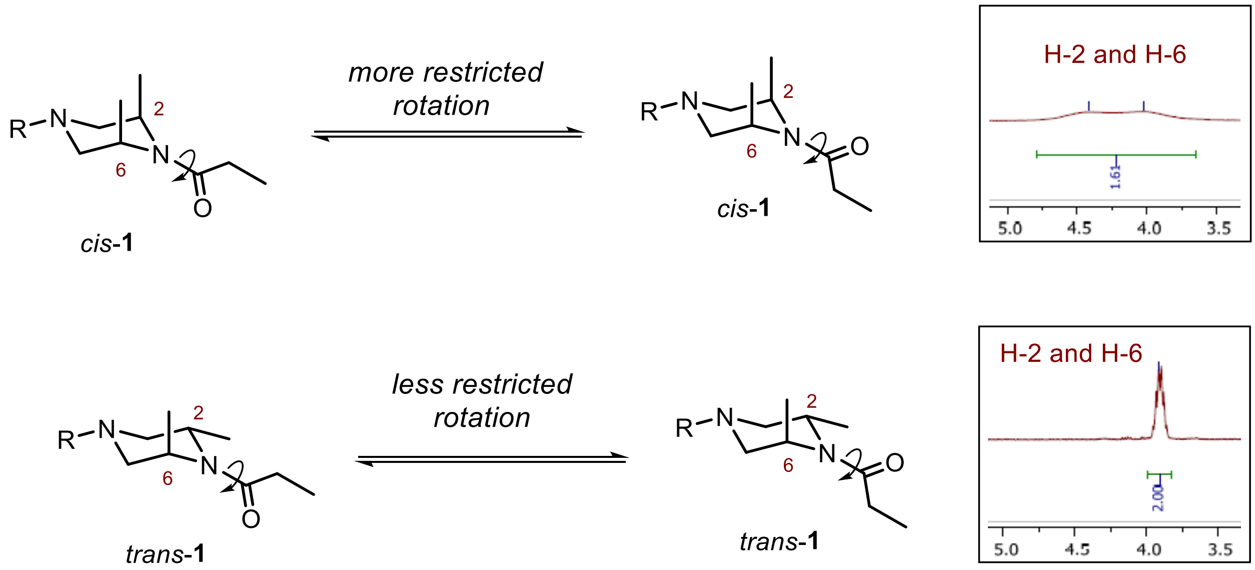
more restricted rotation
cis-1
cis-1
H-2 and H-6
1.61
5.0 4.5 4.0 3.5
less restricted rotation
trans-1
trans-1
H-2 and H-6
2.00
5.0 4.5 4.0 3.5

5. Assignment of *E*/*Z*-configuration and determination of isomer ratio in dioximes 2

For the majority of dioximes **2** signals of *E*- and *Z*-oxime moieties are present in the NMR spectra. The mixture is dynamic, the isomeric ratio changes with time and depends on the solvent.

The stereochemical assignment of oxime groups was performed on the basis of known relationships between the configuration of the C=N bond and chemical shifts of neighboring atoms in ^{1}H and ^{13}C NMR spectra [13, 19]. The configuration assignment was additionally confirmed by 2D ^{1}H–^{1}H NOESY correlations and $^{1}J_{CH}$ coupling constants in the C(N)H unit for dioxime **2a**. For determination of $^{1}J_{CH}$ coupling constants, ^{1}H-^{13}C HSQC experiment was performed without decoupling on ^{1}H nuclei (see copies of NMR spectra section). It was shown that for the *E*-fragment in the *E*,*E*-isomer and *E*,*Z*-isomer, the constant $^{1}J_{CH}$ is 170.8 Hz and 150.6 Hz, respectively. For the *Z*-fragment in the *E*,*Z*-isomer, the constant $^{1}J_{CH}$ is 186.2 Hz. This correlates with the $^{1}J_{CH}$ constants for oxime isomers reported in the literature [20].

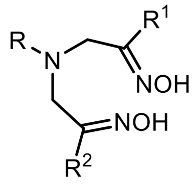

2

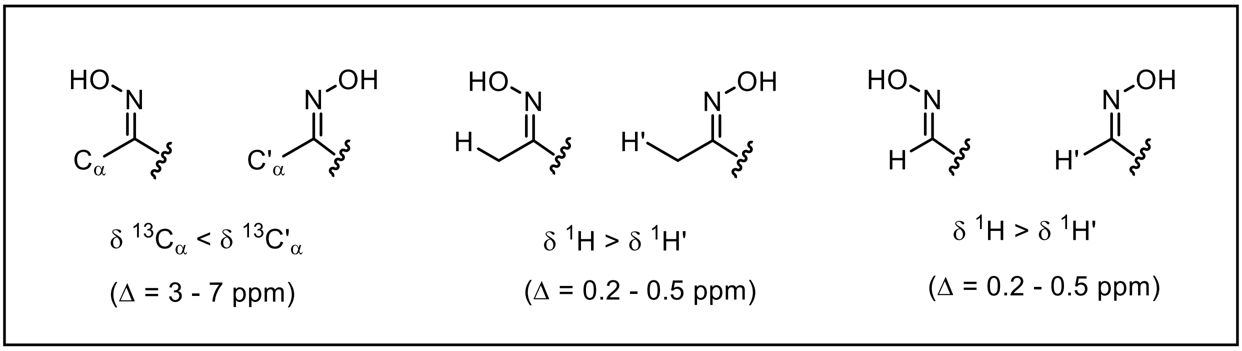

Generally, the chemical shifts of same oxime moieties are conservative and do not depend much on the nature of substituent on the tertiary amine nitrogen as can be seen from the Tables S1 and S2.

Table S1. ^{1}H NMR shifts of aldoxime fragment in isomers of dioximes **2a-h**, **q-s** in ppm.

**Dioxime**	***E*,*E*-2**		***E*,*Z*-2 (*E*-fragment)**		***E*,*Z*-2 (*Z*-fragment)**		***Z*,*Z*-2**	
	δ =CH	δ CH_2	δ =CH	δ CH_2	δ =CH	δ CH_2	δ =CH	δ CH_2
**2a**	7.48	3.28	7.49	3.27	6.90	3.47	6.90	3.48
**2b**	7.36	3.34	7.40	3.31	6.80	3.51	6.88	3.53
**2c**	7.47	3.34	7.50	3.34	6.92	3.53	6.96	3.54
**2d**	7.45	3.27	7.45	3.27	6.87	3.47	7.56	4.28
**2e**	7.45	3.26	7.46	3.24	6.87	3.47	6.91	3.47
**2f**	7.42	3.27	7.44	3.27	6.87	3.51	6.91	3.51
**2g**	7.27	3.30	7.28	3.37	6.77	3.48	6.79	3.42
**2h**	7.32	3.41	7.42	3.27	6.74	3.58	6.80	3.64
**2p**	7.41	3.19	7.44	3.19	6.82	3.39	6.87	3.38
**2q**	7.43	3.19	7.46	3.21	6.83	3.41	6.88	3.43
**2r**	7.37	3.21	7.35	3.16	6.75	3.41	6.67	3.39
**2s**	7.46	3.20	7.48	3.20	6.87	3.42	6.91	3.42

Table S2. ^{1}H NMR shifts of ketooxime fragment in isomers of dioximes **2i-n**, **p-s** in ppm.

**Dioxime**	***E,E*-2**		***E,Z*-2 (*E*-fragment)**		***E,Z*-2 (*Z*-fragment)**		***Z,Z*-2**	
	δ CH_2	δ CH_3	δ CH_2	δ CH_3	δ CH_2	δ CH_3	δ CH_2	δ CH_3
**2i**	3.02	1.90	3.05	1.90	3.30	1.92		
**2j**	3.08	1.88	3.10	1.92	3.37	1.88		
**2k**	3.06	1.87	2.99	1.90	3.52	1.91		
**2l**	3.22	1.91	3.24	1.93	3.47	1.92		
**2m**	3.08	1.93	3.08	1.93	3.36	1.93		
**2n**	3.79	1.68	3.83	1.70	3.96	1.70	4.01	1.73
**2p**	3.11	1.90	3.11	2.16	3.11	1.92	3.11	1.90
**2q**	3.11		3.41		3.40		3.40	
**2r**	3.09		3.08		3.33		3.60	
**2s**	3.15		3.15		3.40		3.47	

The assignment of individual stereoisomers and determination of isomeric ratio was carried out as follows. For symmetrically substituted dioximes **2** ($R^1 = R^2$) three isomers (*E*,*E*-**2**, *E*,*Z*-**2** and *Z*,*Z*-**2**) are theoretically possible. Unsymmetrically substituted ($R^1 \neq R^2$) can exist as forms (*E*,*E*-**2**, *E*,*Z*-**2**, *Z*/*E*-**2** and *Z*,*Z*-**2**). The number of isomers present in the mixture can be determined by the analysis of ^{13}C NMR spectra, in particular from the number of signals of sp^2 carbons of the oxime group. For dioximes **2** two to four isomers were detected. Based on the aforementioned relationships between ^{13}C chemical shifts and the *E*/*Z*-configuration, groups of signals corresponding separately to *E*- and *Z*-fragments could be identified (at least for C=N, CH_2 and CH_3 carbons). From the number of signals in each group, individual isomers can be identified in the mixture. Thus, in the symmetrically substituted dioximes **2a-o** the presence of two signals of *E*-fragments (e.g. C=N carbons) indicates the presence of both *E*,*E*- and *E*,*Z*-isomers. Accordingly, the presence of two sets signals corresponding to *Z*-fragment indicates the presence of *Z*,*Z*- and *E*,*Z*-isomers.

To determine the isomer ratio, the assignment of ^{1}H NMR signals of *E* and *Z* oxime fragments to individual *E*,*E*-, *E*,*Z*-, *Z*/*E*- и *Z*,*Z*-isomers was performed. In complicated cases (e.g. unsymmetrically substituted dioximes), 2D NMR techniques were used for this purpose. In dialdooximes **2a-h**, the signals of C(N)H hydrogens in the *Z*-fragments were often resolved. The minor signal of *Z*-fragment was assigned to *Z*,*Z*-isomer, while the major one corresponded to *E*,*Z*-isomer (integration gave the *E*,*Z*/*Z*,*Z* ratio). The amount of *E*,*E*-isomer was calculated accordingly from the overall integral of *E*-C(N)H fragment minus the integral of *E*,*Z*-isomer. In the case of unsymmetrically substituted keto-aldo-oximes **2p-s**, resolved signals of protons of methyl or methylene groups corresponding to the *E*-oximino fragment were needed for quantification.

For the diketooximes **2i-o**, only two isomers, namely *E*,*E* and *E*,*Z*, were present in the mixture. The absence of the *Z*,*Z*-isomer was indicated by the presence of only one ^{13}C NMR signal in the region of *Z*-C=N bond and two signals of the *E*-C=N bond (same for the methyl groups). Determination of the isomer ratio was accomplished by integrating the individual signals of the CH_2 groups belonging to the *Z*- and *E*-fragments in the ^{1}H NMR spectra and calculated using the formula:

E,*E* : *E*,*Z* = [**I**(all *E*-CH_2)–**I**(Z-CH_2)]/[**I**(Z-CH_2)×2], where **I** – integral of signal in ^{1}H NMR.

An example of ^{1}H and ^{13}C NMR spectra with the assignment of individual isomers and calculation of their ratio is shown below:

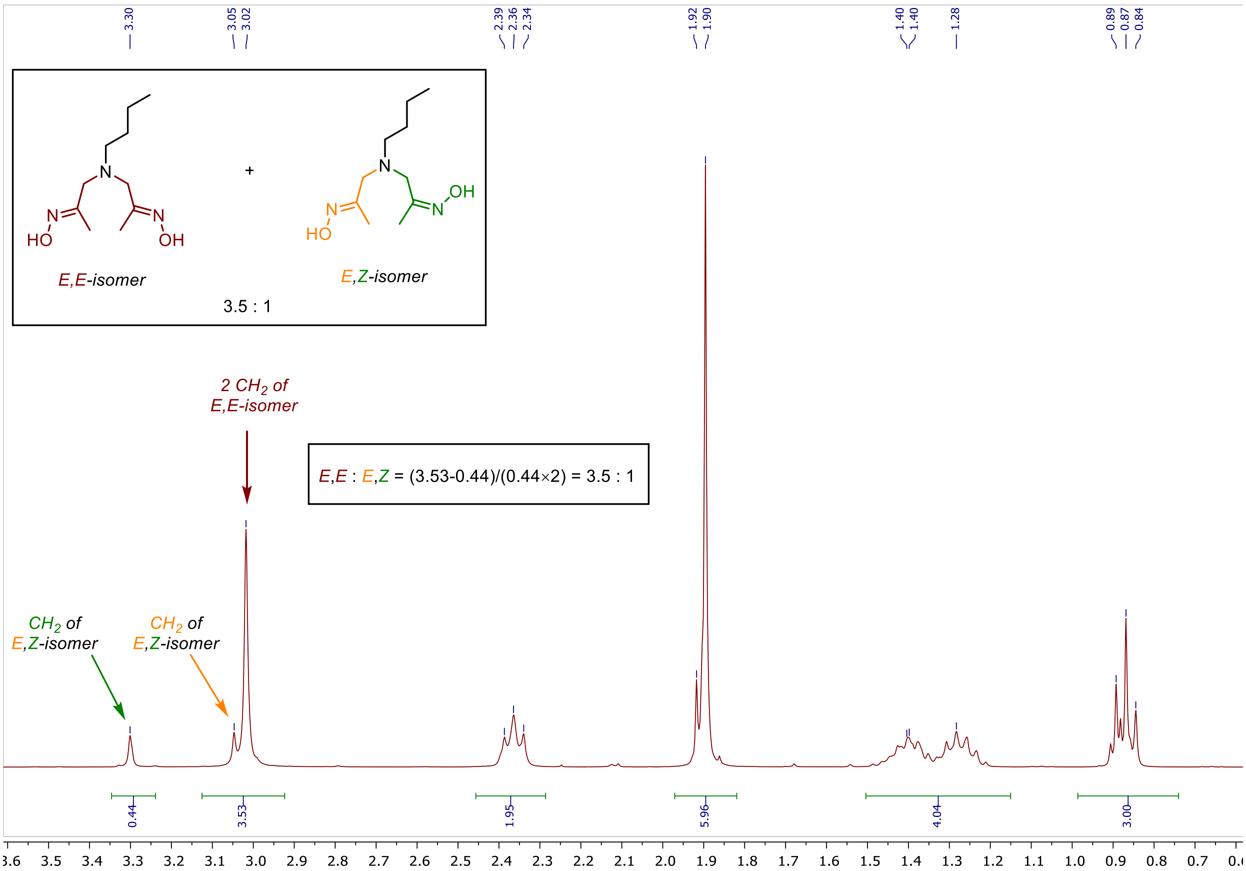

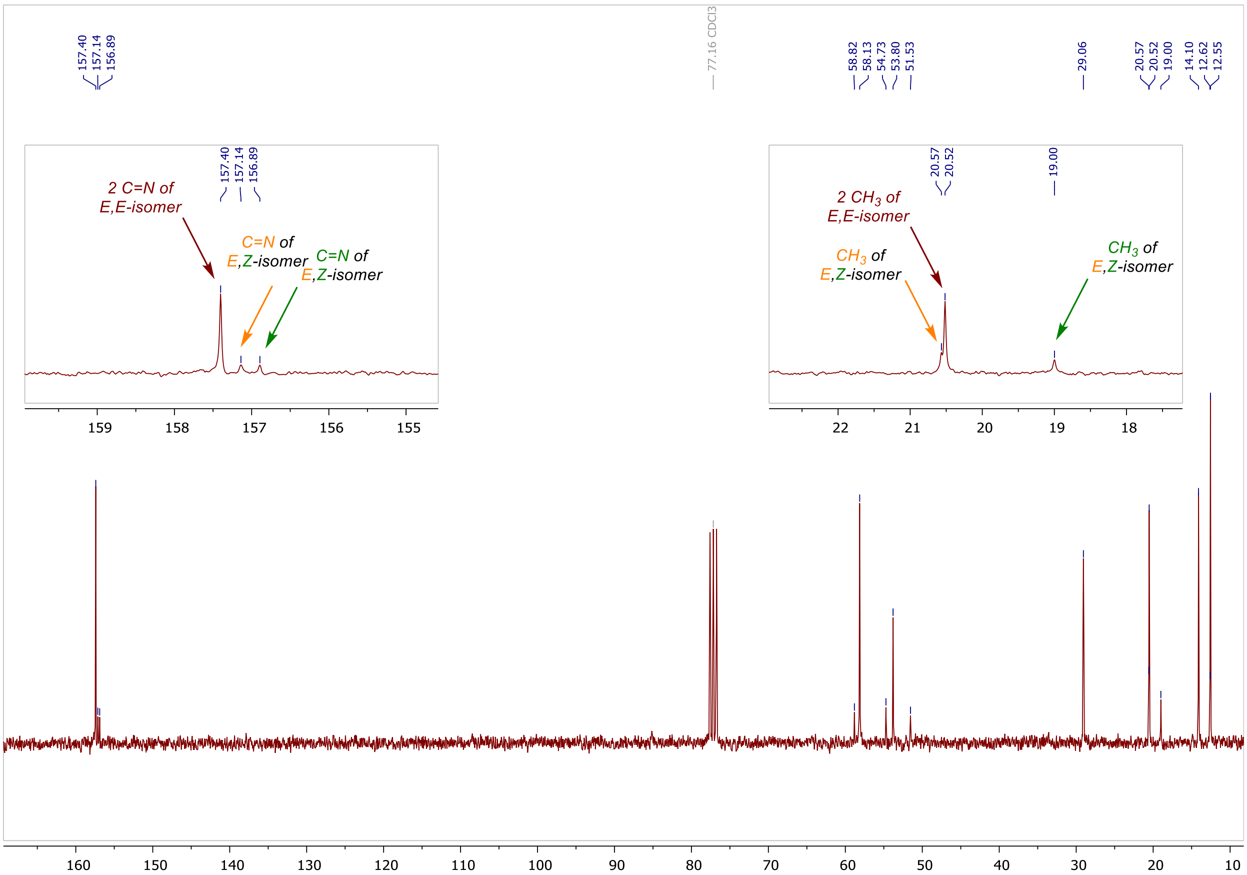

6. Copies of NMR spectra

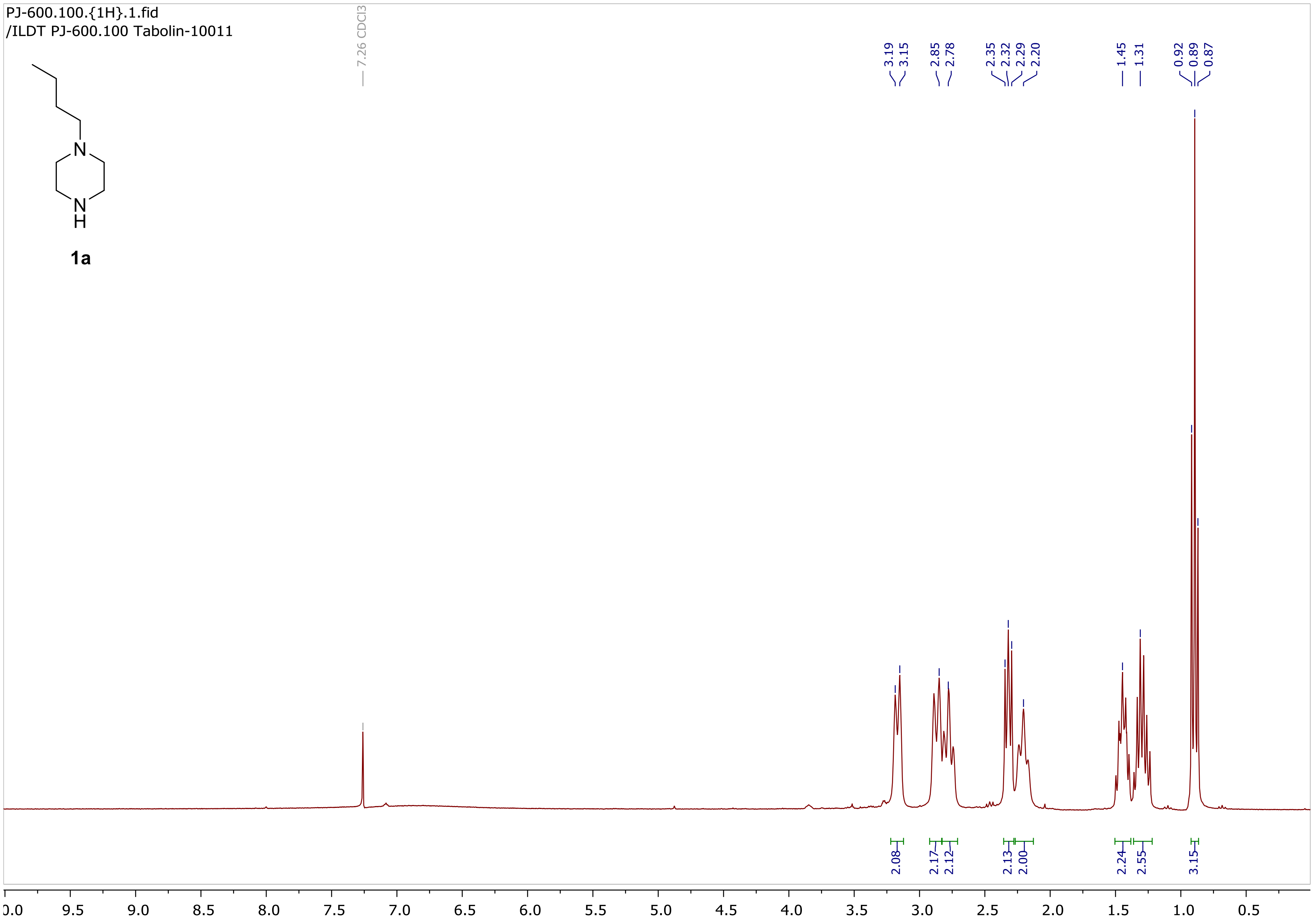
PJ-600.100.{1H}.1.fid
/ILDT PJ-600.100 Tabolin-10011
7.26 CDCl3
3.19
3.15
2.85
2.78
2.35
2.32
2.29
2.20
1.45
1.31
0.92
0.89
0.87
2.08
2.17
2.12
2.13
2.00
2.24
2.55
3.15
9.5
9.0
8.5
8.0
7.5
7.0
6.5
6.0
5.5
5.0
4.5
4.0
3.5
3.0
2.5
2.0
1.5
1.0
0.5

1a

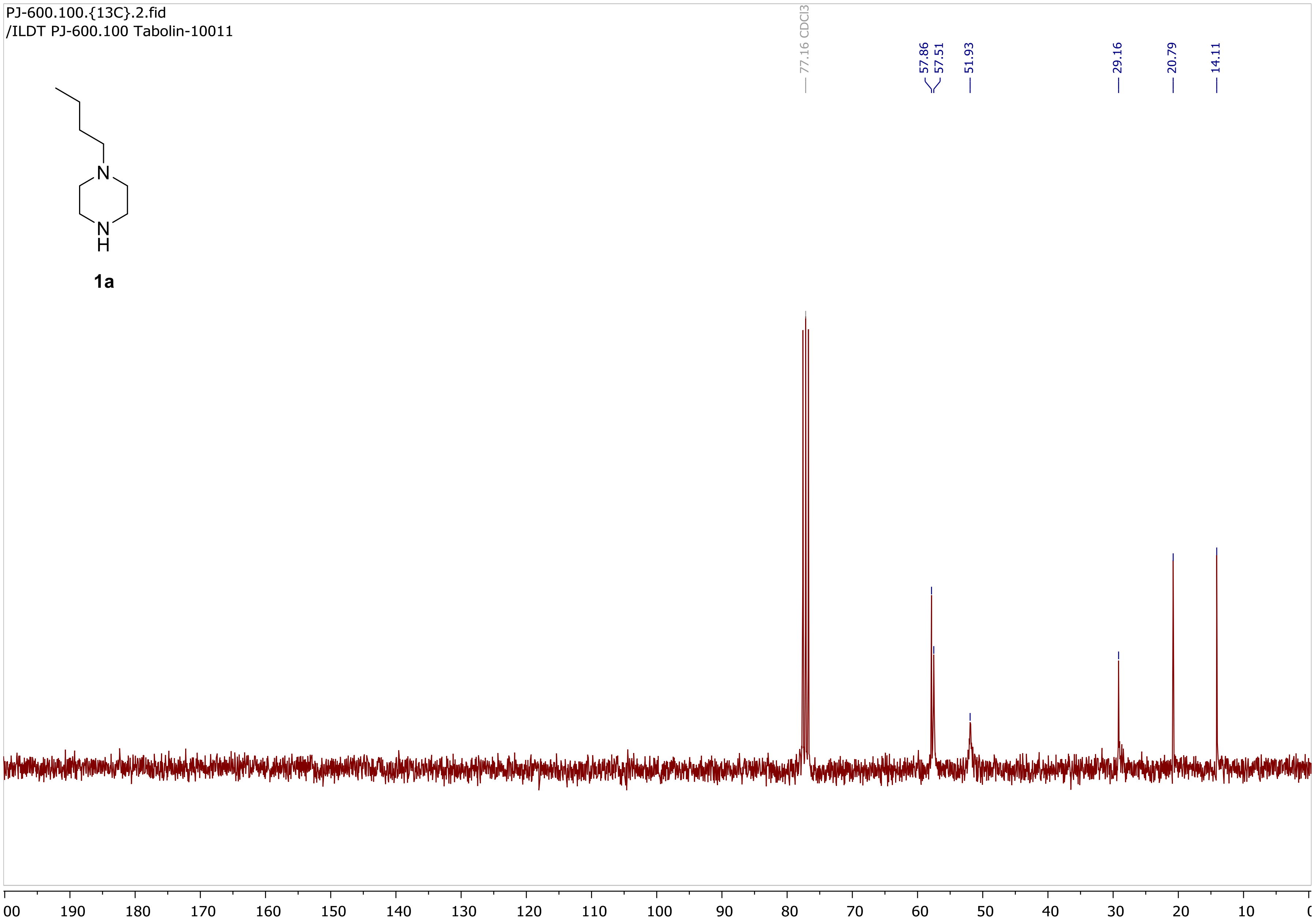
PJ-600.100.{13C}.2.fid
/ILDT PJ-600.100 Tabolin-10011
77.16 CDCl3
57.86
57.51
51.93
29.16
20.79
14.11
N
N
H
1a
00 190 180 170 160 150 140 130 120 110 100 90 80 70 60 50 40 30 20 10

PJ-600.100.{13C}deptsp135.3.fid
/ILDT PJ-600.100 Tabolin-10011

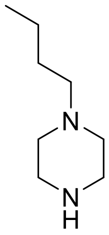

1a

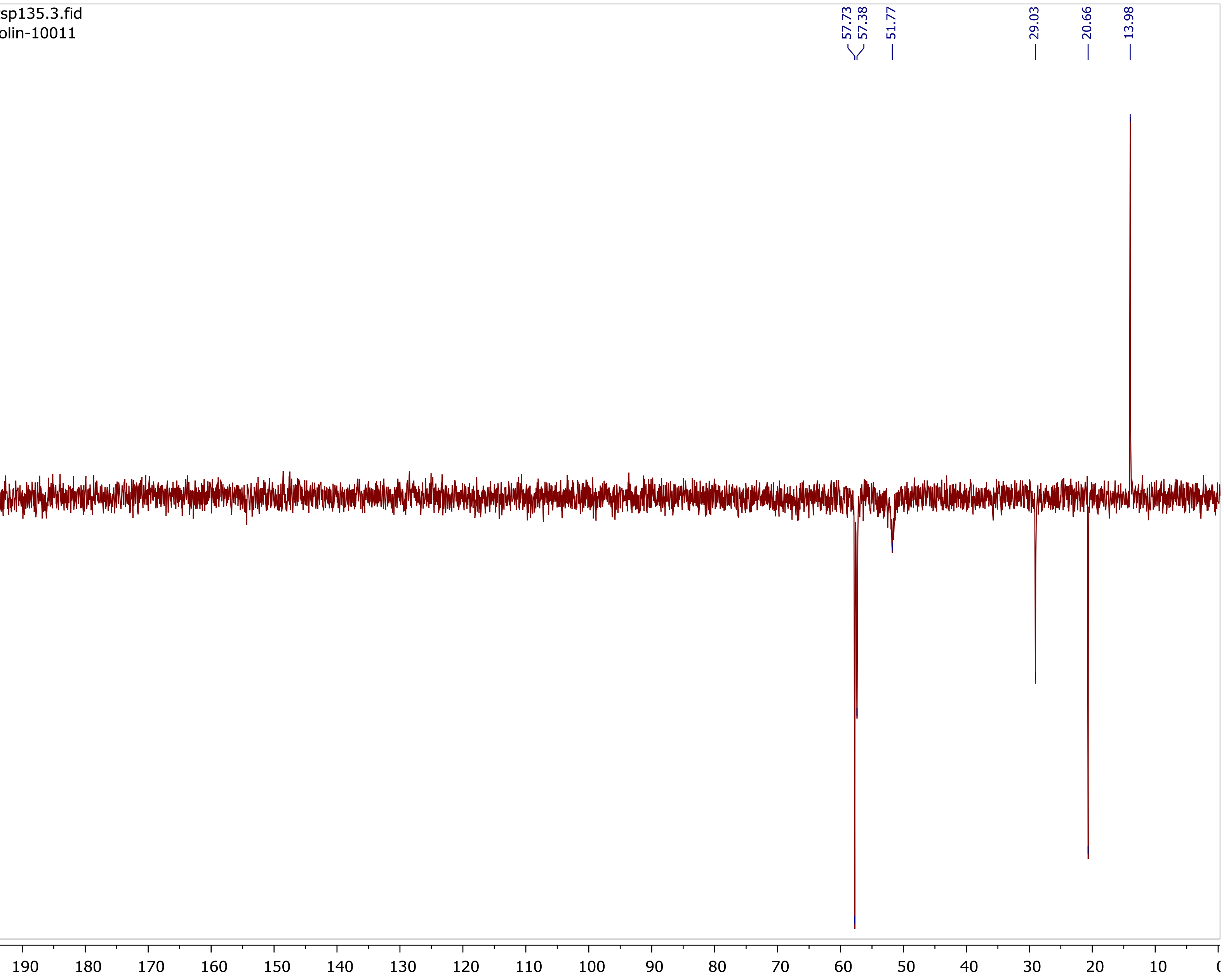

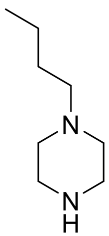

1a

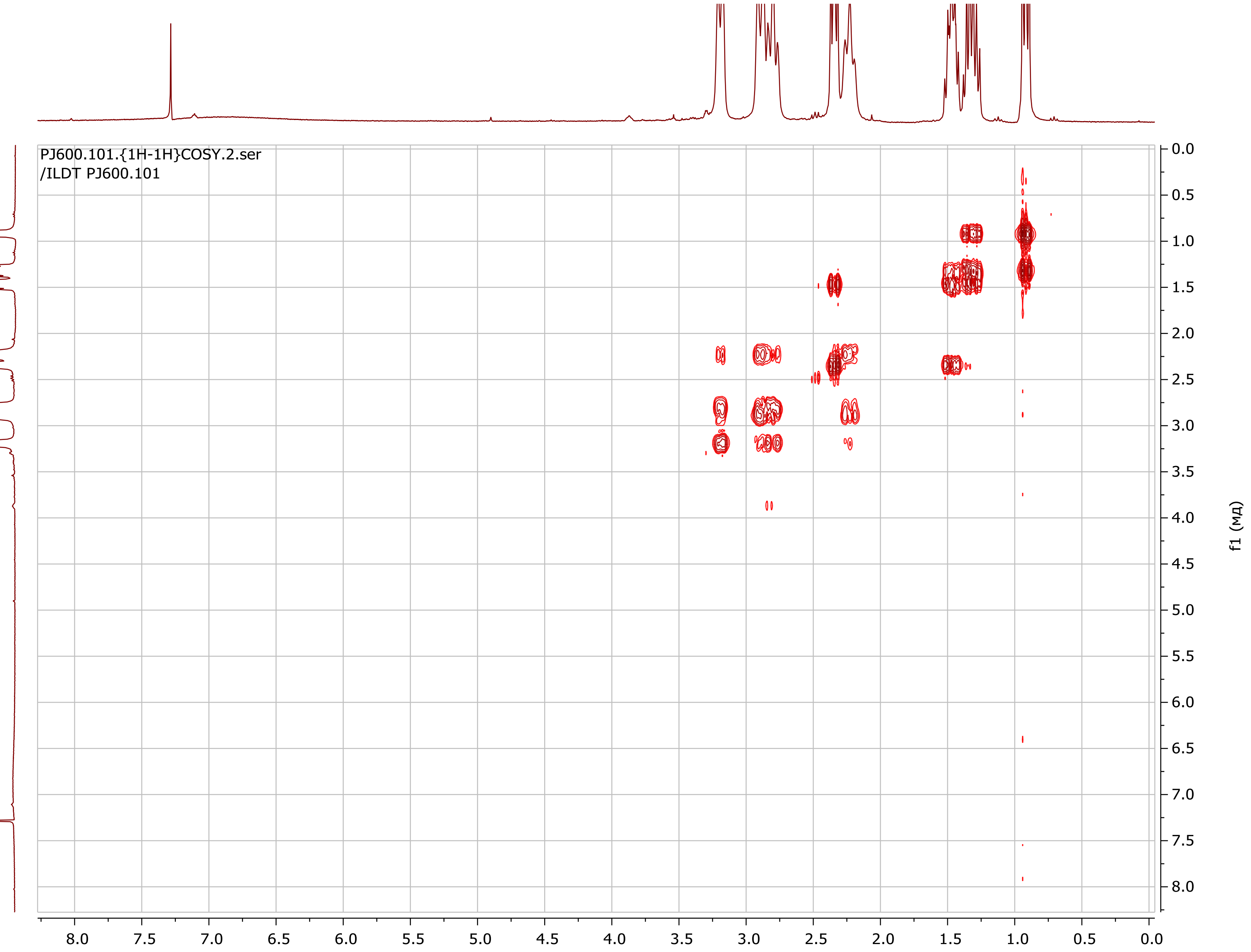

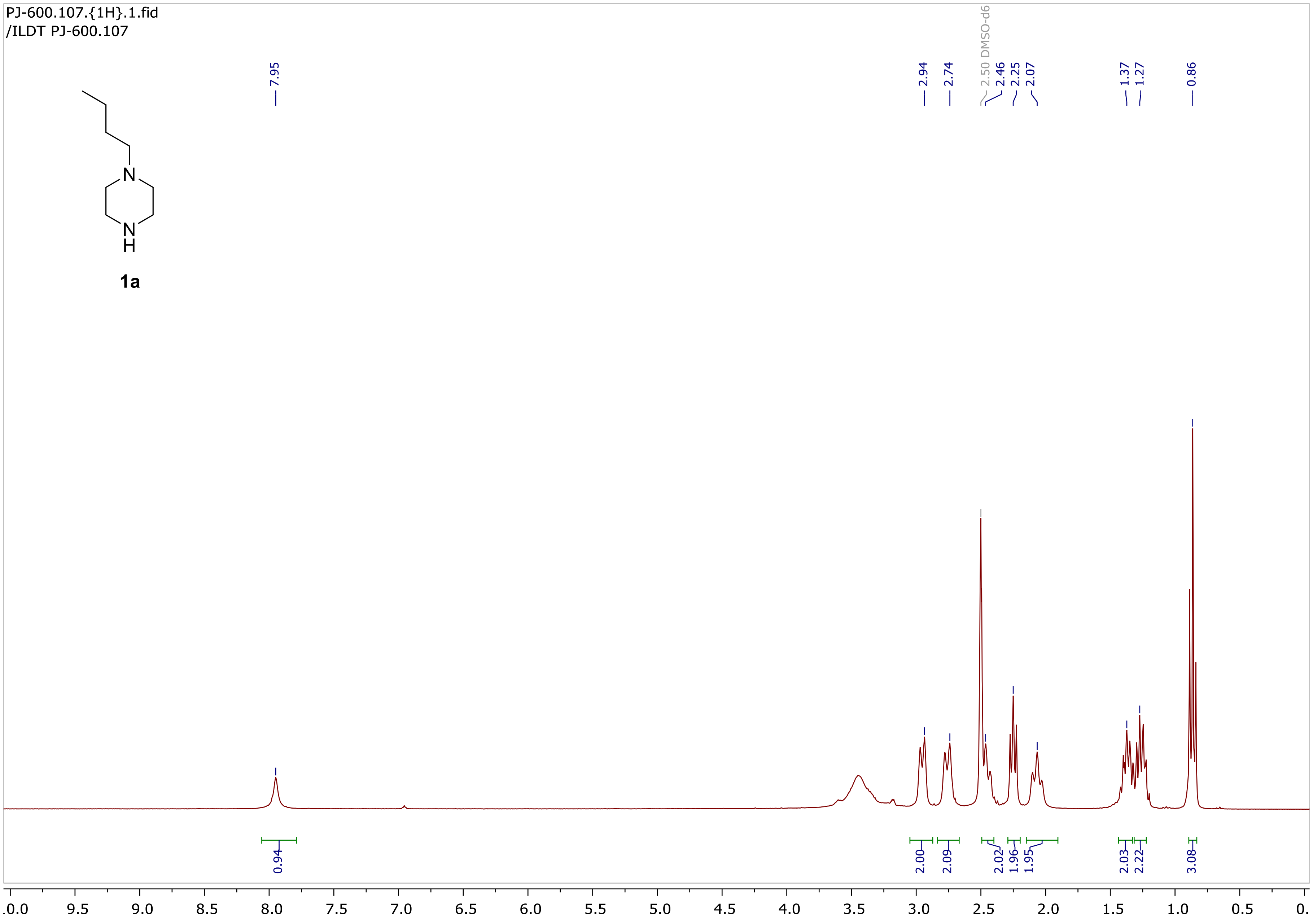

PJ-600.107.{1H}.1.fid
/ILDT PJ-600.107
1a
7.95
2.94
2.74
2.50 DMSO-d6
2.46
2.25
2.07
1.37
1.27
0.86
0.94
2.00
2.09
2.02
1.96
1.95
2.03
2.22
3.08
.0.0
9.5
9.0
8.5
8.0
7.5
7.0
6.5
6.0
5.5
5.0
4.5
4.0
3.5
3.0
2.5
2.0
1.5
1.0
0.5
0.

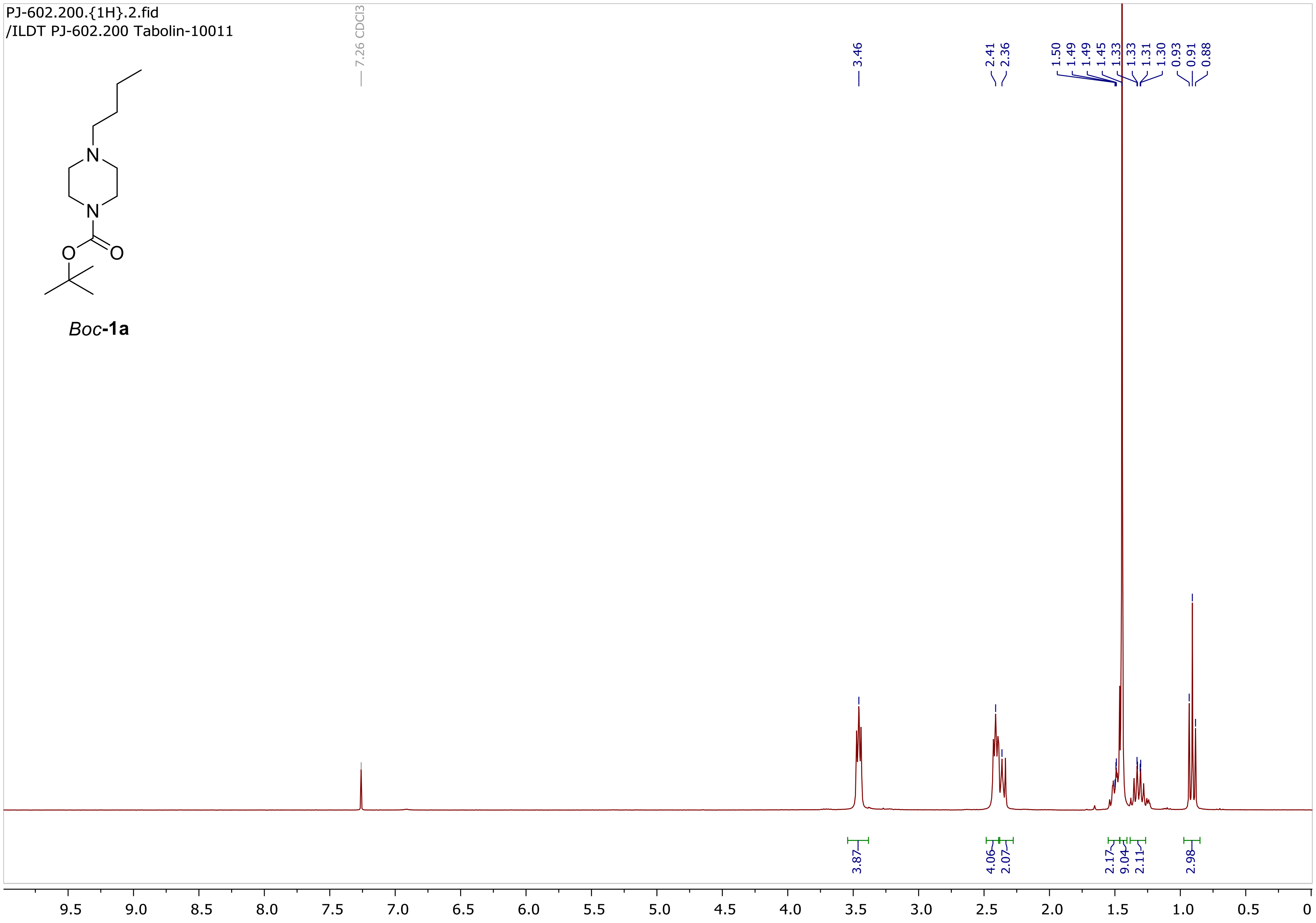
PJ-602.200.{1H}.2.fid
/ILDT PJ-602.200 Tabolin-10011
7.26 CDCl3
3.46
2.41
2.36
1.50
1.49
1.49
1.45
1.33
1.33
1.31
1.30
0.93
0.91
0.88
N
N
O
O
Boc-1a
3.87
4.06
2.07
2.17
9.04
2.11
2.98
9.5 9.0 8.5 8.0 7.5 7.0 6.5 6.0 5.5 5.0 4.5 4.0 3.5 3.0 2.5 2.0 1.5 1.0 0.5 0

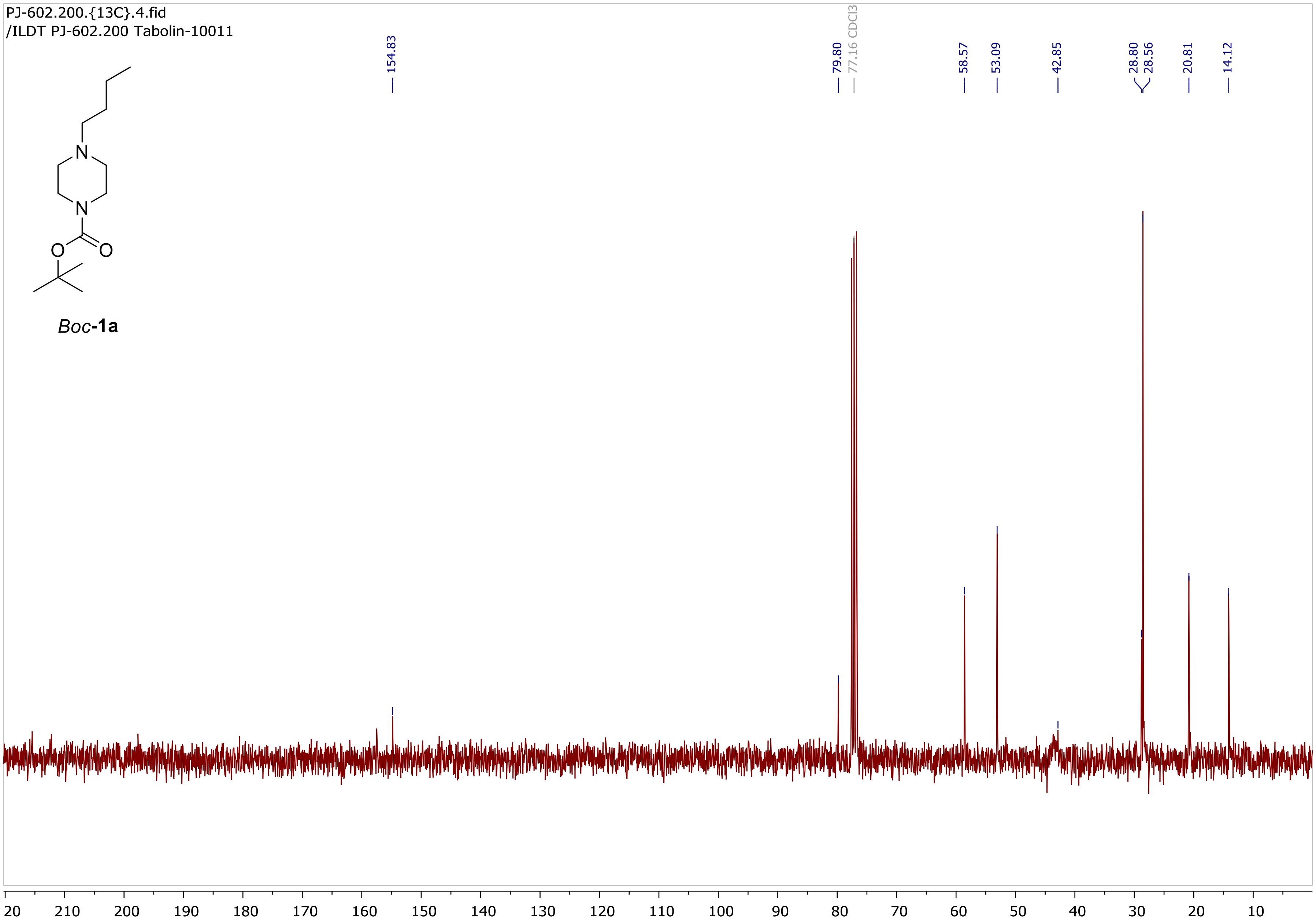

PJ-602.200.{13C}.4.fid
/ILDT PJ-602.200 Tabolin-10011
154.83
79.80
77.16 CDCl3
58.57
53.09
42.85
28.80
28.56
20.81
14.12
Boc-1a
20
210
200
190
180
170
160
150
140
130
120
110
100
90
80
70
60
50
40
30
20
10

PJ-602.200.{13C}deptsp135.5.fid
/ILDT PJ-602.200 Tabolin-10011

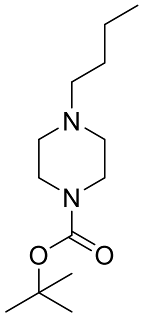

Boc-**1a**

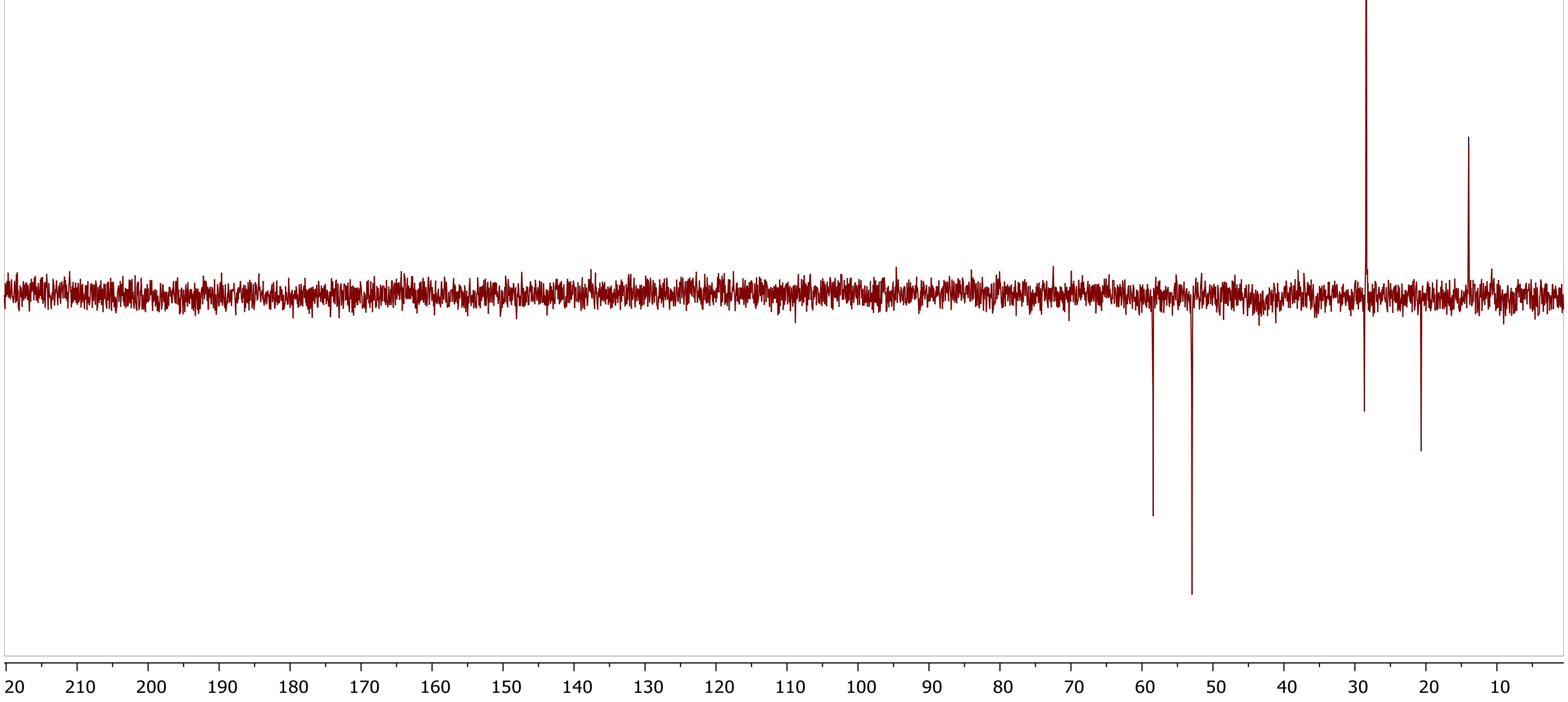

PJ-638.200.{1H}.1.fid
Avance-300, CDCl3

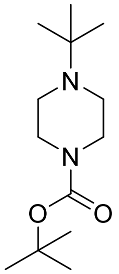

Boc-**1b**

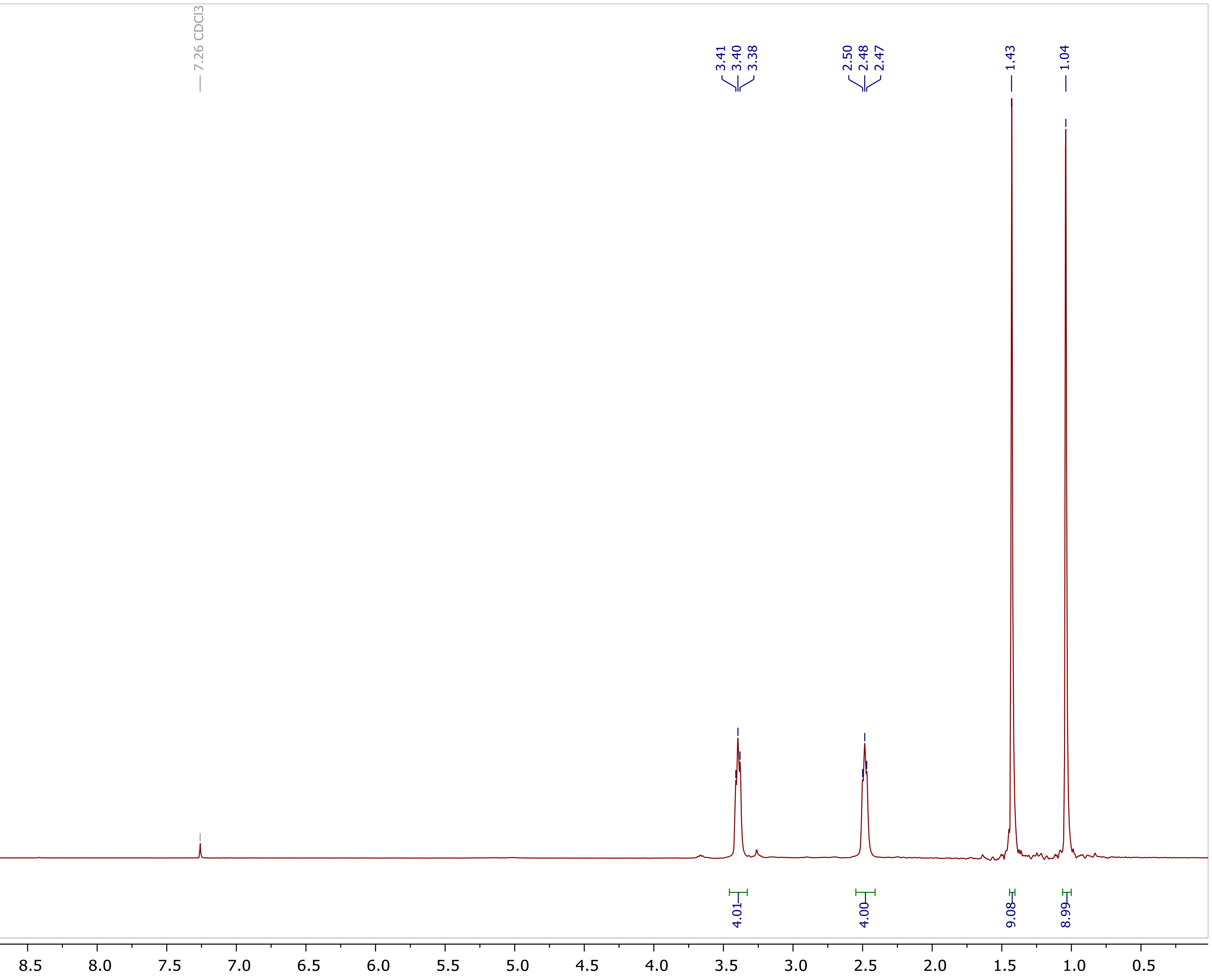

PJ-638.200.{13C}.1.fid
/ILDT PJ-638.200 Tabolin-10011

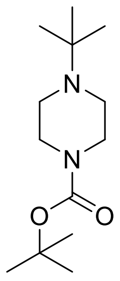

Boc-**1b**

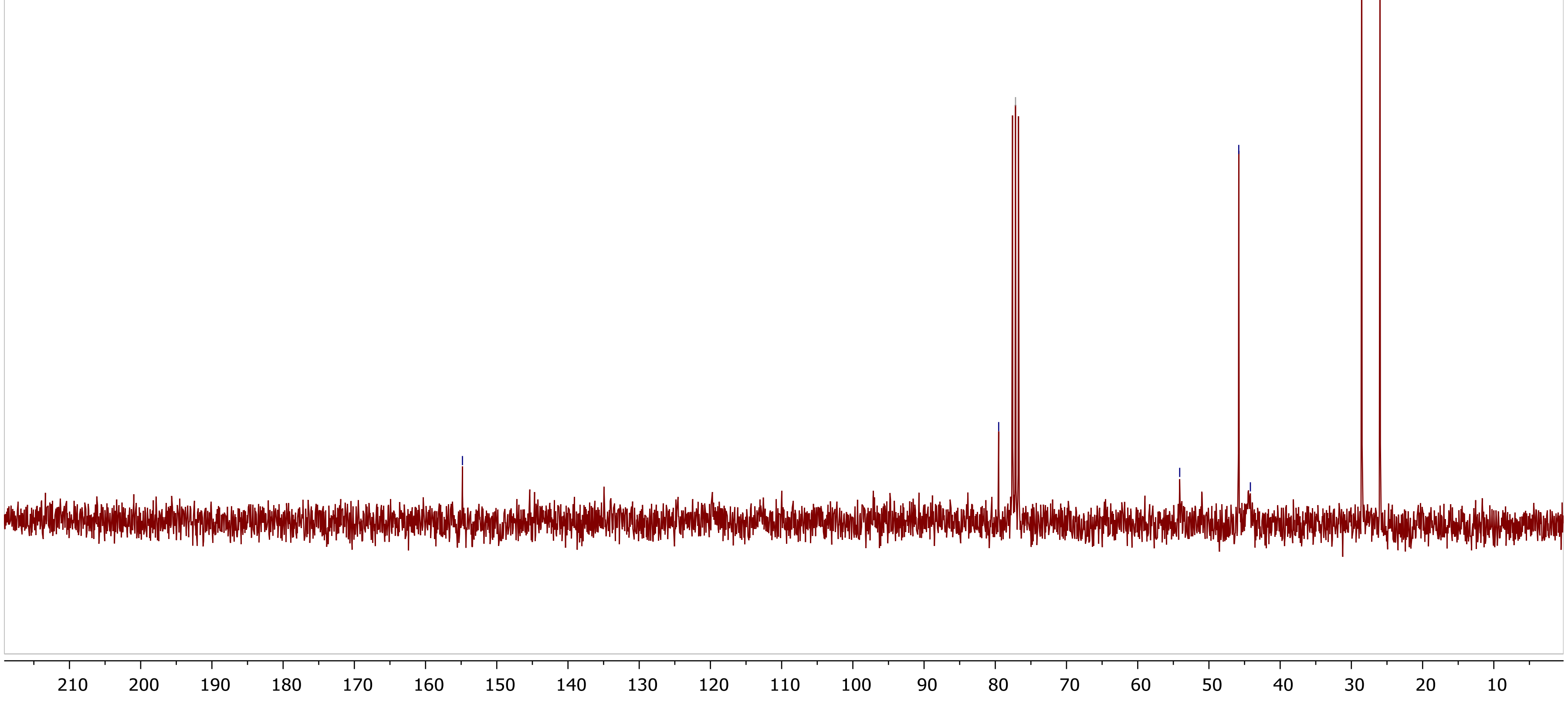

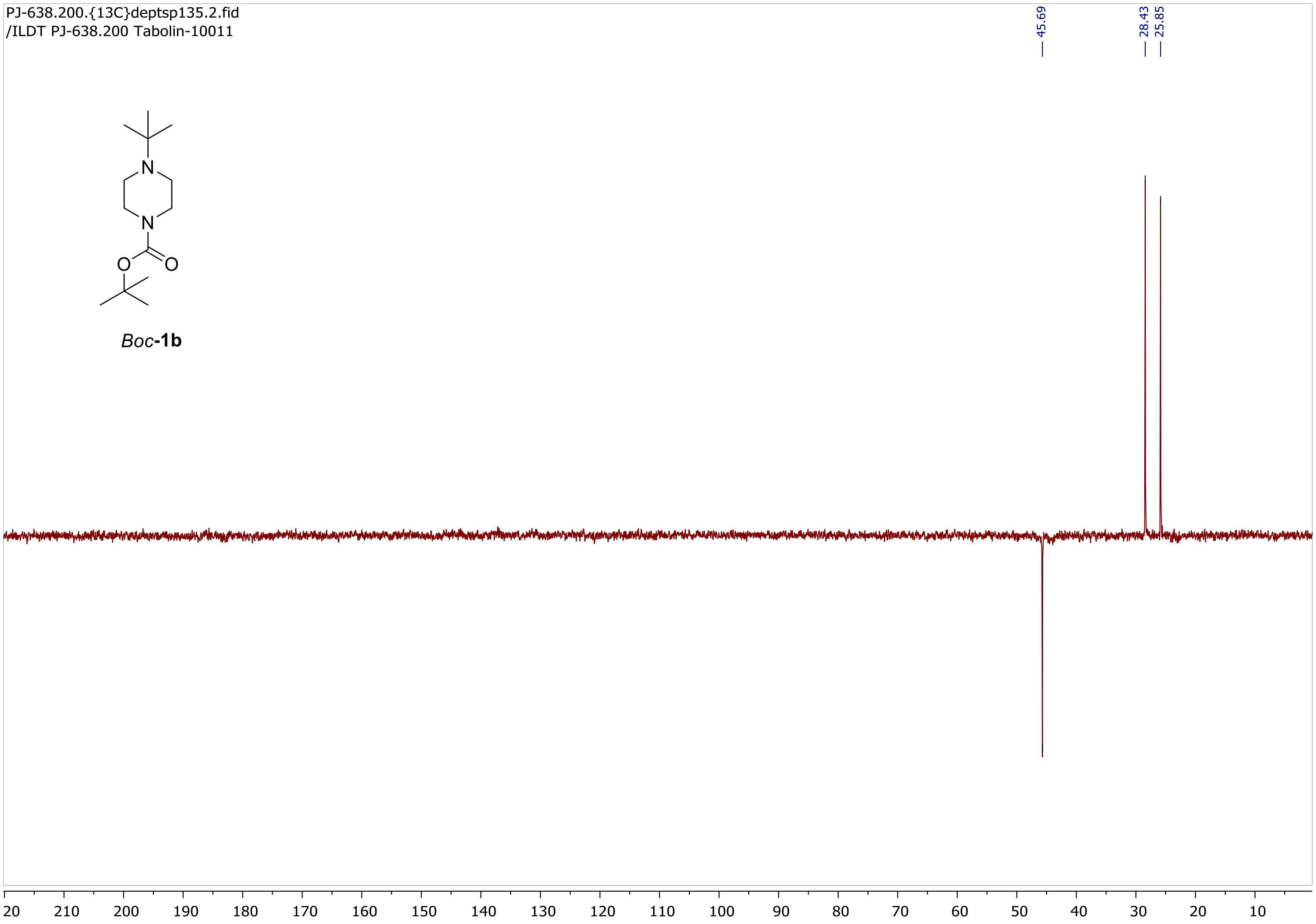
PJ-638.200.{13C}deptsp135.2.fid
/ILDT PJ-638.200 Tabolin-10011
45.69
28.43
25.85
N
N
O
O
Boc-**1b**
20
210
200
190
180
170
160
150
140
130
120
110
100
90
80
70
60
50
40
30
20
10

PJ-653.200.{1H}.1.fid
/ILDT PJ-653.200 Tabolin-10011

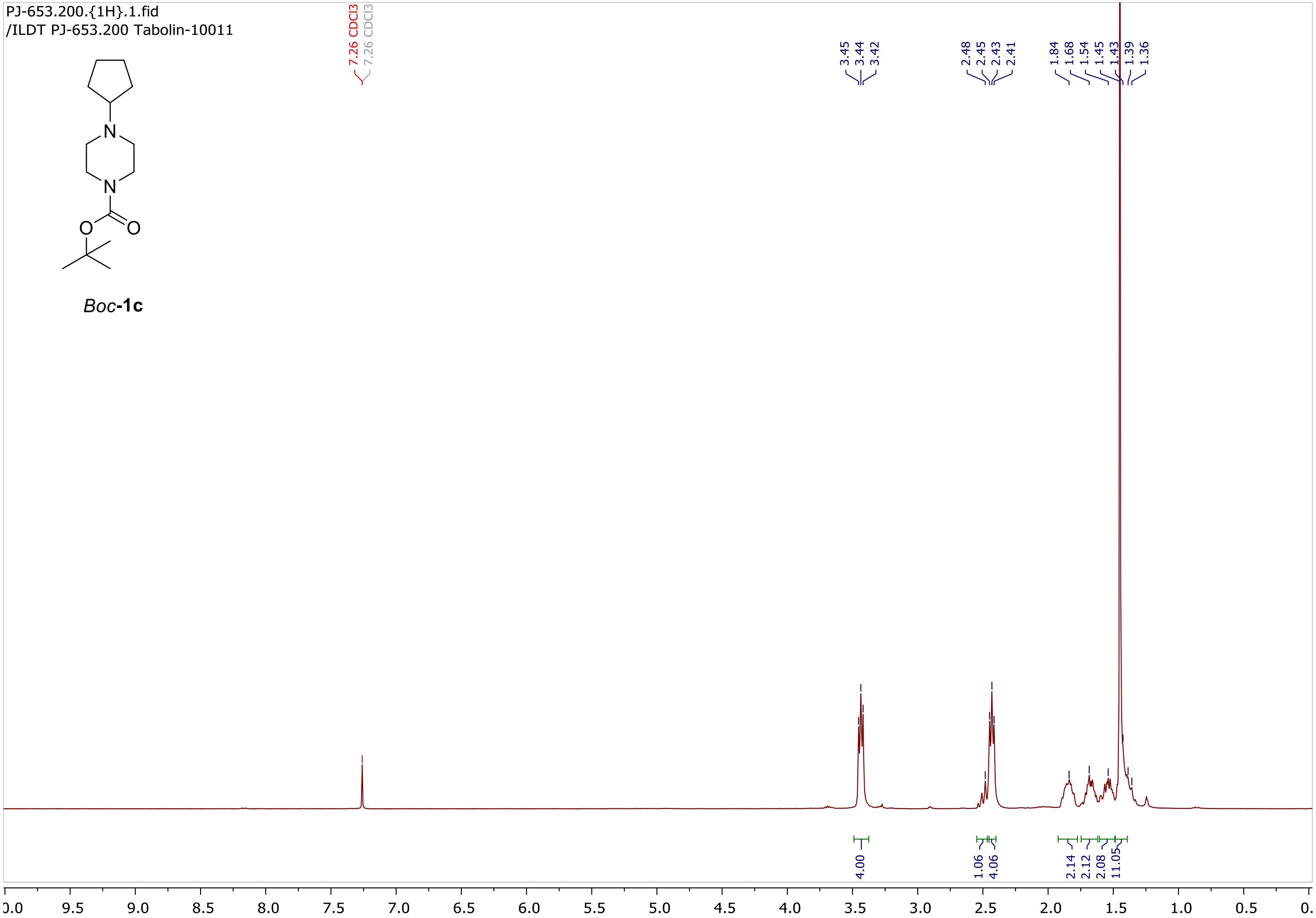

Boc-**1c**

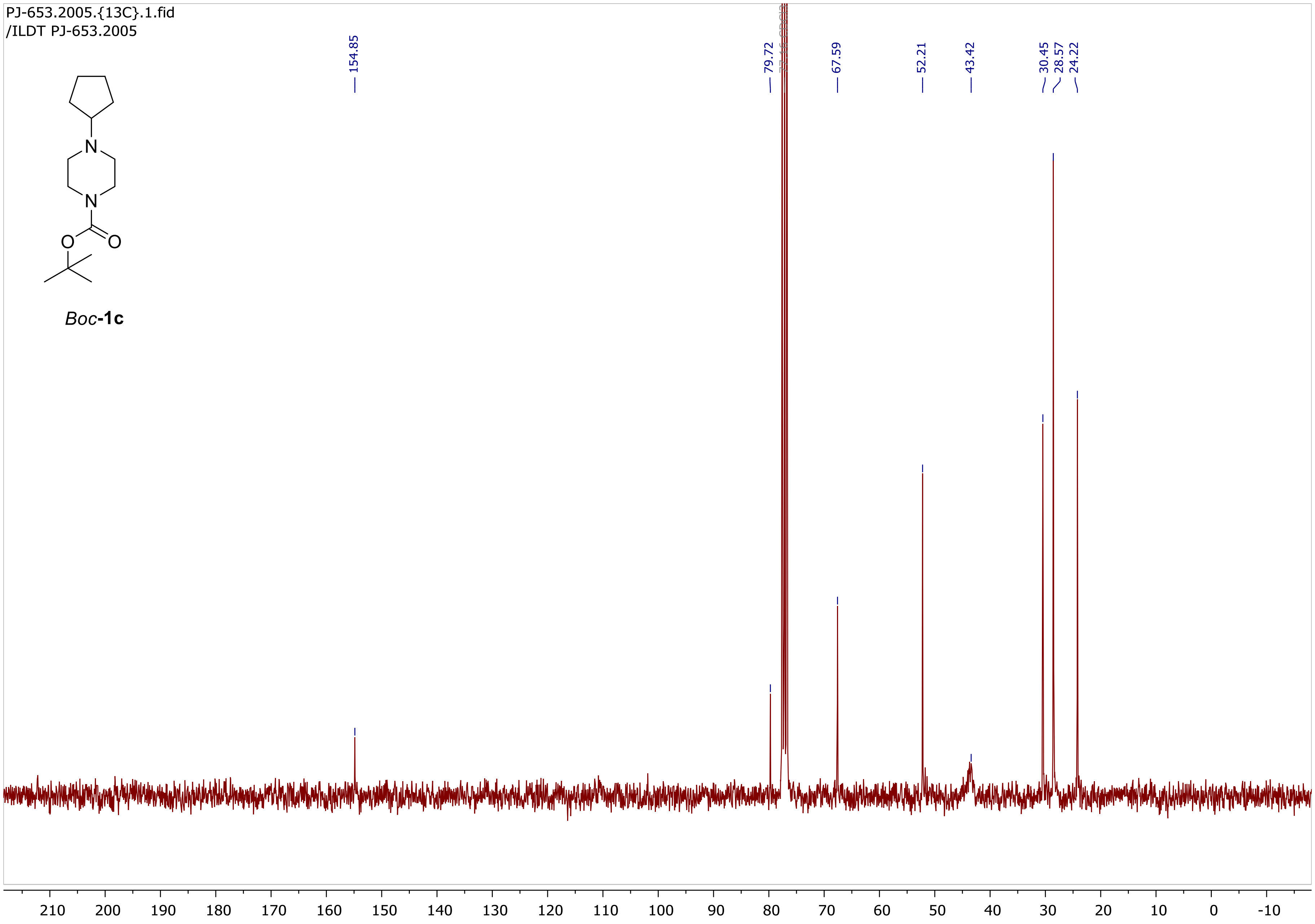

PJ-653.2005.{13C}.1.fid
/ILDT PJ-653.2005
154.85
79.72
67.59
52.21
43.42
30.45
28.57
24.22
N
N
O
O
Boc-**1c**
210
200
190
180
170
160
150
140
130
120
110
100
90
80
70
60
50
40
30
20
10
0
-10

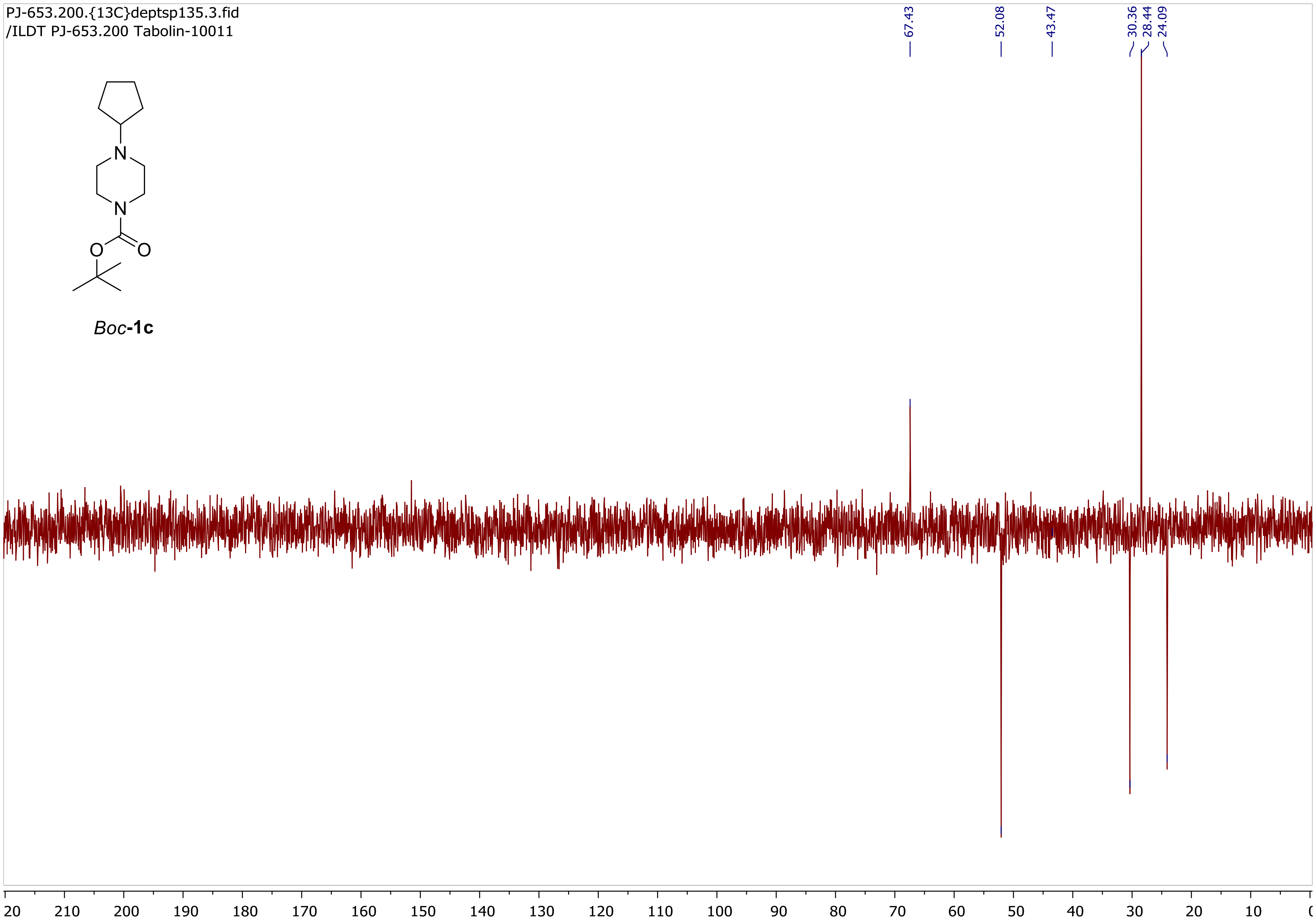

PJ-653.200.{13C}deptsp135.3.fid
/ILDT PJ-653.200 Tabolin-10011
67.43
52.08
43.47
30.36
28.44
24.09
N
N
O
O
Boc-**1c**
20
210
200
190
180
170
160
150
140
130
120
110
100
90
80
70
60
50
40
30
20
10

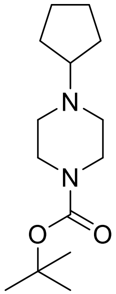

Boc-**1c**

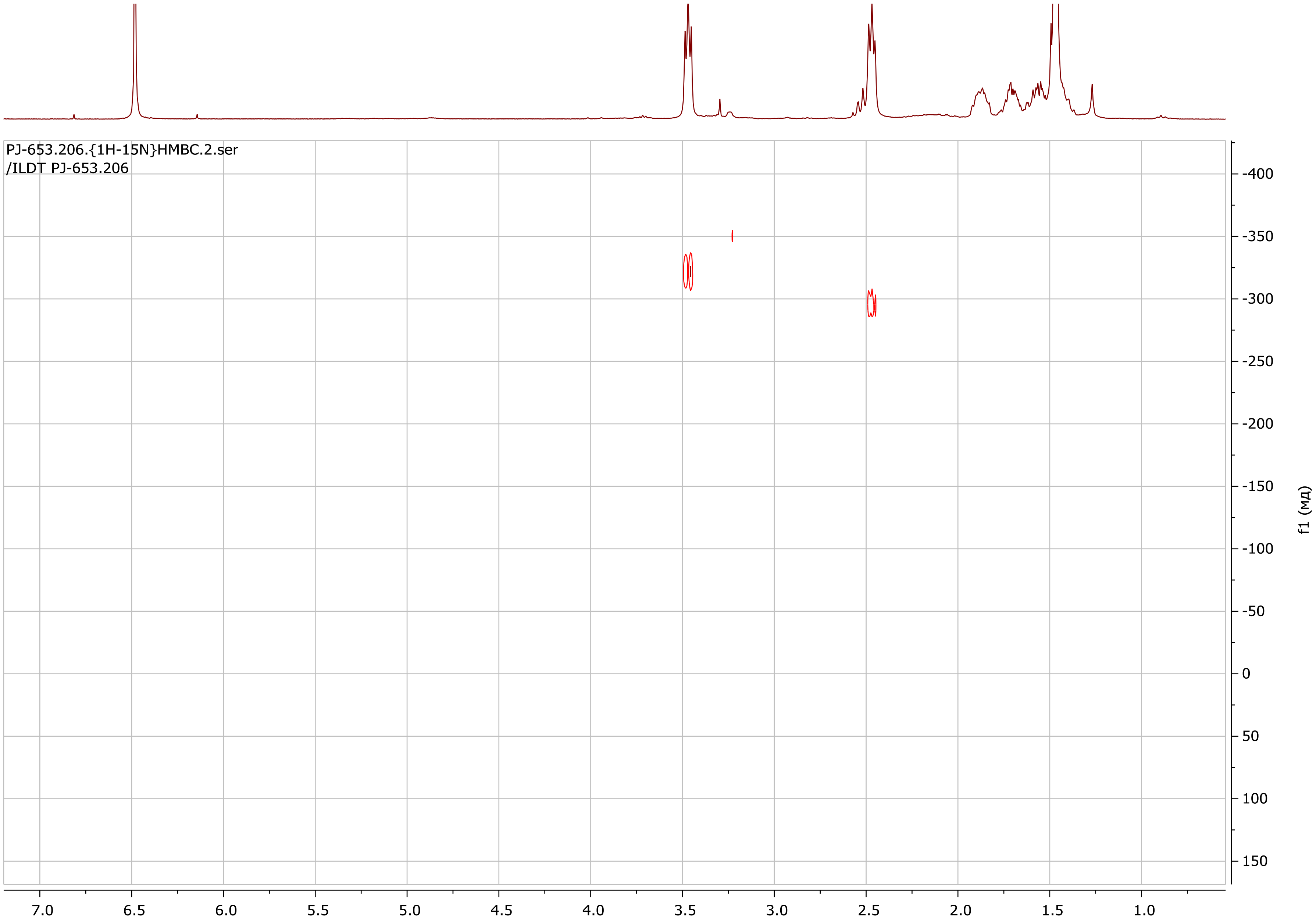

PJ-653.206.{1H-15N}HMBC.2.ser
/ILDT PJ-653.206
-400
-350
-300
-250
-200
-150
-100
-50
0
50
100
150
f1 (мд)
7.0
6.5
6.0
5.5
5.0
4.5
4.0
3.5
3.0
2.5
2.0
1.5
1.0

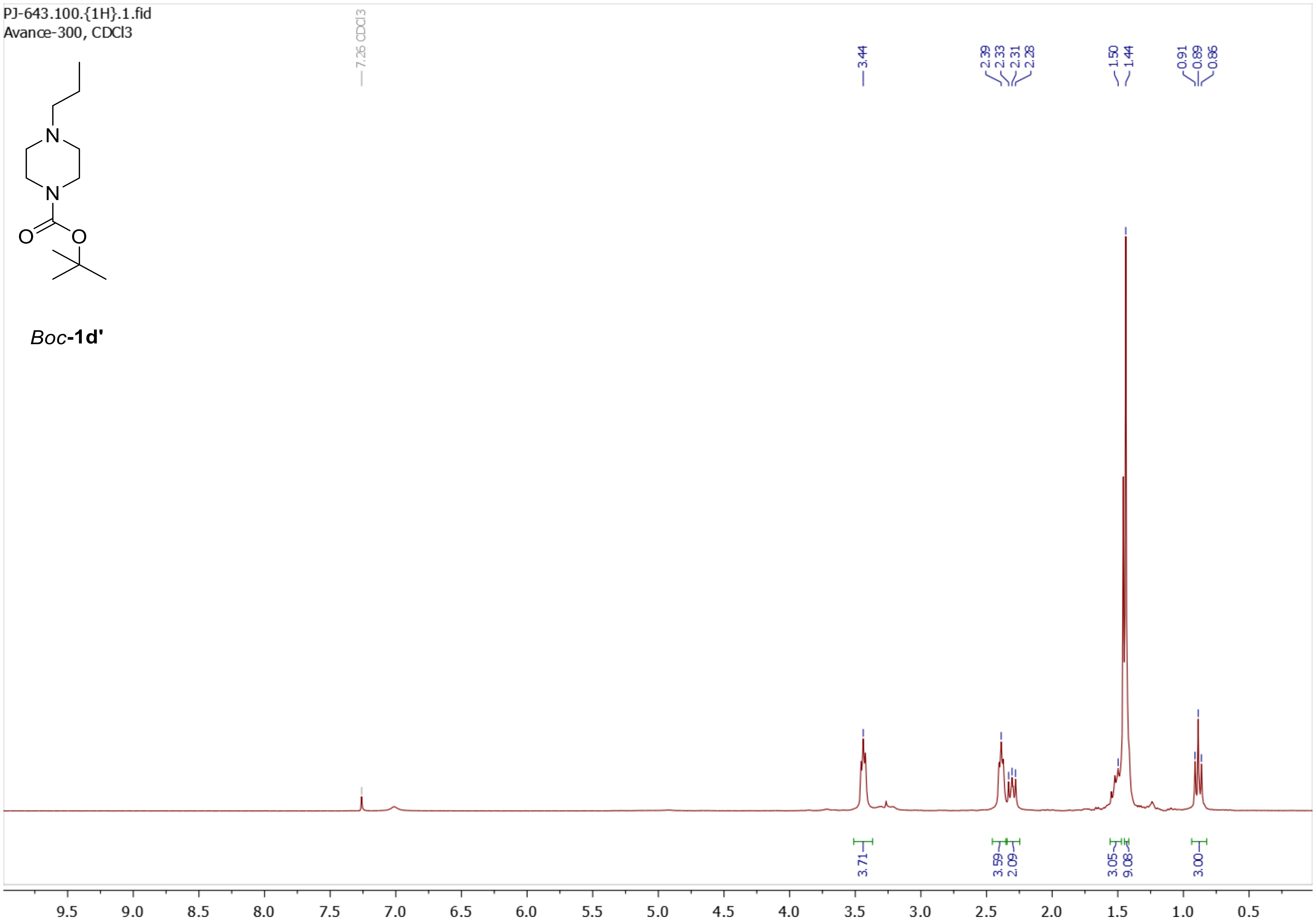
PJ-643.100.{1H}.1.fid
Avance-300, CDCl3
Boc-1d'
7.26 CDCl3
3.44
2.39
2.33
2.31
2.28
1.50
1.44
0.91
0.89
0.86
3.71
3.59
2.09
3.05
9.08
3.00
9.5 9.0 8.5 8.0 7.5 7.0 6.5 6.0 5.5 5.0 4.5 4.0 3.5 3.0 2.5 2.0 1.5 1.0 0.5

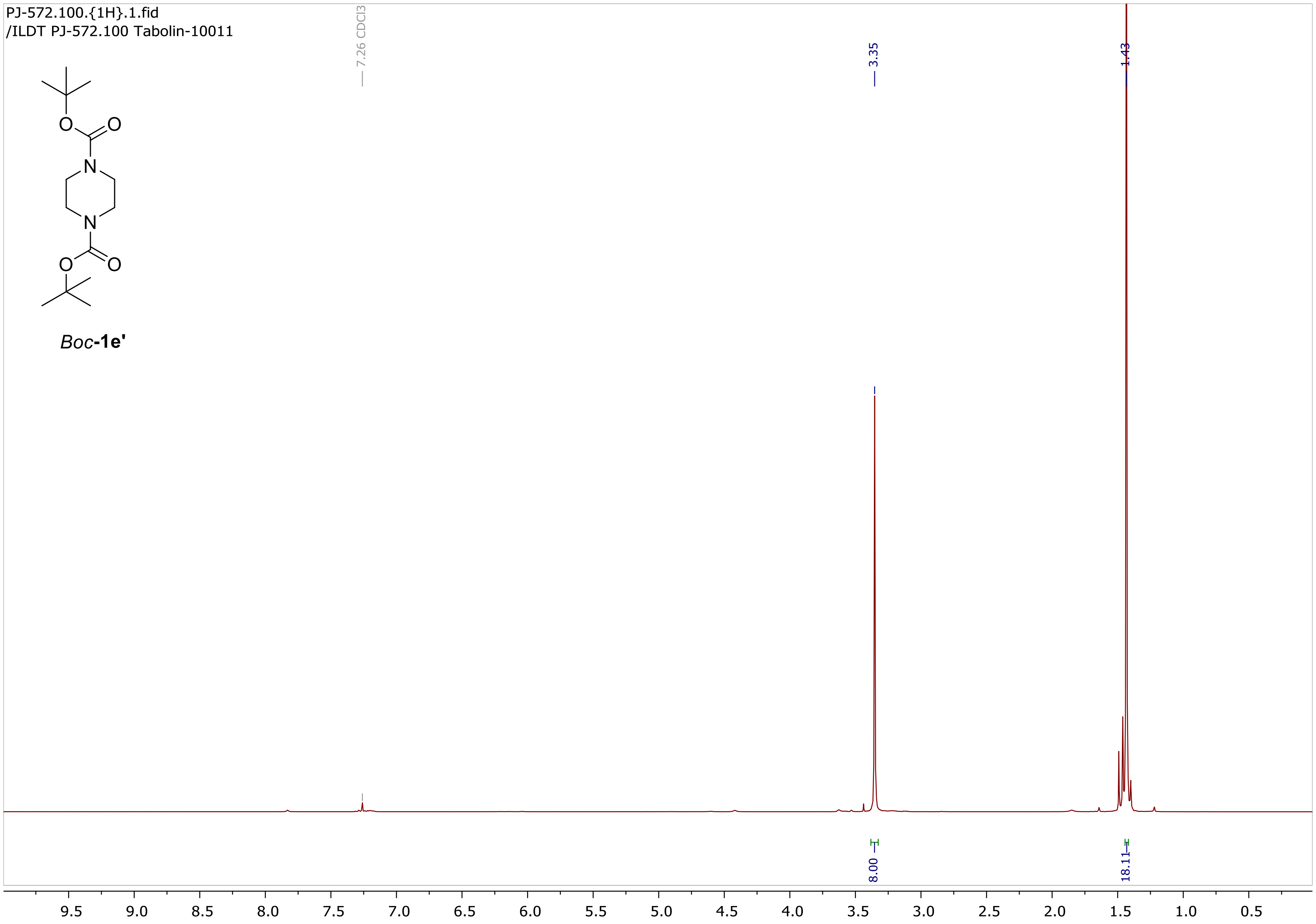
PJ-572.100.{1H}.1.fid
/ILDT PJ-572.100 Tabolin-10011
7.26 CDCl3
3.35
1.43
Boc-**1e'**
8.00
18.11
9.5
9.0
8.5
8.0
7.5
7.0
6.5
6.0
5.5
5.0
4.5
4.0
3.5
3.0
2.5
2.0
1.5
1.0
0.5

PJ-572.100.{13C}.2.fid
/ILDT PJ-572.100 Tabolin-10011

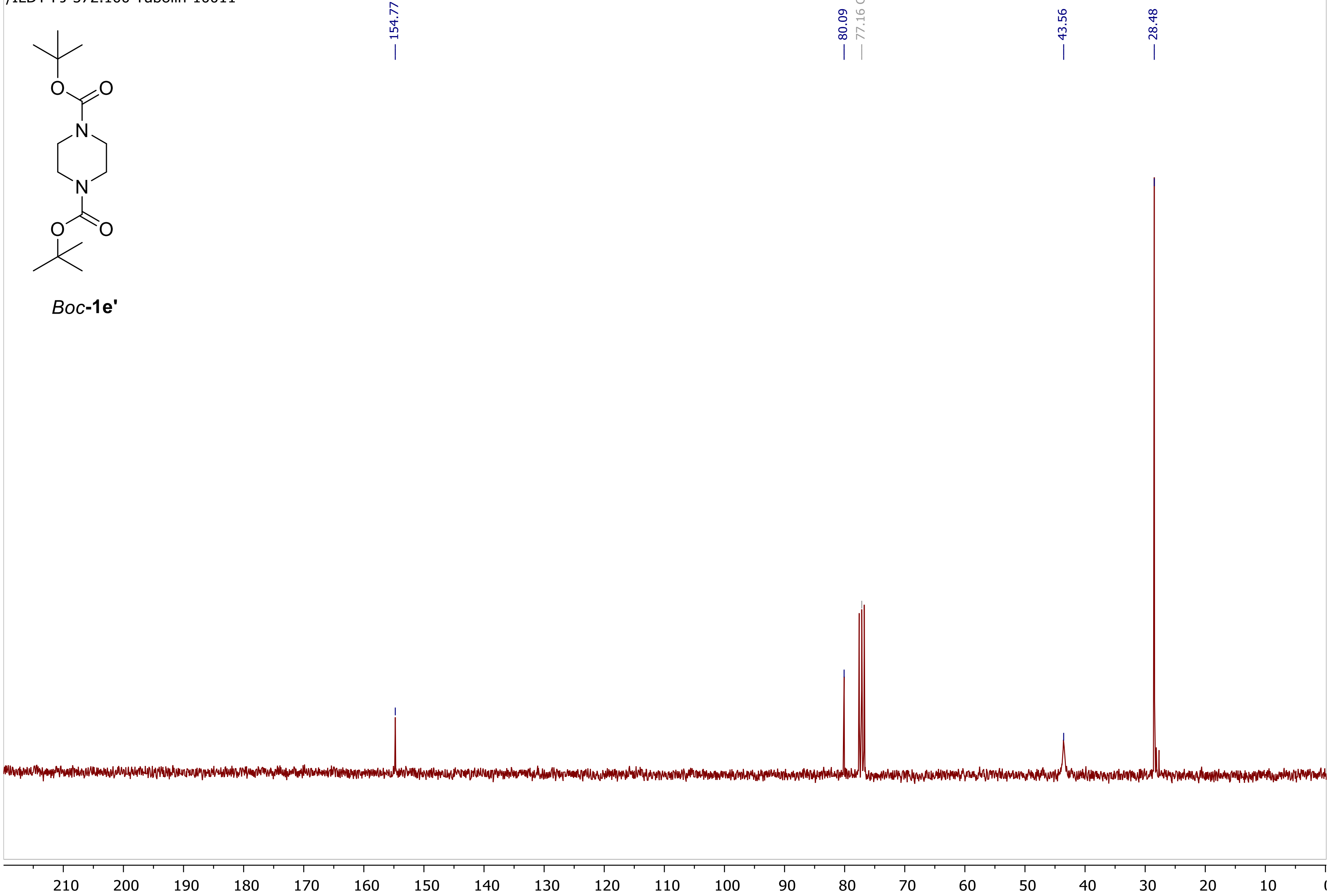

PJ-572.100.{13C}deptsp135.3.fid
/ILDT PJ-572.100 Tabolin-10011

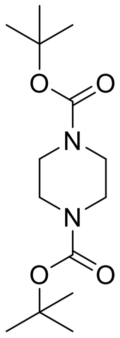

Boc-**1e'**

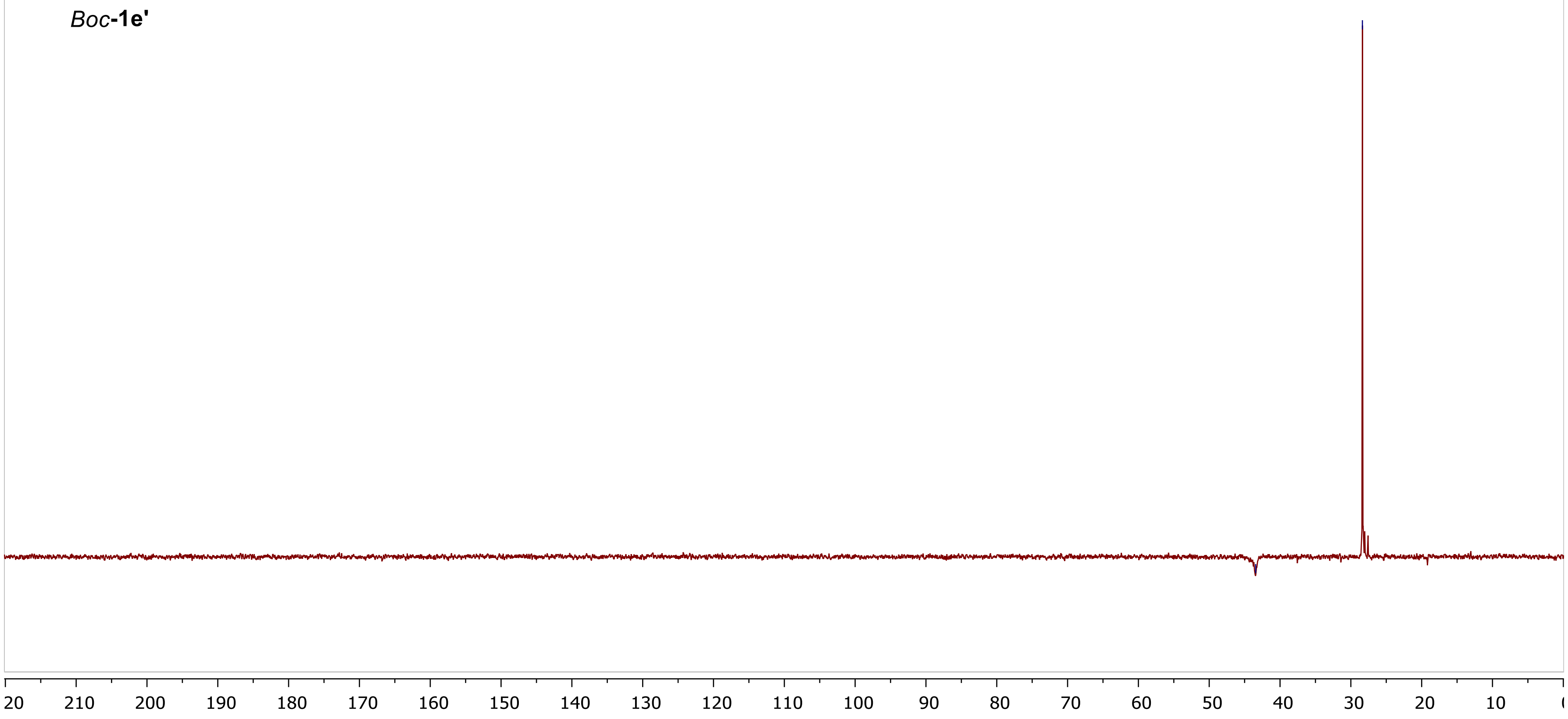

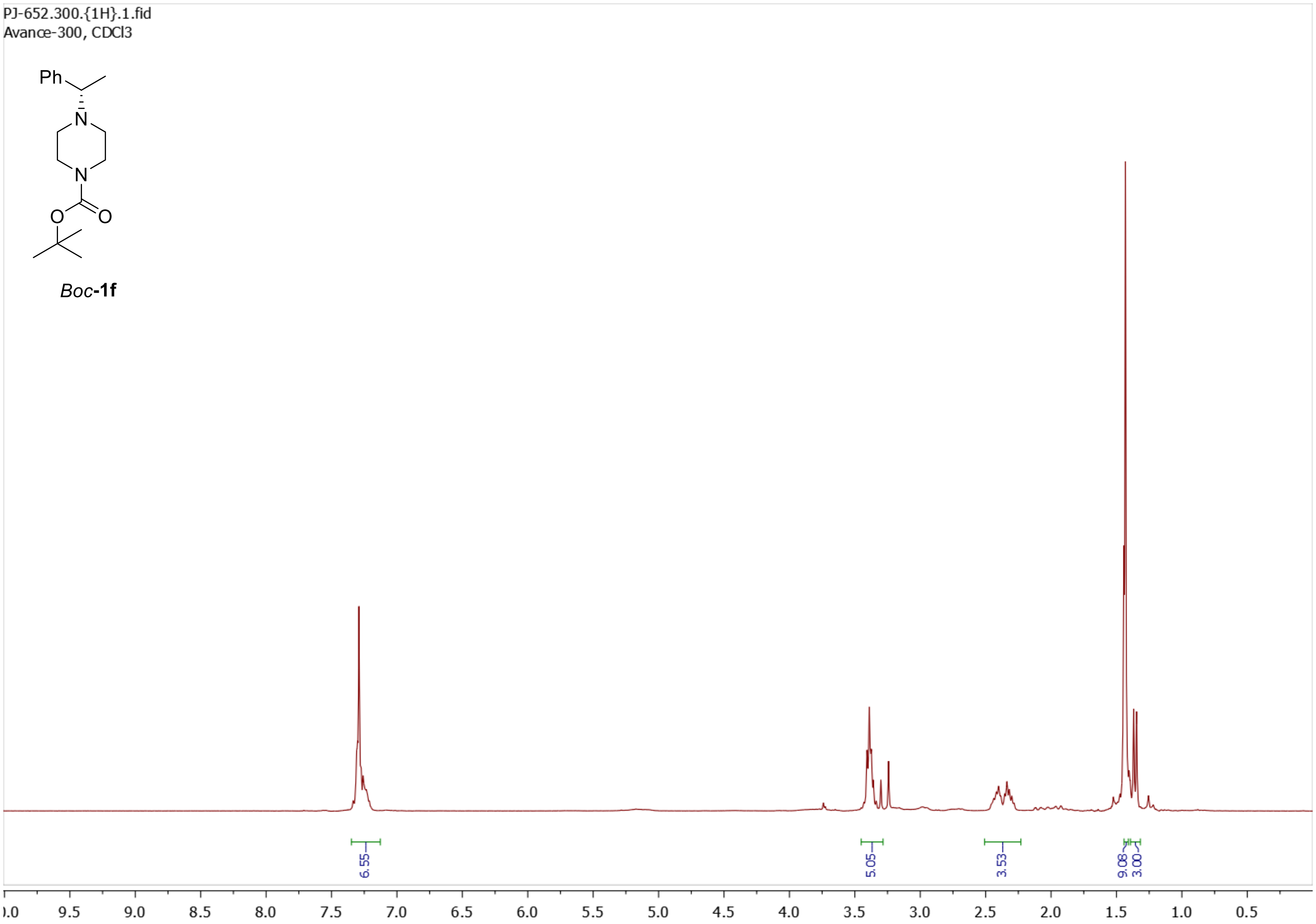
PJ-652.300.{1H}.1.fid
Avance-300, CDCl3
Ph
N
N
O
O
Boc-1f
6.55
5.05
3.53
9.08
3.00
0.0
9.5
9.0
8.5
8.0
7.5
7.0
6.5
6.0
5.5
5.0
4.5
4.0
3.5
3.0
2.5
2.0
1.5
1.0
0.5

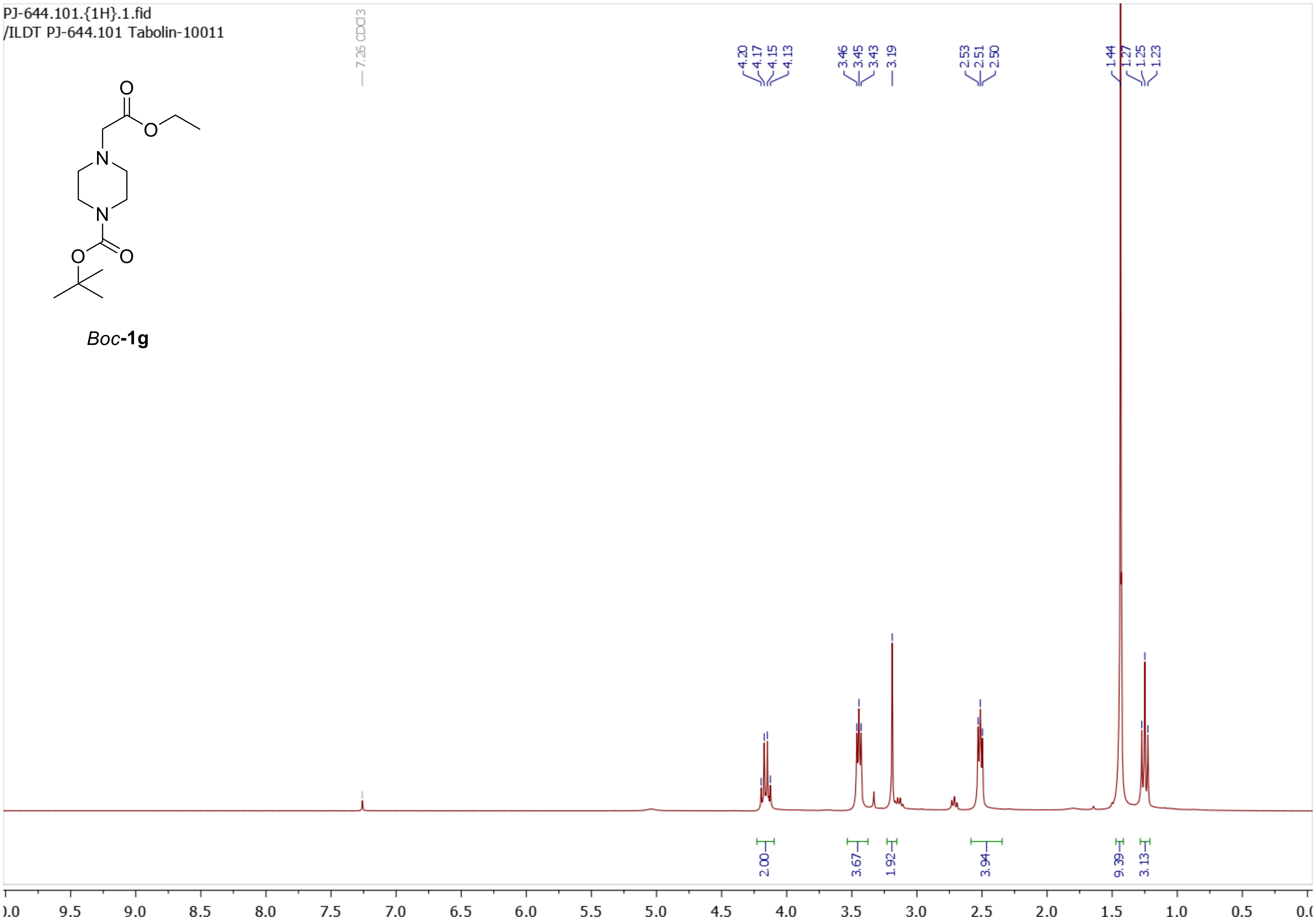
PJ-644.101.{1H}.1.fid
/ILDT PJ-644.101 Tabolin-10011
7.26 CDCl3
4.20
4.17
4.15
4.13
3.46
3.45
3.43
3.19
2.53
2.51
2.50
1.44
1.27
1.25
1.23
Boc-1g
2.00
3.67
1.92
3.94
9.39
3.13
9.5
9.0
8.5
8.0
7.5
7.0
6.5
6.0
5.5
5.0
4.5
4.0
3.5
3.0
2.5
2.0
1.5
1.0
0.5

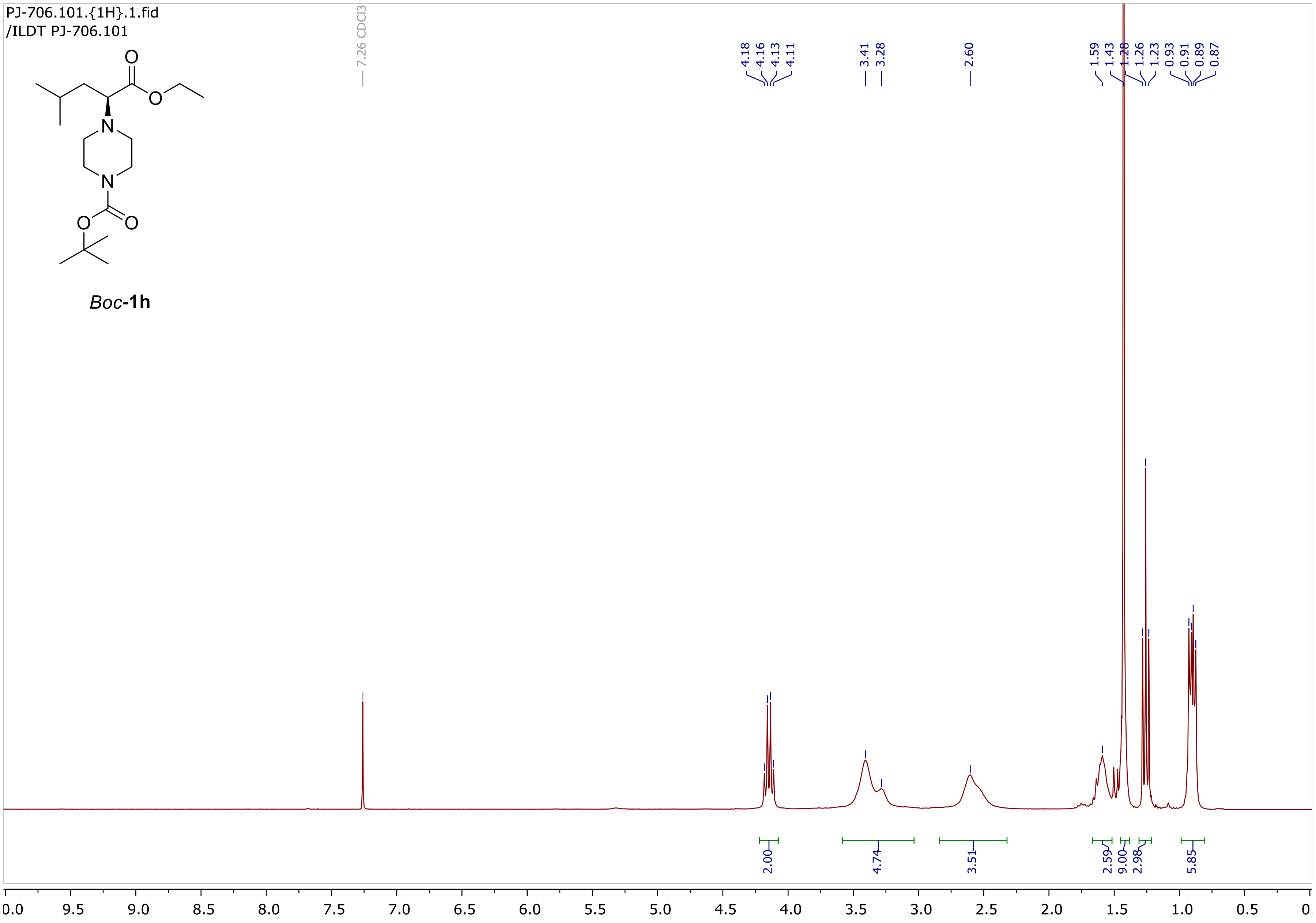
PJ-706.101.{1H}.1.fid
/ILDT PJ-706.101
Boc-1h
7.26 CDCl3
4.18
4.16
4.13
4.11
3.41
3.28
2.60
1.59
1.43
1.28
1.26
1.23
0.93
0.91
0.89
0.87
2.00
4.74
3.51
2.59
9.00
2.98
5.85
10.0
9.5
9.0
8.5
8.0
7.5
7.0
6.5
6.0
5.5
5.0
4.5
4.0
3.5
3.0
2.5
2.0
1.5
1.0
0.5
0.

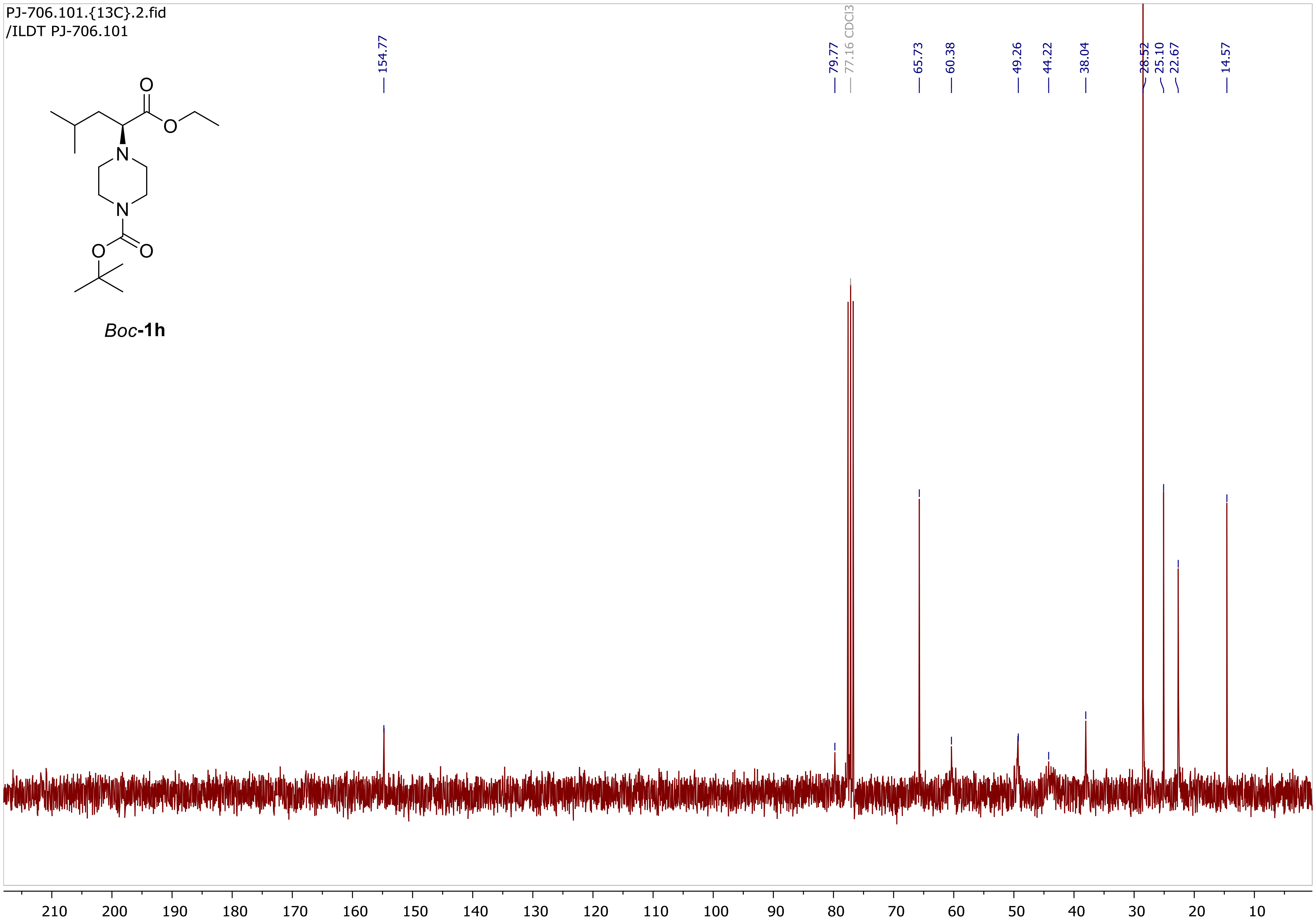
PJ-706.101.{13C}.2.fid
/ILDT PJ-706.101
154.77
79.77
77.16 CDCl3
65.73
60.38
49.26
44.22
38.04
28.52
25.10
22.67
14.57
Boc-1h
210
200
190
180
170
160
150
140
130
120
110
100
90
80
70
60
50
40
30
20
10

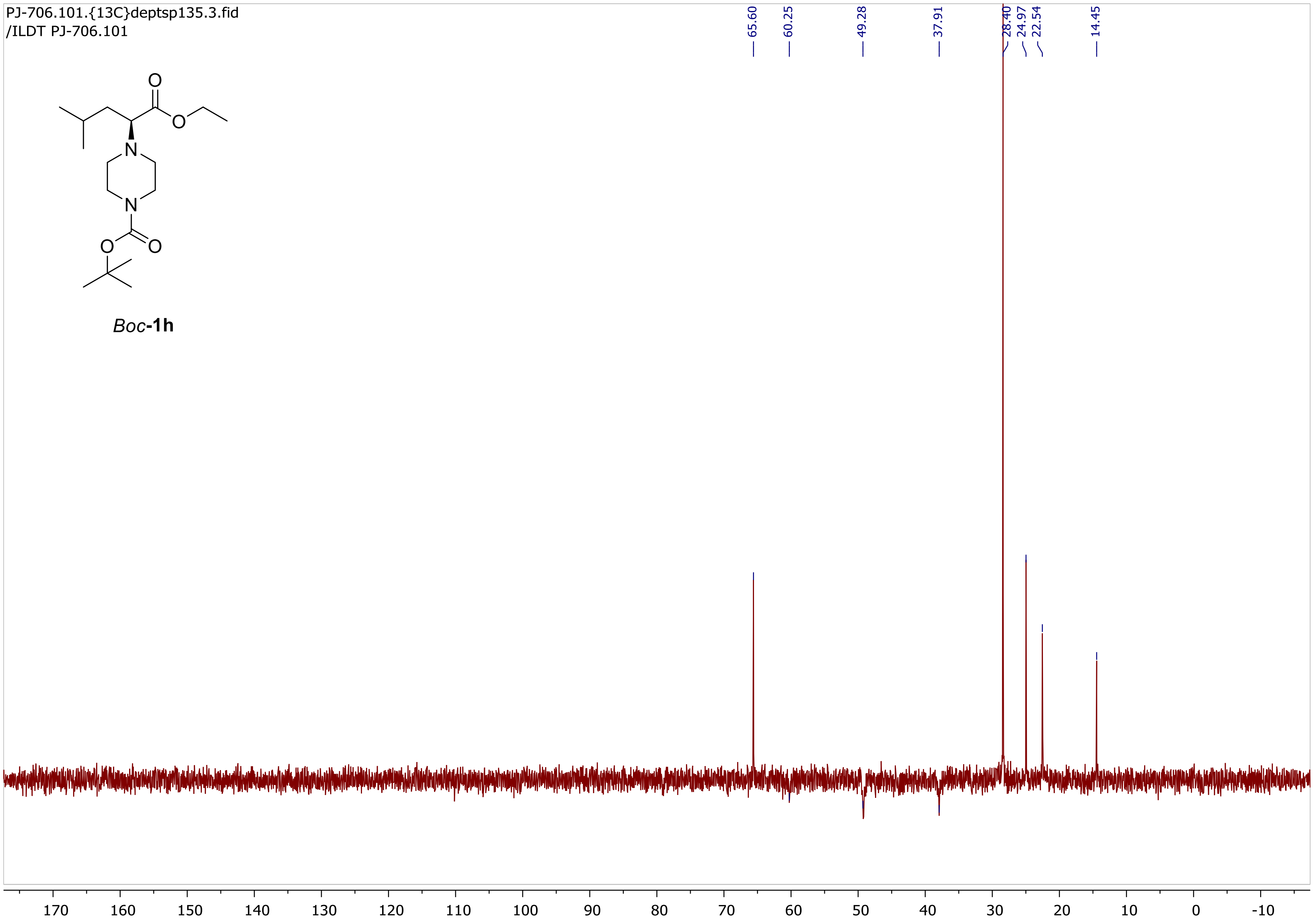
PJ-706.101.{13C}deptsp135.3.fid
/ILDT PJ-706.101
65.60
60.25
49.28
37.91
28.40
24.97
22.54
14.45
170
160
150
140
130
120
110
100
90
80
70
60
50
40
30
20
10
0
-10

Boc-**1h**

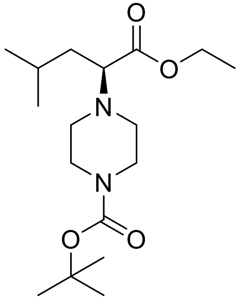

Boc-**1h**

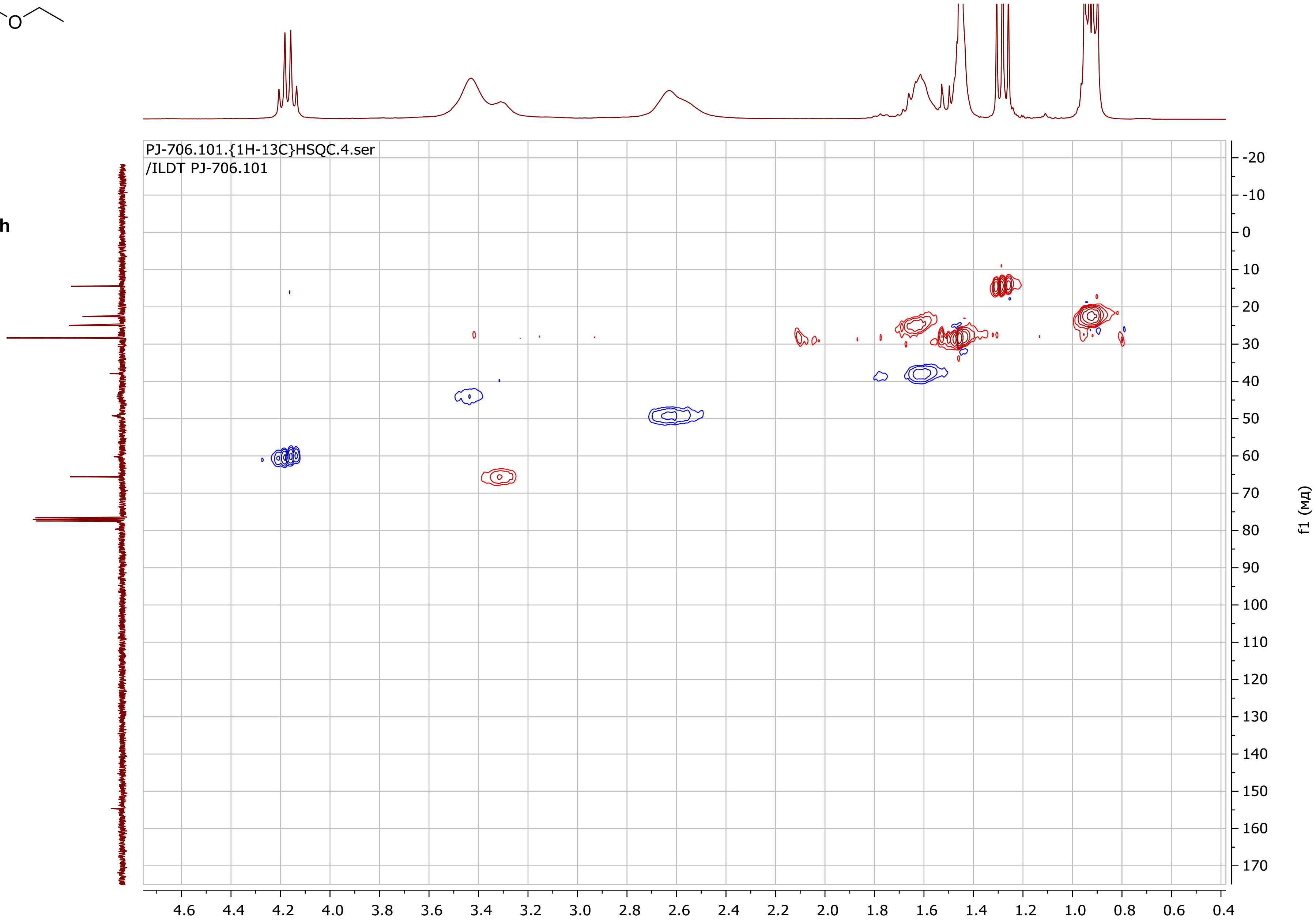

PJ-661.100.{1H}.1.fid
/ILDT PJ-661.100 Tabolin-10011

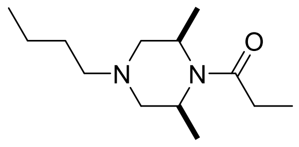

2,6-*cis*-*EtCO*-**1i**

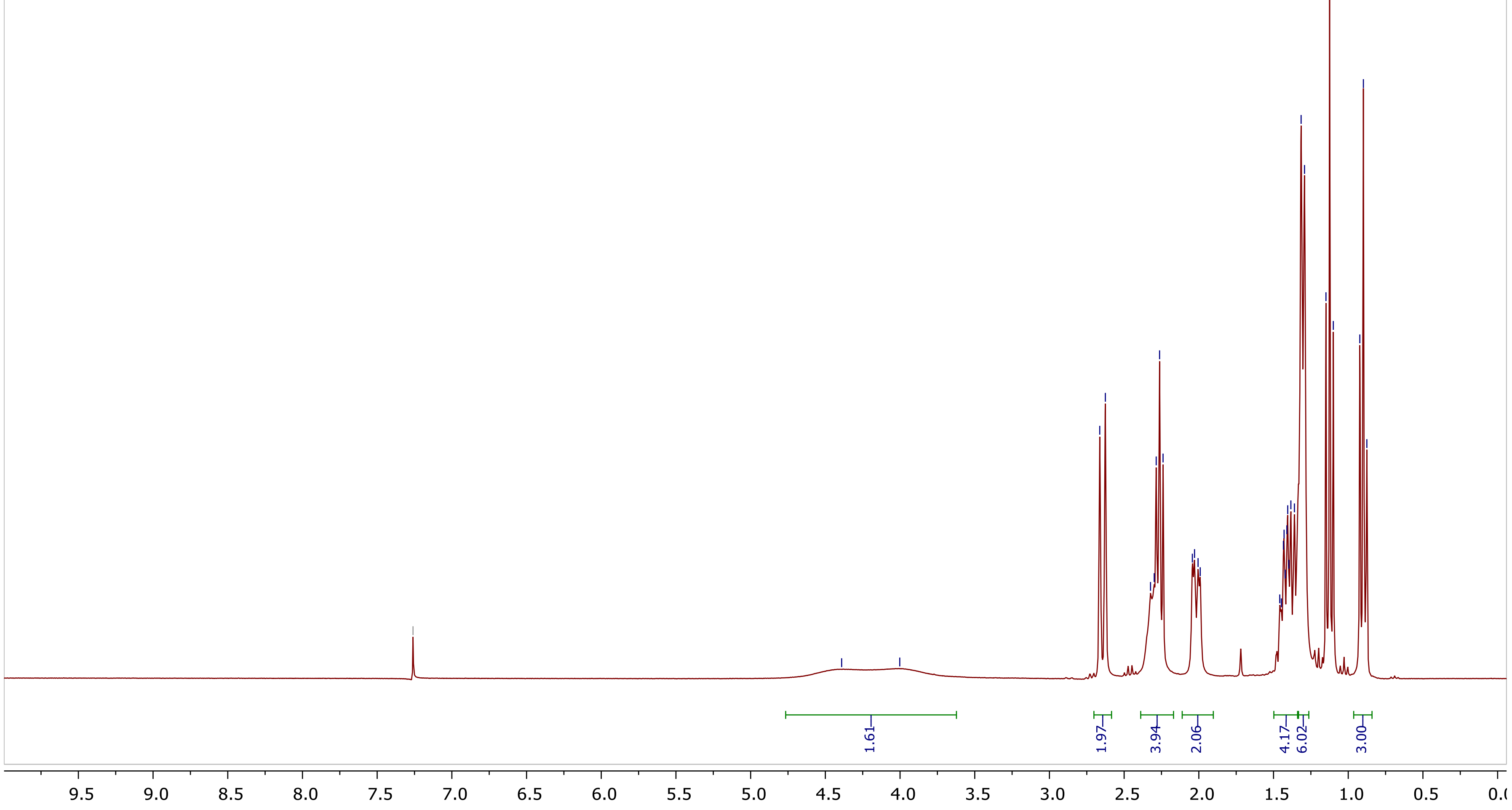

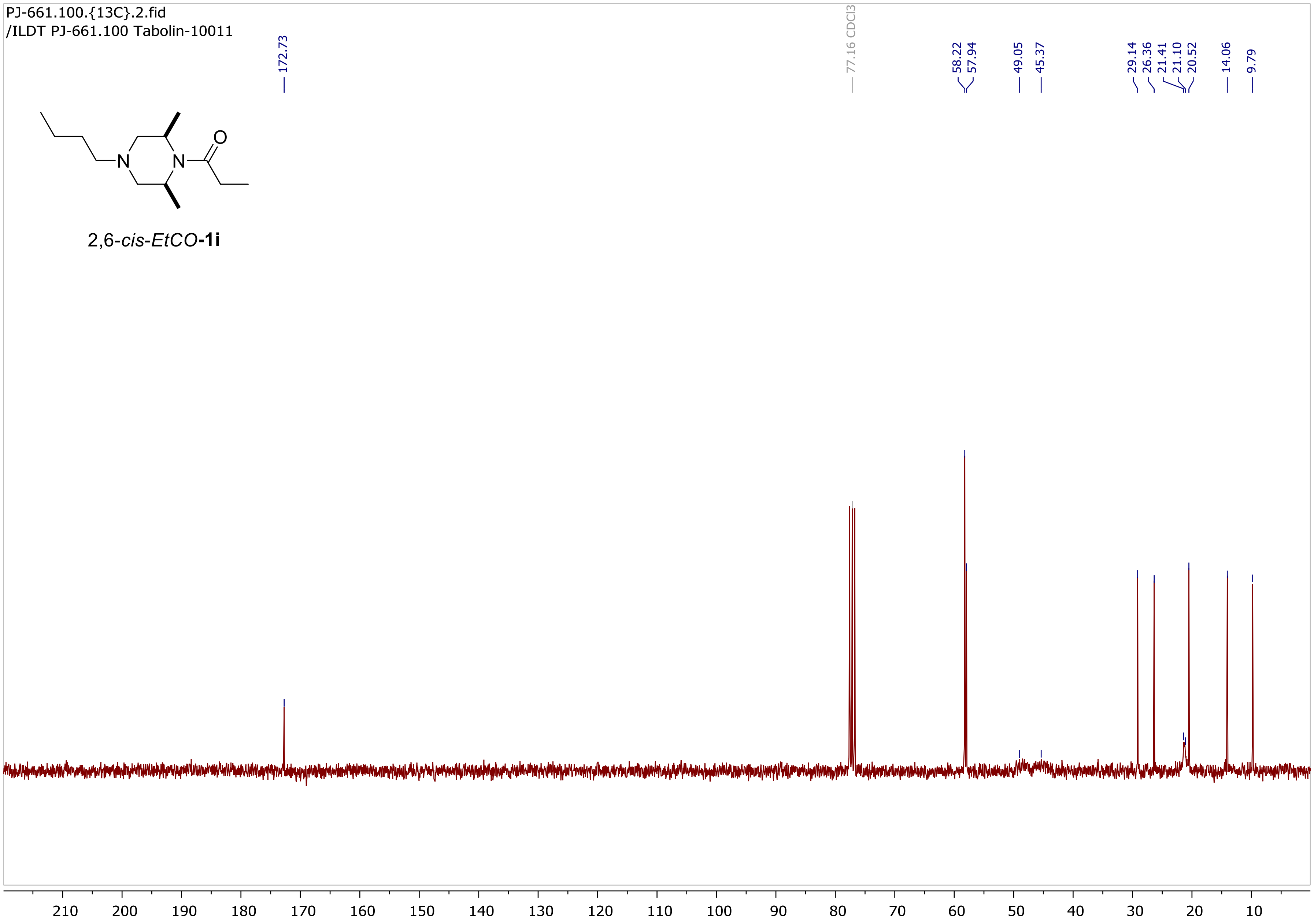
PJ-661.100.{13C}.2.fid
/ILDT PJ-661.100 Tabolin-10011
172.73
77.16 CDCl3
58.22
57.94
49.05
45.37
29.14
26.36
21.41
21.10
20.52
14.06
9.79
2,6-cis-EtCO-1i
210 200 190 180 170 160 150 140 130 120 110 100 90 80 70 60 50 40 30 20 10

PJ-661.100.{13C}deptsp135.6.fid
/ILDT PJ-661.100 Tabolin-10011

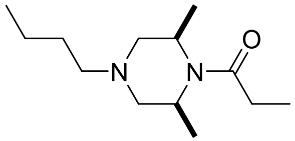

2,6-*cis*-*EtCO*-**1i**

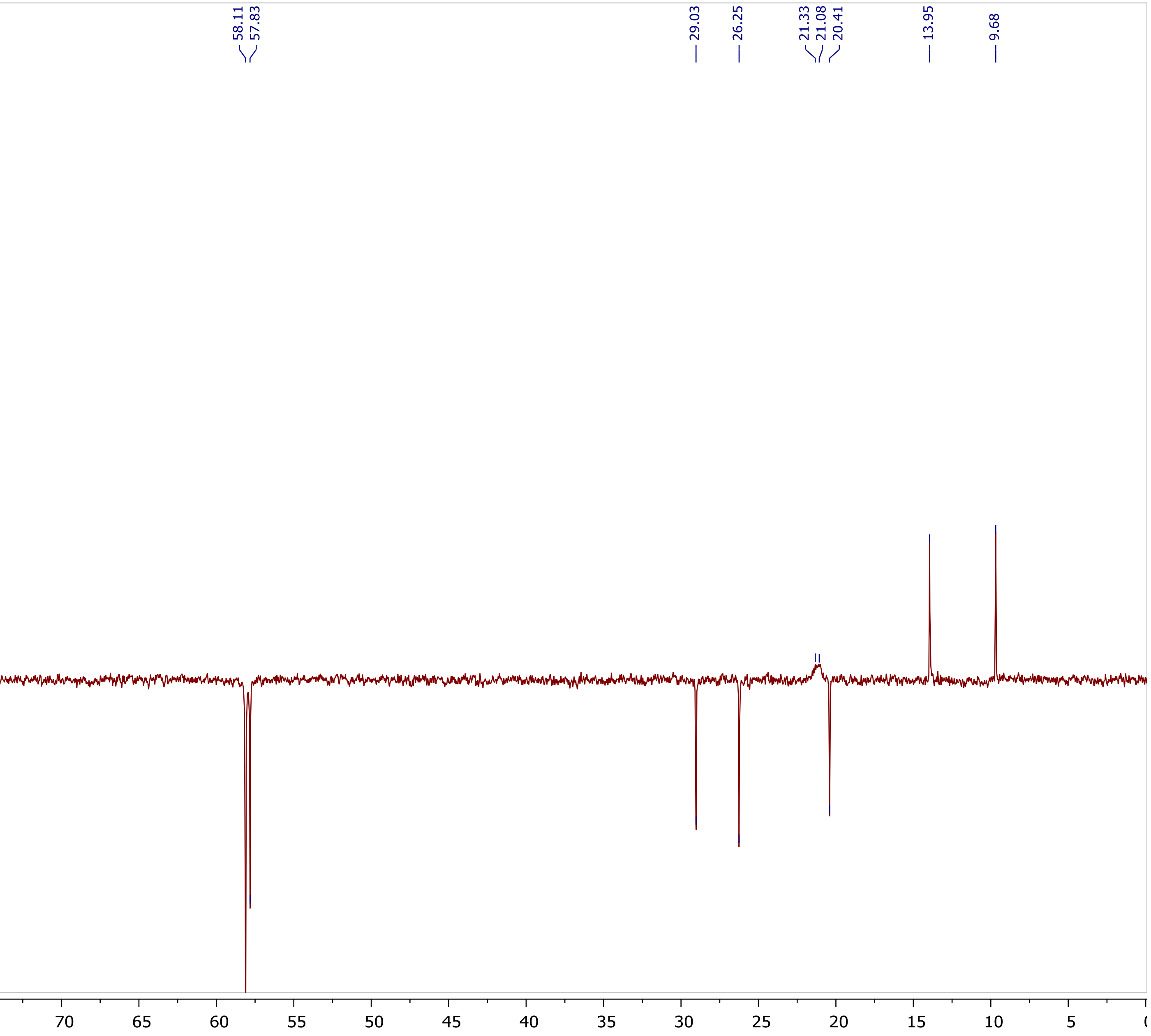

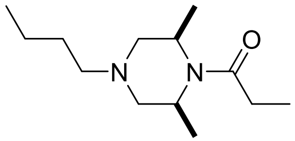

2,6-*cis*-*EtCO*-**1i**

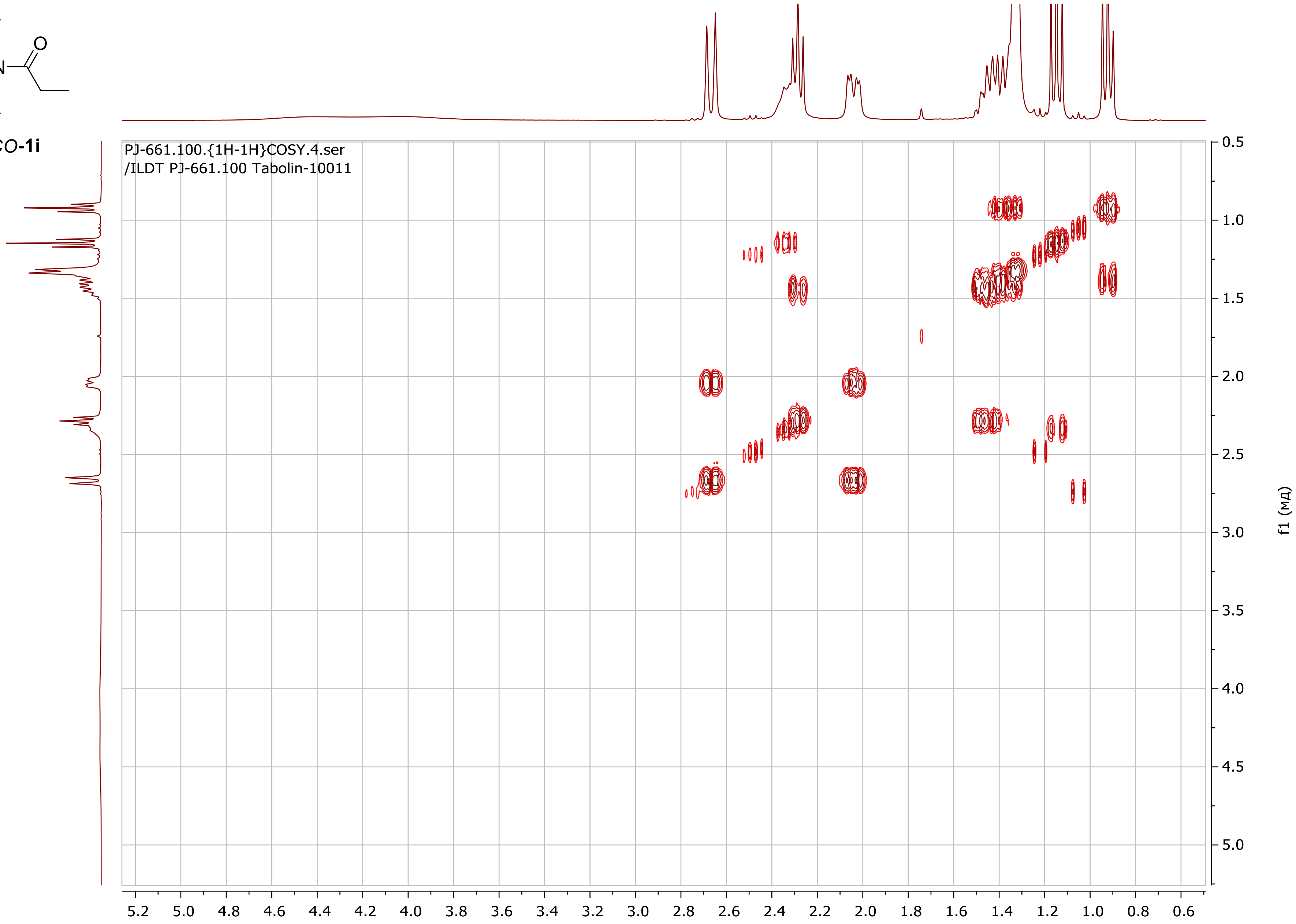

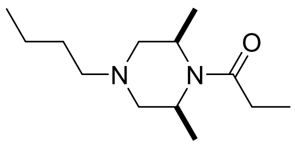

2,6-*cis*-*EtCO*-**1i**

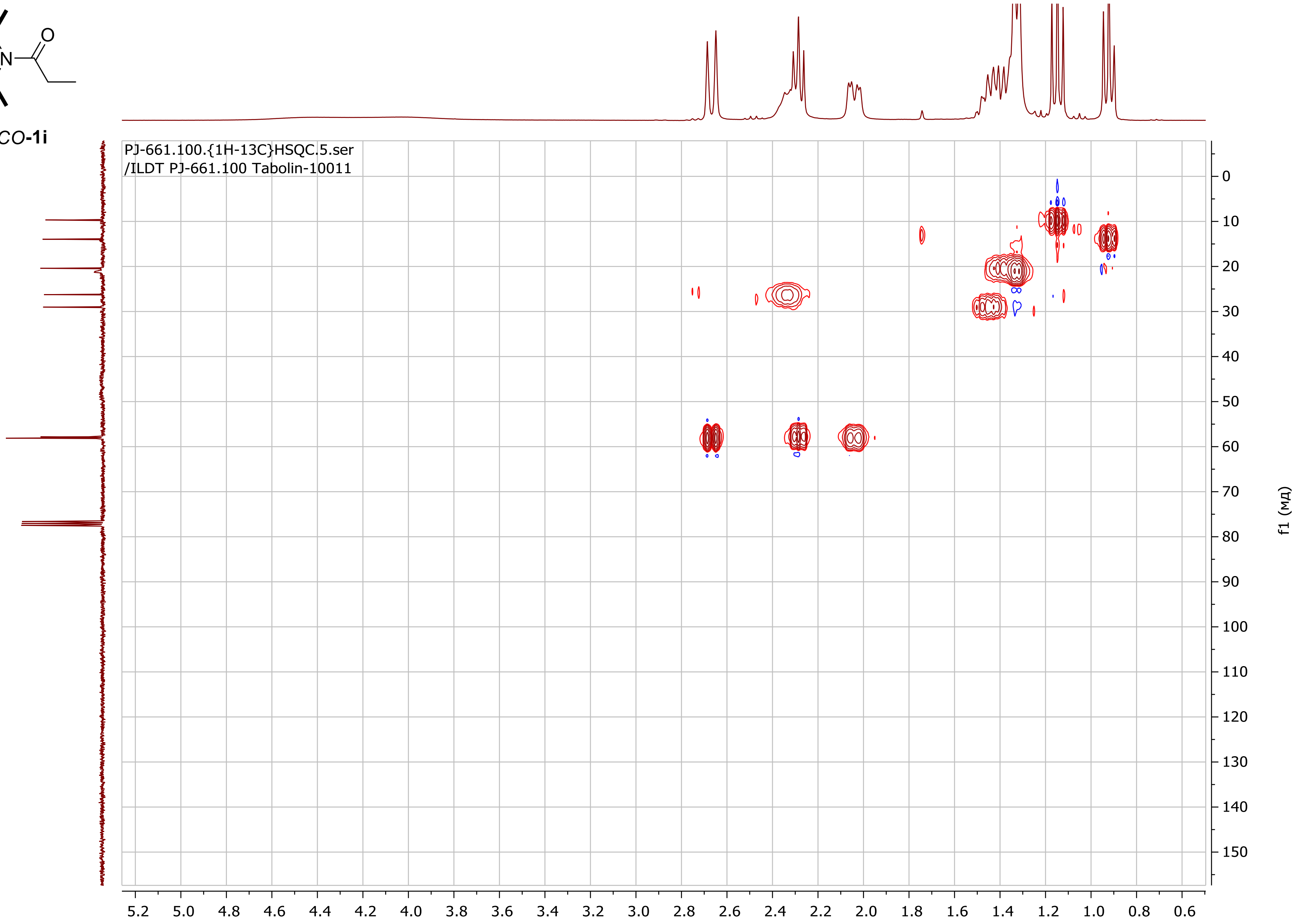

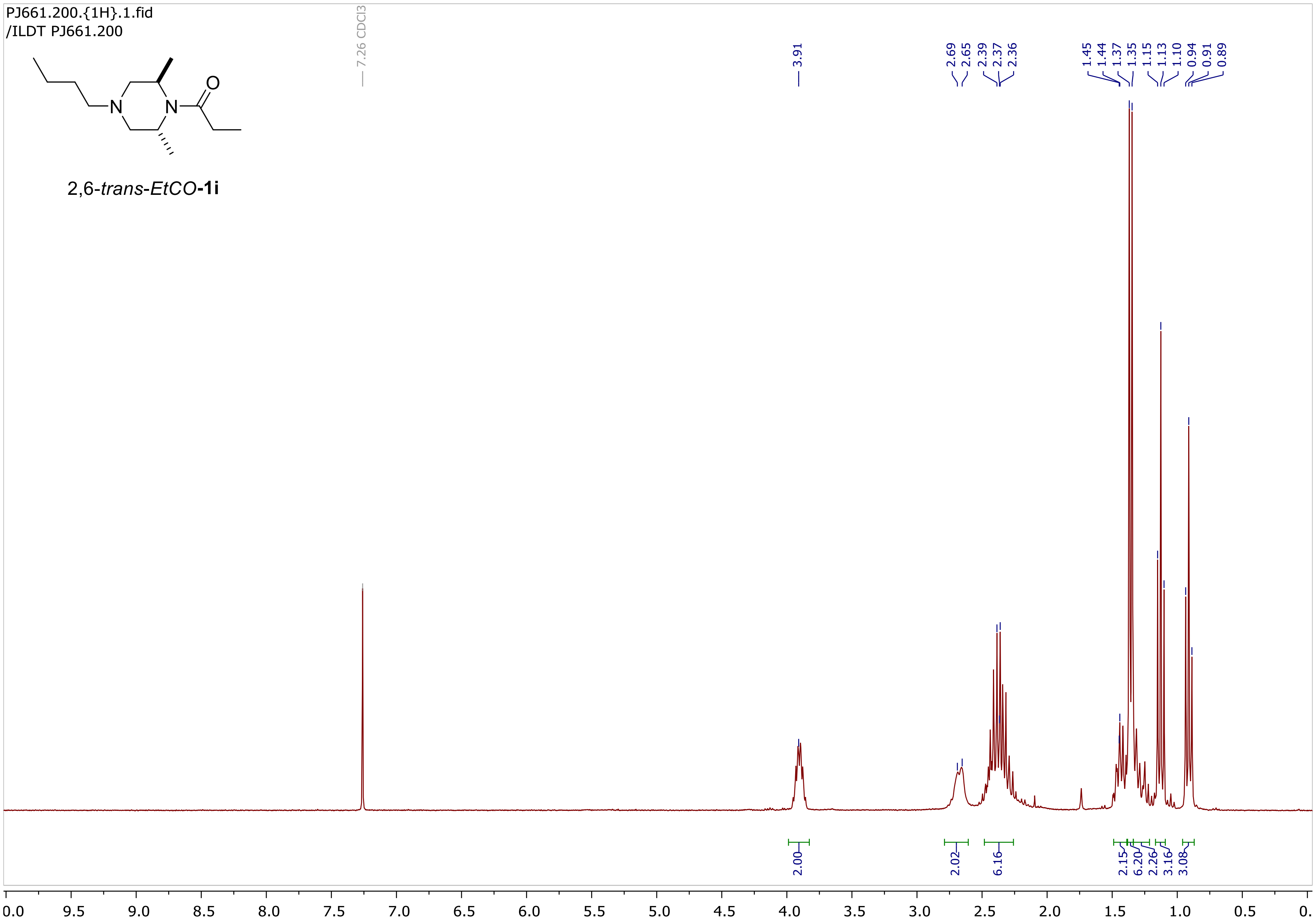

2,6-*trans*-*EtCO*-**1i**

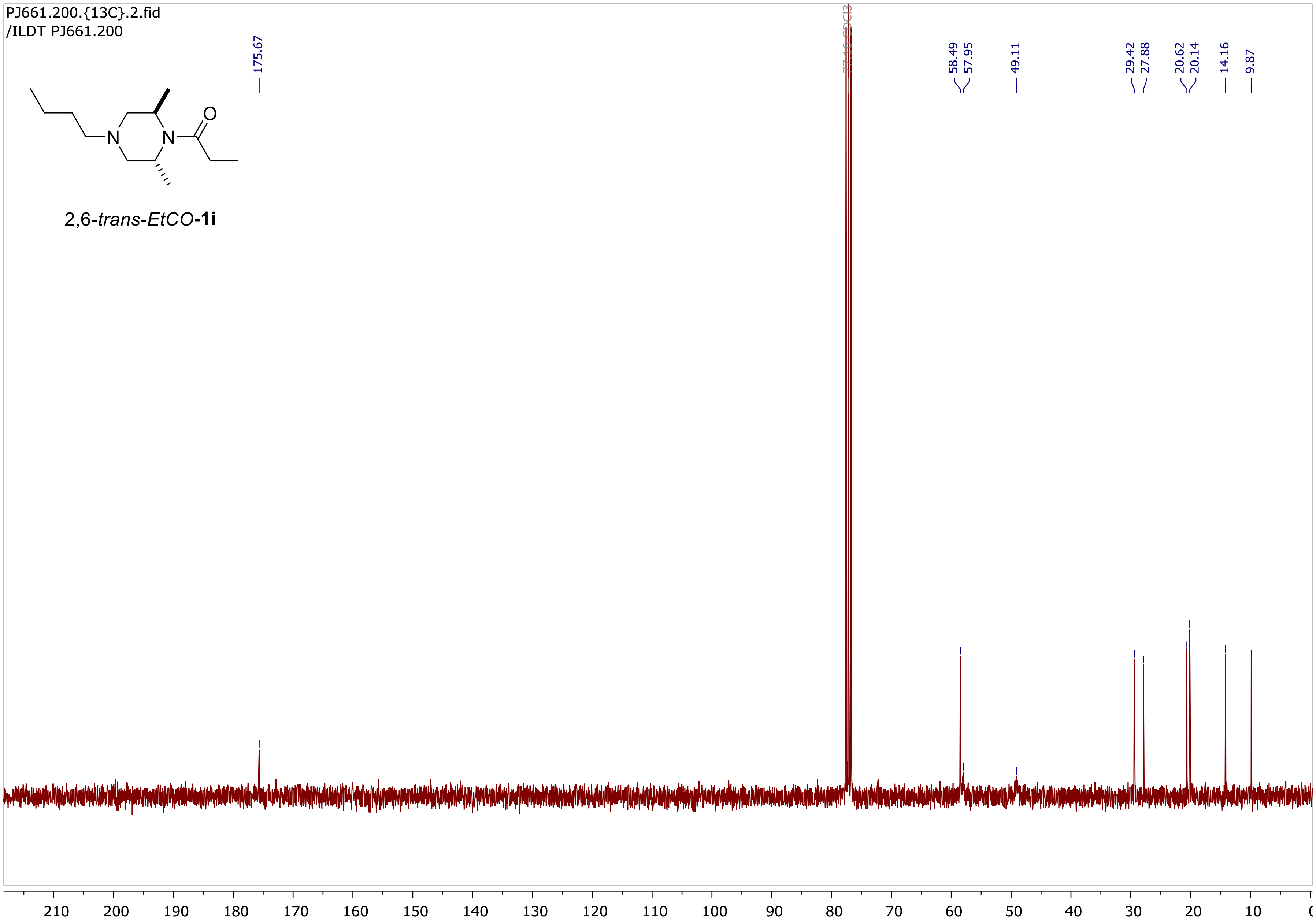
PJ661.200.{13C}.2.fid
/ILDT PJ661.200
2,6-*trans*-*EtCO*-**1i**
175.67
77.16 CDCl3
58.49
57.95
49.11
29.42
27.88
20.62
20.14
14.16
9.87
210 200 190 180 170 160 150 140 130 120 110 100 90 80 70 60 50 40 30 20 10

PJ661.200.{13C}deptsp135.3.fid
/ILDT PJ661.200

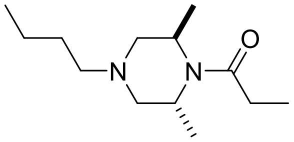

2,6-*trans*-*EtCO*-**1i**

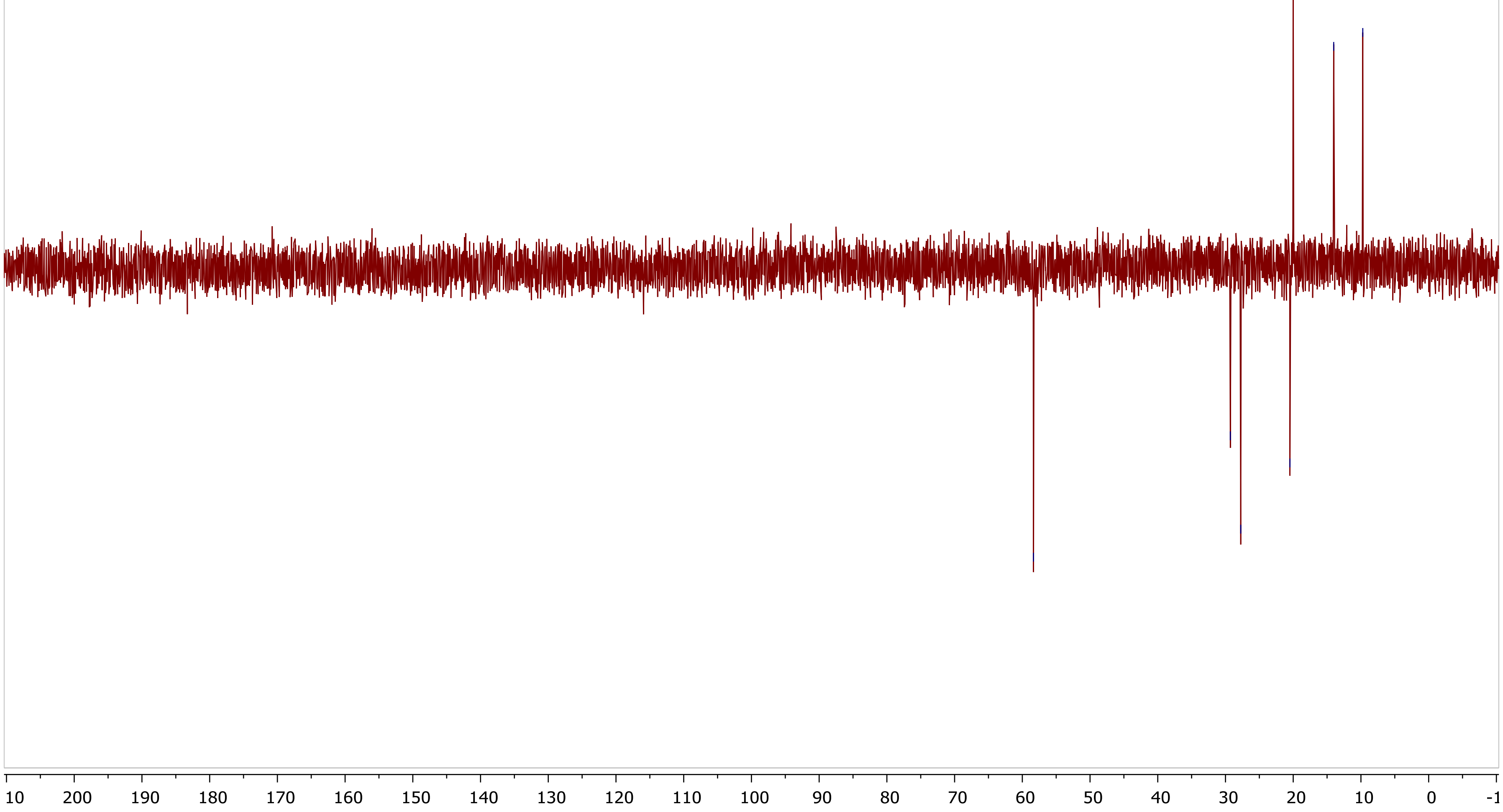

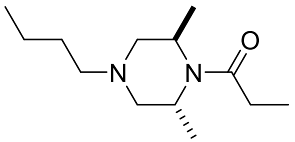

2,6-*trans*-*EtCO*-**1i**

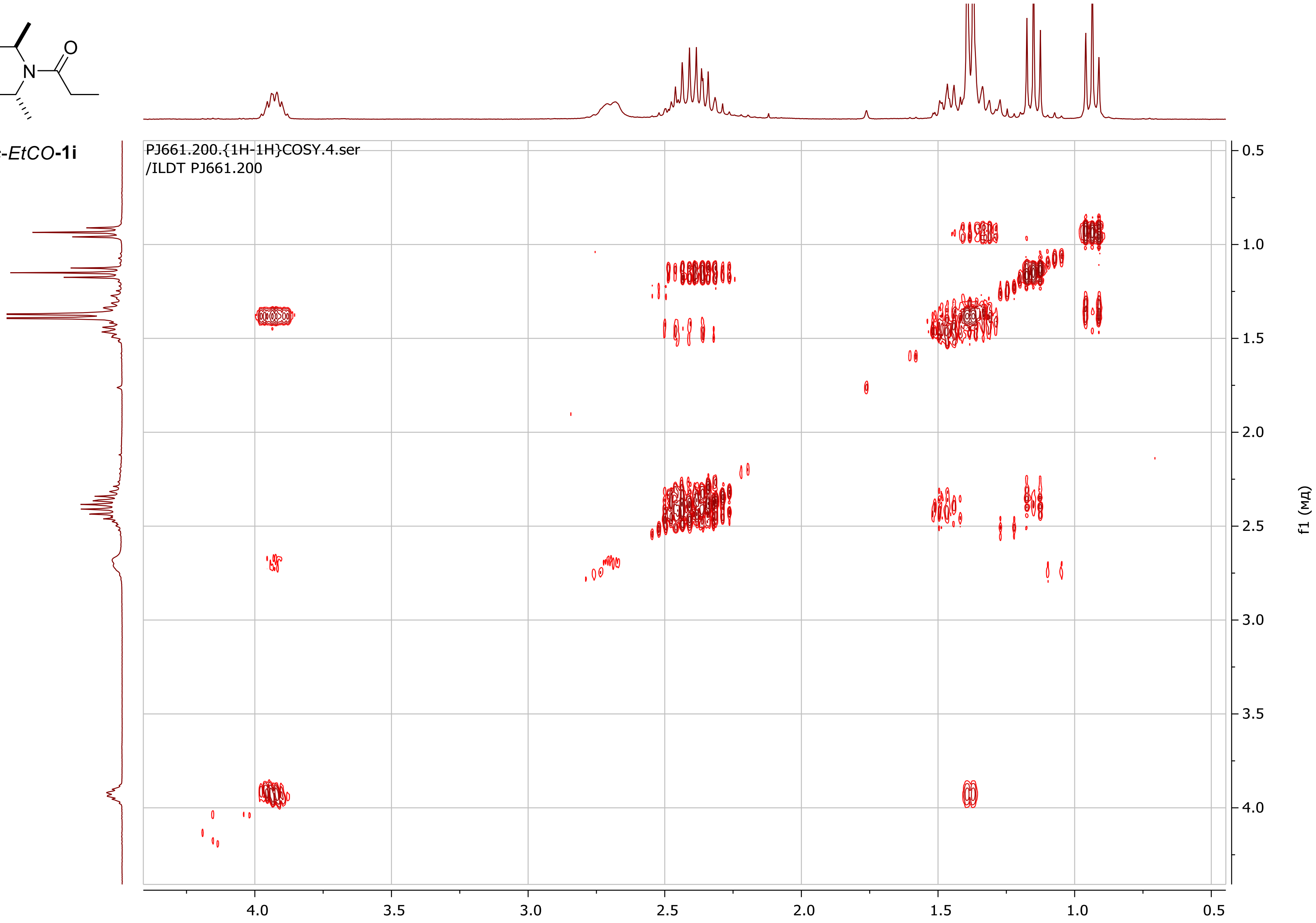

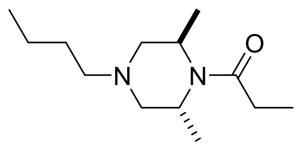

2,6-*trans-EtCO*-**1i**

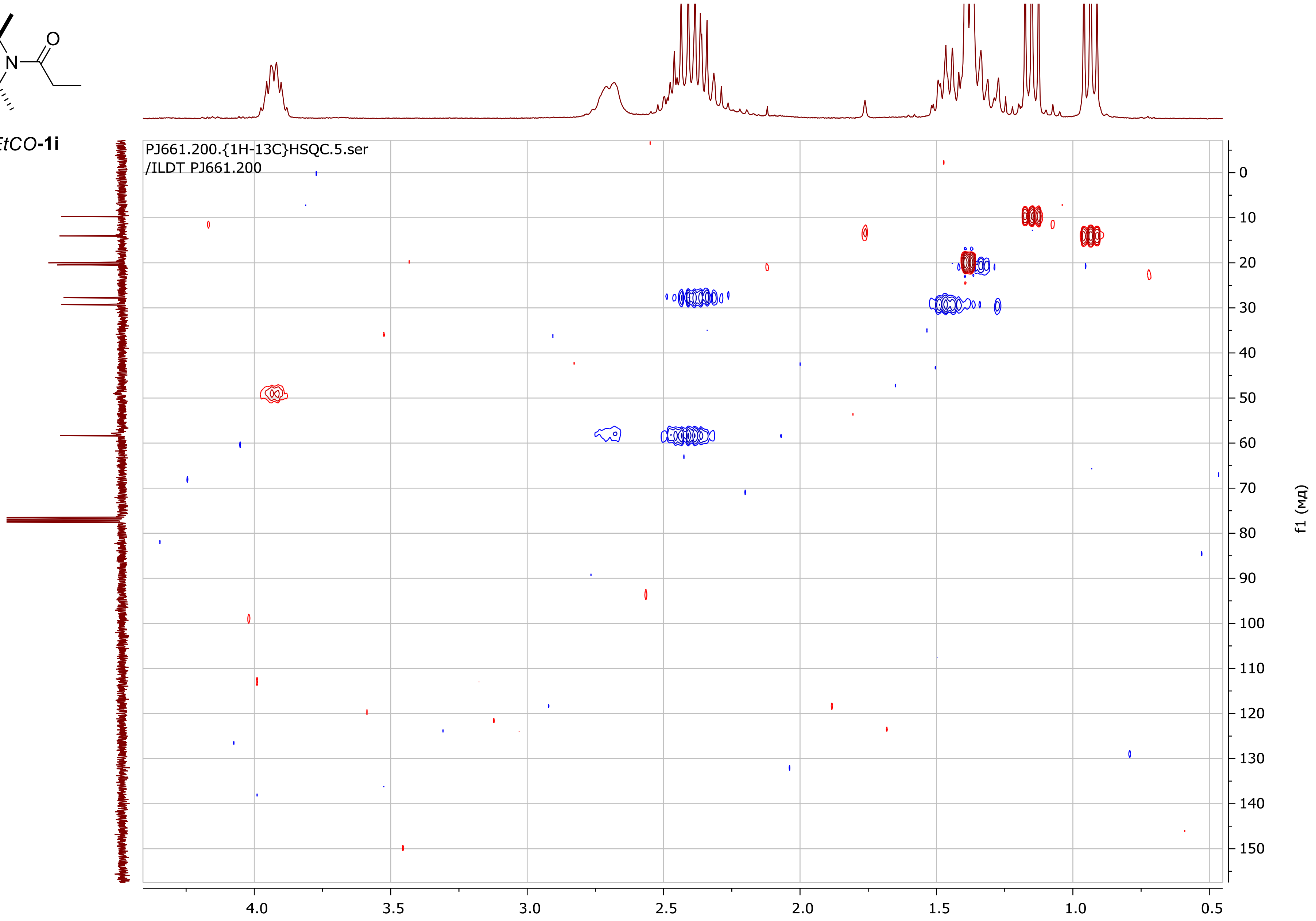

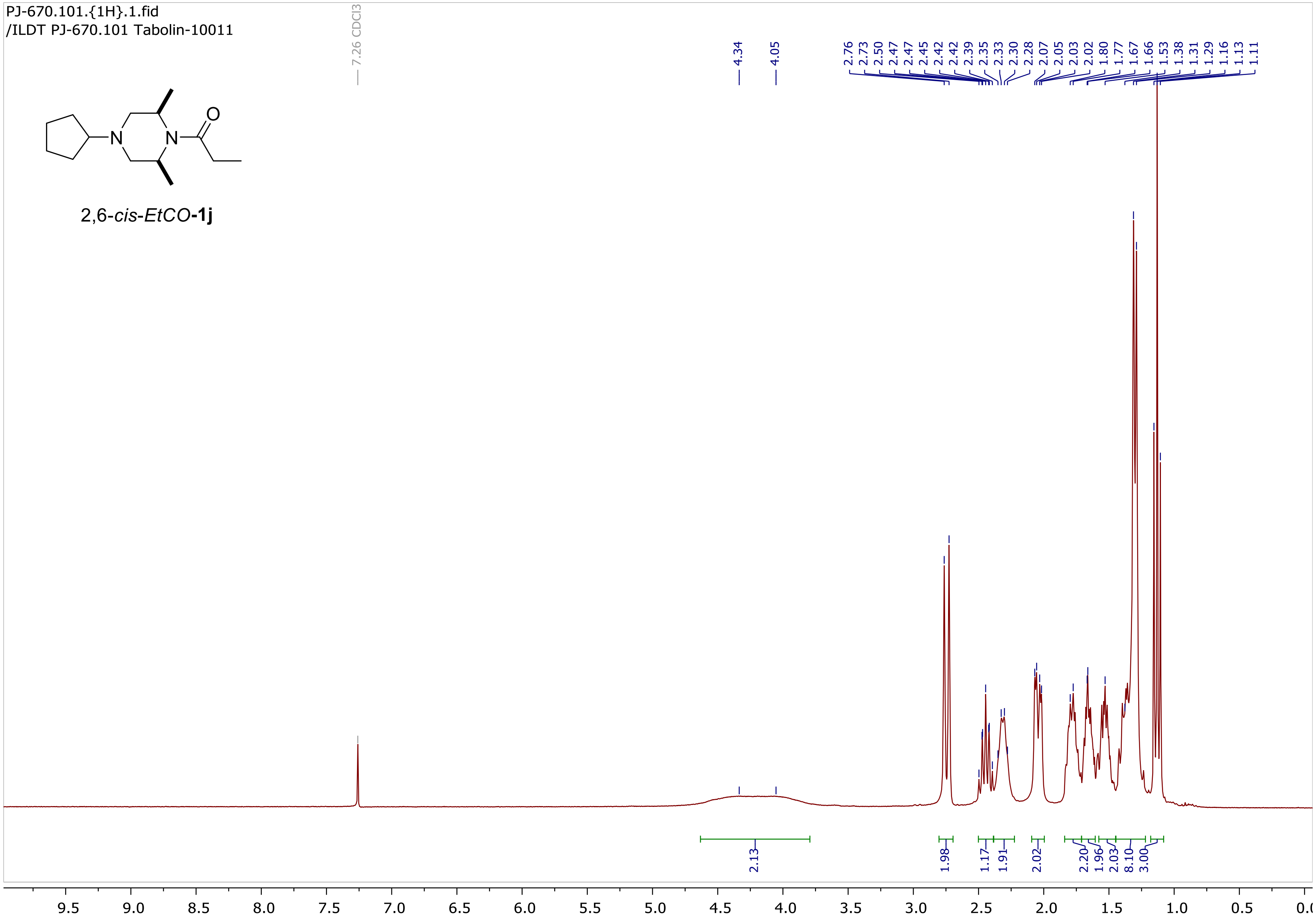

2,6-*cis*-*EtCO*-**1j**

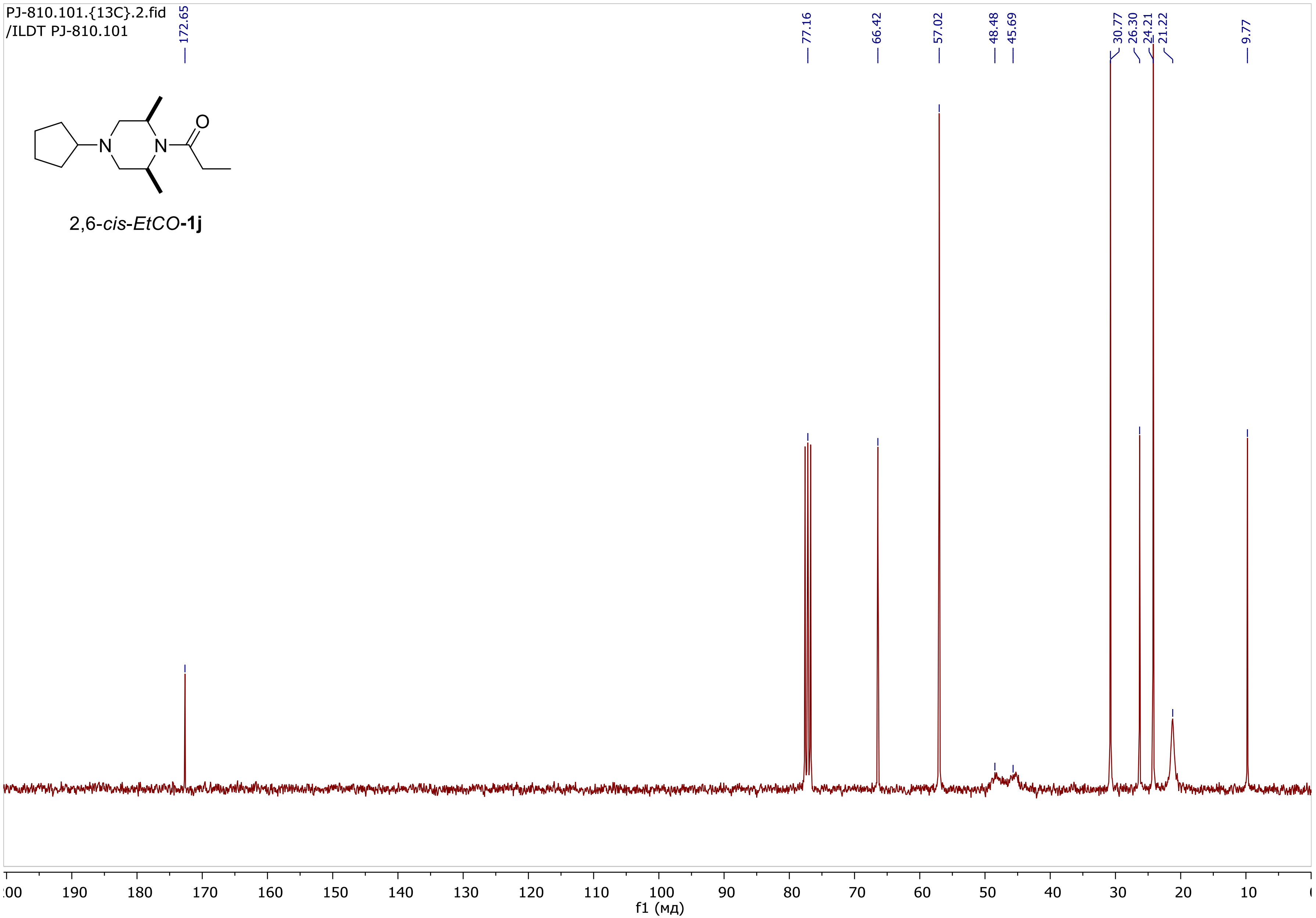
PJ-810.101.{13C}.2.fid
/ILDT PJ-810.101
172.65
77.16
66.42
57.02
48.48
45.69
30.77
26.30
24.21
21.22
9.77
2,6-cis-EtCO-1j
f1 (мд)

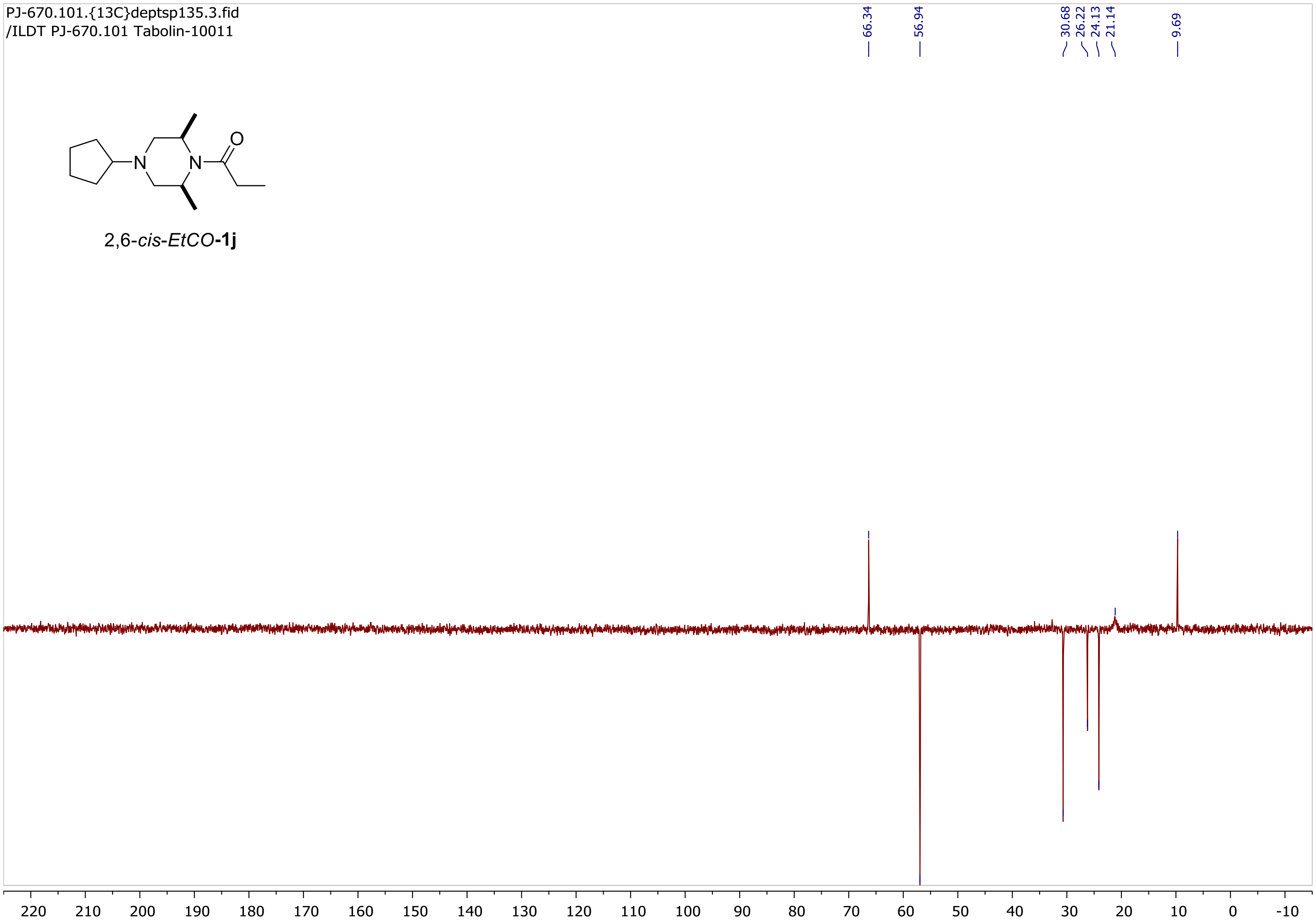
PJ-670.101.{13C}deptsp135.3.fid
/ILDT PJ-670.101 Tabolin-10011
66.34
56.94
30.68
26.22
24.13
21.14
9.69
2,6-*cis*-*EtCO*-**1j**
220 210 200 190 180 170 160 150 140 130 120 110 100 90 80 70 60 50 40 30 20 10 0 -10

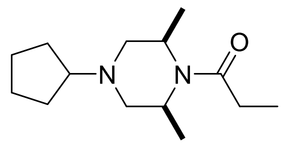

2,6-*cis*-*EtCO*-**1j**

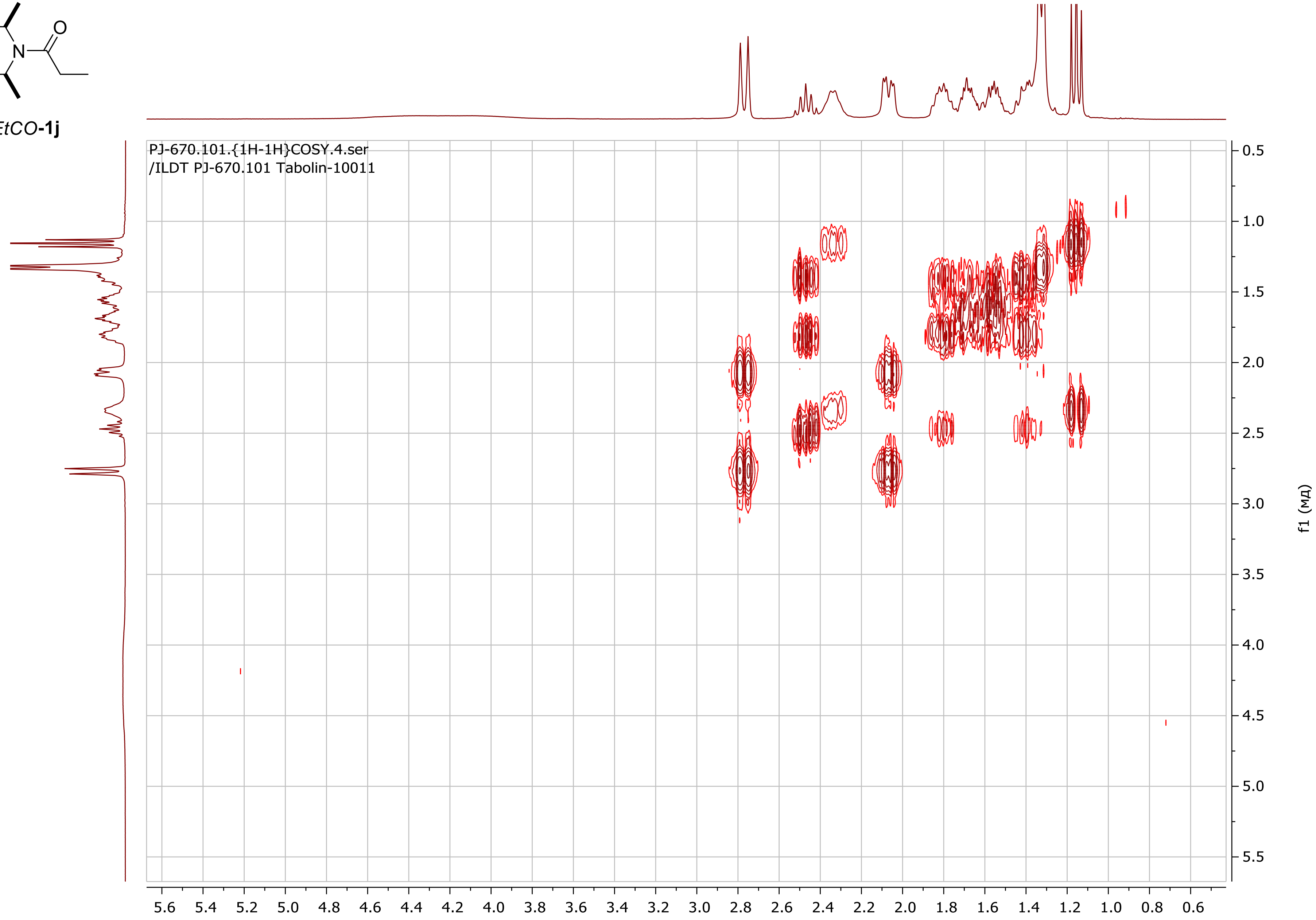

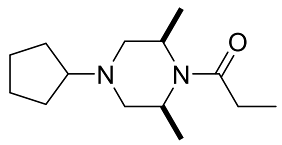

2,6-*cis*-*EtCO*-**1j**

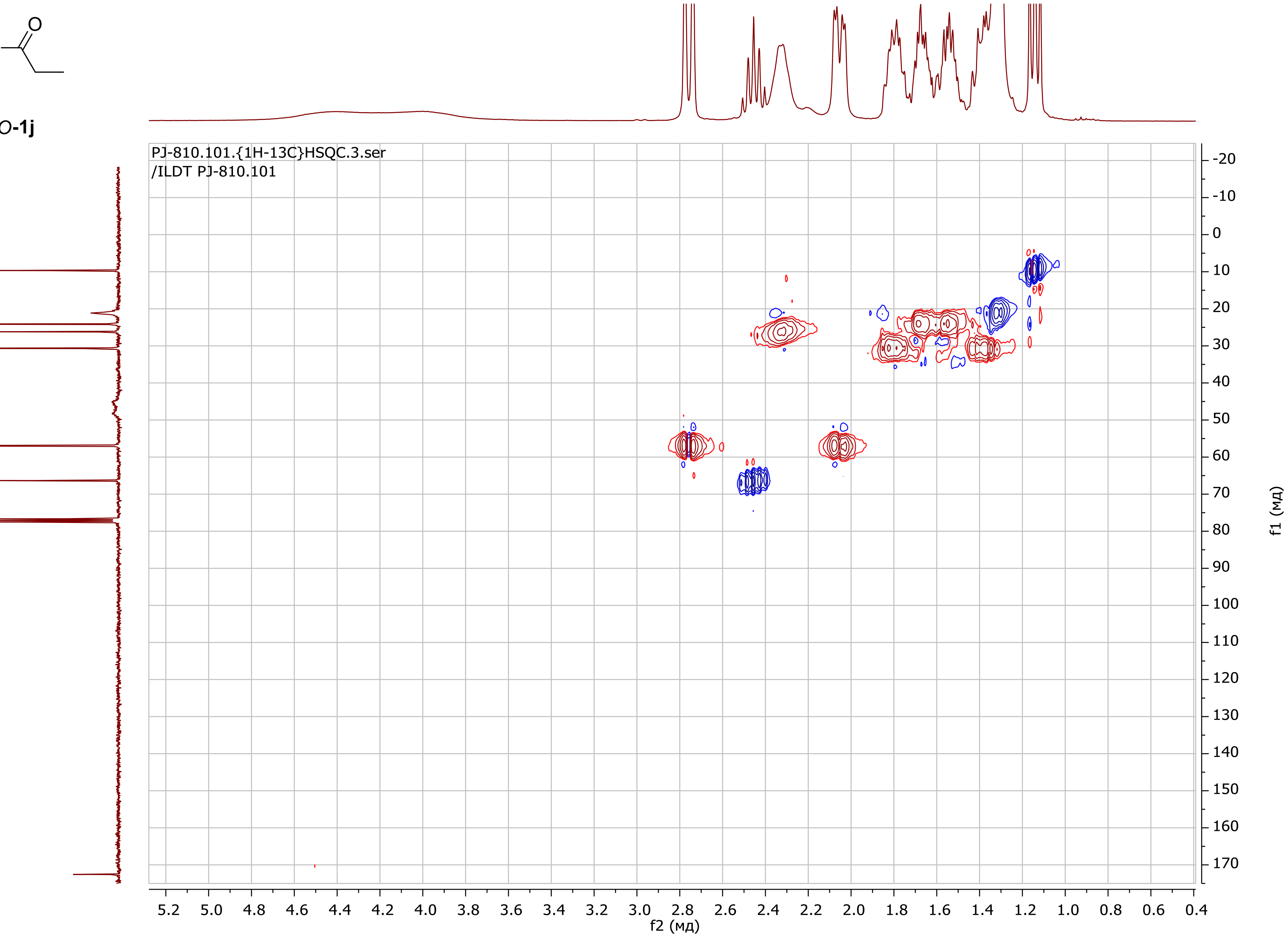

PJ-670.300.{1H}.1.fid
/ILDT PJ-670.300 Tabolin-10011

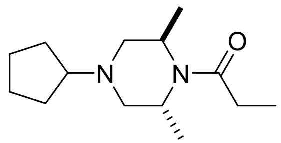

2,6-*trans*-*EtCO*-**1j**

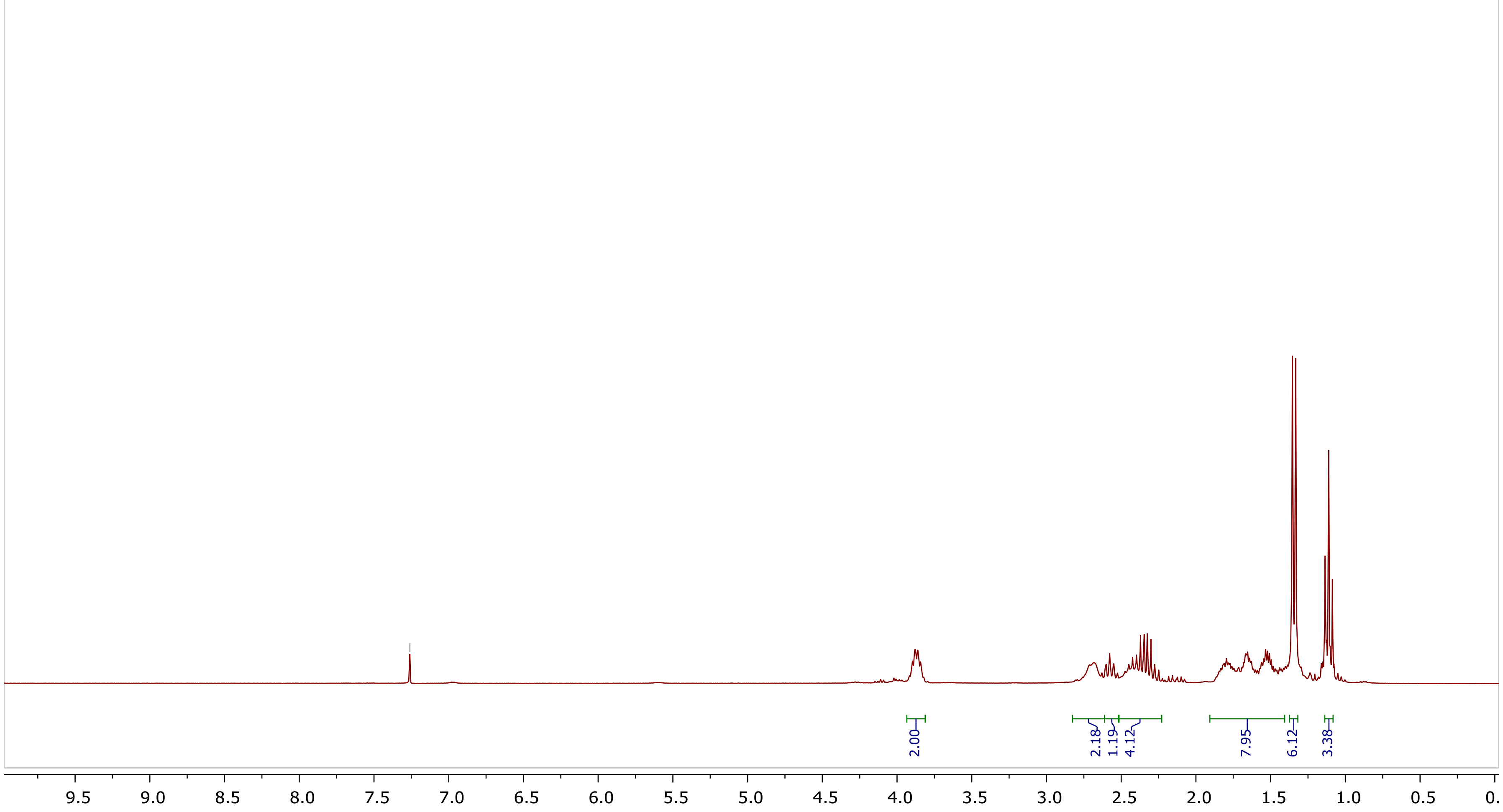

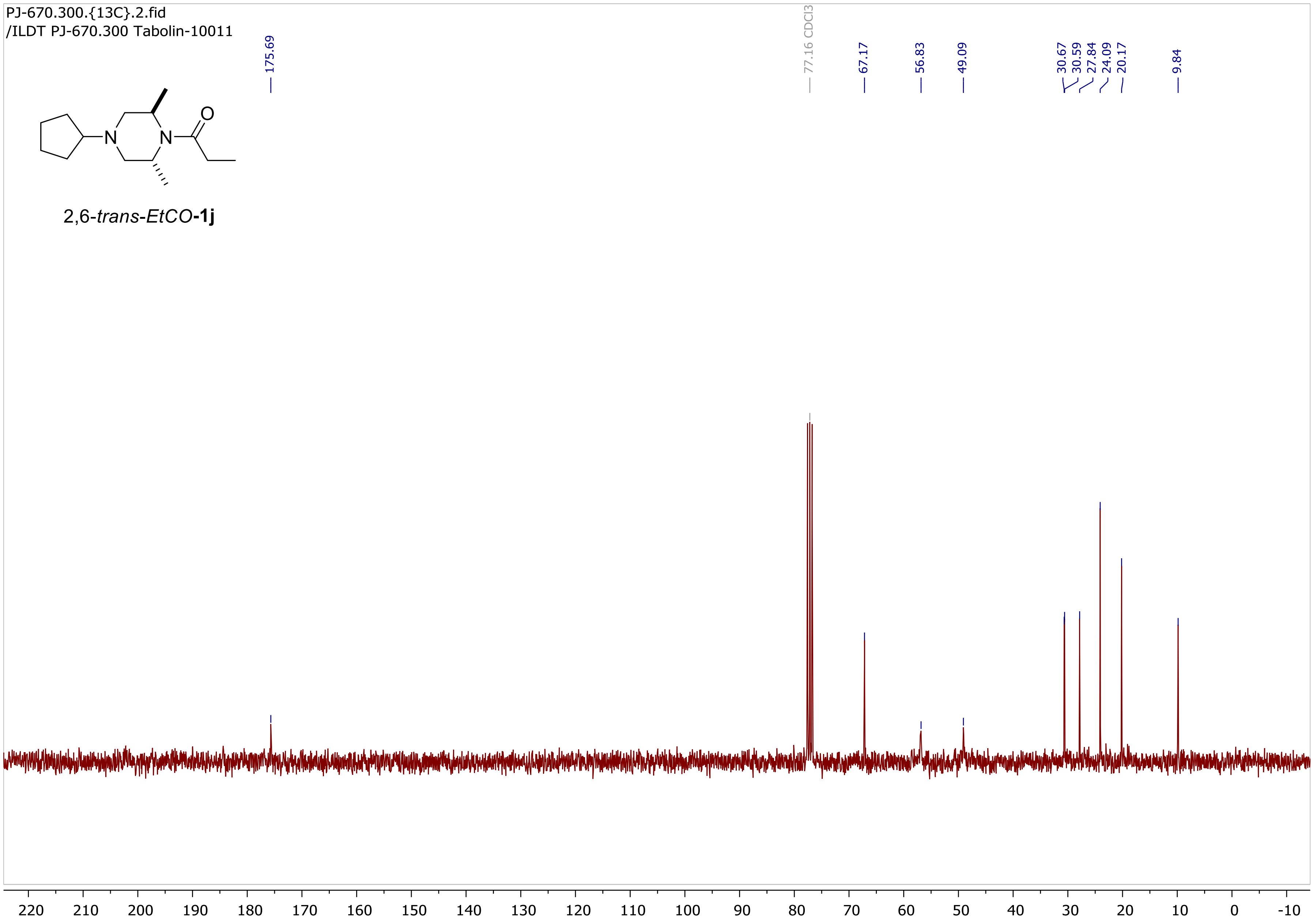
PJ-670.300.{13C}.2.fid
/ILDT PJ-670.300 Tabolin-10011
2,6-*trans*-EtCO-**1j**
175.69
77.16 CDCl3
67.17
56.83
49.09
30.67
30.59
27.84
24.09
20.17
9.84
220 210 200 190 180 170 160 150 140 130 120 110 100 90 80 70 60 50 40 30 20 10 0 -10

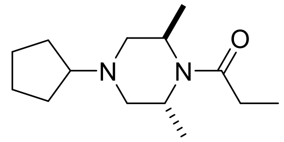

2,6-*trans*-EtCO-**1j**

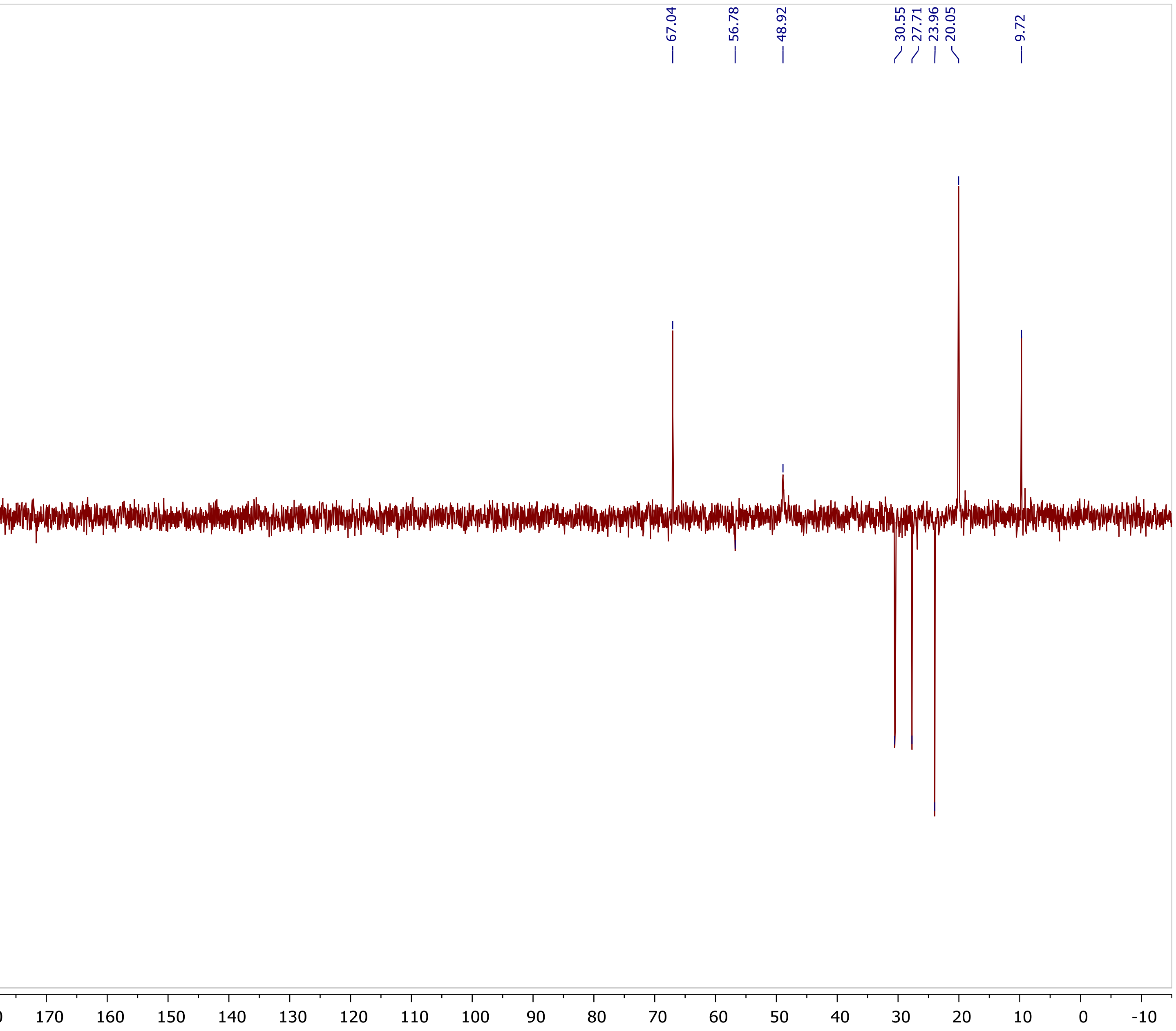

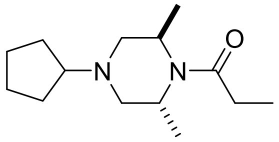

2,6-*trans*-*EtCO*-**1j**

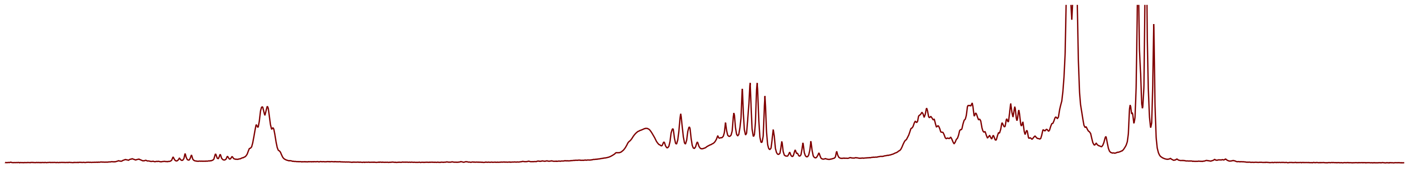

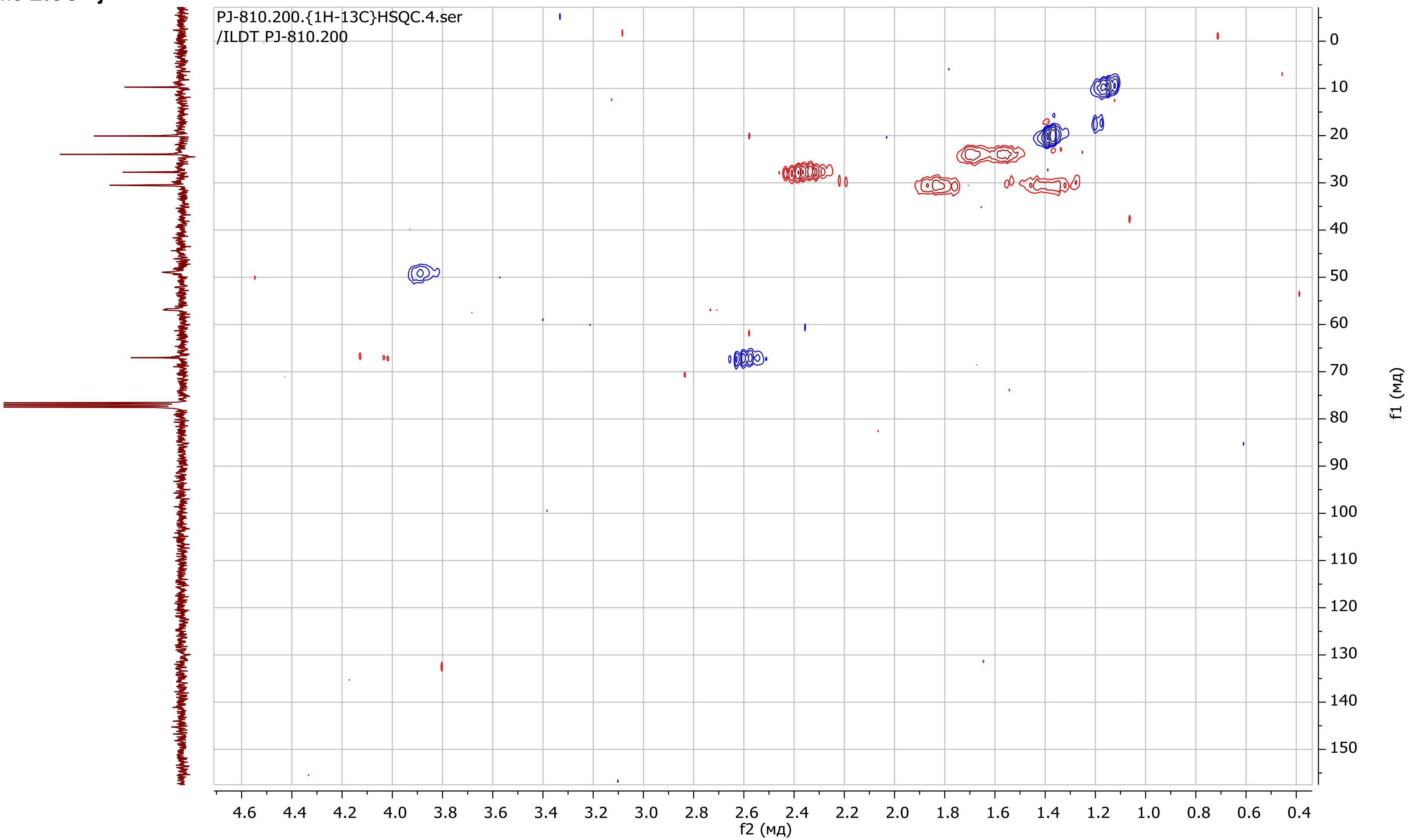

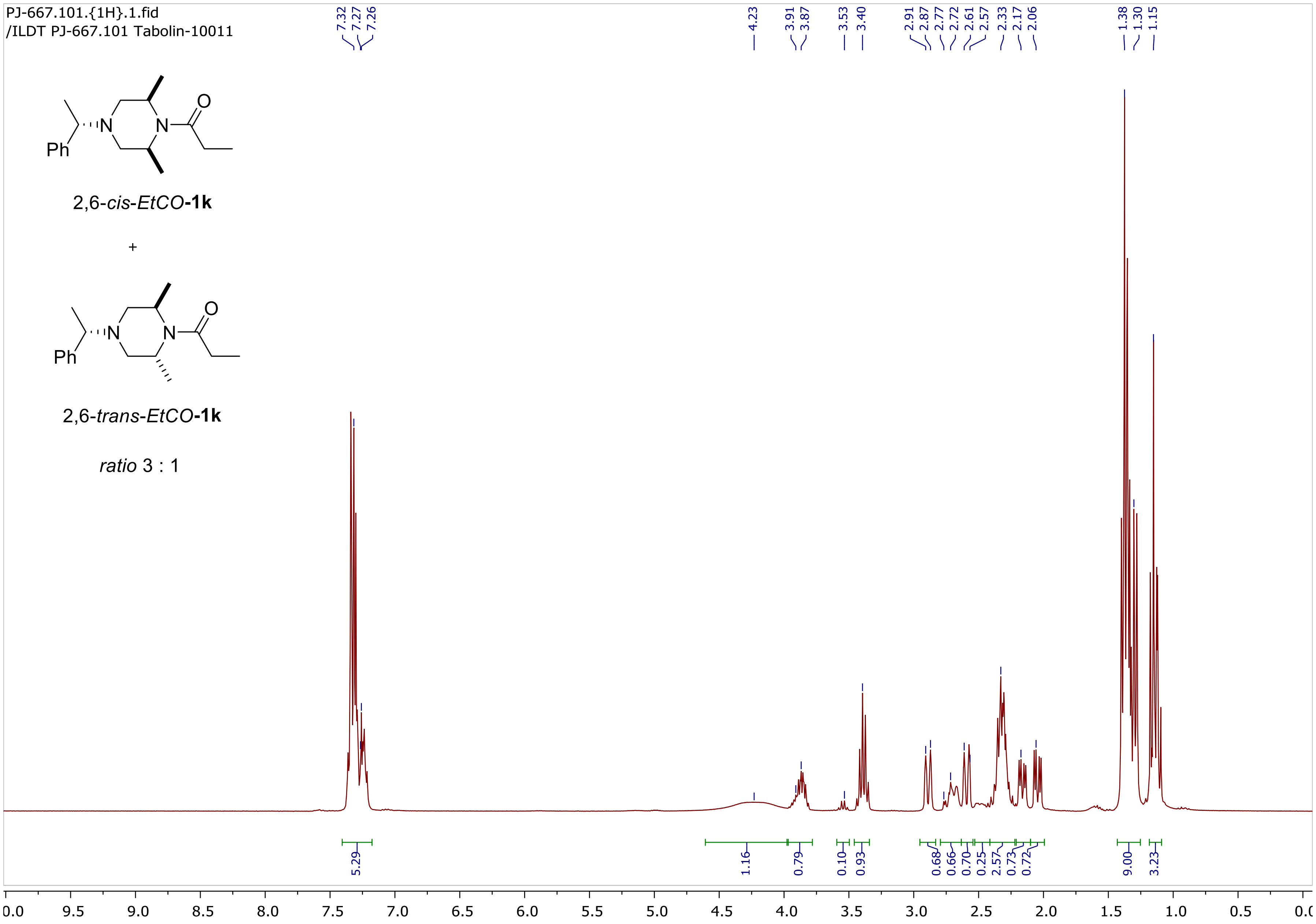
PJ-667.101.{1H}.1.fid
/ILDT PJ-667.101 Tabolin-10011
7.32
7.27
7.26
4.23
3.91
3.87
3.53
3.40
2.91
2.87
2.77
2.72
2.61
2.57
2.33
2.17
2.06
1.38
1.30
1.15
2,6-*cis-EtCO*-**1k**
+
2,6-*trans-EtCO*-**1k**
ratio 3 : 1
Ph
N
O
5.29
1.16
0.79
0.10
0.93
0.68
0.66
0.70
0.25
2.57
0.73
0.72
9.00
3.23
0.0
9.5
9.0
8.5
8.0
7.5
7.0
6.5
6.0
5.5
5.0
4.5
4.0
3.5
3.0
2.5
2.0
1.5
1.0
0.5
0.0

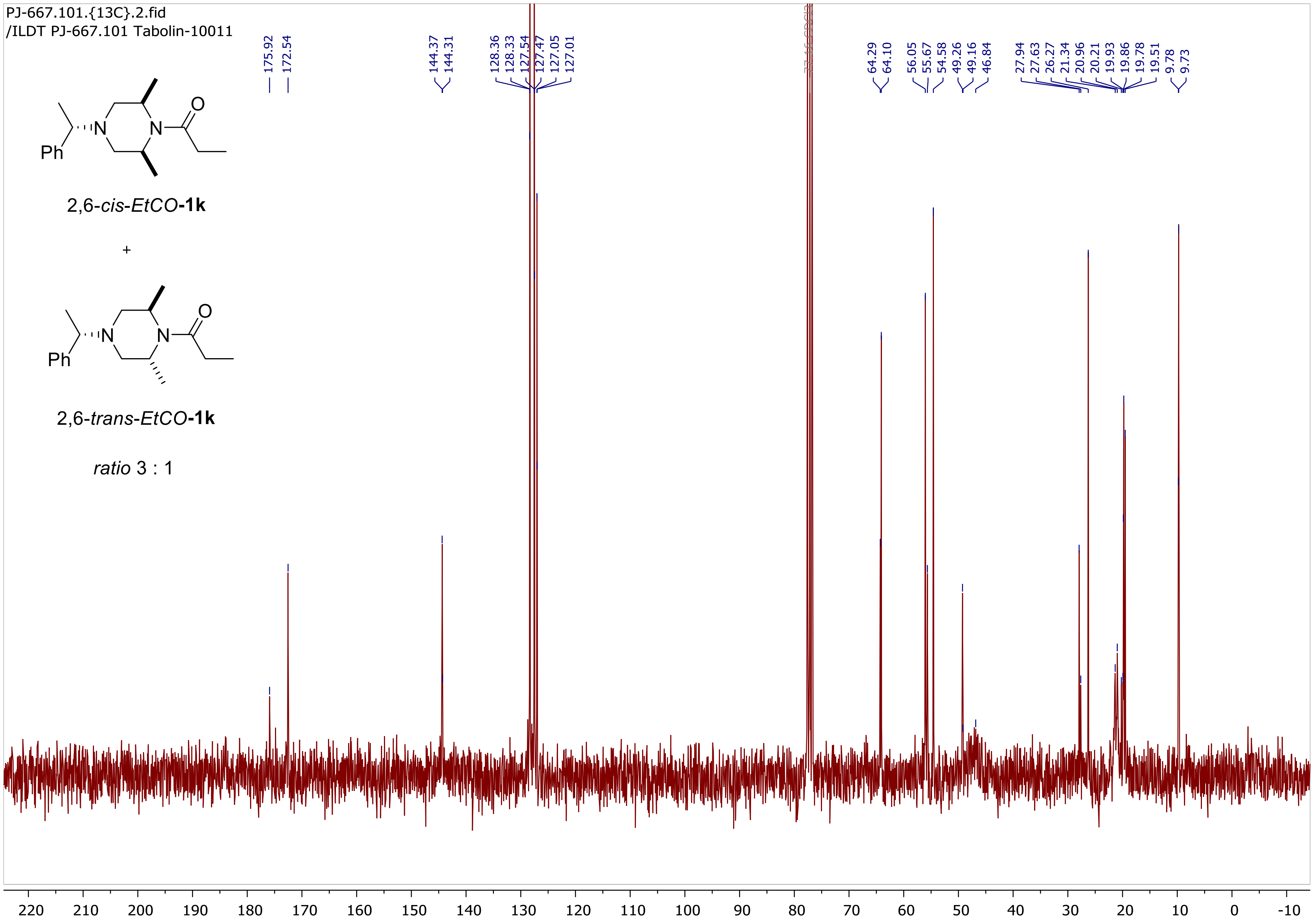
PJ-667.101.{13C}.2.fid
/ILDT PJ-667.101 Tabolin-10011
175.92
172.54
144.37
144.31
128.36
128.33
127.54
127.47
127.05
127.01
64.29
64.10
56.05
55.67
54.58
49.26
49.16
46.84
27.94
27.63
26.27
21.34
20.96
20.21
19.93
19.86
19.78
19.51
9.78
9.73
Ph
N
O
2,6-cis-EtCO-1k
+
2,6-trans-EtCO-1k
ratio 3 : 1
220 210 200 190 180 170 160 150 140 130 120 110 100 90 80 70 60 50 40 30 20 10 0 -10

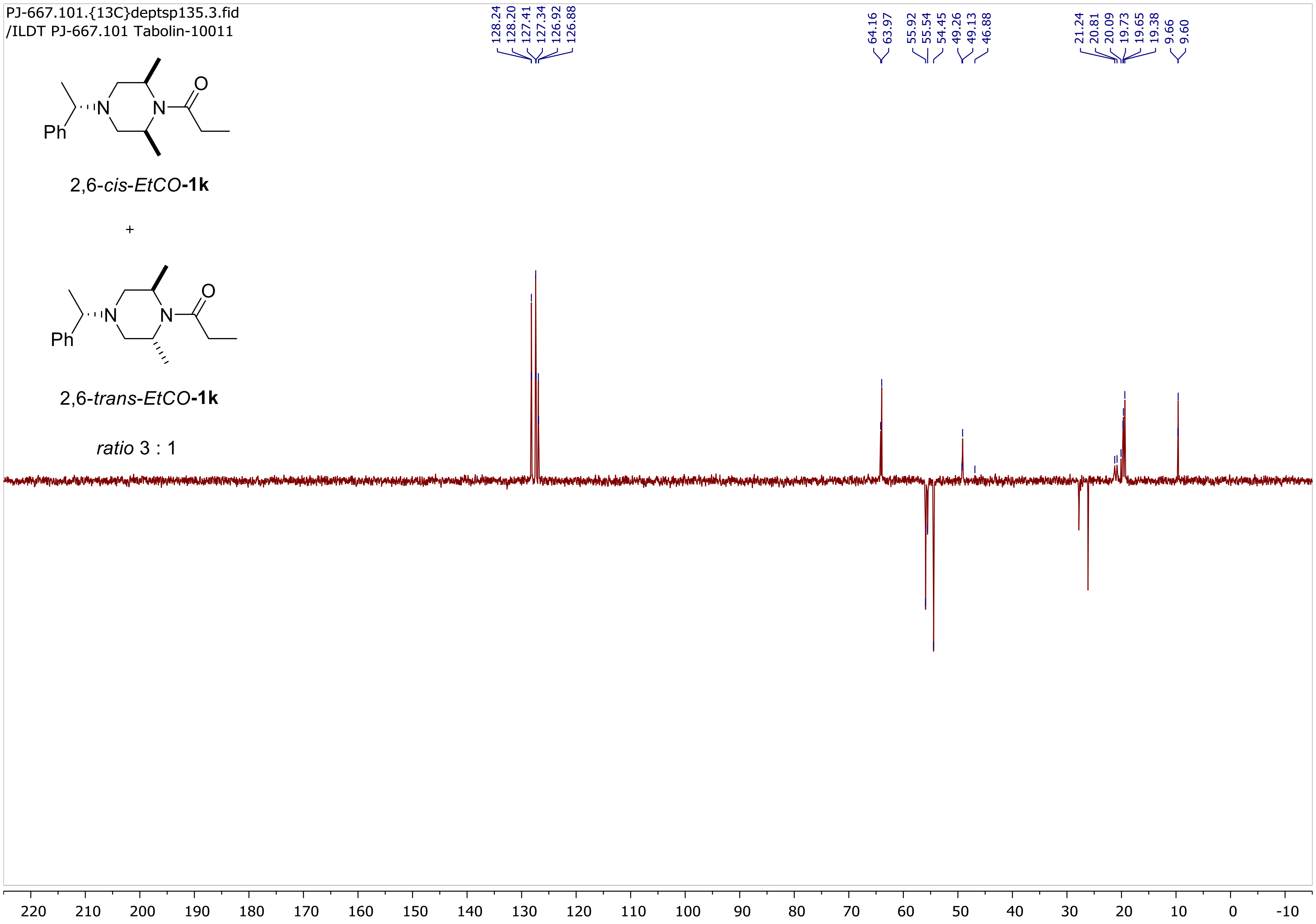

2,6-*cis*-*EtCO*-**1k**

+

2,6-*trans*-*EtCO*-**1k**

ratio 3 : 1

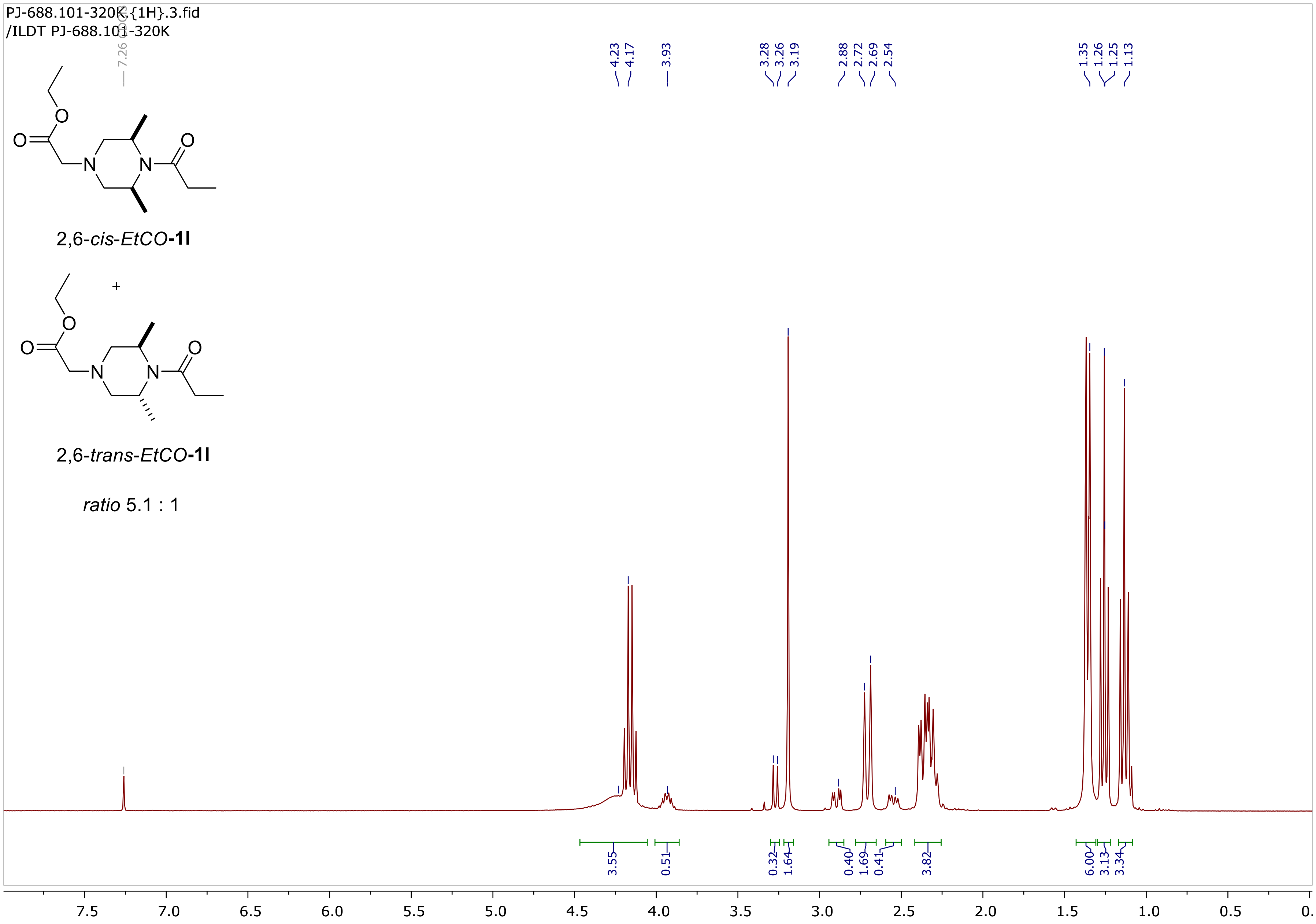
PJ-688.101-320K.{1H}.3.fid
/ILDT PJ-688.101-320K
2,6-cis-EtCO-1l
+
2,6-trans-EtCO-1l
ratio 5.1 : 1
7.26
4.23
4.17
3.93
3.28
3.26
3.19
2.88
2.72
2.69
2.54
1.35
1.26
1.25
1.13
3.55
0.51
0.32
1.64
0.40
1.69
0.41
3.82
6.00
3.13
3.34
7.5
7.0
6.5
6.0
5.5
5.0
4.5
4.0
3.5
3.0
2.5
2.0
1.5
1.0
0.5
0.

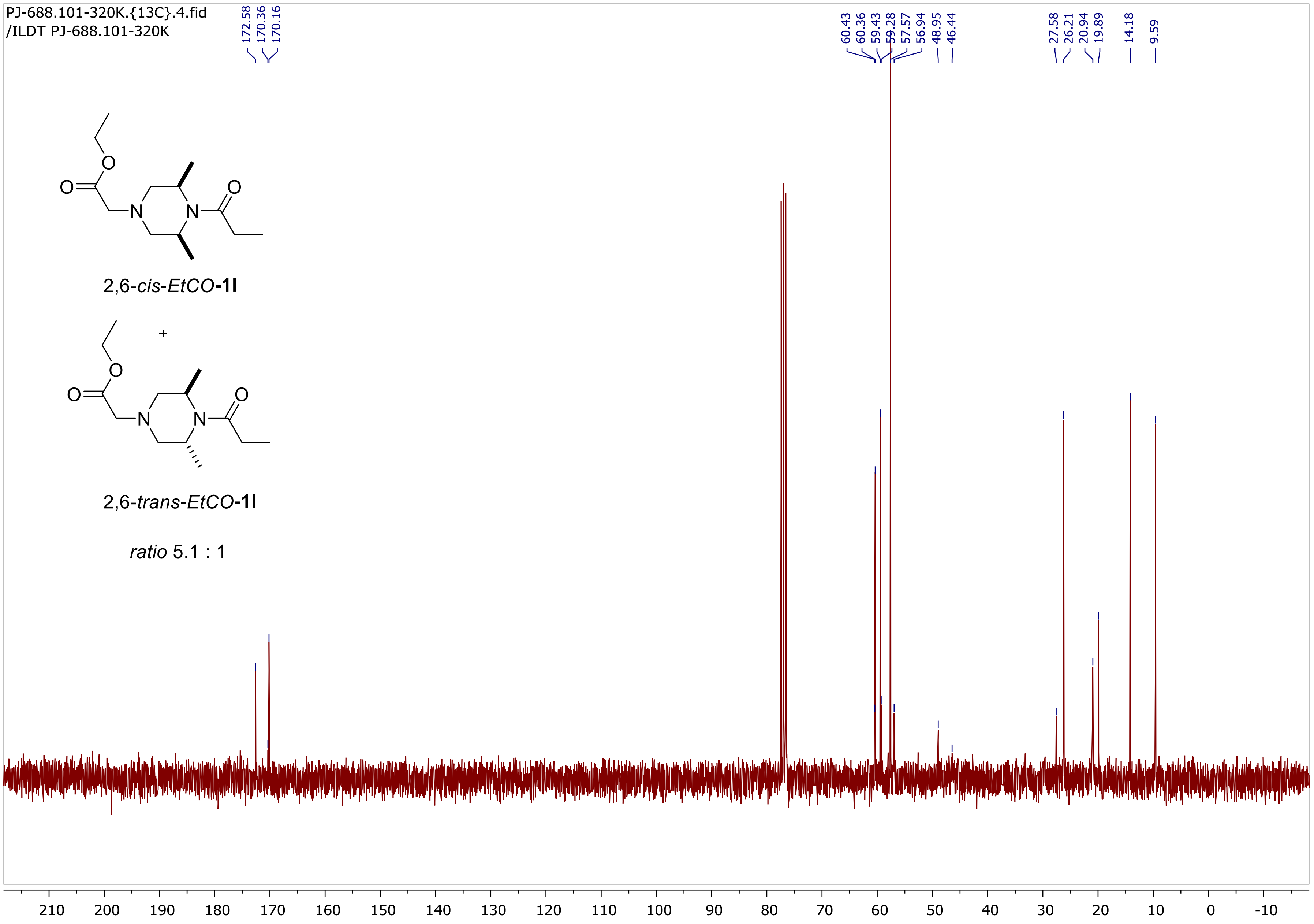
PJ-688.101-320K.{13C}.4.fid
/ILDT PJ-688.101-320K
172.58
170.36
170.16
60.43
60.36
59.43
59.28
57.57
56.94
48.95
46.44
27.58
26.21
20.94
19.89
14.18
9.59
2,6-*cis*-*EtCO*-**1l**
+
2,6-*trans*-*EtCO*-**1l**
ratio 5.1 : 1
210 200 190 180 170 160 150 140 130 120 110 100 90 80 70 60 50 40 30 20 10 0 -10

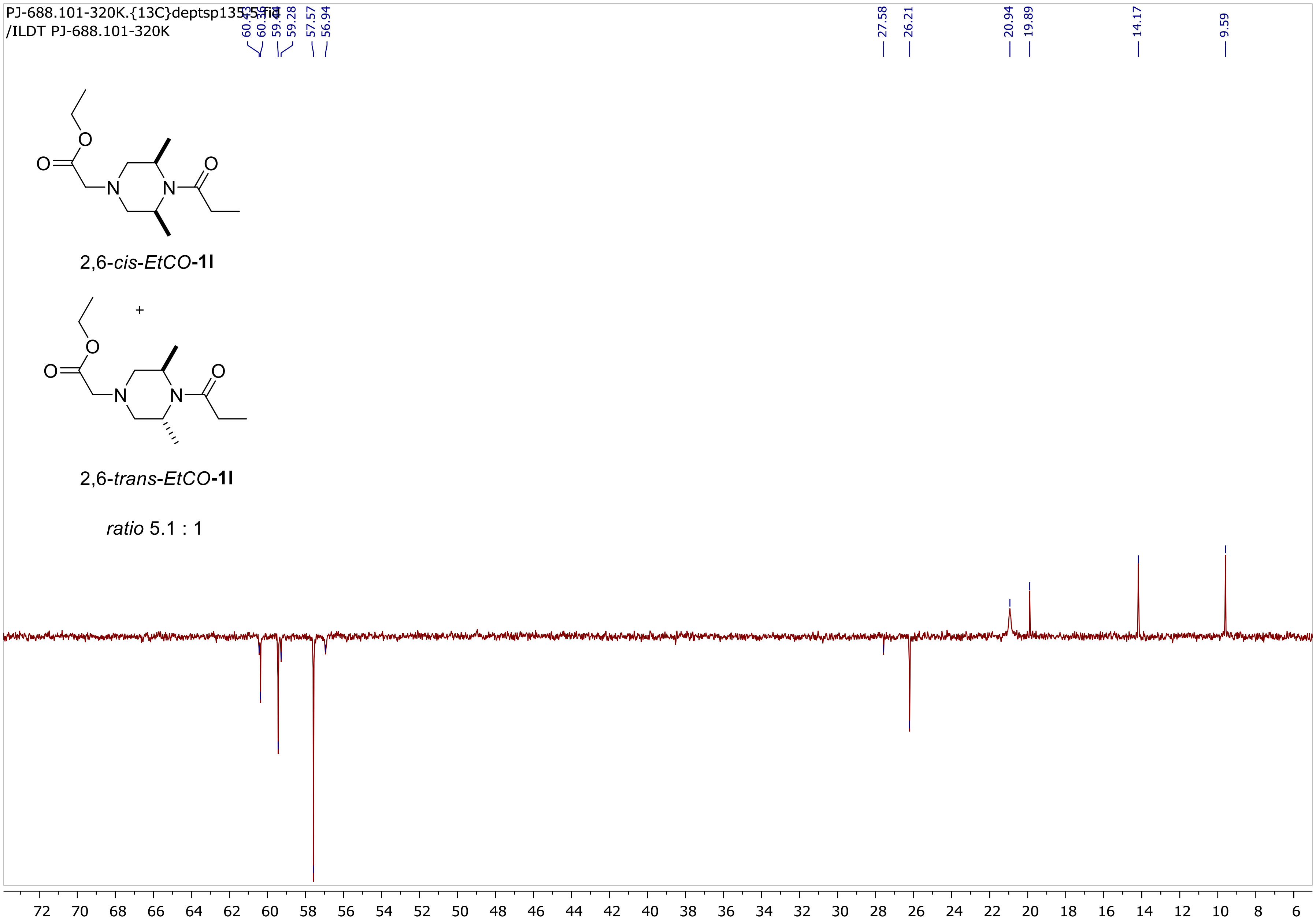

PJ-688.101-320K.{13C}deptsp135.5.fid
/ILDT PJ-688.101-320K
60.43
60.36
59.44
59.28
57.57
56.94
27.58
26.21
20.94
19.89
14.17
9.59
O
N
2,6-cis-EtCO-1l
+
2,6-trans-EtCO-1l
ratio 5.1 : 1
72
70
68
66
64
62
60
58
56
54
52
50
48
46
44
42
40
38
36
34
32
30
28
26
24
22
20
18
16
14
12
10
8
6

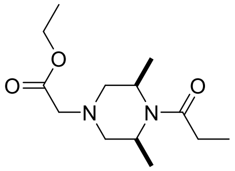

2,6-*cis*-*EtCO*-**1l**

+

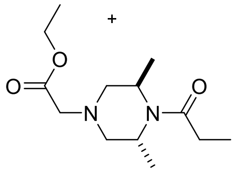

2,6-*trans*-*EtCO*-**1l**

ratio 5.1 : 1

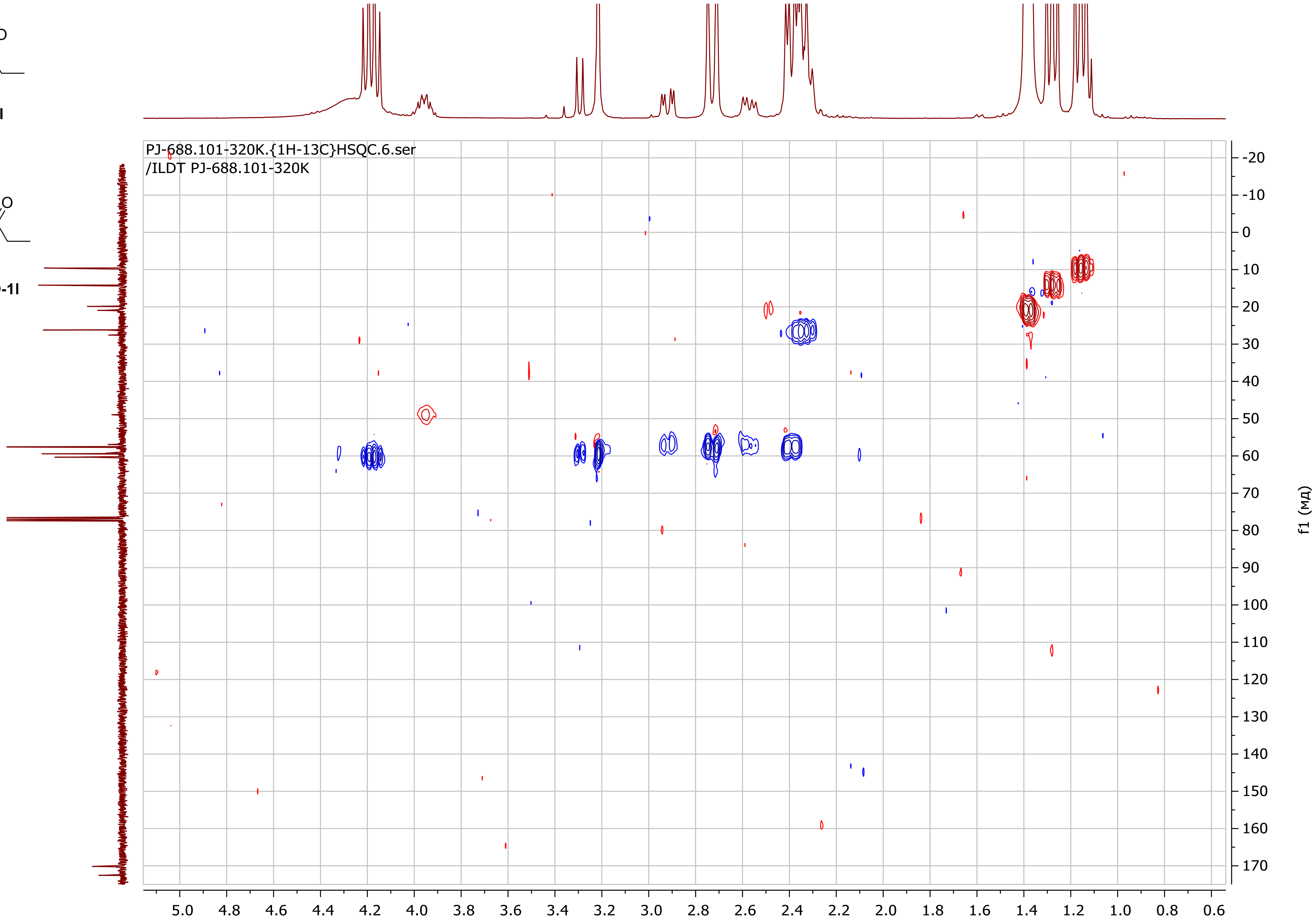

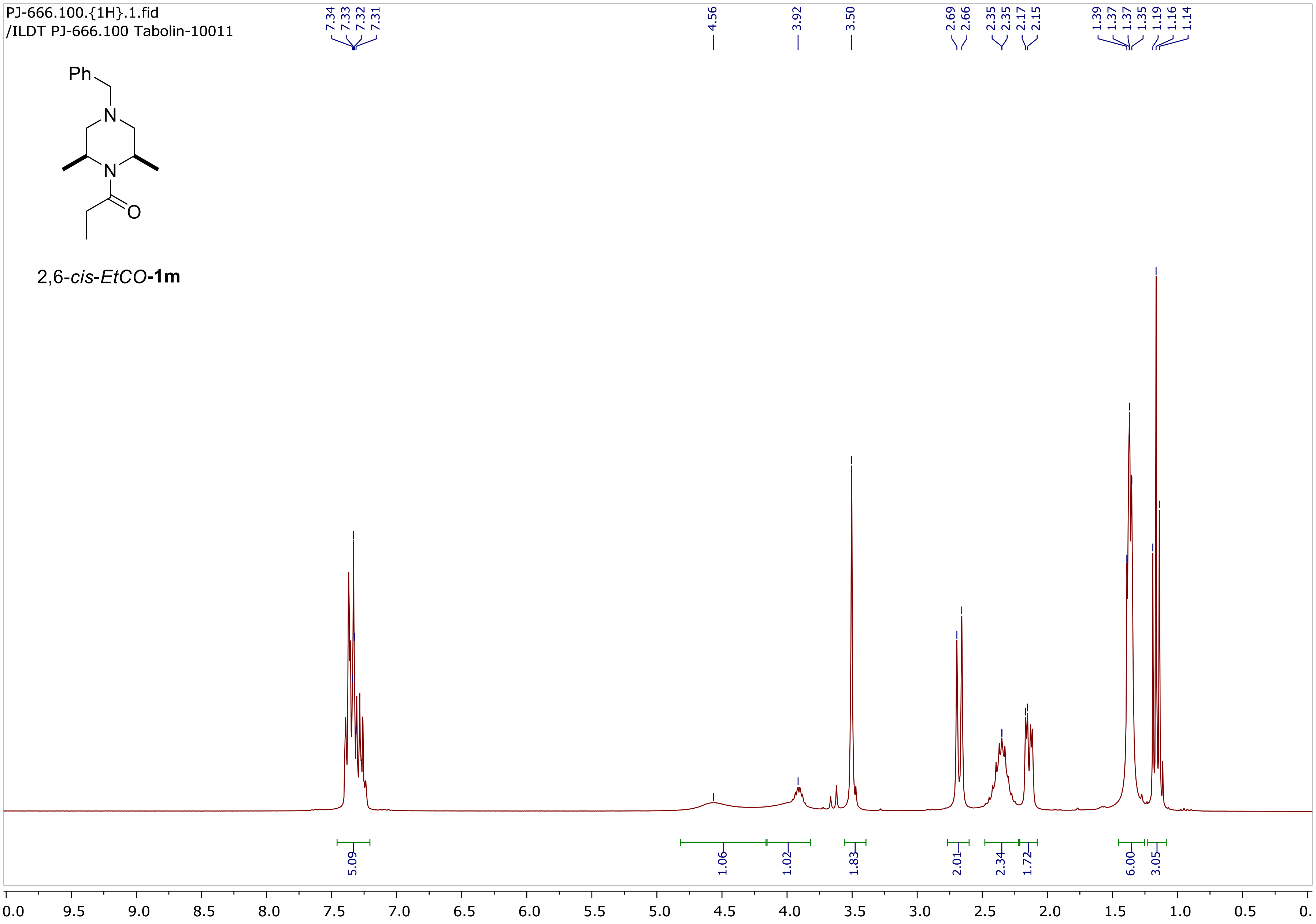
PJ-666.100.{1H}.1.fid
/ILDT PJ-666.100 Tabolin-10011
7.34
7.33
7.32
7.31
4.56
3.92
3.50
2.69
2.66
2.35
2.35
2.17
2.15
1.39
1.37
1.37
1.35
1.19
1.16
1.14
Ph
N
N
O
2,6-cis-EtCO-1m
5.09
1.06
1.02
1.83
2.01
2.34
1.72
6.00
3.05
0.0
9.5
9.0
8.5
8.0
7.5
7.0
6.5
6.0
5.5
5.0
4.5
4.0
3.5
3.0
2.5
2.0
1.5
1.0
0.5
0.

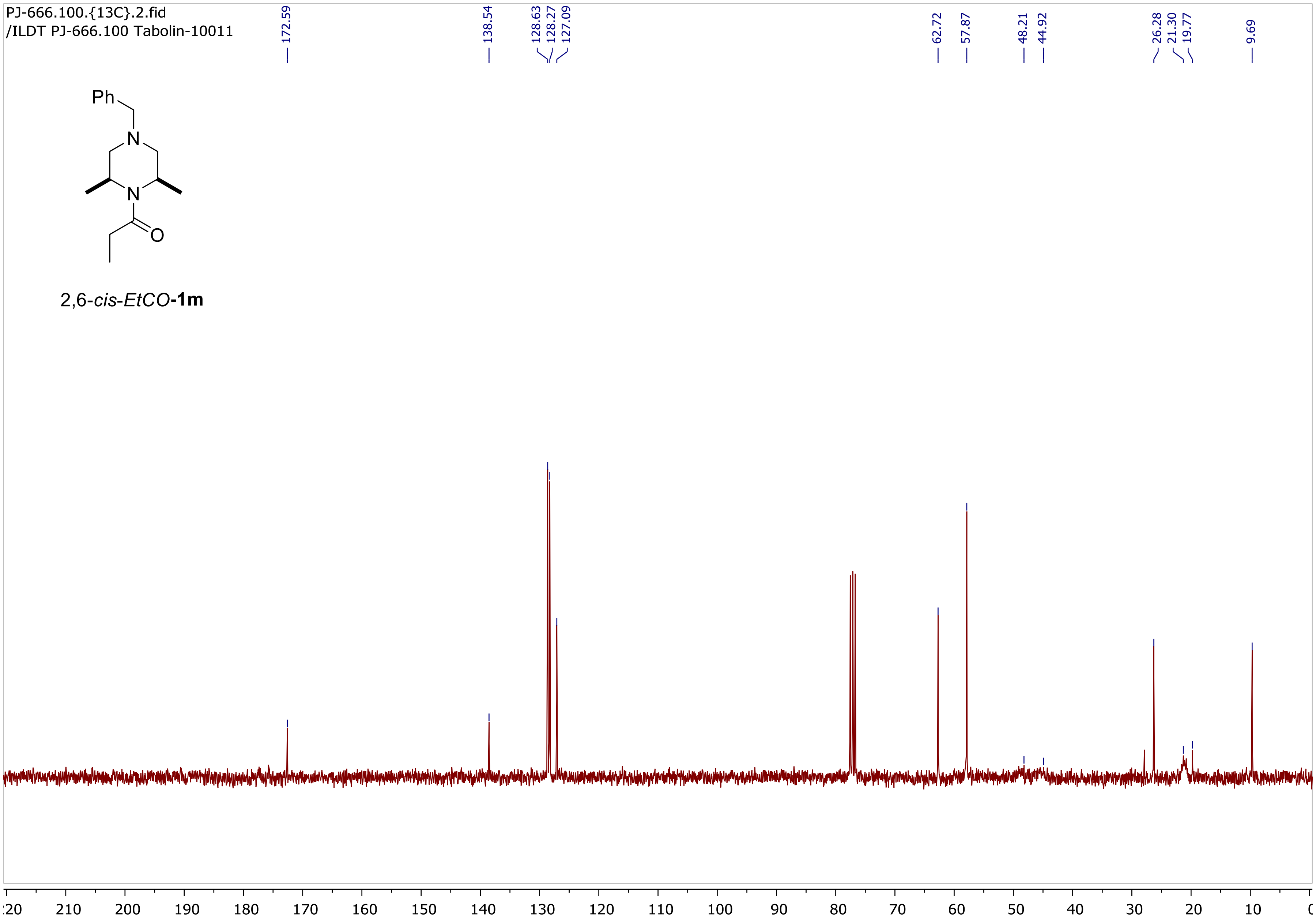

PJ-666.100.{13C}.2.fid
/ILDT PJ-666.100 Tabolin-10011
172.59
138.54
128.63
128.27
127.09
62.72
57.87
48.21
44.92
26.28
21.30
19.77
9.69
Ph
N
N
O
2,6-cis-EtCO-1m
210
200
190
180
170
160
150
140
130
120
110
100
90
80
70
60
50
40
30
20
10

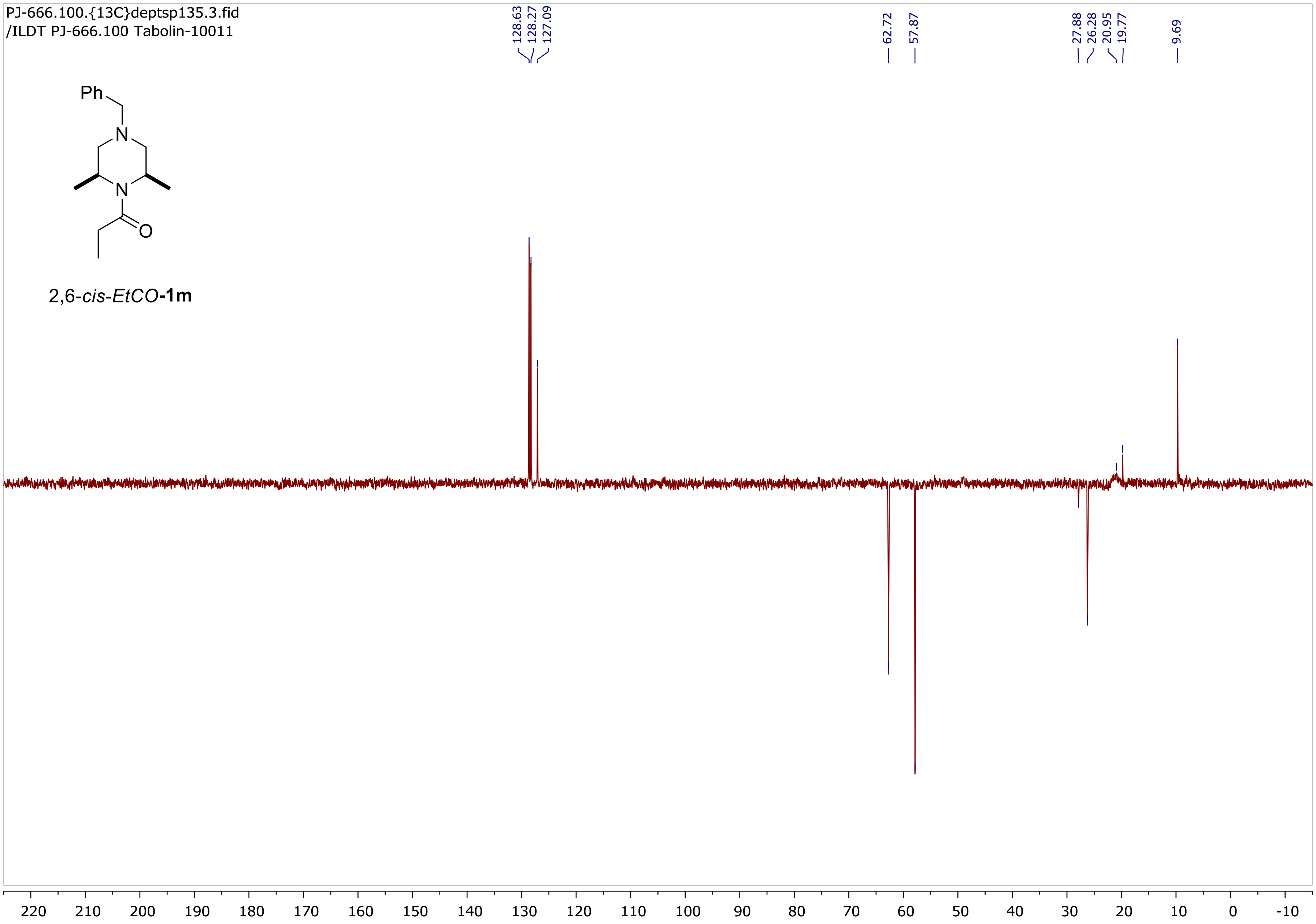
PJ-666.100.{13C}deptsp135.3.fid
/ILDT PJ-666.100 Tabolin-10011
128.63
128.27
127.09
62.72
57.87
27.88
26.28
20.95
19.77
9.69
Ph
N
N
O
2,6-*cis*-*EtCO*-**1m**
220
210
200
190
180
170
160
150
140
130
120
110
100
90
80
70
60
50
40
30
20
10
0
-10

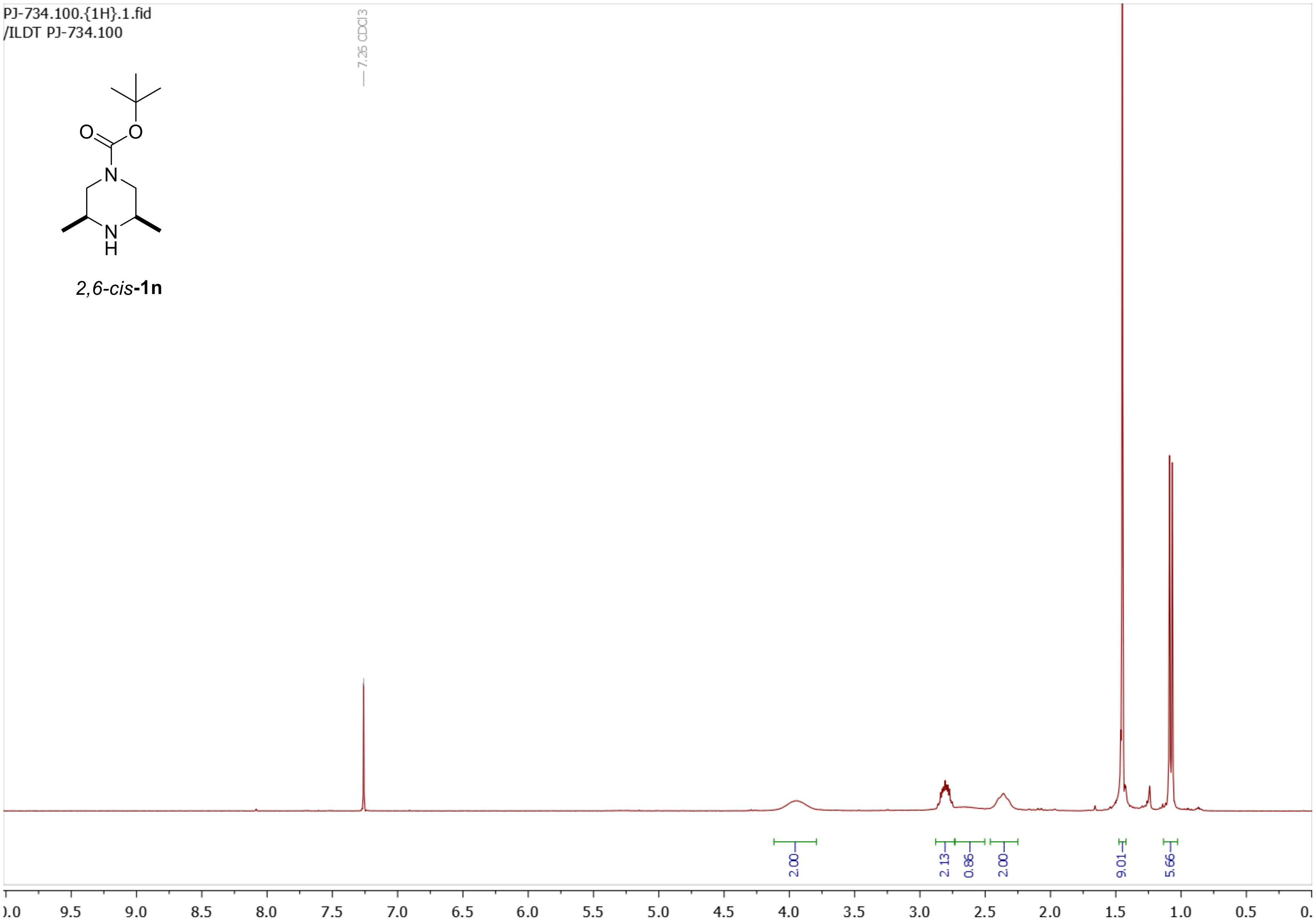
PJ-734.100.{1H}.1.fid
/ILDT PJ-734.100
7.26 CDCl3
2,6-*cis*-**1n**
2.00
2.13
0.86
2.00
9.01
5.66
0.0
9.5
9.0
8.5
8.0
7.5
7.0
6.5
6.0
5.5
5.0
4.5
4.0
3.5
3.0
2.5
2.0
1.5
1.0
0.5
0.

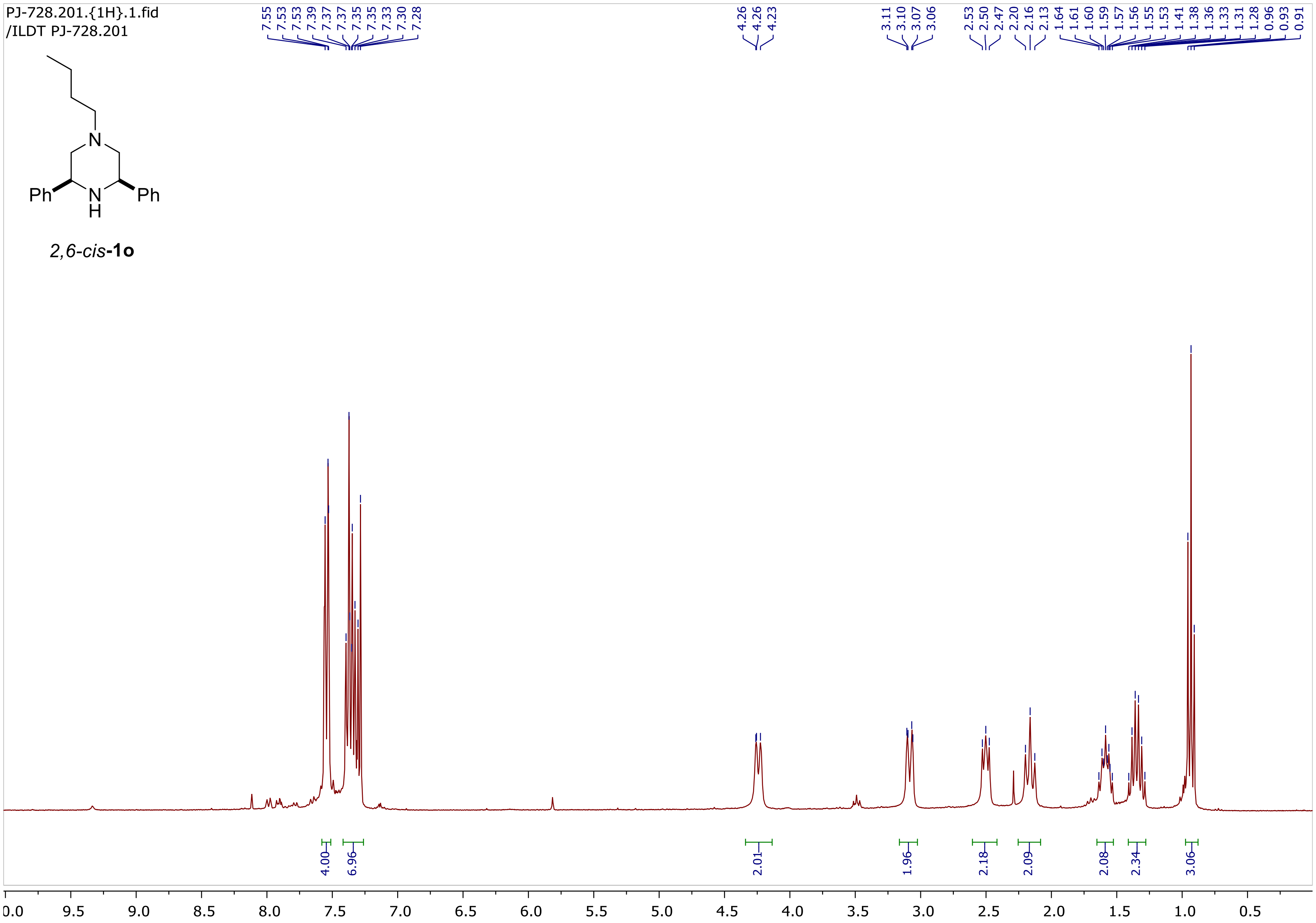
PJ-728.201.{1H}.1.fid
/ILDT PJ-728.201
7.55 7.53 7.53 7.39 7.37 7.37 7.35 7.35 7.33 7.30 7.28
4.26 4.26 4.23
3.11 3.10 3.07 3.06
2.53 2.50 2.47 2.20 2.16 2.13 1.64 1.61 1.60 1.59 1.57 1.56 1.55 1.53 1.41 1.38 1.36 1.33 1.31 1.28 0.96 0.93 0.91
N
Ph
N
H
Ph
2,6-cis-1o
4.00 6.96 2.01 1.96 2.18 2.09 2.08 2.34 3.06
10.0 9.5 9.0 8.5 8.0 7.5 7.0 6.5 6.0 5.5 5.0 4.5 4.0 3.5 3.0 2.5 2.0 1.5 1.0 0.5

PJ-728.201.{13C}.2.fid
/ILDT PJ-728.201

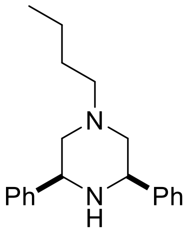

2,6-cis-**1o**

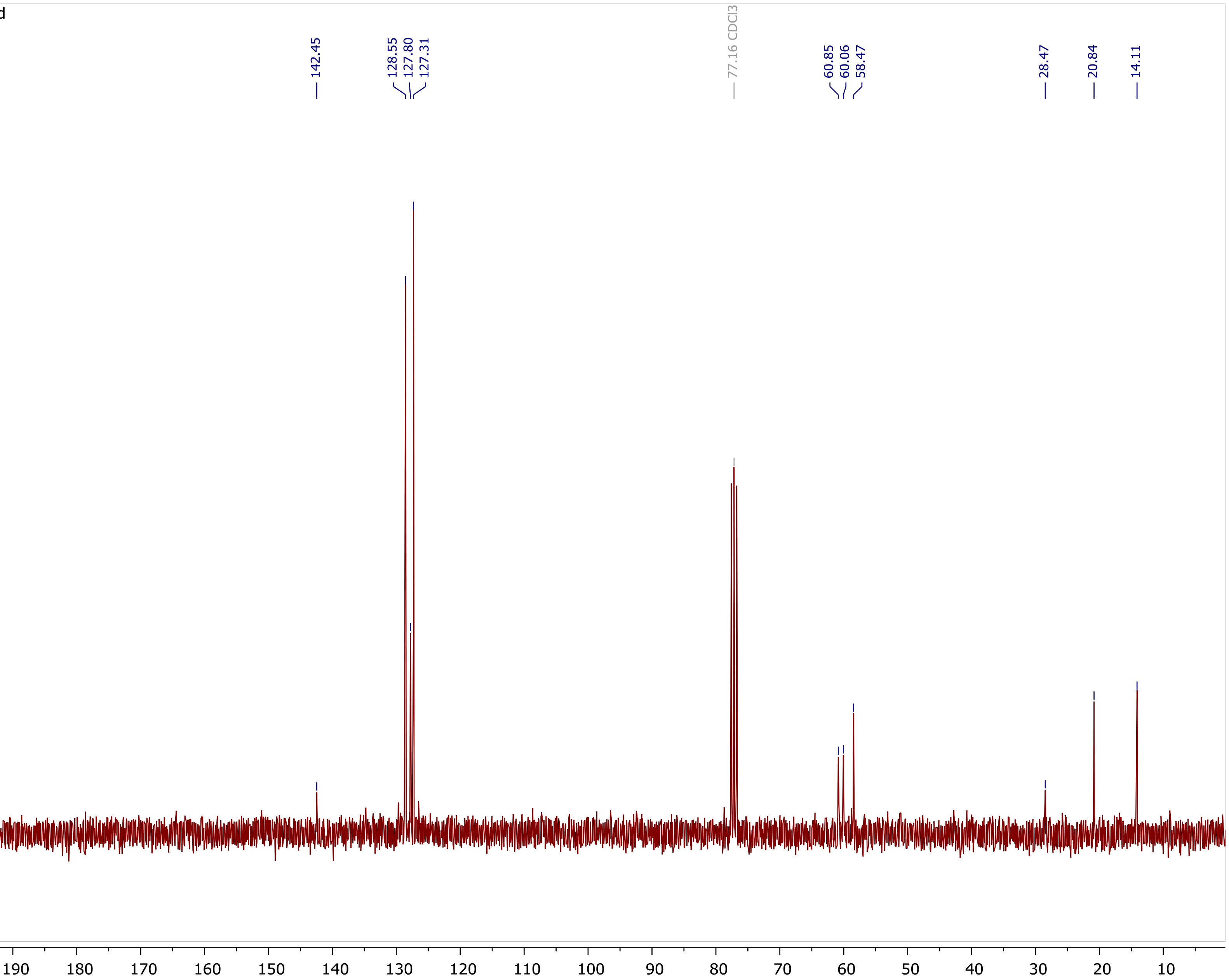

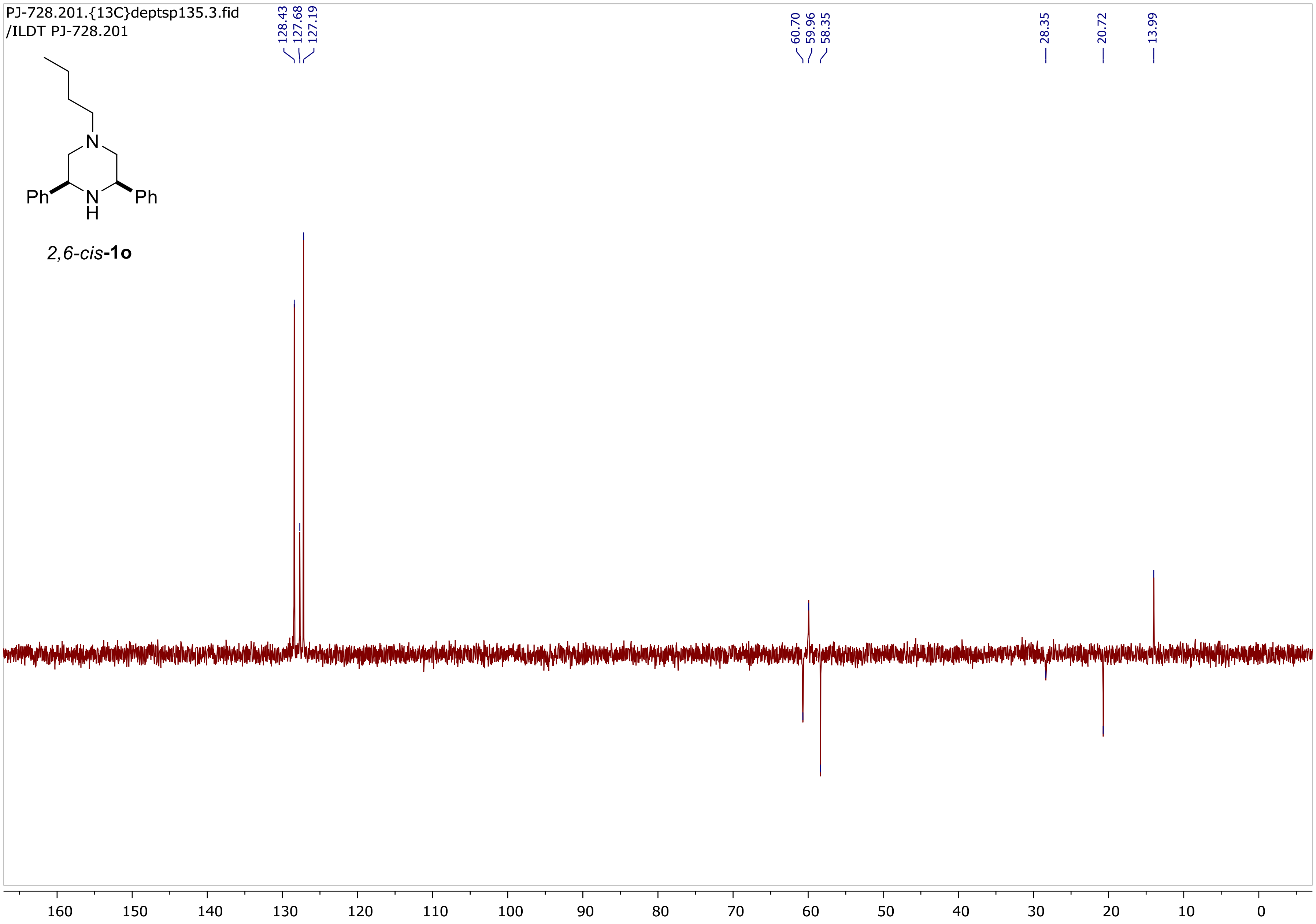
PJ-728.201.{13C}deptsp135.3.fid
/ILDT PJ-728.201
128.43
127.68
127.19
60.70
59.96
58.35
28.35
20.72
13.99
N
Ph
N
H
Ph
160
150
140
130
120
110
100
90
80
70
60
50
40
30
20
10
0

2,6-*cis*-**1o**

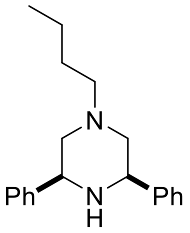

2,6-*cis*-**1o**

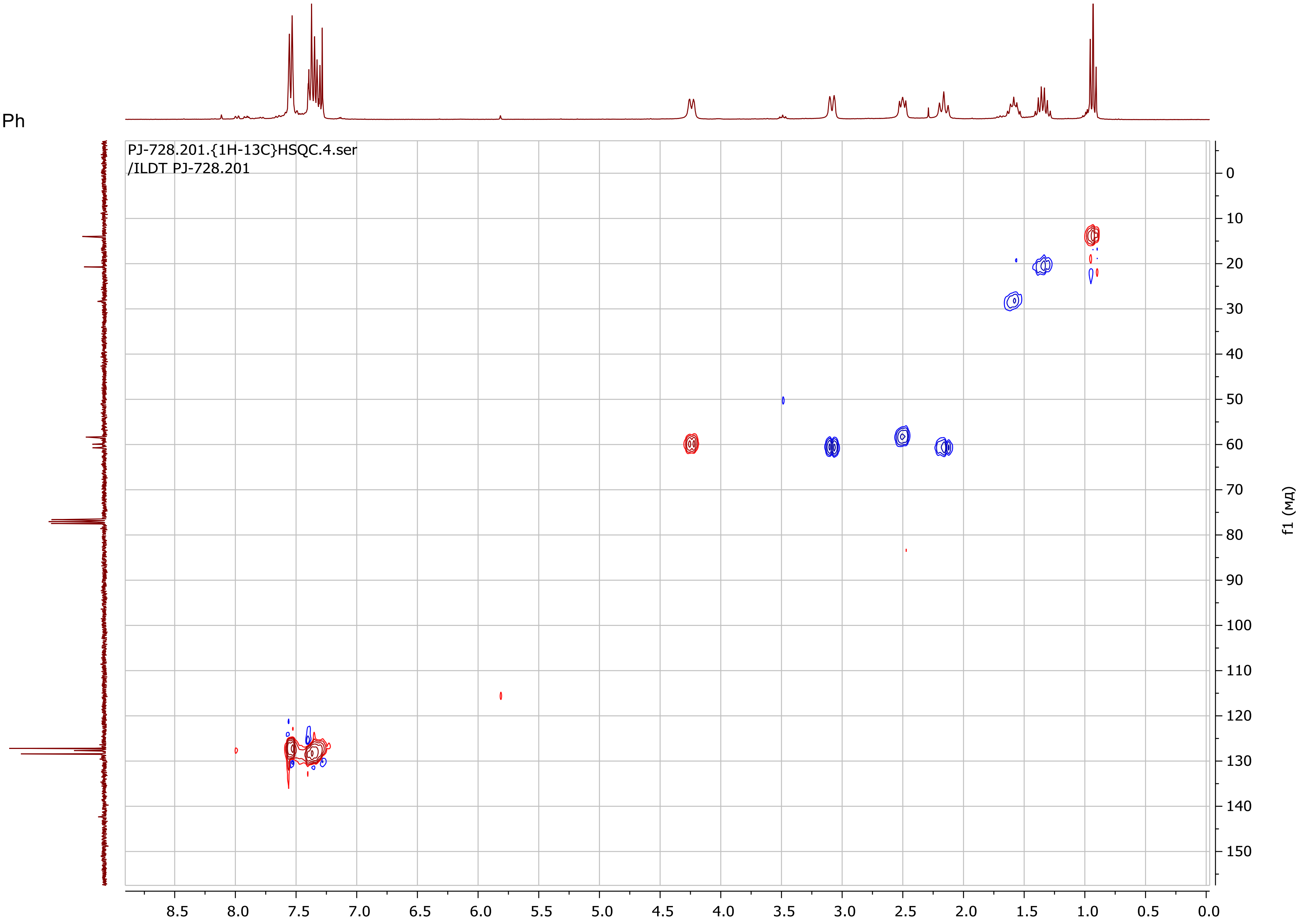

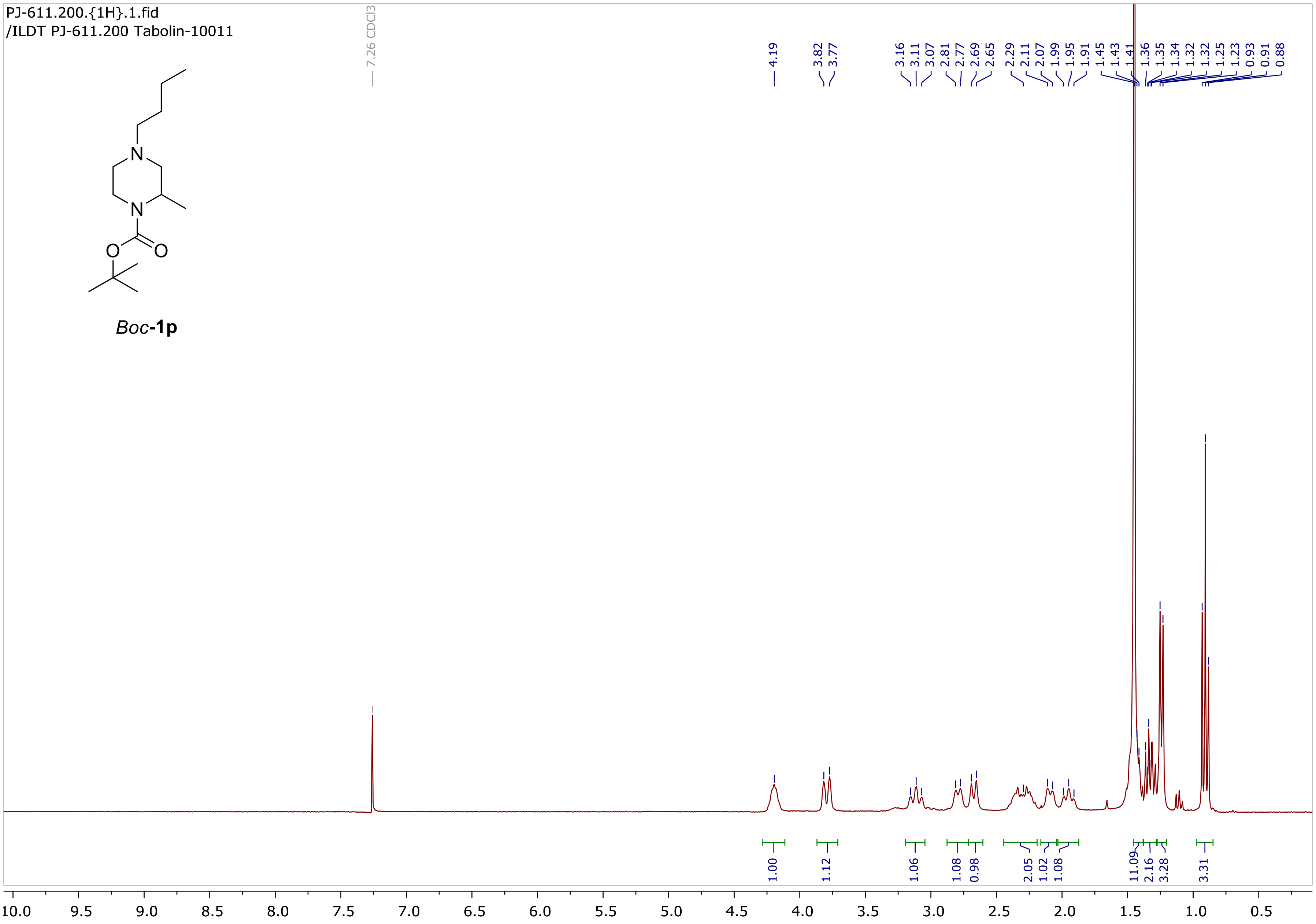
PJ-611.200.{1H}.1.fid
/ILDT PJ-611.200 Tabolin-10011
Boc-1p
7.26 CDCl3
4.19
3.82
3.77
3.16
3.11
3.07
2.81
2.77
2.69
2.65
2.29
2.11
2.07
1.99
1.95
1.91
1.45
1.43
1.41
1.36
1.35
1.34
1.32
1.32
1.25
1.23
0.93
0.91
0.88
1.00
1.12
1.06
1.08
0.98
2.05
1.02
1.08
11.09
2.16
3.28
3.31
10.0
9.5
9.0
8.5
8.0
7.5
7.0
6.5
6.0
5.5
5.0
4.5
4.0
3.5
3.0
2.5
2.0
1.5
1.0
0.5

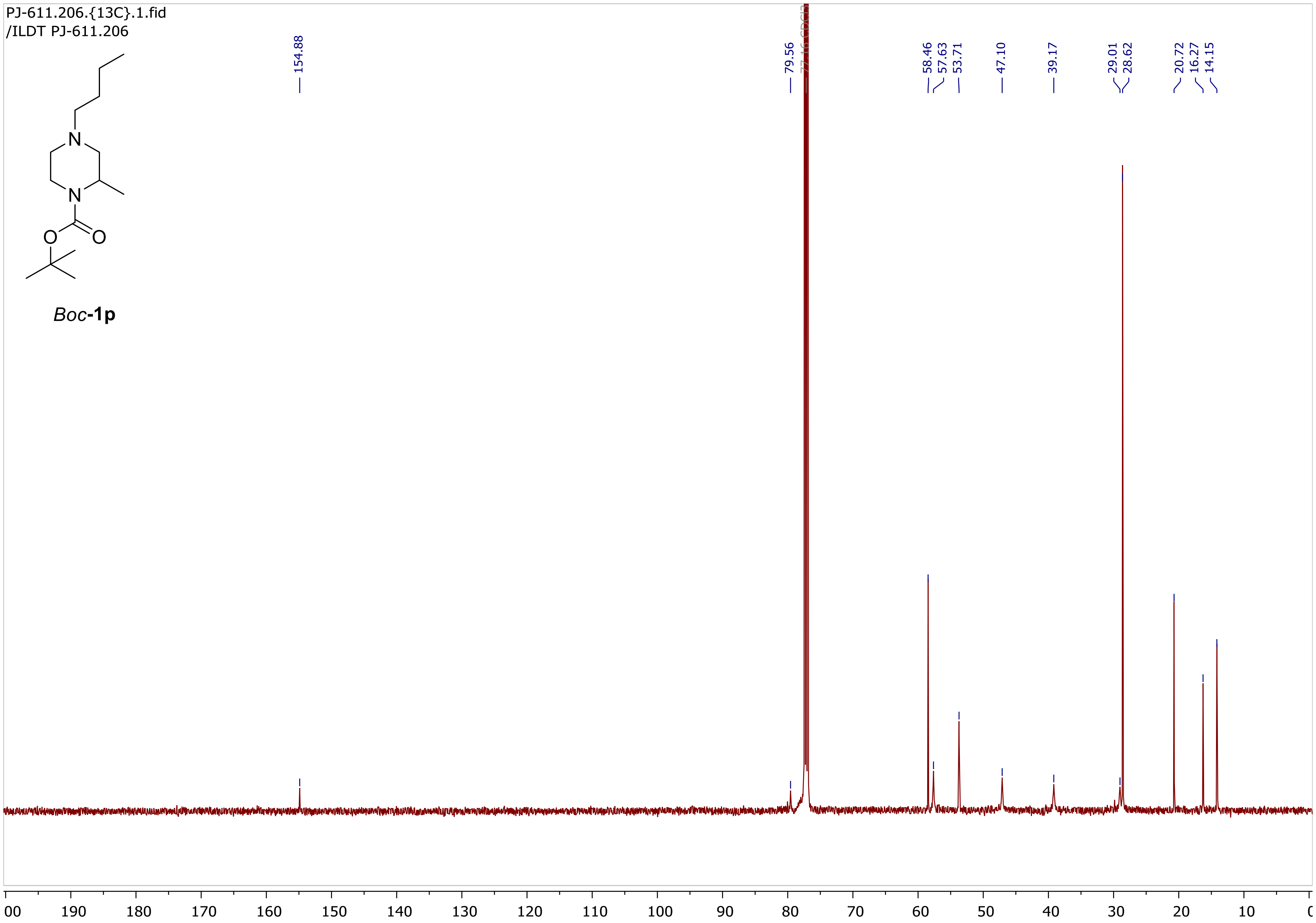

PJ-611.206.{13C}.1.fid
/ILDT PJ-611.206
154.88
79.56
58.46
57.63
53.71
47.10
39.17
29.01
28.62
20.72
16.27
14.15
Boc-1p

PJ-611.100.{13C}deptsp135.3.fid
/ILDT PJ-611.100 Tabolin-10011

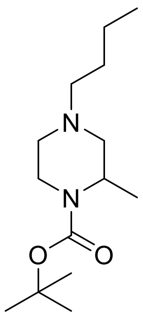

Boc-**1p**

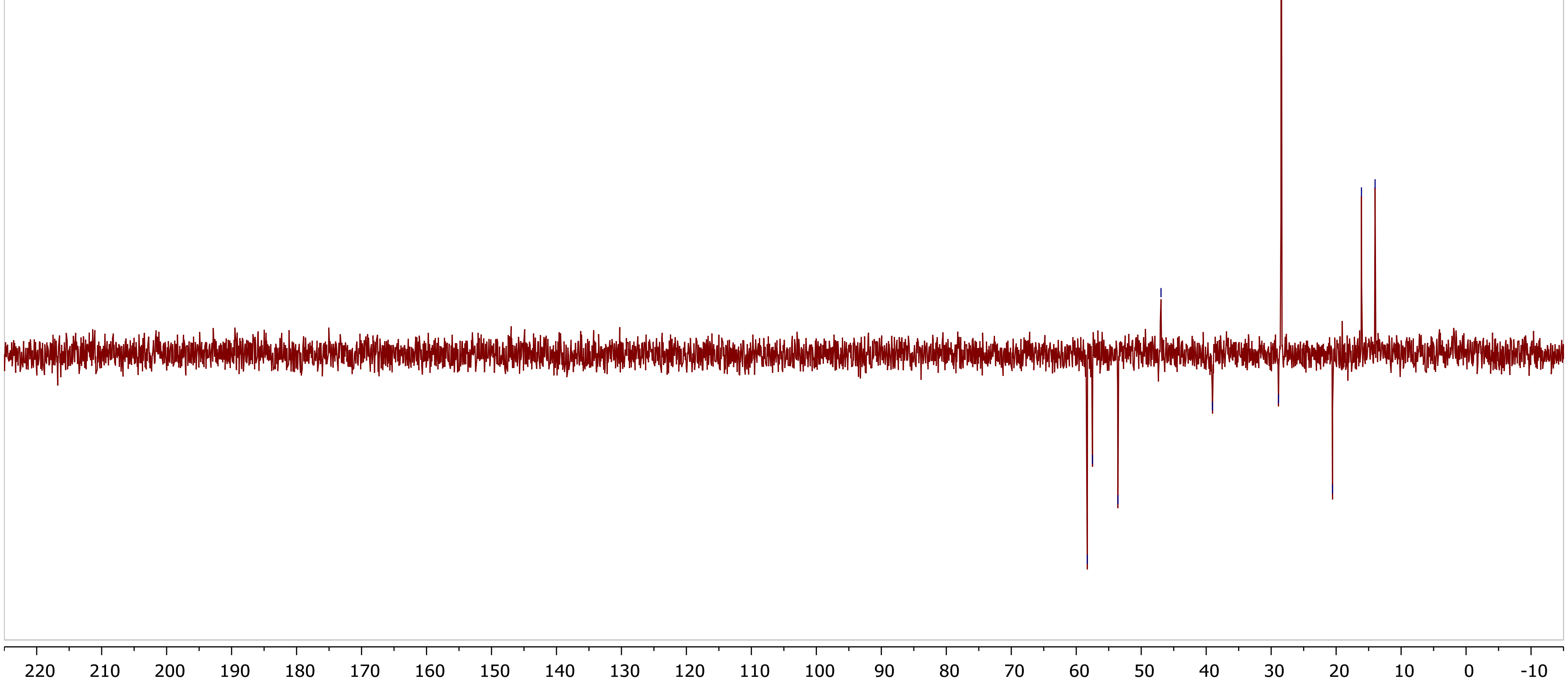

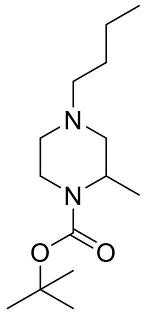
N
N
O
O

Boc-**1p**

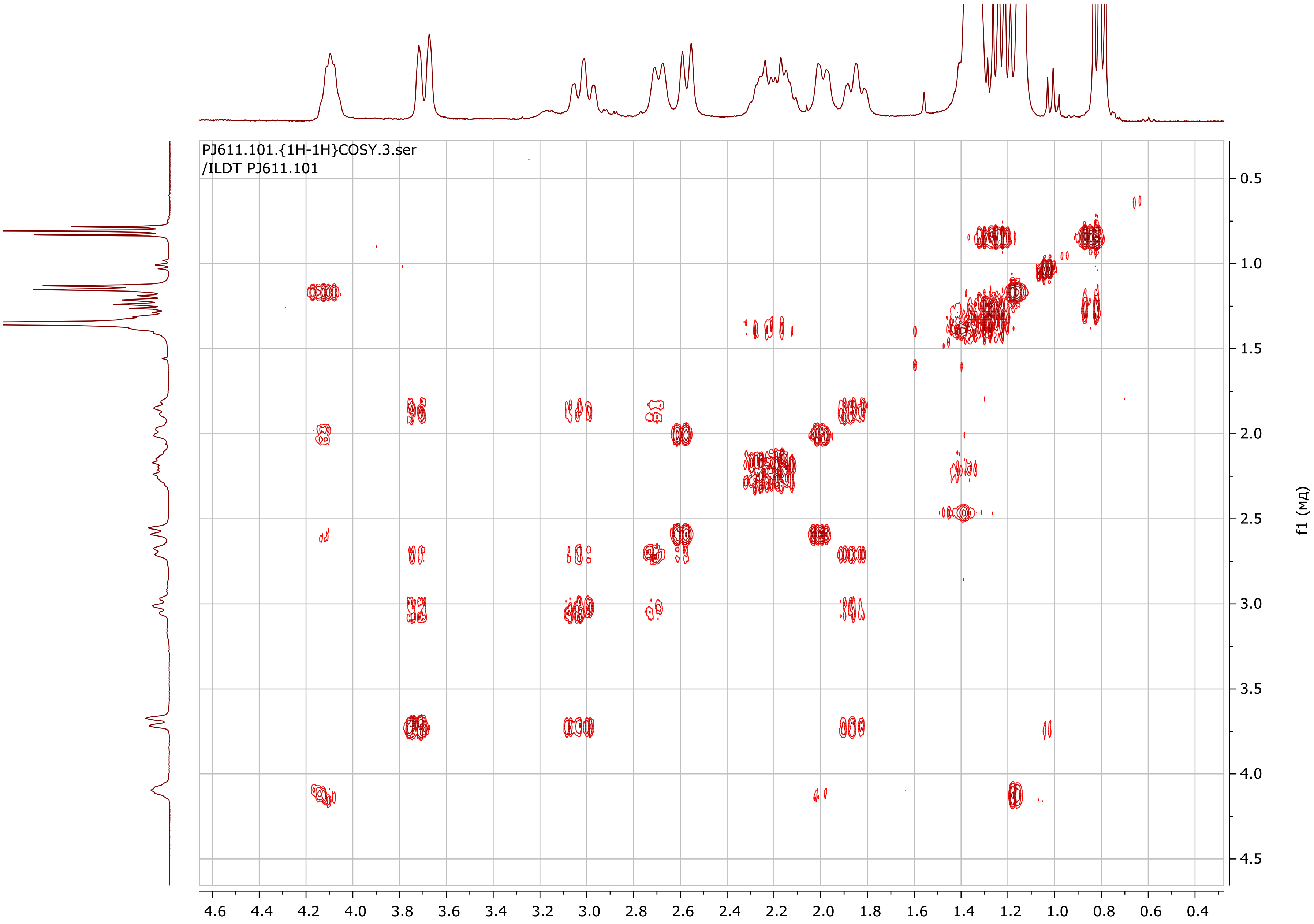
PJ611.101.{1H-1H}COSY.3.ser
/ILDT PJ611.101
f1 (мд)
4.6
4.4
4.2
4.0
3.8
3.6
3.4
3.2
3.0
2.8
2.6
2.4
2.2
2.0
1.8
1.6
1.4
1.2
1.0
0.8
0.6
0.4
0.5
1.0
1.5
2.0
2.5
3.0
3.5
4.0
4.5

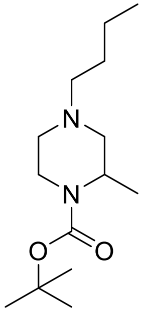

Boc-**1p**

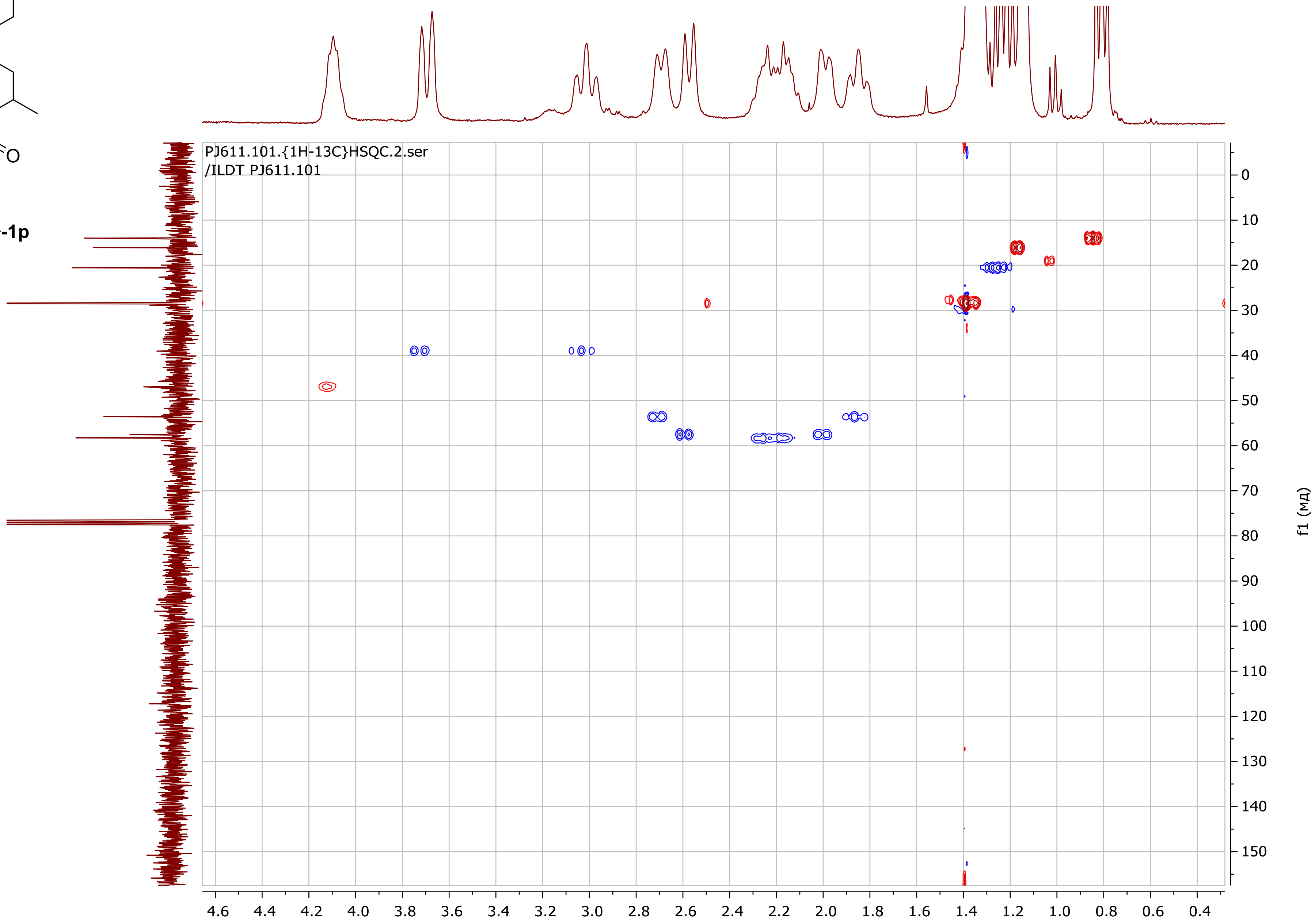

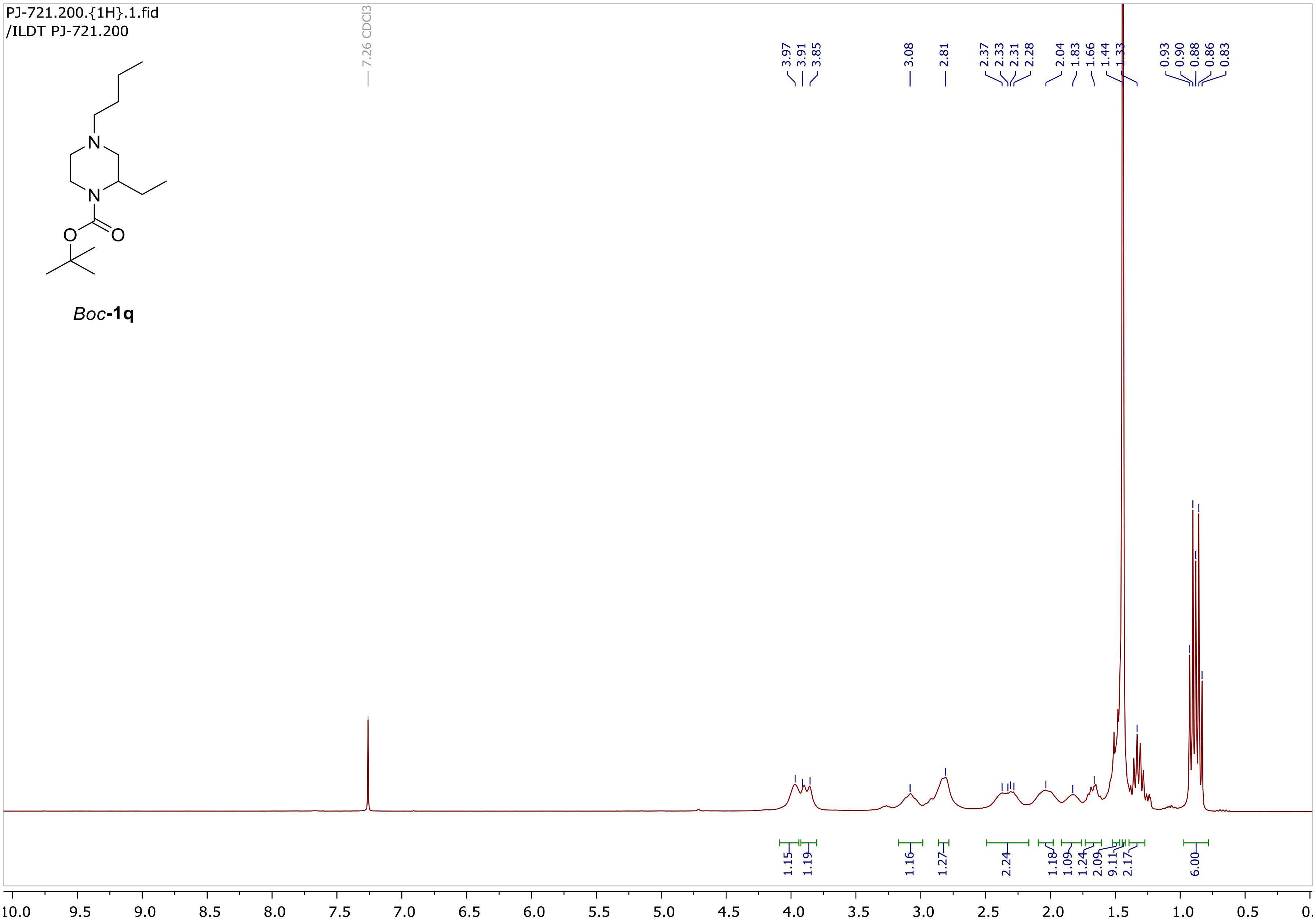
PJ-721.200.{1H}.1.fid
/ILDT PJ-721.200
7.26 CDCl3
3.97
3.91
3.85
3.08
2.81
2.37
2.33
2.31
2.28
2.04
1.83
1.66
1.44
1.33
0.93
0.90
0.88
0.86
0.83
O
O
N
N
Boc-1q
1.15
1.19
1.16
1.27
2.24
1.18
1.09
1.24
2.09
9.11
2.17
6.00
10.0 9.5 9.0 8.5 8.0 7.5 7.0 6.5 6.0 5.5 5.0 4.5 4.0 3.5 3.0 2.5 2.0 1.5 1.0 0.5

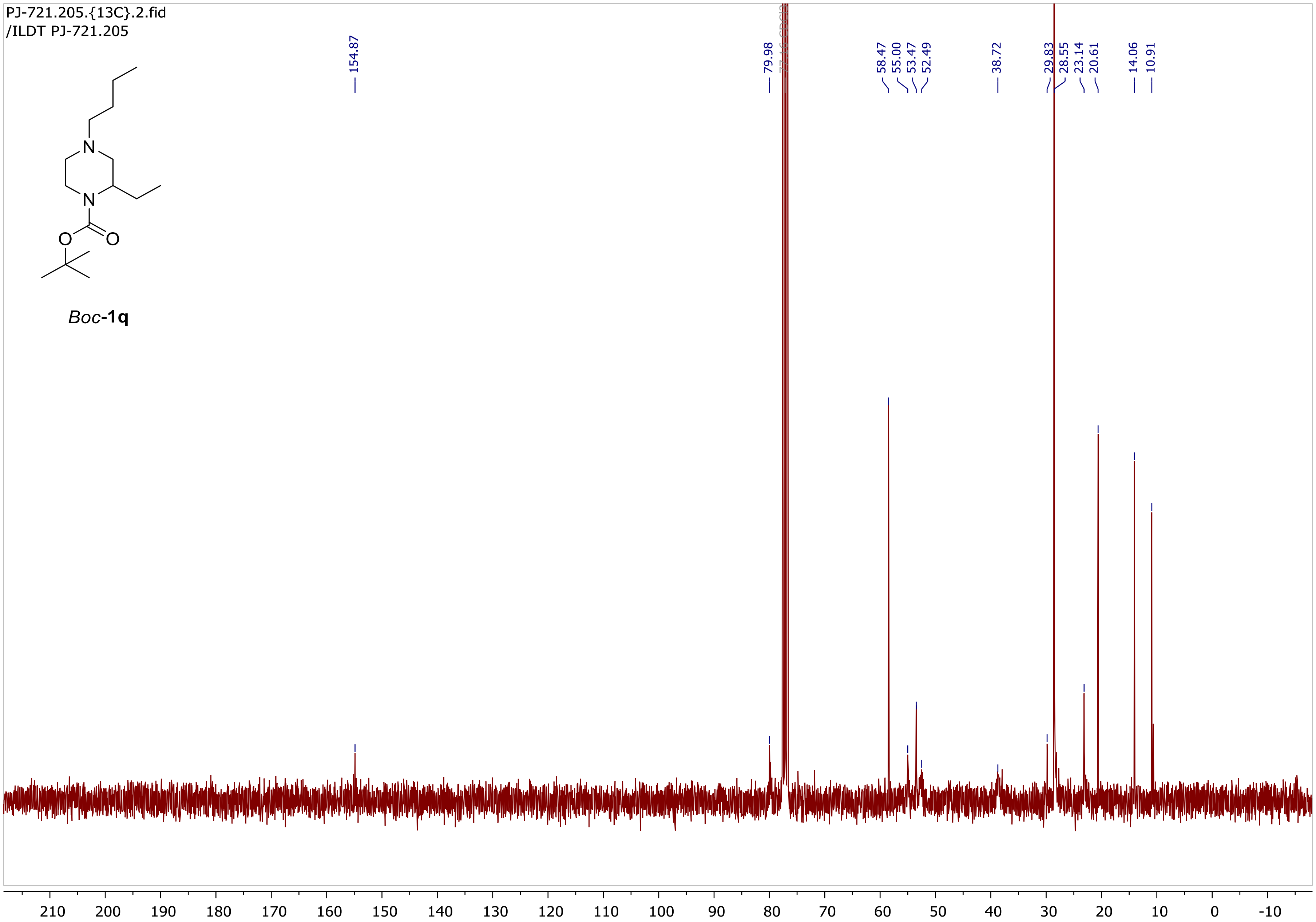
PJ-721.205.{13C}.2.fid
/ILDT PJ-721.205
154.87
79.98
58.47
55.00
53.47
52.49
38.72
29.83
28.55
23.14
20.61
14.06
10.91
Boc-1q
210 200 190 180 170 160 150 140 130 120 110 100 90 80 70 60 50 40 30 20 10 0 -10

PJ-721.200.{13C}deptsp135.3.fid
/ILDT PJ-721.200

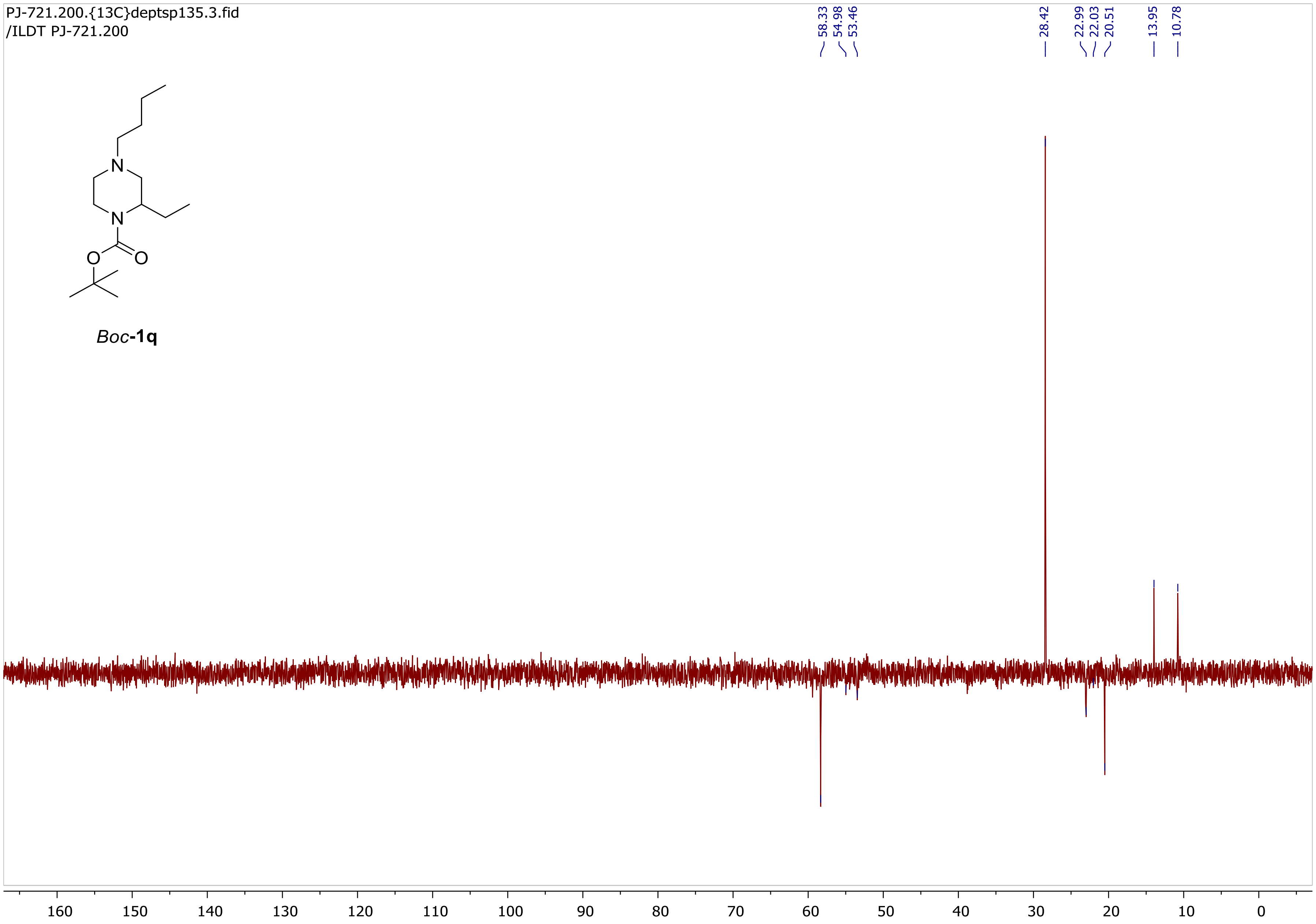

Boc-**1q**

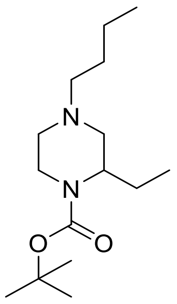

Boc-**1q**

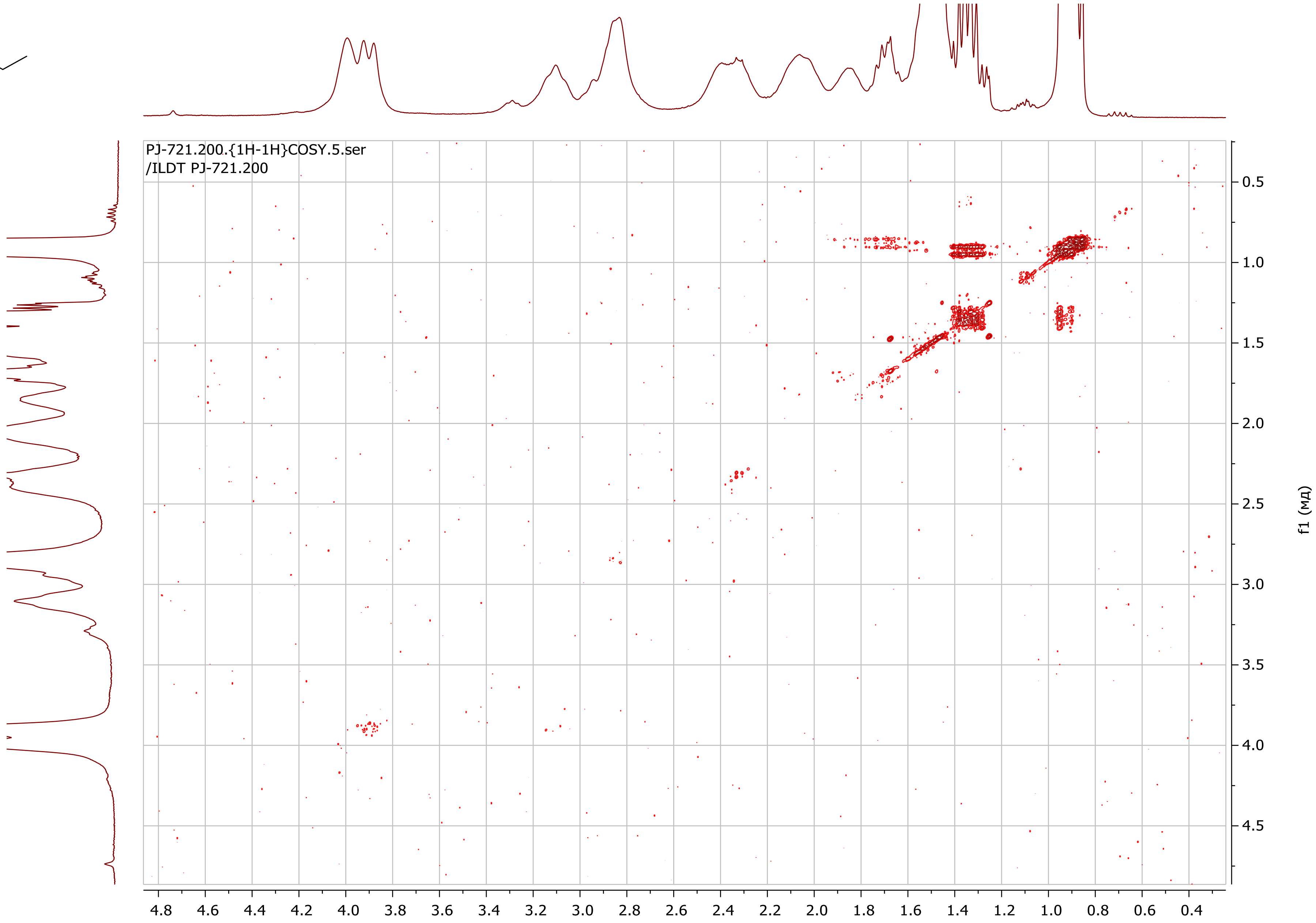

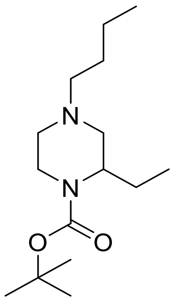

Boc-**1q**

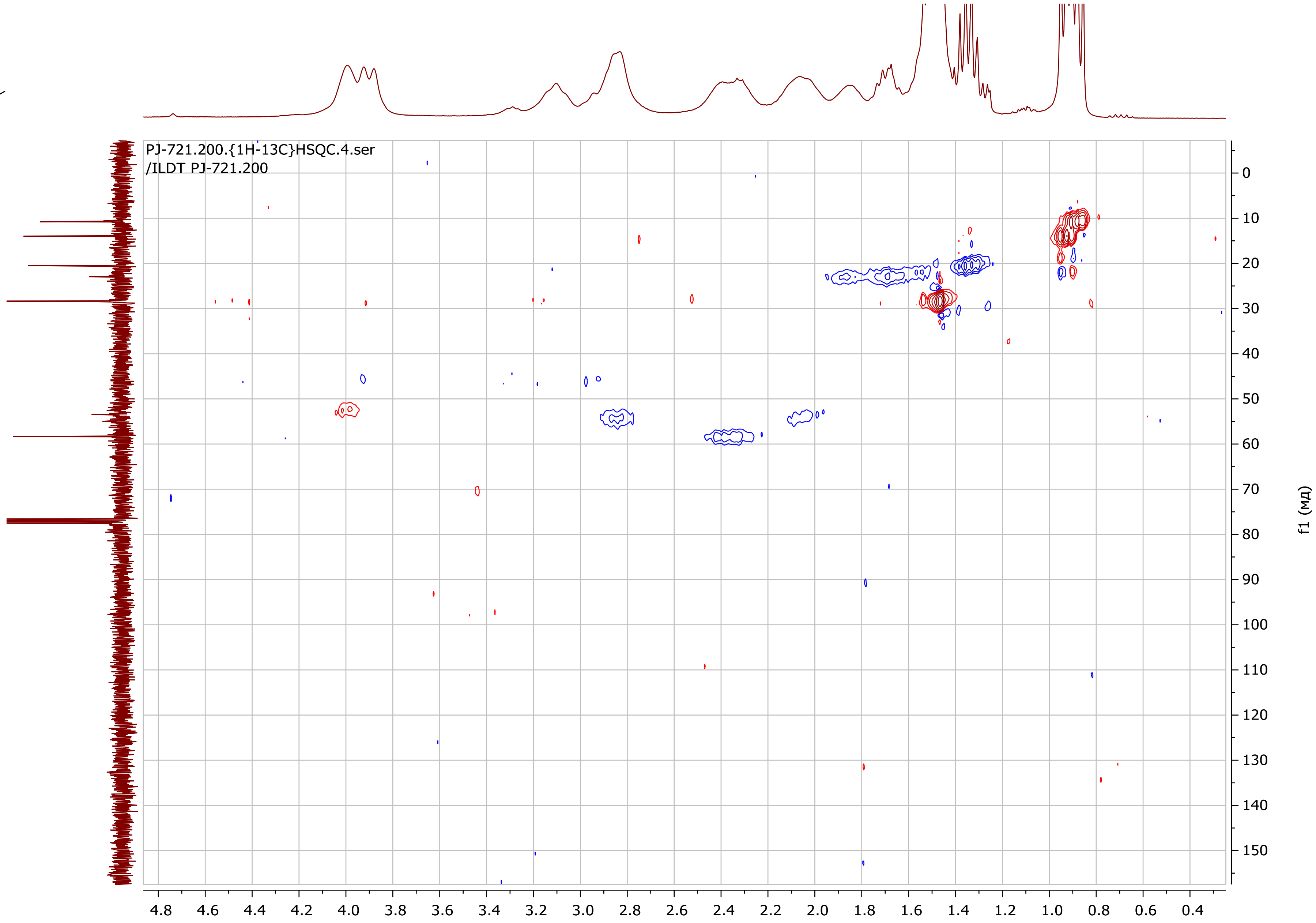
PJ-721.200.{1H-13C}HSQC.4.ser
/ILDT PJ-721.200
f1 (мд)
4.8 4.6 4.4 4.2 4.0 3.8 3.6 3.4 3.2 3.0 2.8 2.6 2.4 2.2 2.0 1.8 1.6 1.4 1.2 1.0 0.8 0.6 0.4
0 10 20 30 40 50 60 70 80 90 100 110 120 130 140 150

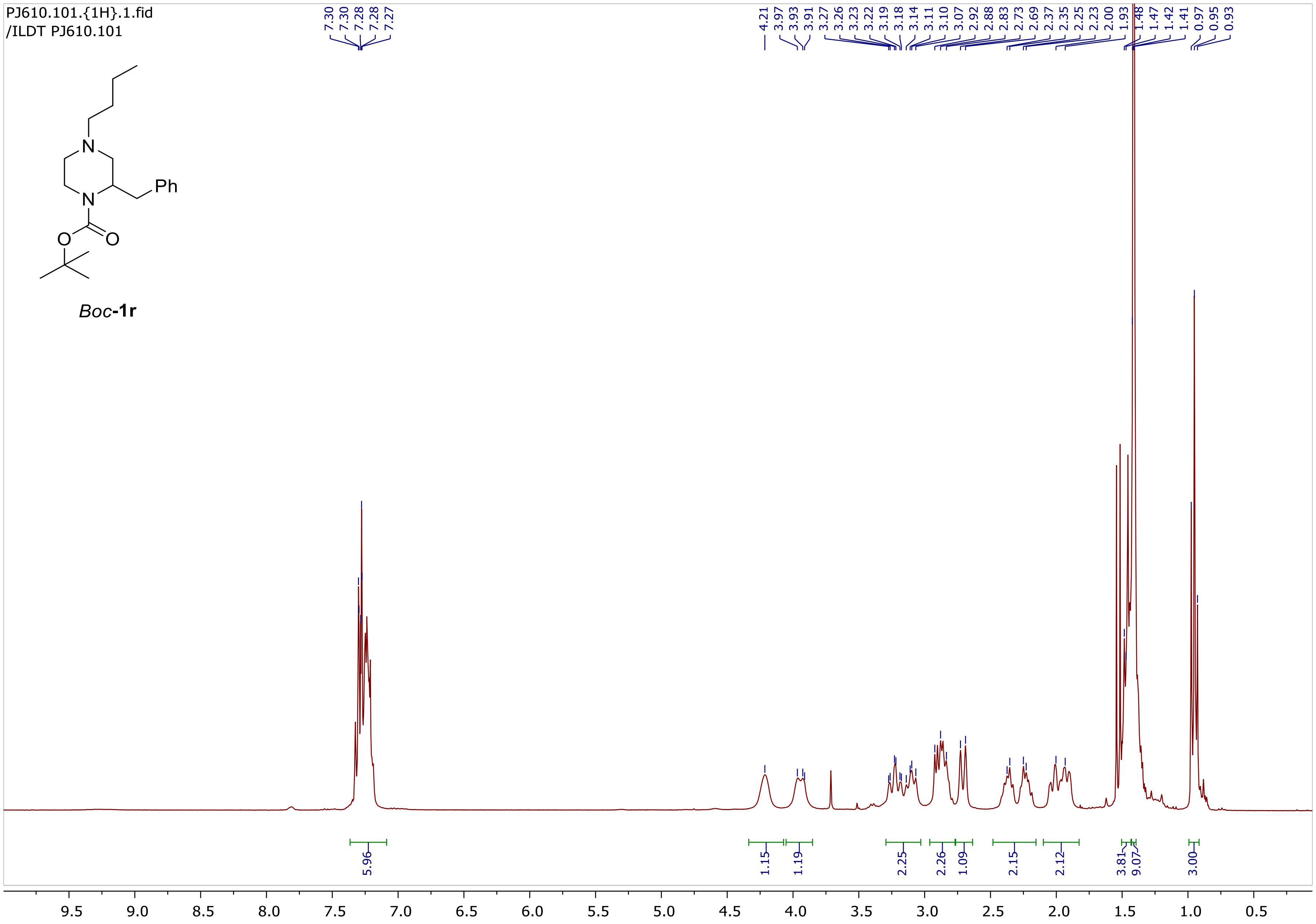
PJ610.101.{1H}.1.fid
/ILDT PJ610.101
7.30 7.30 7.28 7.28 7.27
4.21 3.97 3.93 3.91 3.27 3.26 3.23 3.22 3.19 3.18 3.14 3.11 3.10 3.07 2.92 2.88 2.83 2.73 2.69 2.37 2.35 2.25 2.23 2.00 1.93 1.48 1.47 1.42 1.41 0.97 0.95 0.93
N
N
Ph
O
O
Boc-1r
5.96
1.15
1.19
2.25
2.26
1.09
2.15
2.12
3.81
9.07
3.00
9.5 9.0 8.5 8.0 7.5 7.0 6.5 6.0 5.5 5.0 4.5 4.0 3.5 3.0 2.5 2.0 1.5 1.0 0.5

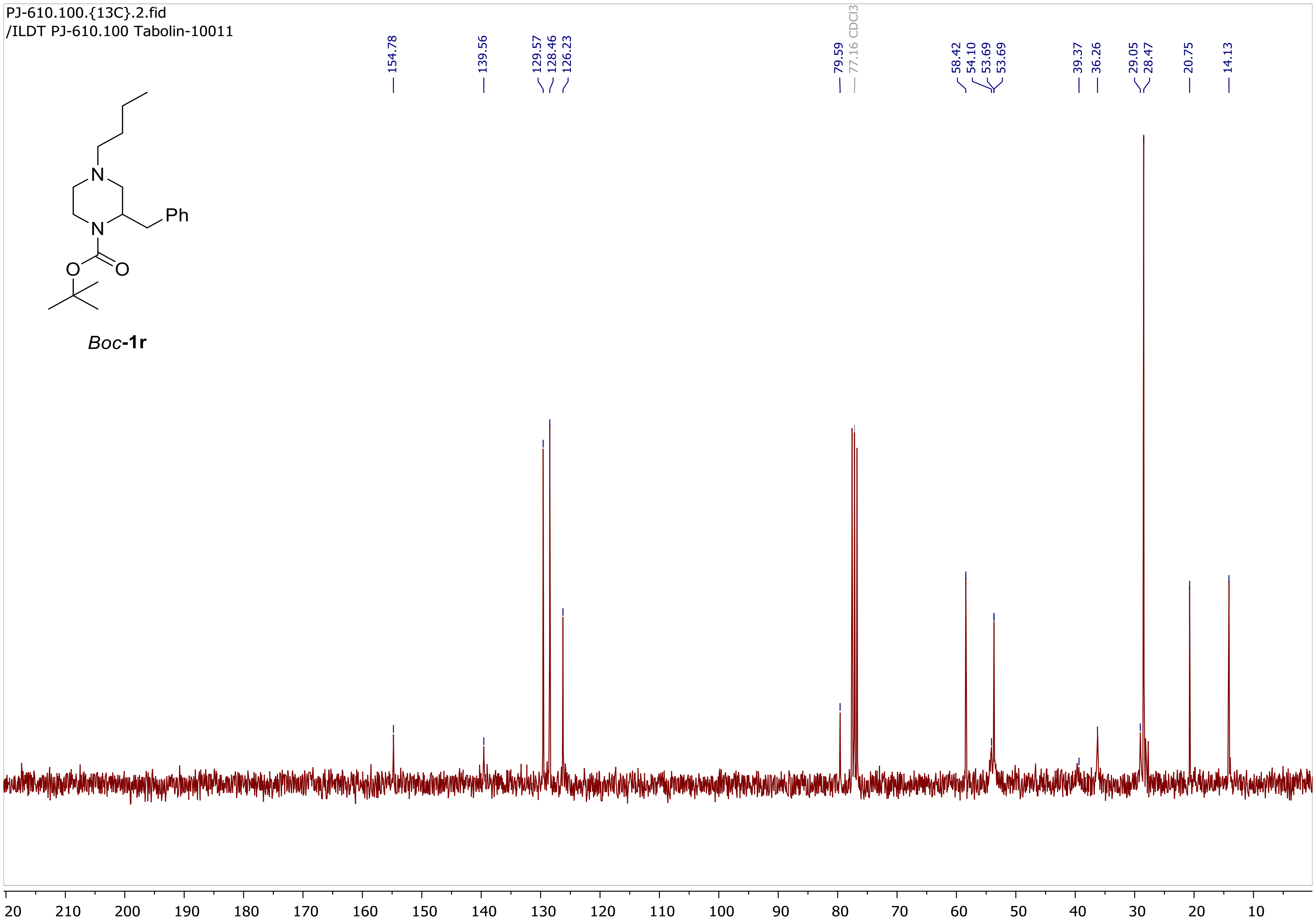
PJ-610.100.{13C}.2.fid
/ILDT PJ-610.100 Tabolin-10011
154.78
139.56
129.57
128.46
126.23
79.59
77.16 CDCl3
58.42
54.10
53.69
53.69
39.37
36.26
29.05
28.47
20.75
14.13
N
N
Ph
O
O
Boc-**1r**
20
210
200
190
180
170
160
150
140
130
120
110
100
90
80
70
60
50
40
30
20
10

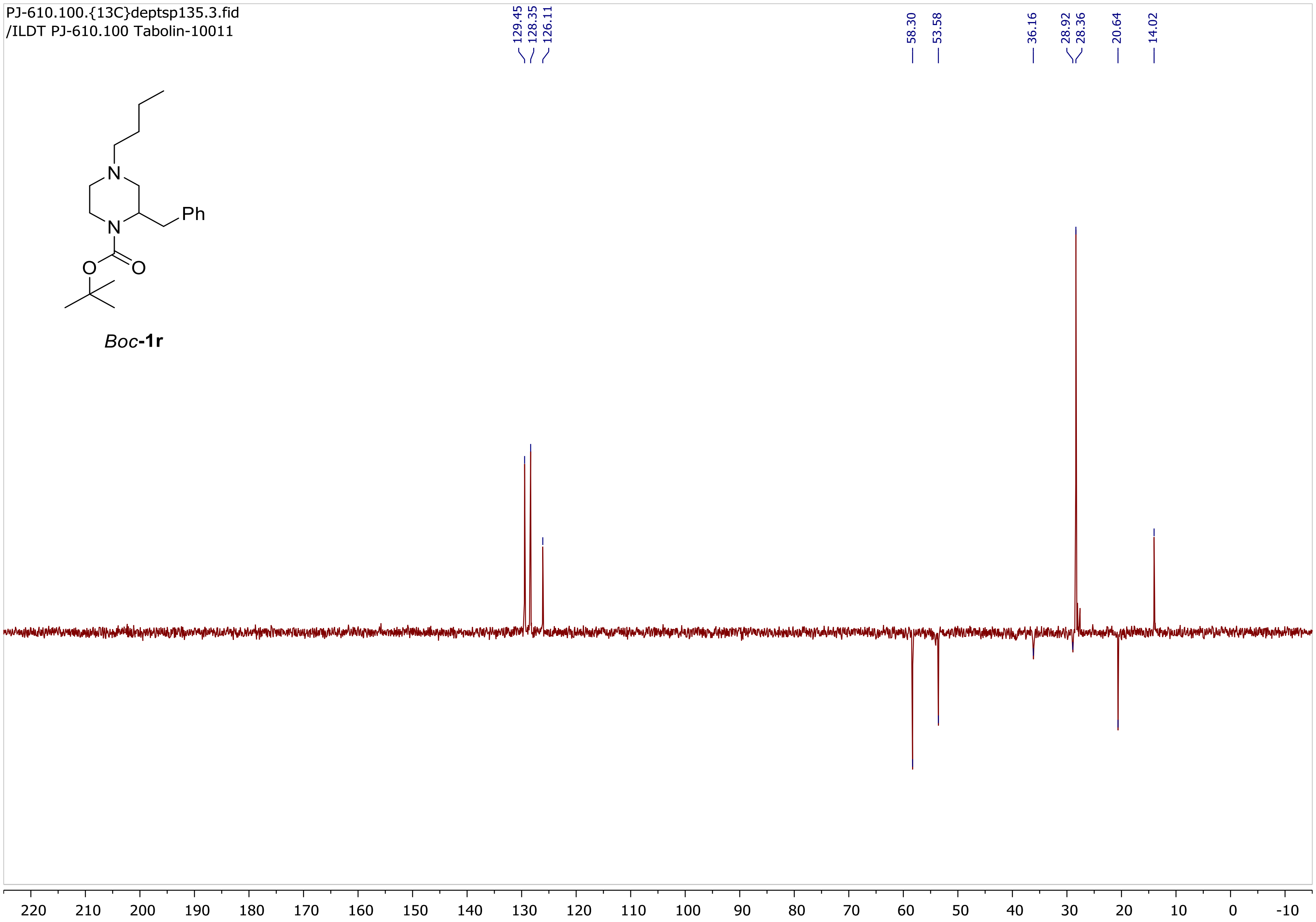
PJ-610.100.{13C}deptsp135.3.fid
/ILDT PJ-610.100 Tabolin-10011
129.45
128.35
126.11
58.30
53.58
36.16
28.92
28.36
20.64
14.02
N
N
Ph
O
O
Boc-**1r**
220 210 200 190 180 170 160 150 140 130 120 110 100 90 80 70 60 50 40 30 20 10 0 -10

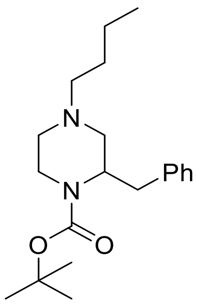

Boc-**1r**

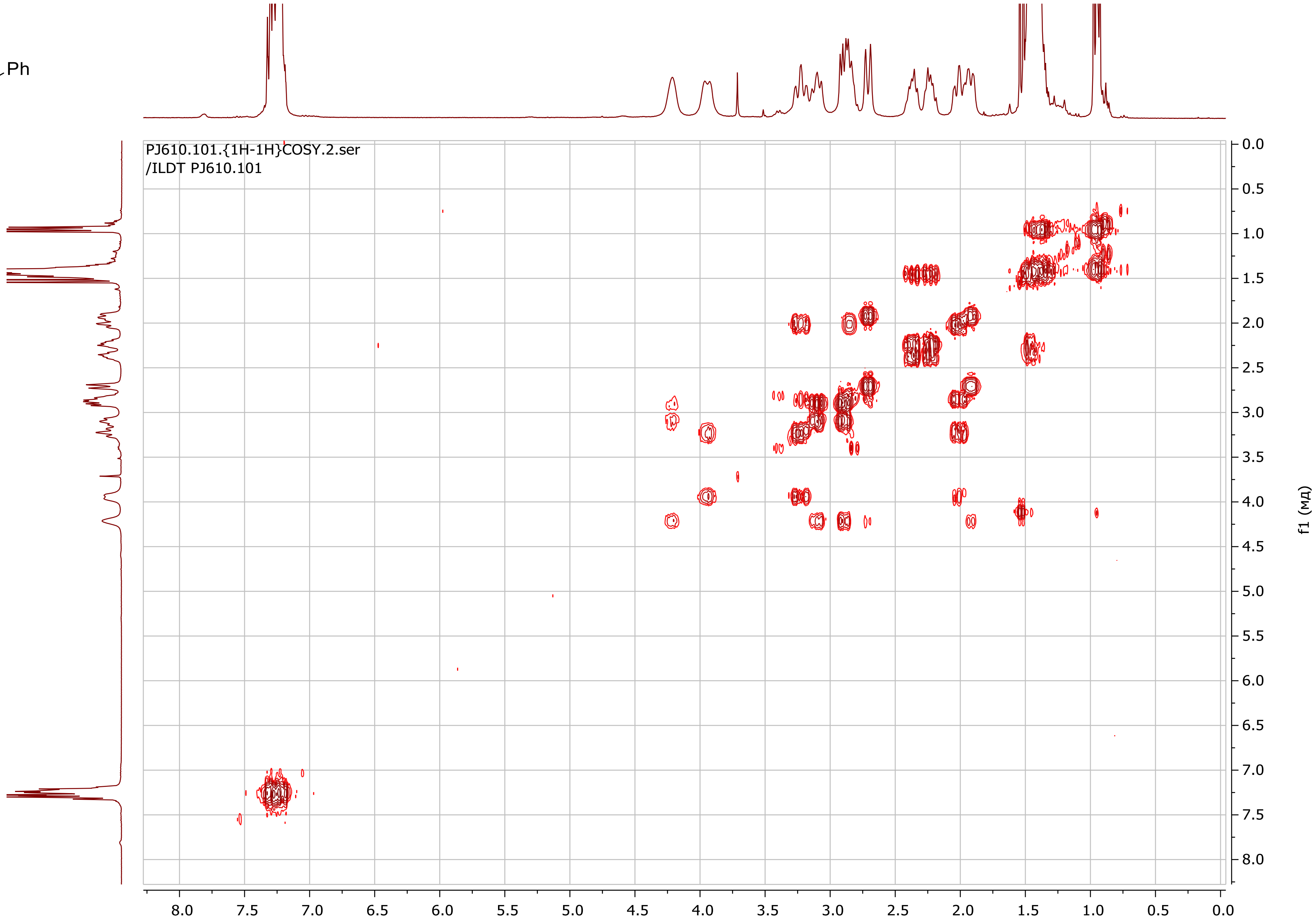

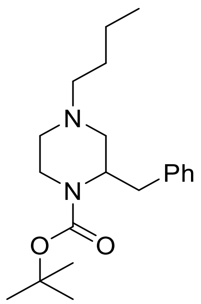

Boc-**1r**

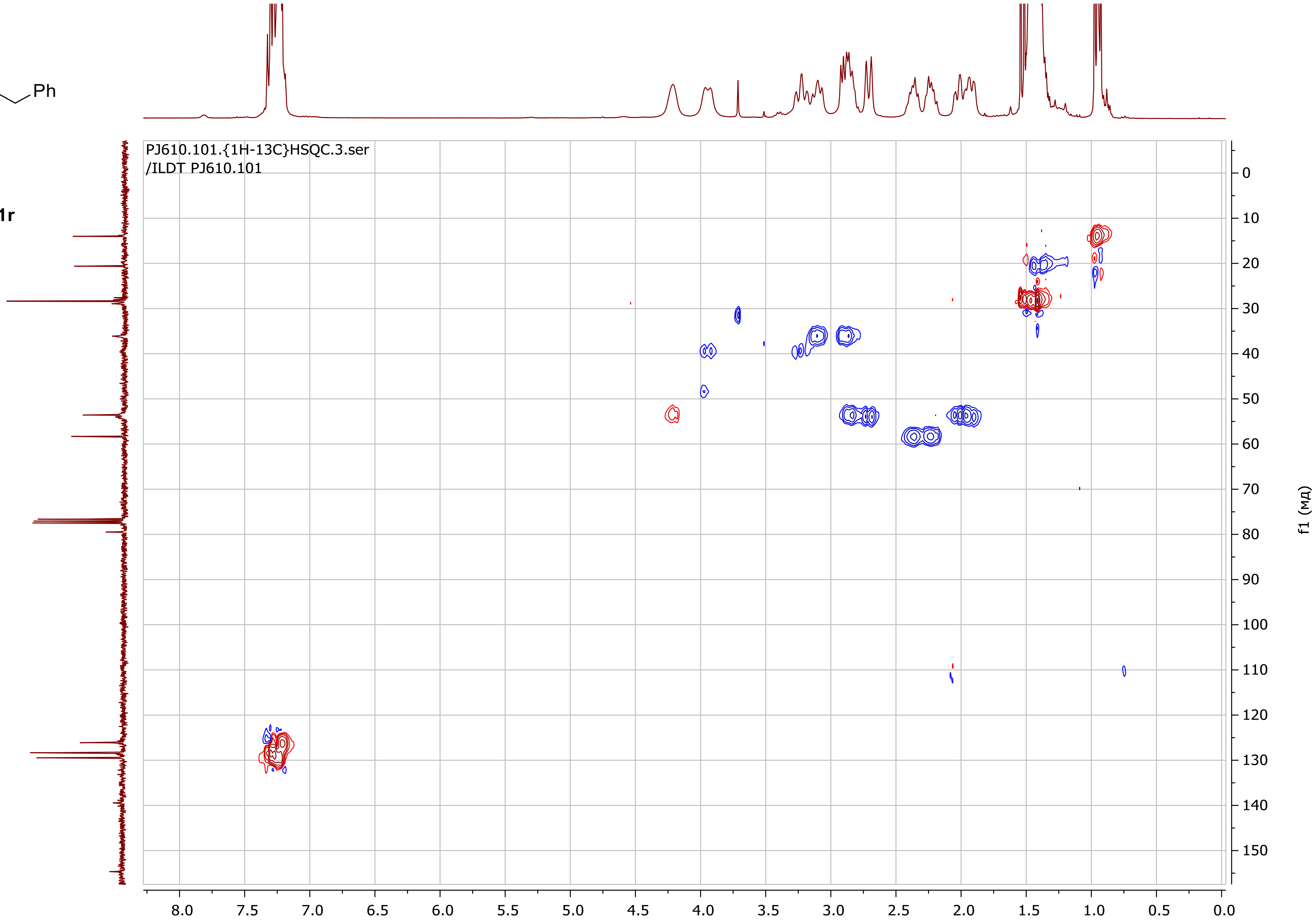

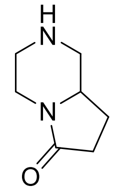

5

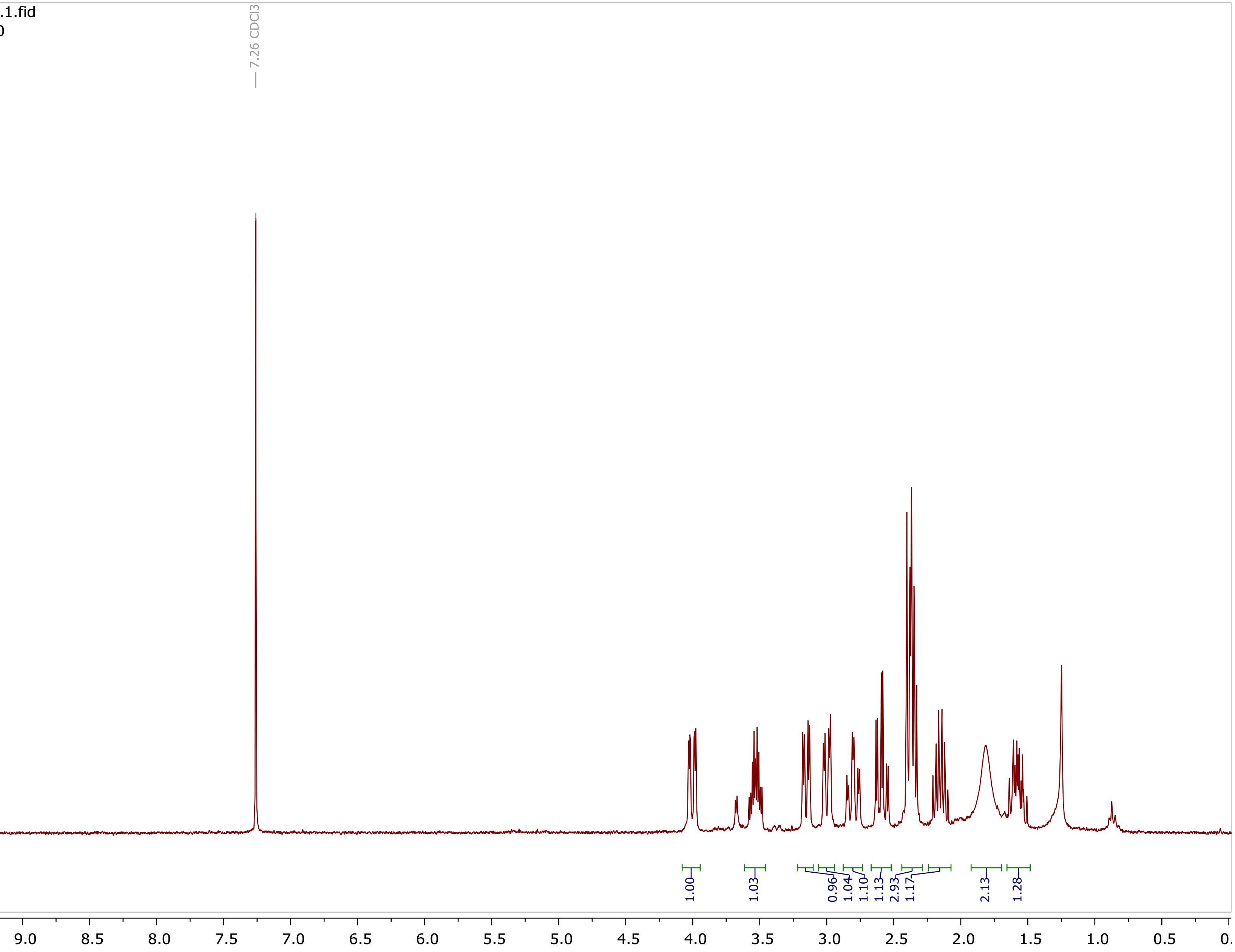

PJ615.100.{1H}.1.fid
/ILDT PJ615.100
7.26 CDCl3
1.00
1.03
0.96
1.04
1.10
1.13
2.93
1.17
2.13
1.28
10.0 9.5 9.0 8.5 8.0 7.5 7.0 6.5 6.0 5.5 5.0 4.5 4.0 3.5 3.0 2.5 2.0 1.5 1.0 0.5 0.

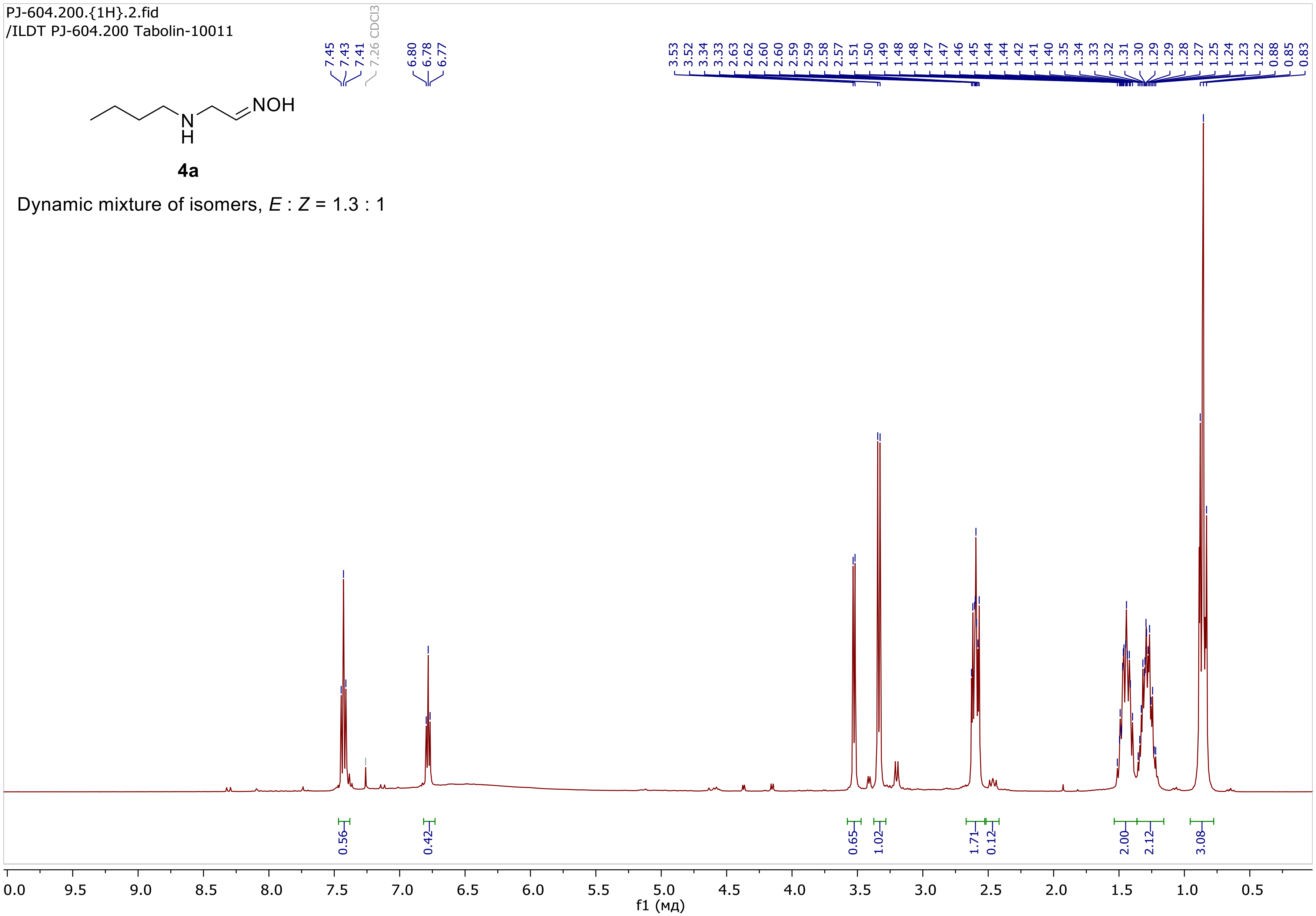
PJ-604.200.{1H}.2.fid
/ILDT PJ-604.200 Tabolin-10011
NOH
N
H
4a
Dynamic mixture of isomers, E : Z = 1.3 : 1
f1 (мд)

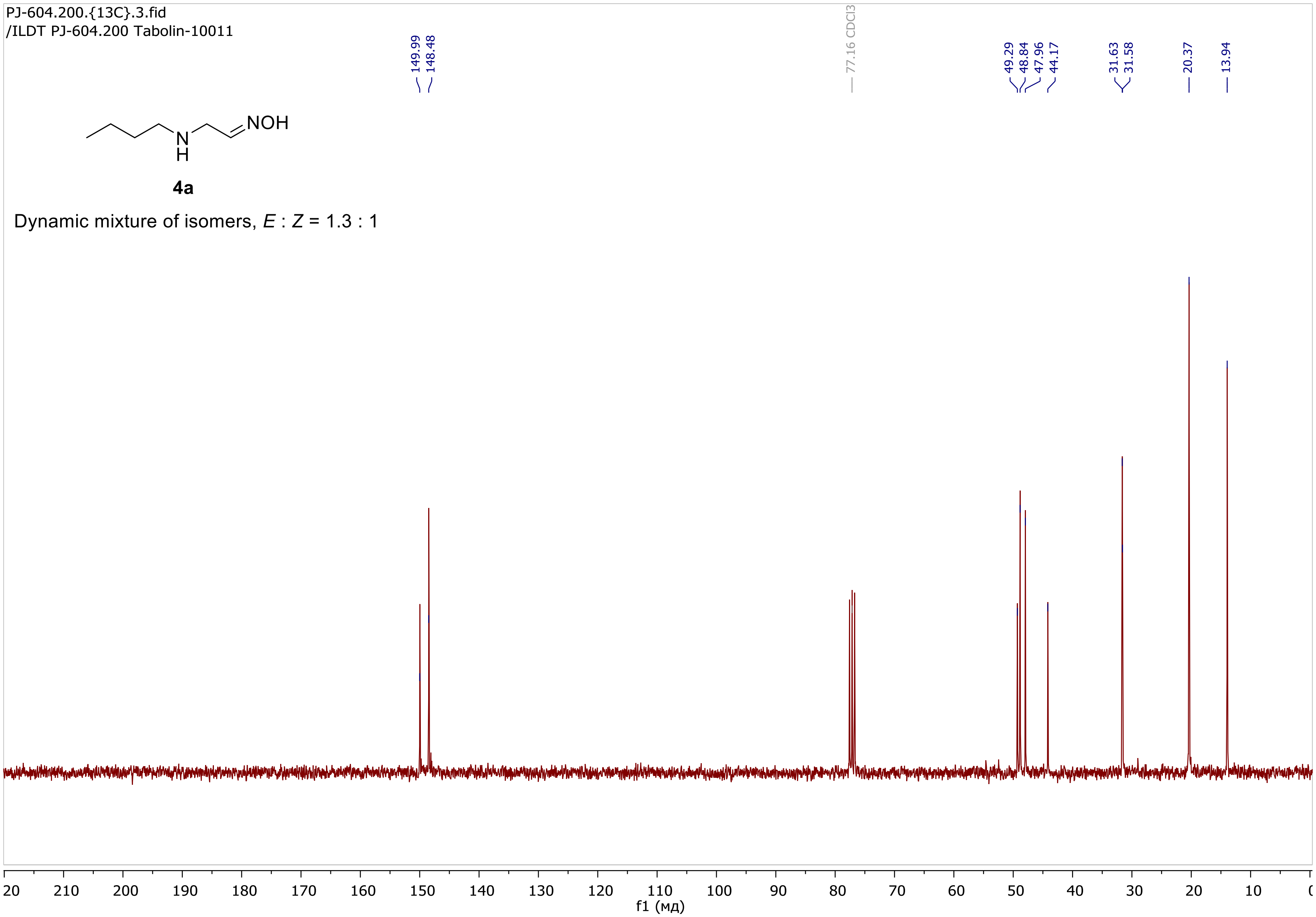
PJ-604.200.{13C}.3.fid
/ILDT PJ-604.200 Tabolin-10011
NOH
N
H
4a
Dynamic mixture of isomers, E : Z = 1.3 : 1
149.99
148.48
77.16 CDCl3
49.29
48.84
47.96
44.17
31.63
31.58
20.37
13.94
f1 (мд)

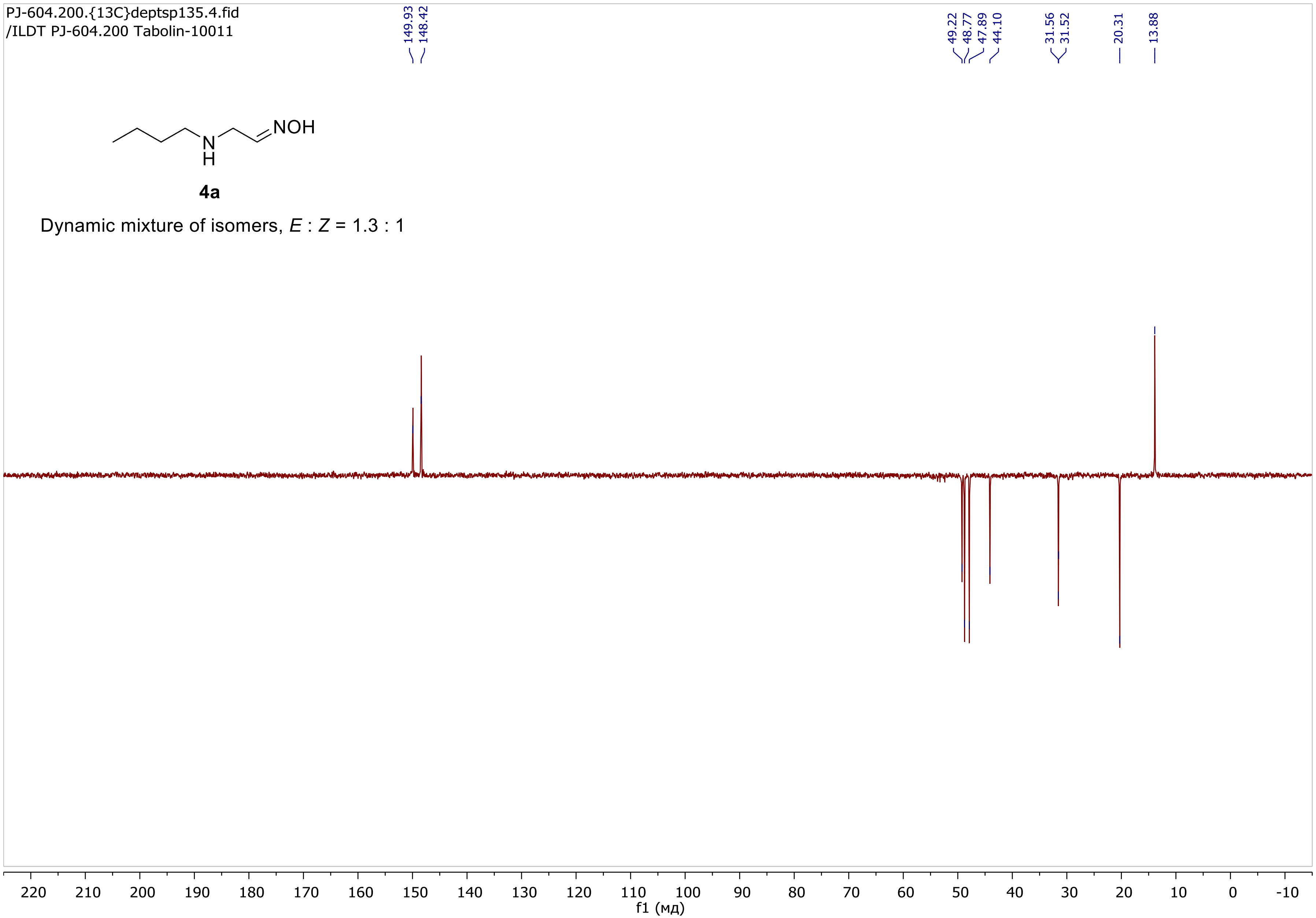
PJ-604.200.{13C}deptsp135.4.fid
/ILDT PJ-604.200 Tabolin-10011
149.93
148.42
49.22
48.77
47.89
44.10
31.56
31.52
20.31
13.88
NOH
N
H
4a
Dynamic mixture of isomers, E : Z = 1.3 : 1
220 210 200 190 180 170 160 150 140 130 120 110 100 90 80 70 60 50 40 30 20 10 0 -10
f1 (мд)

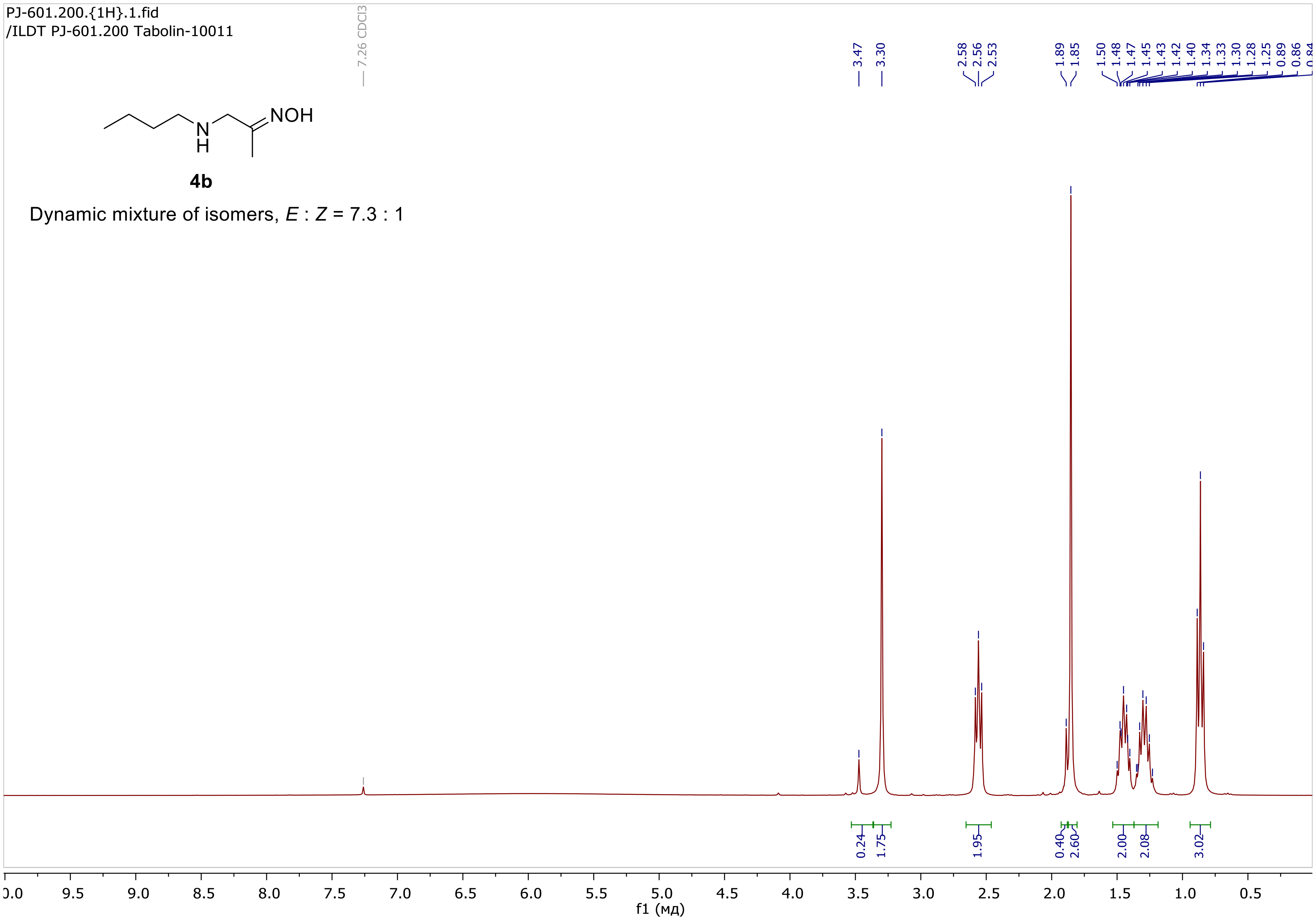

PJ-601.200.{1H}.1.fid
/ILDT PJ-601.200 Tabolin-10011
7.26 CDCl3
NOH
N
H
4b
Dynamic mixture of isomers, E : Z = 7.3 : 1
3.47
3.30
2.58
2.56
2.53
1.89
1.85
1.50
1.48
1.47
1.45
1.43
1.42
1.40
1.34
1.33
1.30
1.28
1.25
0.89
0.86
0.24
1.75
1.95
0.40
2.60
2.00
2.08
3.02
9.5
9.0
8.5
8.0
7.5
7.0
6.5
6.0
5.5
5.0
4.5
4.0
3.5
3.0
2.5
2.0
1.5
1.0
0.5
f1 (мд)

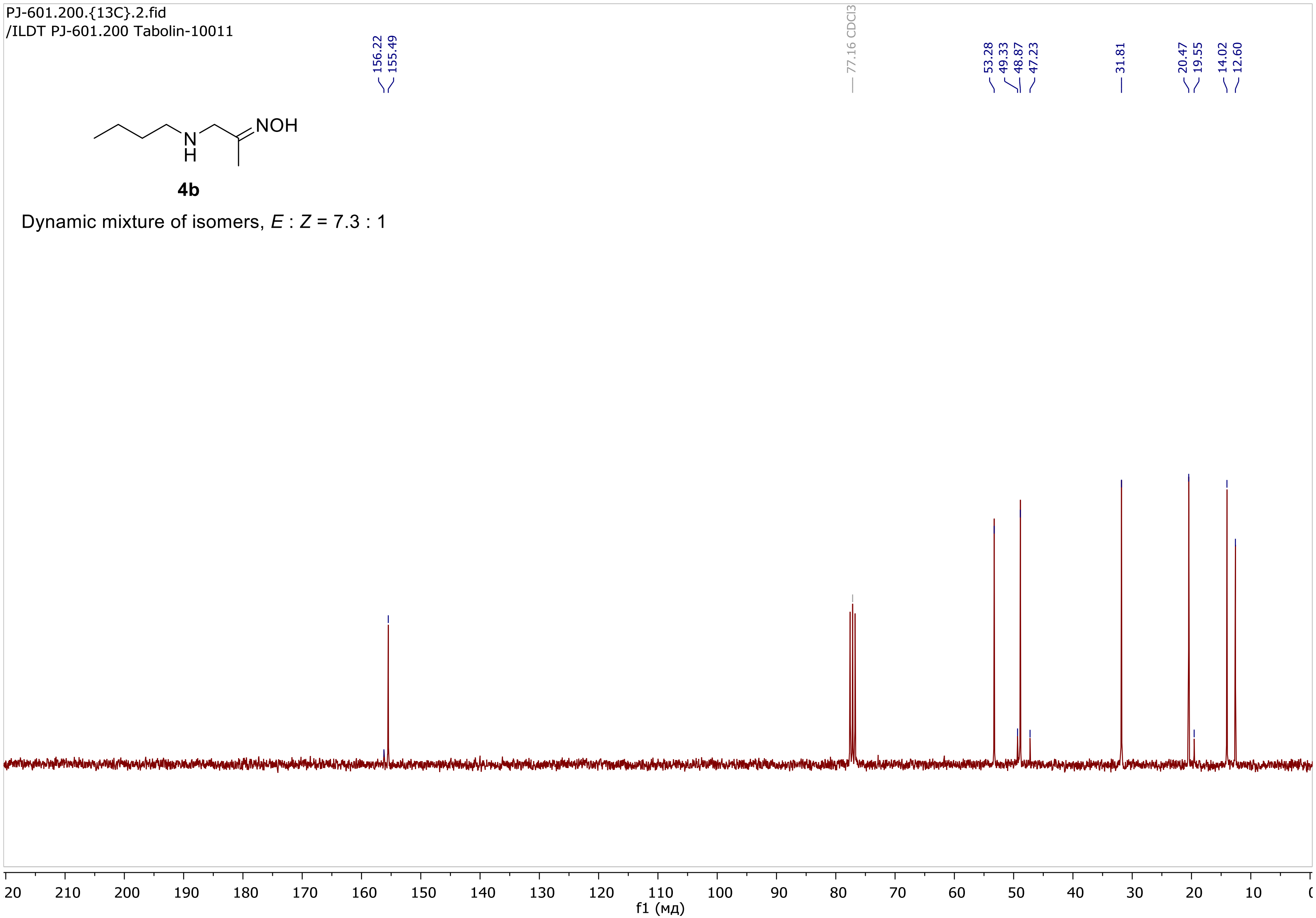
PJ-601.200.{13C}.2.fid
/ILDT PJ-601.200 Tabolin-10011
156.22
155.49
77.16 CDCl3
53.28
49.33
48.87
47.23
31.81
20.47
19.55
14.02
12.60
NOH
N
H
4b
Dynamic mixture of isomers, E : Z = 7.3 : 1
f1 (мд)

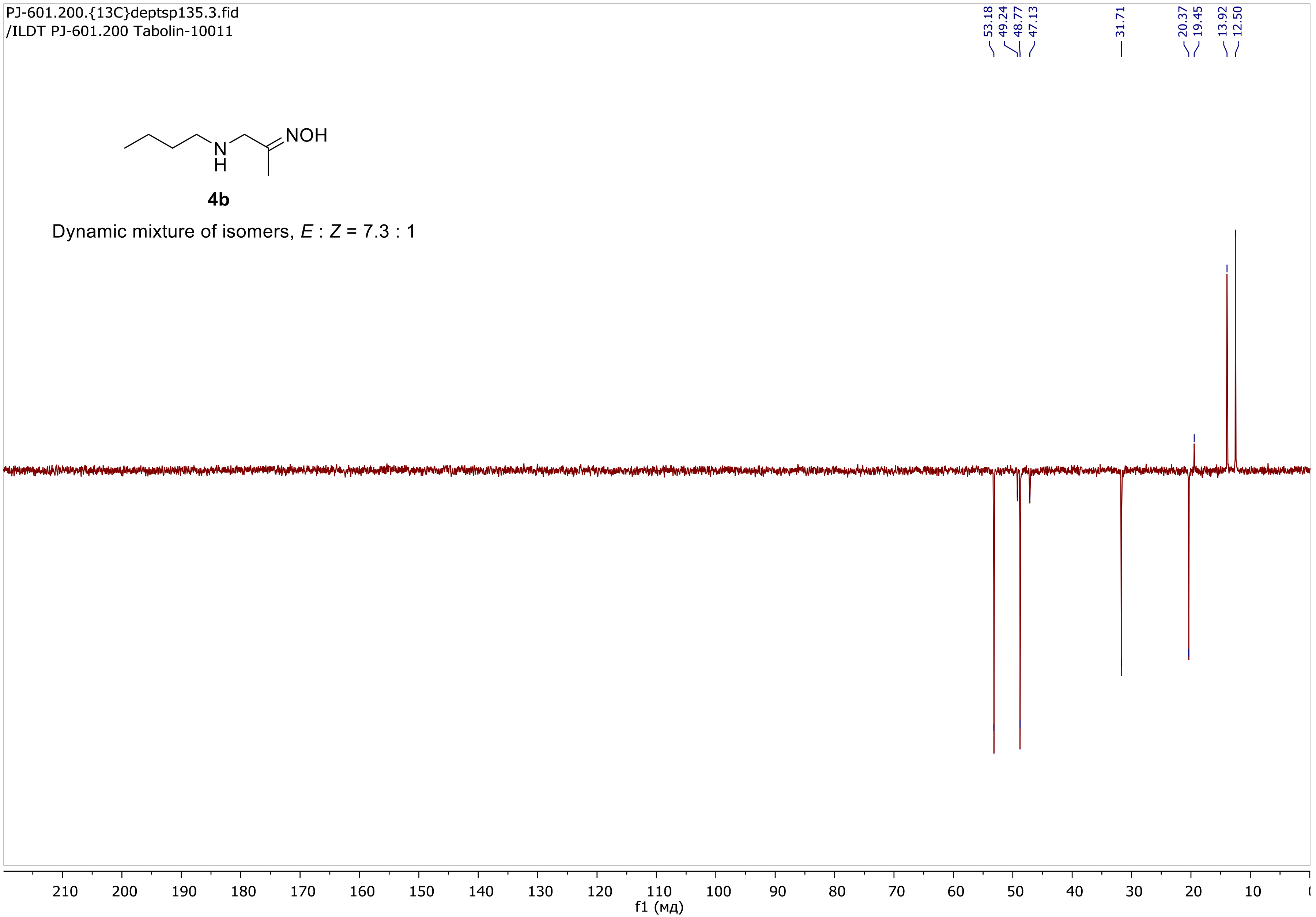

4b

Dynamic mixture of isomers, *E* : *Z* = 7.3 : 1

PJ-609.200.{1H}.1.fid
/ILDT PJ-609.200 Tabolin-10011

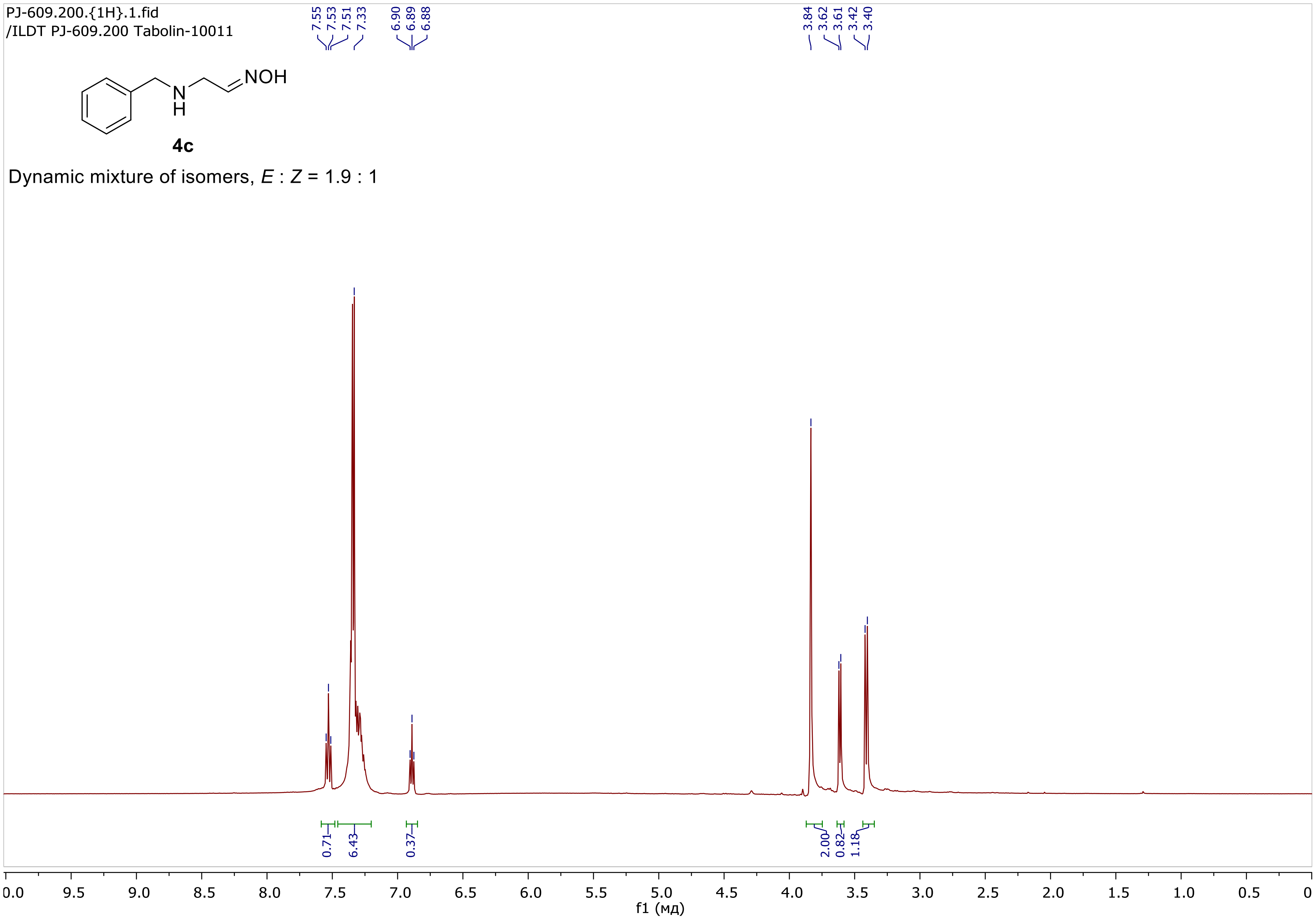

Dynamic mixture of isomers, *E* : *Z* = 1.9 : 1

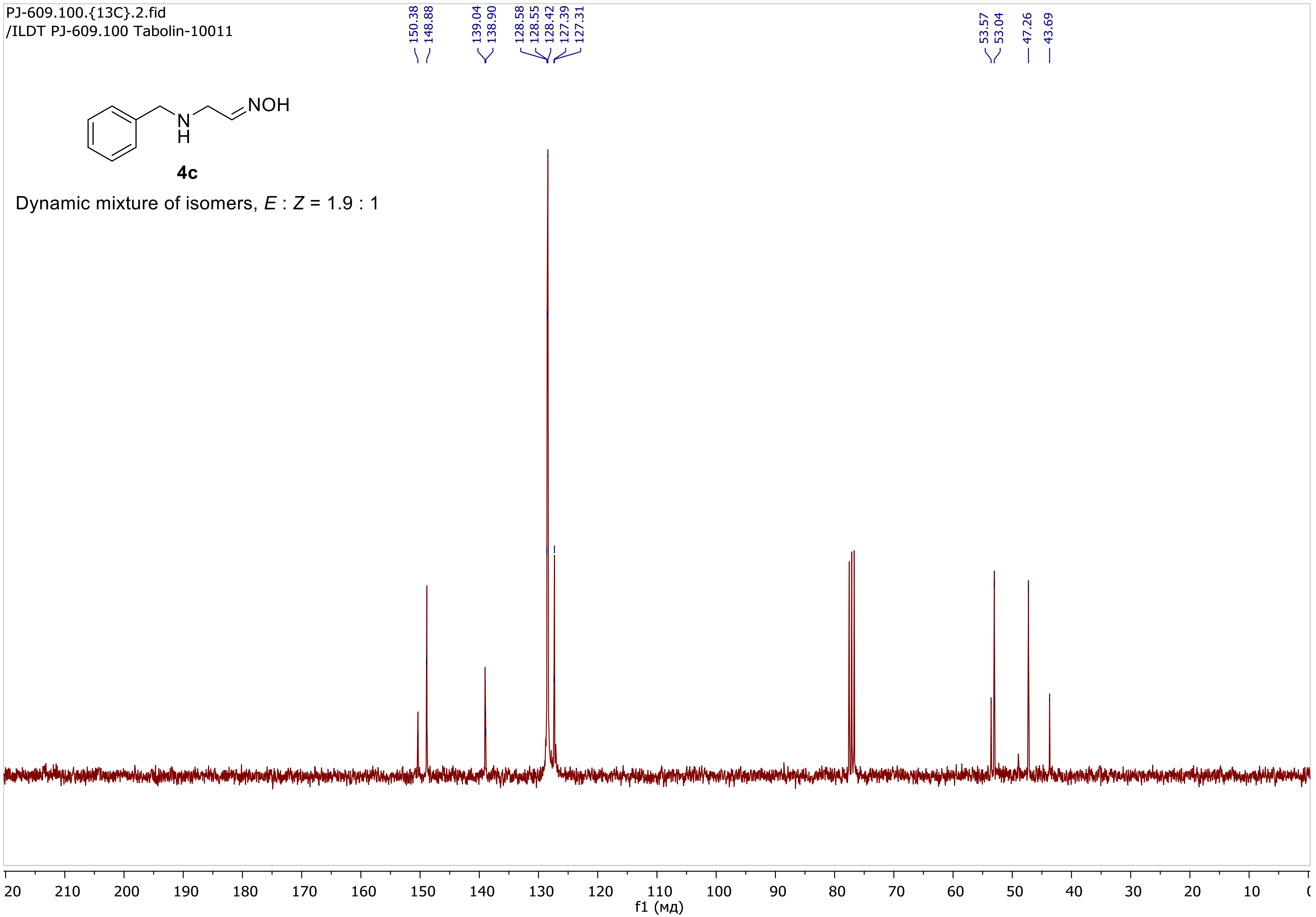
PJ-609.100.{13C}.2.fid
/ILDT PJ-609.100 Tabolin-10011
150.38
148.88
139.04
138.90
128.58
128.55
128.42
127.39
127.31
53.57
53.04
47.26
43.69
NOH
N
H
4c
Dynamic mixture of isomers, *E* : *Z* = 1.9 : 1
20 210 200 190 180 170 160 150 140 130 120 110 100 90 80 70 60 50 40 30 20 10
f1 (мд)

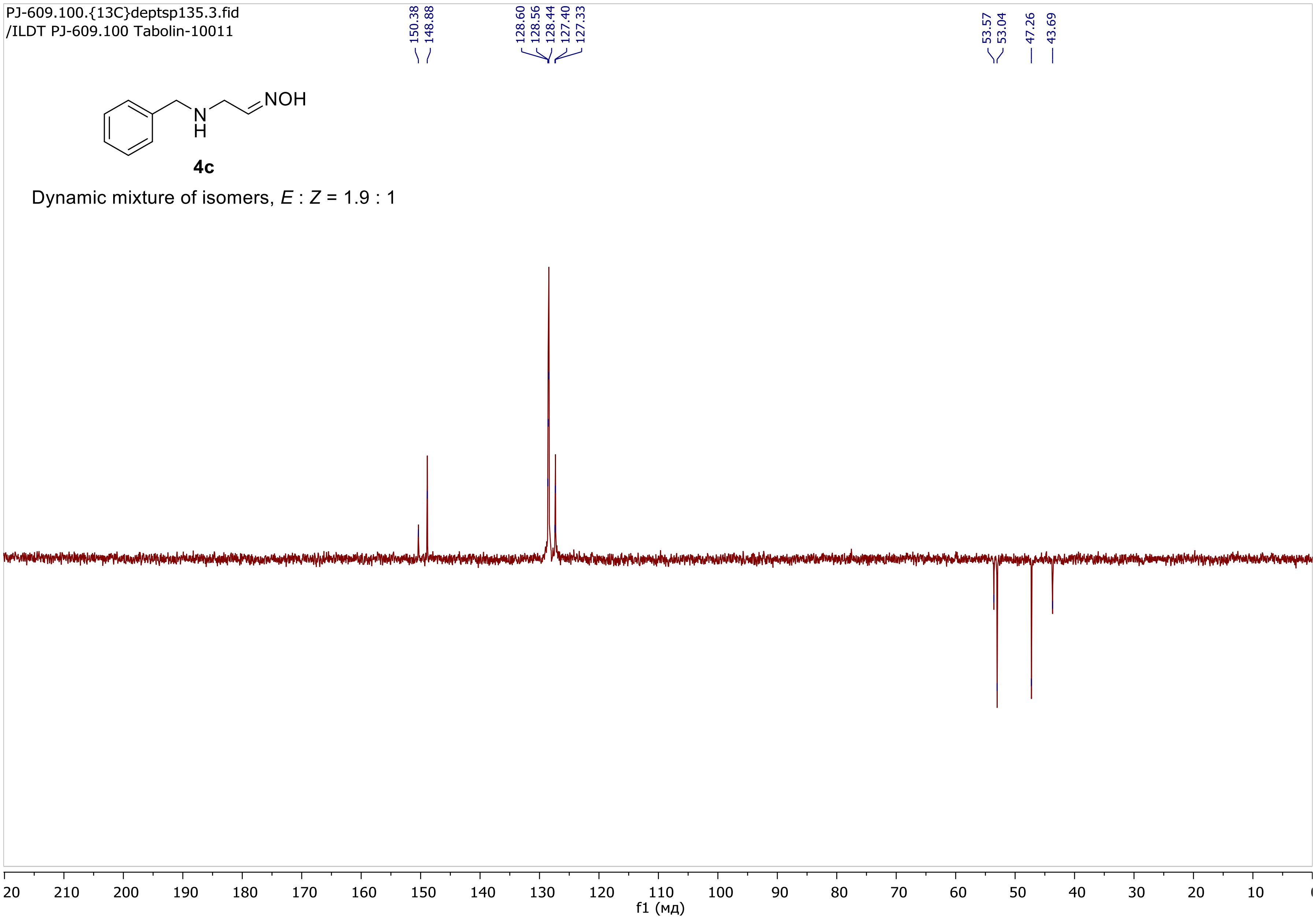
PJ-609.100.{13C}deptsp135.3.fid
/ILDT PJ-609.100 Tabolin-10011
150.38
148.88
128.60
128.56
128.44
127.40
127.33
53.57
53.04
47.26
43.69
NOH
N
H
4c
Dynamic mixture of isomers, E : Z = 1.9 : 1
20 210 200 190 180 170 160 150 140 130 120 110 100 90 80 70 60 50 40 30 20 10
f1 (мд)

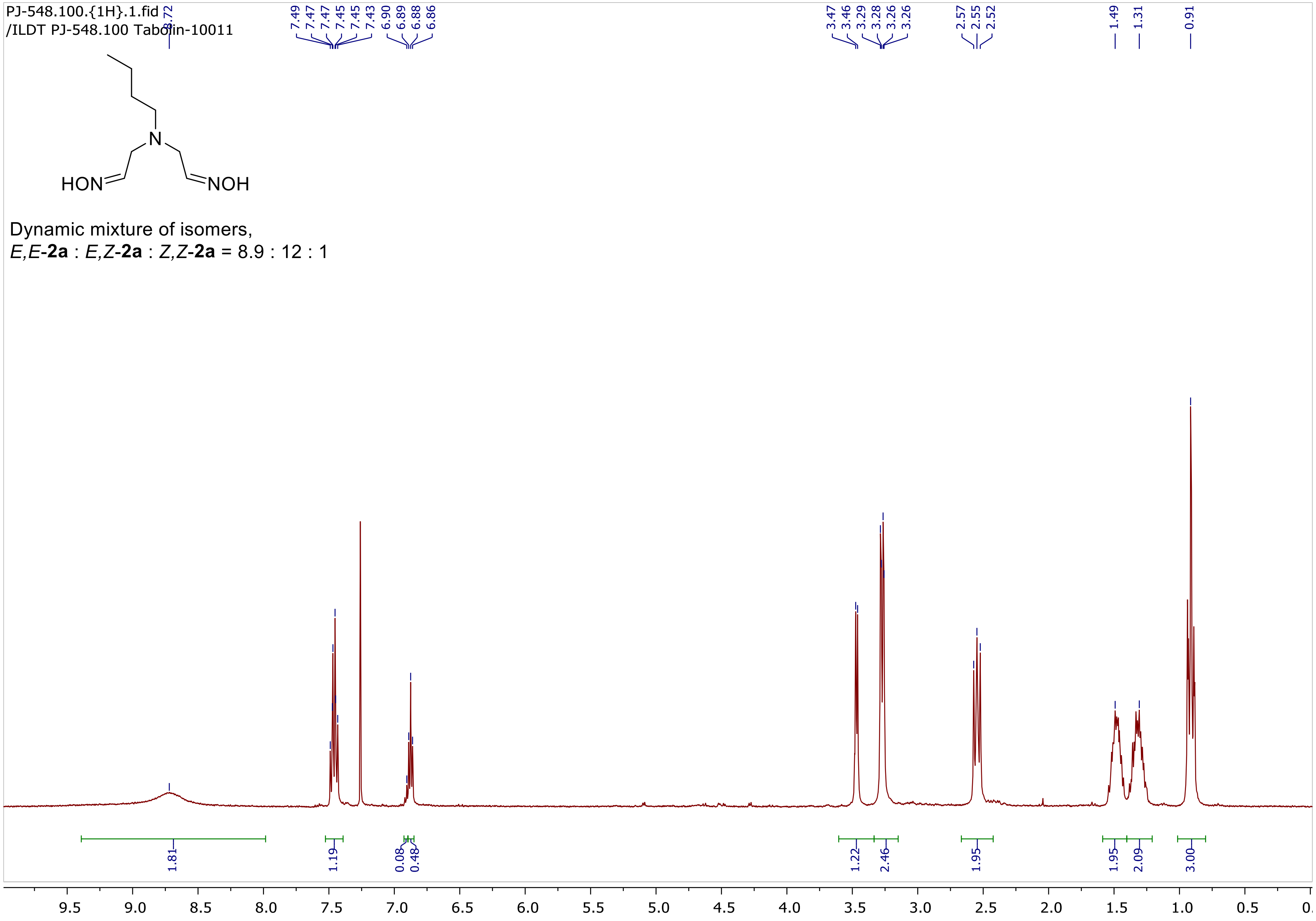
PJ-548.100.{1H}.1.fid
/ILDT PJ-548.100 Tabolin-10011
HON
N
NOH
Dynamic mixture of isomers,
E,*E*-**2a** : *E*,*Z*-**2a** : *Z*,*Z*-**2a** = 8.9 : 12 : 1
8.72
7.49 7.47 7.47 7.45 7.45 7.43
6.90 6.89 6.88 6.86
3.47 3.46 3.29 3.28 3.26 3.26
2.57 2.55 2.52
1.49
1.31
0.91
1.81
1.19
0.08
0.48
1.22
2.46
1.95
1.95
2.09
3.00
9.5 9.0 8.5 8.0 7.5 7.0 6.5 6.0 5.5 5.0 4.5 4.0 3.5 3.0 2.5 2.0 1.5 1.0 0.5 0.

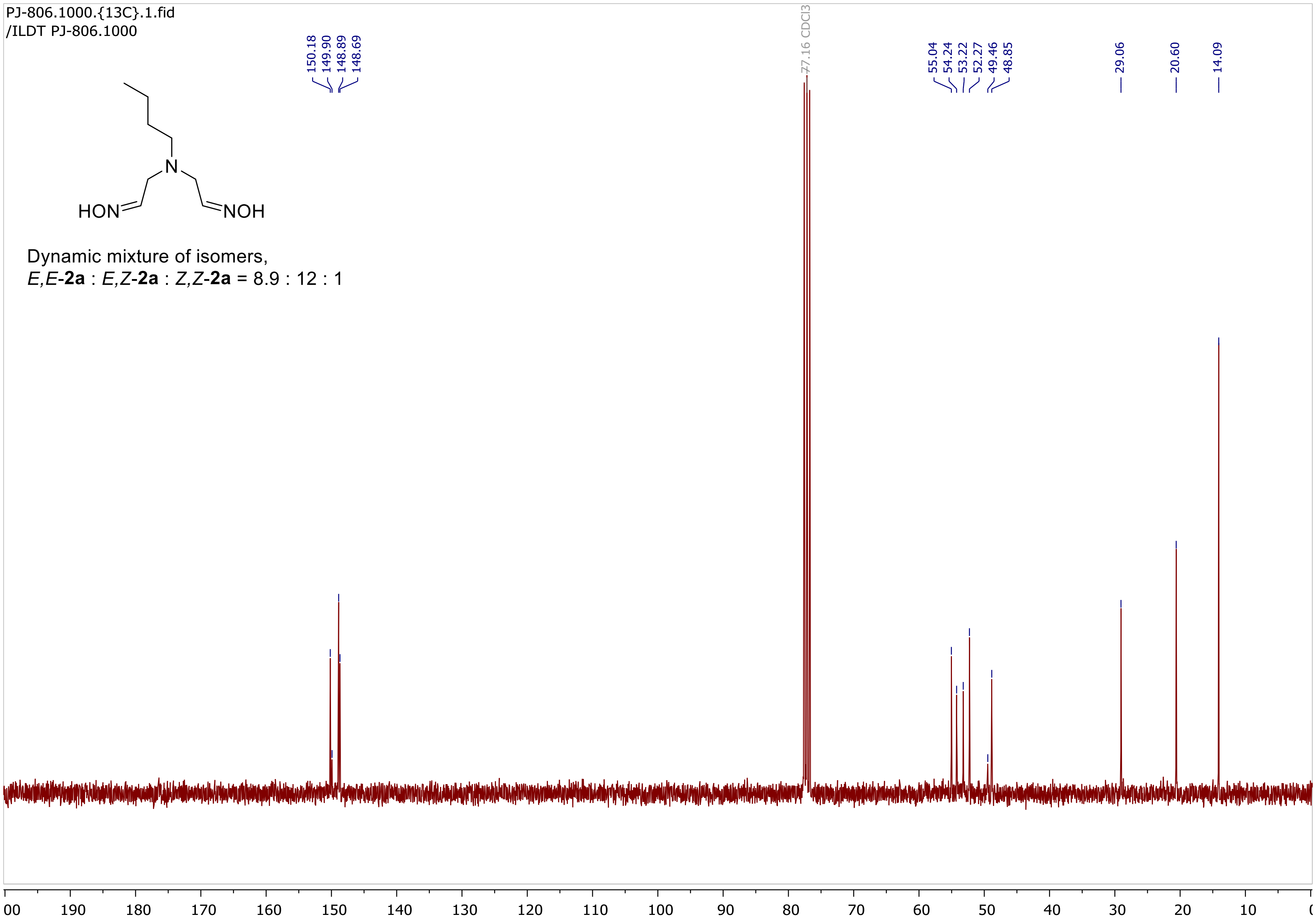

PJ-806.1000.{13C}.1.fid
/ILDT PJ-806.1000
HON
N
NOH
Dynamic mixture of isomers,
E,E-**2a** : *E,Z*-**2a** : *Z,Z*-**2a** = 8.9 : 12 : 1
150.18
149.90
148.89
148.69
77.16 CDCl3
55.04
54.24
53.22
52.27
49.46
48.85
29.06
20.60
14.09
00
190
180
170
160
150
140
130
120
110
100
90
80
70
60
50
40
30
20
10

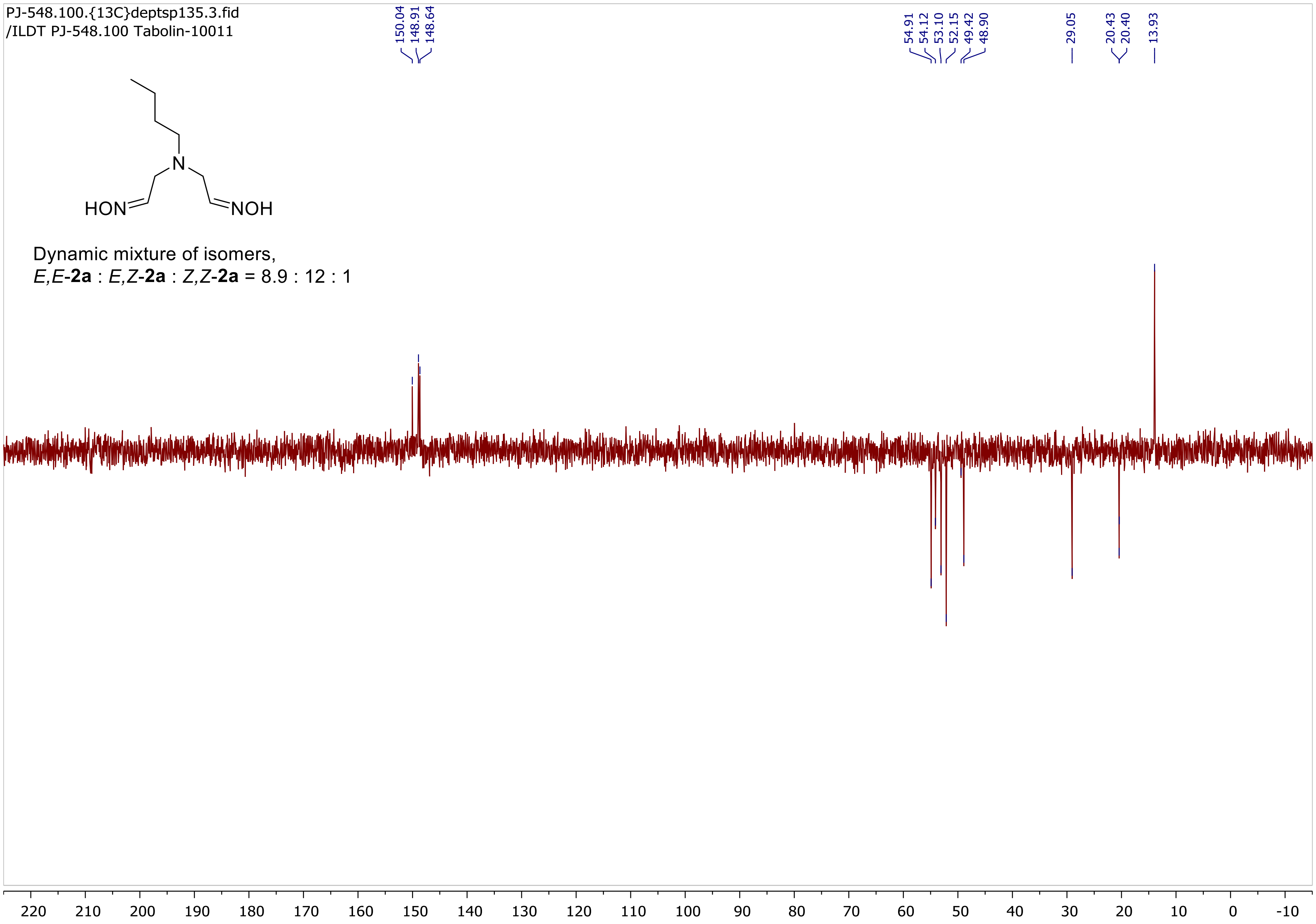
PJ-548.100.{13C}deptsp135.3.fid
/ILDT PJ-548.100 Tabolin-10011
150.04
148.91
148.64
54.91
54.12
53.10
52.15
49.42
48.90
29.05
20.43
20.40
13.93
HON
N
NOH
Dynamic mixture of isomers,
E,*E*-**2a** : *E*,*Z*-**2a** : *Z*,*Z*-**2a** = 8.9 : 12 : 1
220 210 200 190 180 170 160 150 140 130 120 110 100 90 80 70 60 50 40 30 20 10 0 -10

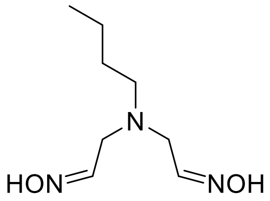

Dynamic mixture of isomers,
E,*E*-**2a** : *E*,*Z*-**2a** : *Z*,*Z*-**2a** =
8.9 : 12 : 1

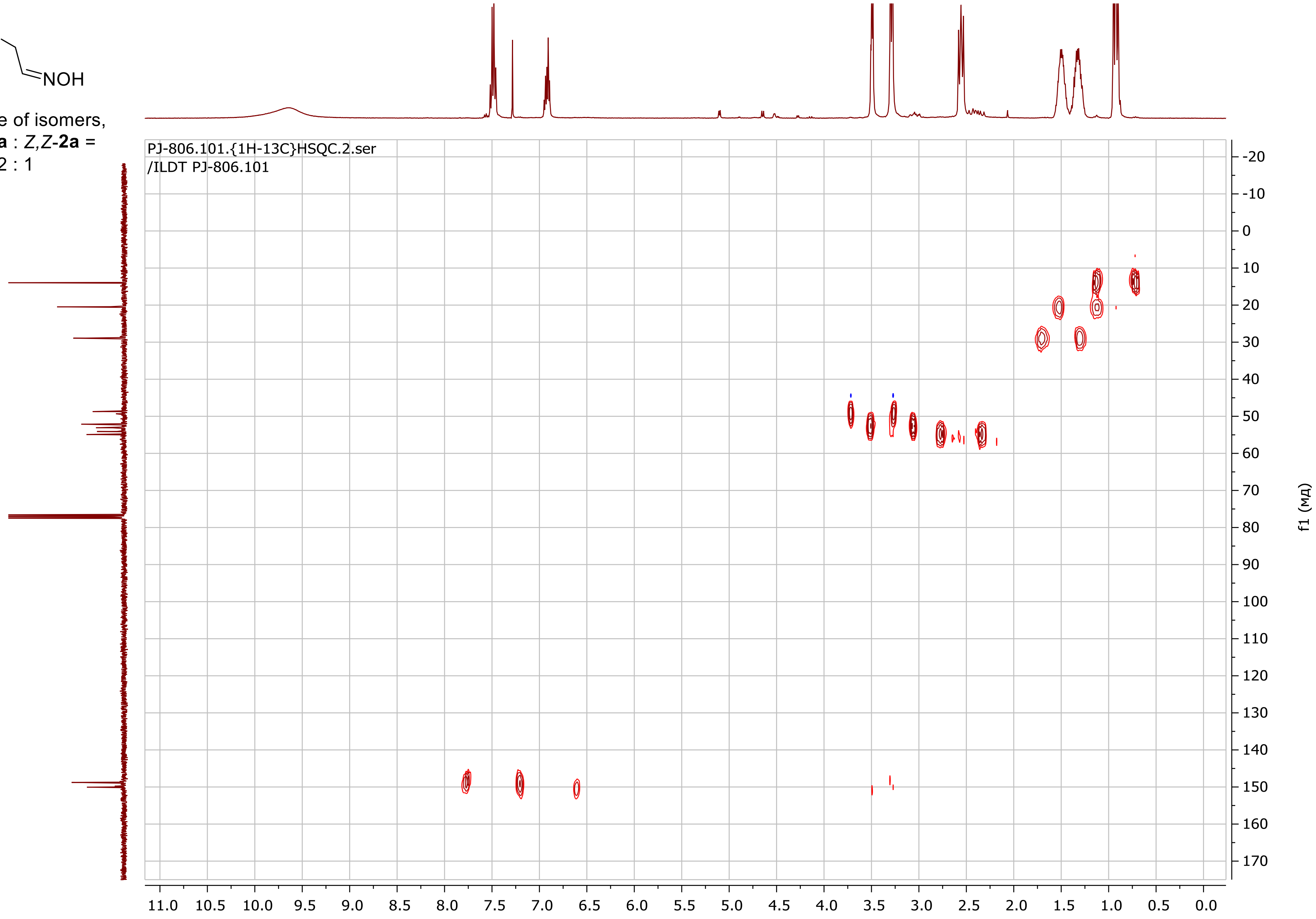

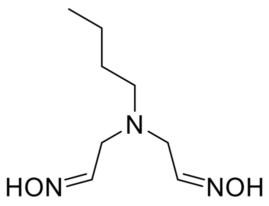

Dynamic mixture of isomers,
E,*E*-**2a** : *E*,*Z*-**2a** : *Z*,*Z*-**2a** =
8.9 : 12 : 1

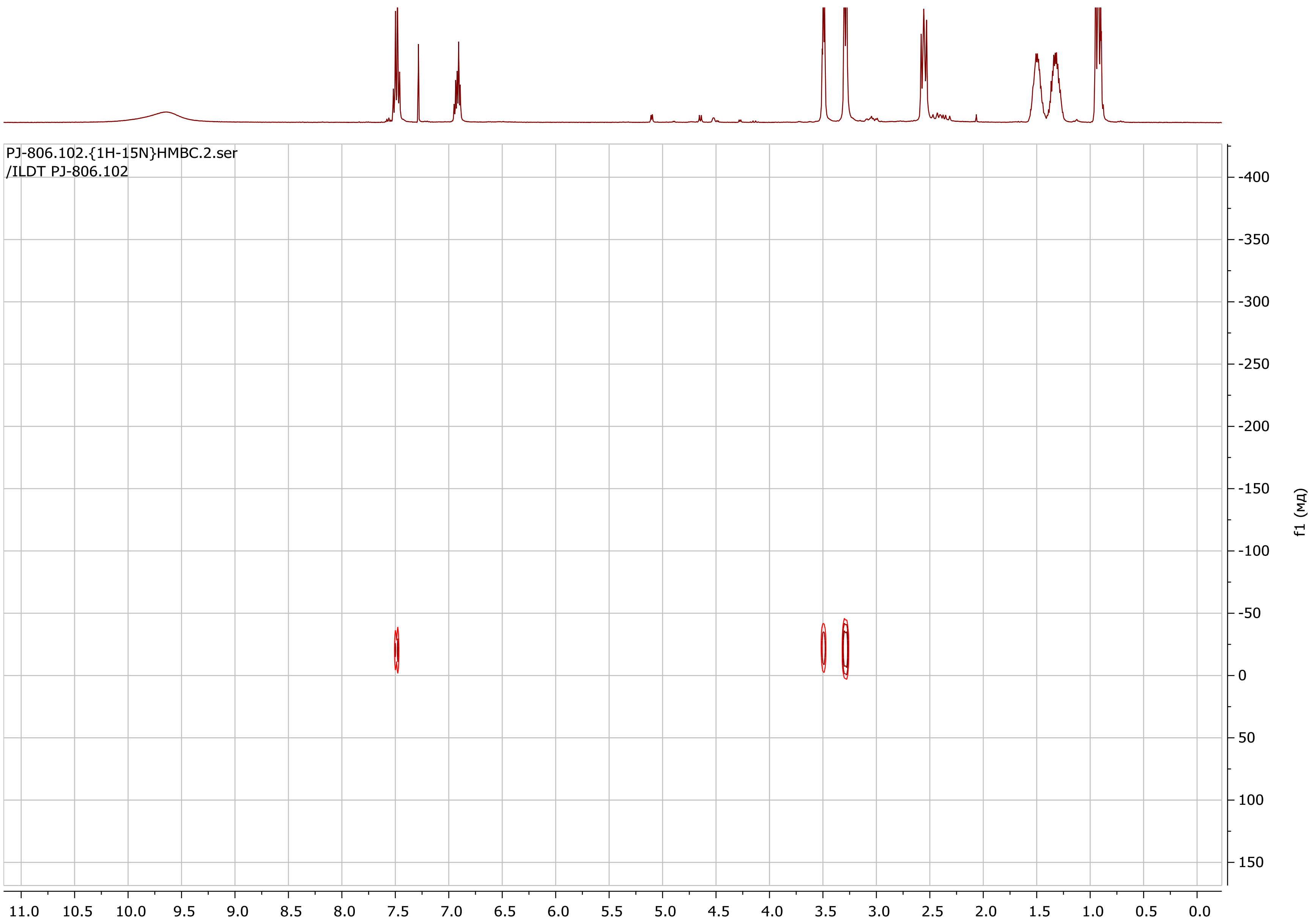

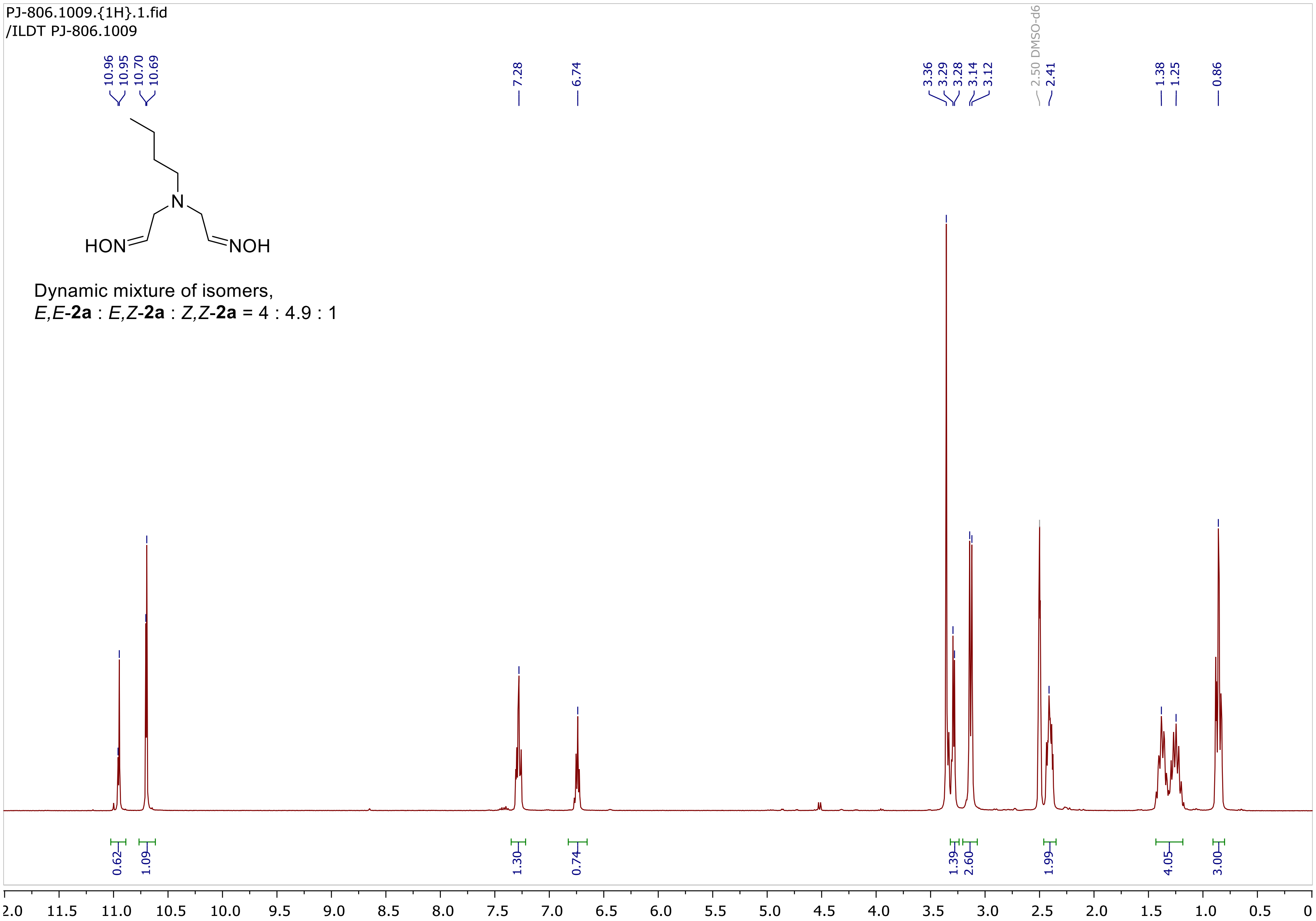
PJ-806.1009.{1H}.1.fid
/ILDT PJ-806.1009
10.96 10.95 10.70 10.69
7.28
6.74
3.36 3.29 3.28 3.14 3.12
2.50 DMSO-d6
2.41
1.38 1.25
0.86
HON
N
NOH
Dynamic mixture of isomers,
E,E-2a : E,Z-2a : Z,Z-2a = 4 : 4.9 : 1
0.62 1.09 1.30 0.74 1.39 2.60 1.99 4.05 3.00
12.0 11.5 11.0 10.5 10.0 9.5 9.0 8.5 8.0 7.5 7.0 6.5 6.0 5.5 5.0 4.5 4.0 3.5 3.0 2.5 2.0 1.5 1.0 0.5 0

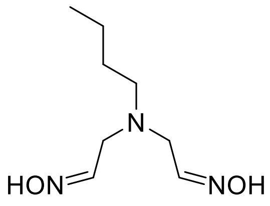

Dynamic mixture of isomers,
E,*E*-**2a** : *E*,*Z*-**2a** : *Z*,*Z*-**2a** =
4 : 4.9 : 1

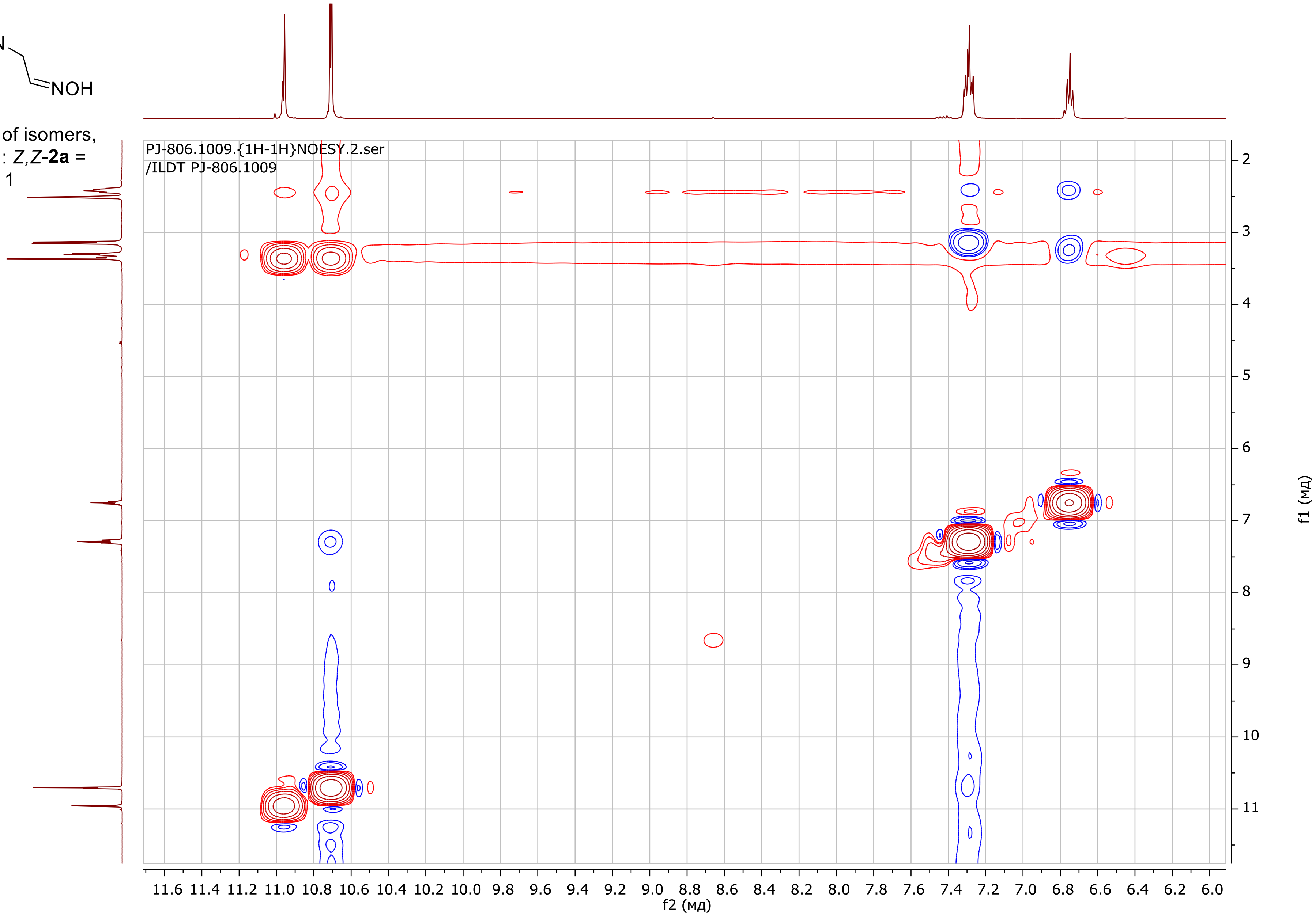

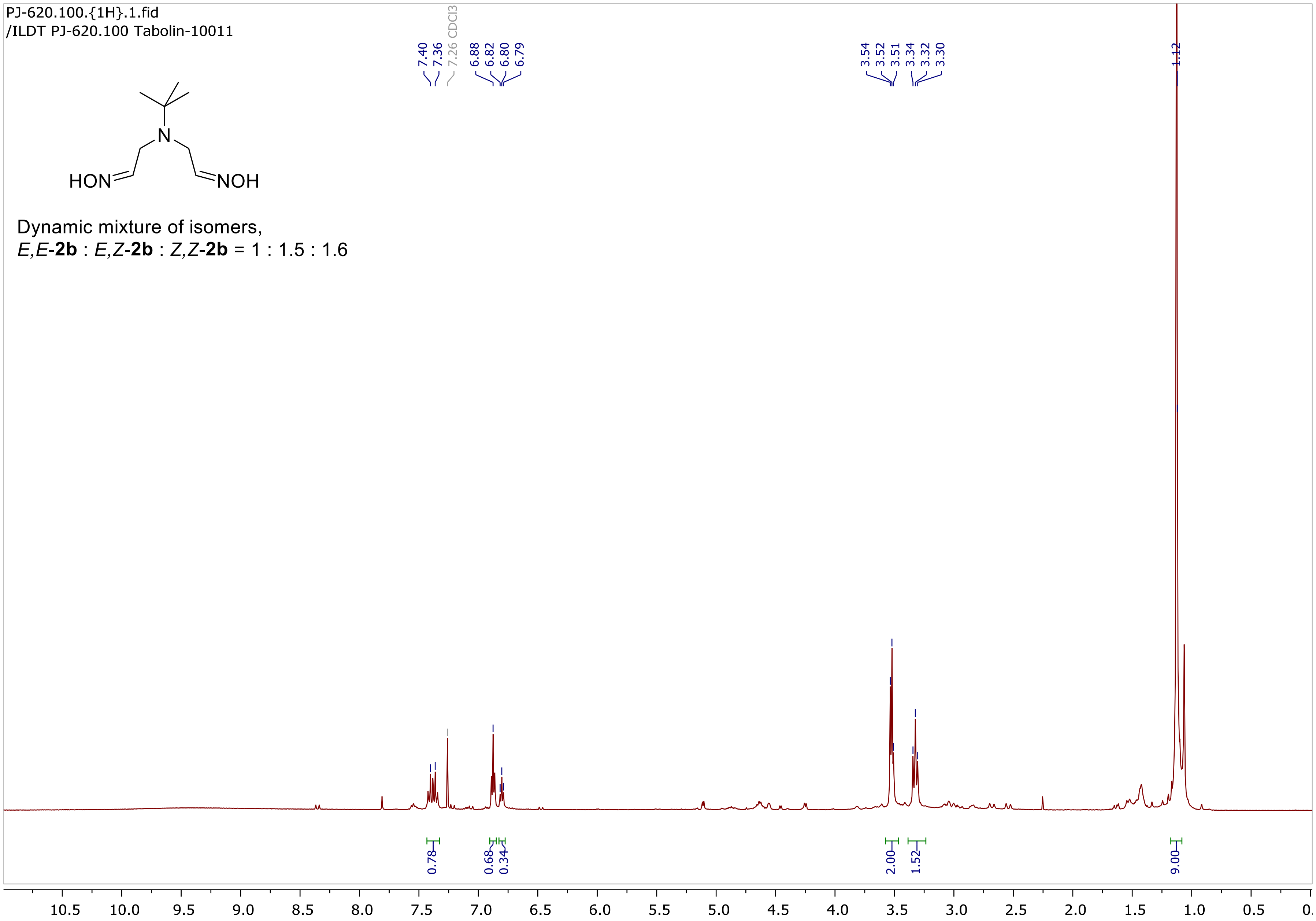
PJ-620.100.{1H}.1.fid
/ILDT PJ-620.100 Tabolin-10011
HON
N
NOH
Dynamic mixture of isomers,
E,E-**2b** : *E,Z*-**2b** : *Z,Z*-**2b** = 1 : 1.5 : 1.6
7.40
7.36
7.26 CDCl3
6.88
6.82
6.80
6.79
3.54
3.52
3.51
3.34
3.32
3.30
1.12
0.78
0.68
0.34
2.00
1.52
9.00
10.5 10.0 9.5 9.0 8.5 8.0 7.5 7.0 6.5 6.0 5.5 5.0 4.5 4.0 3.5 3.0 2.5 2.0 1.5 1.0 0.5 0.

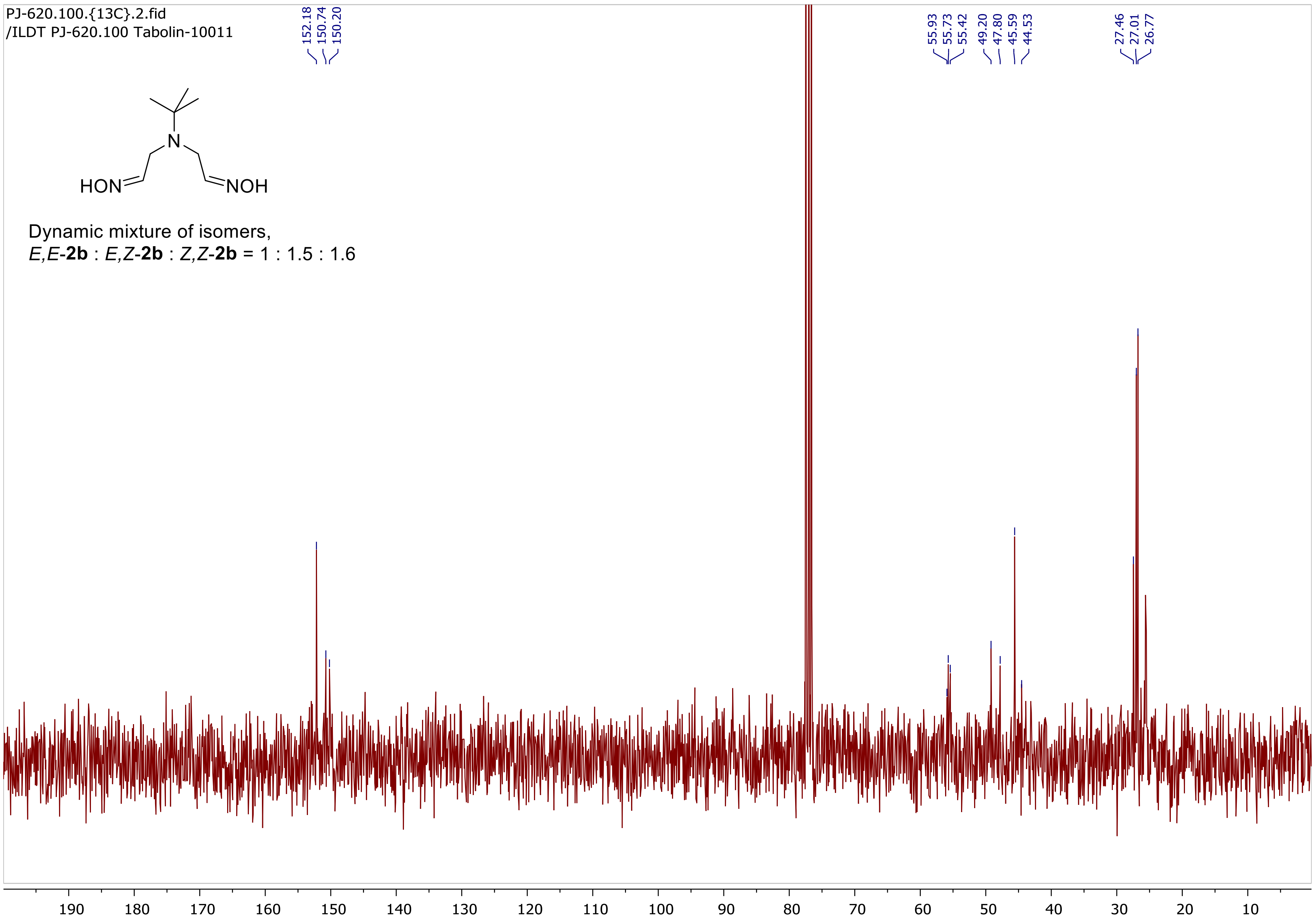
PJ-620.100.{13C}.2.fid
/ILDT PJ-620.100 Tabolin-10011
152.18
150.74
150.20
55.93
55.73
55.42
49.20
47.80
45.59
44.53
27.46
27.01
26.77
HON
N
NOH
Dynamic mixture of isomers,
E,E-**2b** : *E,Z*-**2b** : *Z,Z*-**2b** = 1 : 1.5 : 1.6
190
180
170
160
150
140
130
120
110
100
90
80
70
60
50
40
30
20
10

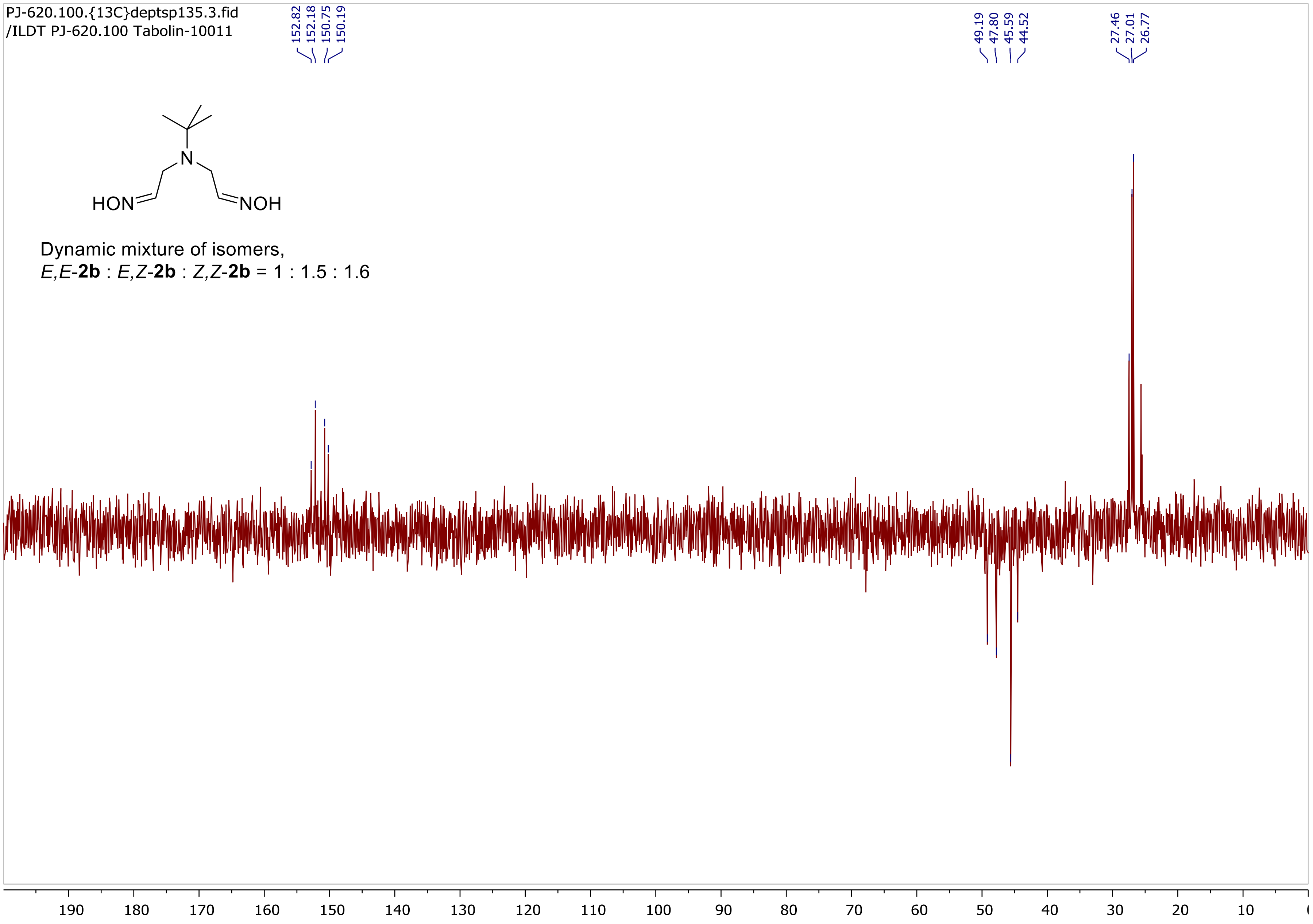
PJ-620.100.{13C}deptsp135.3.fid
/ILDT PJ-620.100 Tabolin-10011
152.82
152.18
150.75
150.19
49.19
47.80
45.59
44.52
27.46
27.01
26.77
HON
N
NOH
Dynamic mixture of isomers,
E,*E*-**2b** : *E*,*Z*-**2b** : *Z*,*Z*-**2b** = 1 : 1.5 : 1.6
190
180
170
160
150
140
130
120
110
100
90
80
70
60
50
40
30
20
10

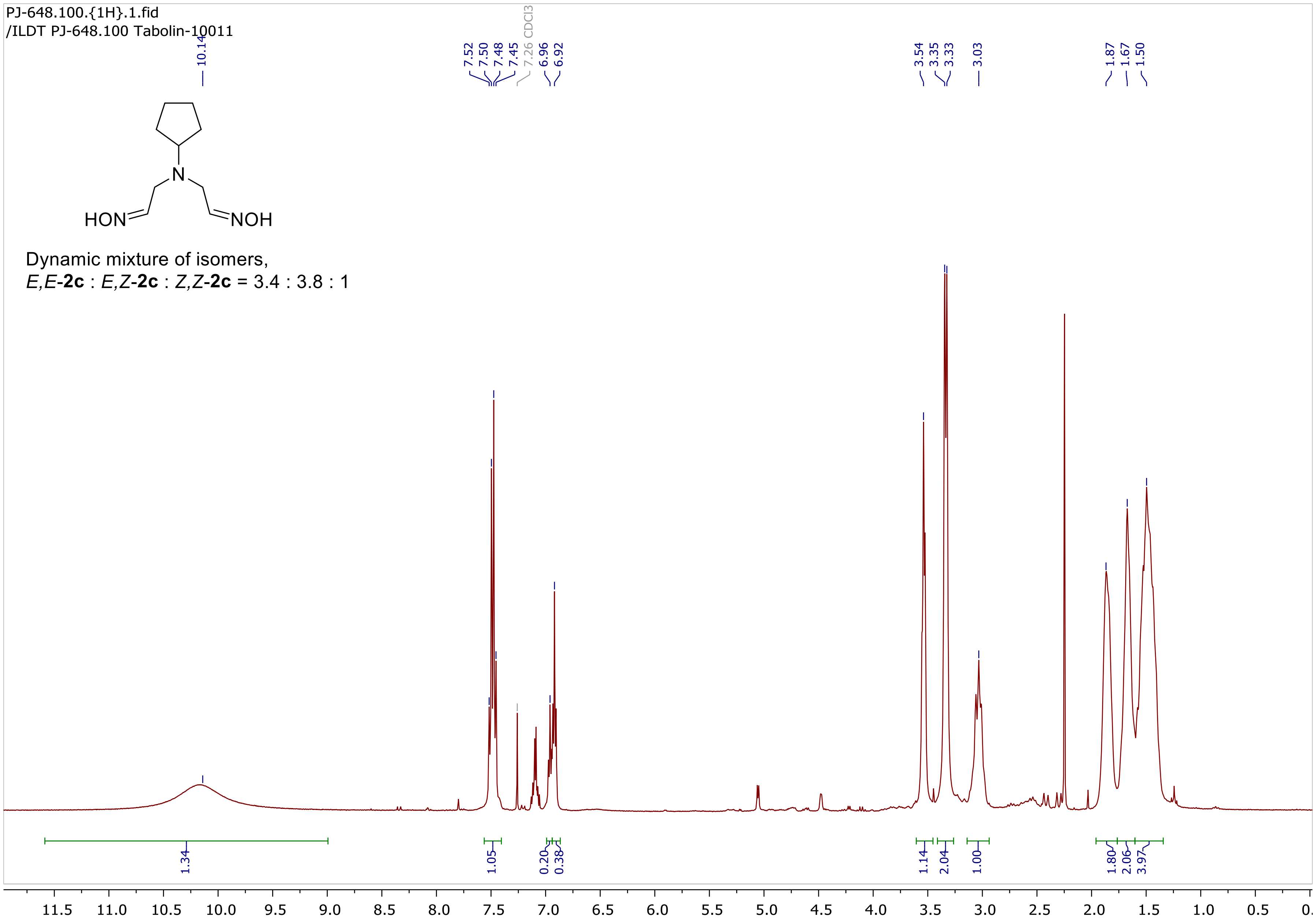
PJ-648.100.{1H}.1.fid
/ILDT PJ-648.100 Tabolin-10011
10.14
7.52
7.50
7.48
7.45
7.26 CDCl3
6.96
6.92
3.54
3.35
3.33
3.03
1.87
1.67
1.50
HON
N
NOH
Dynamic mixture of isomers,
E,*E*-**2c** : *E*,*Z*-**2c** : *Z*,*Z*-**2c** = 3.4 : 3.8 : 1
1.34
1.05
0.20
0.38
1.14
2.04
1.00
1.80
2.06
3.97
11.5
11.0
10.5
10.0
9.5
9.0
8.5
8.0
7.5
7.0
6.5
6.0
5.5
5.0
4.5
4.0
3.5
3.0
2.5
2.0
1.5
1.0
0.5

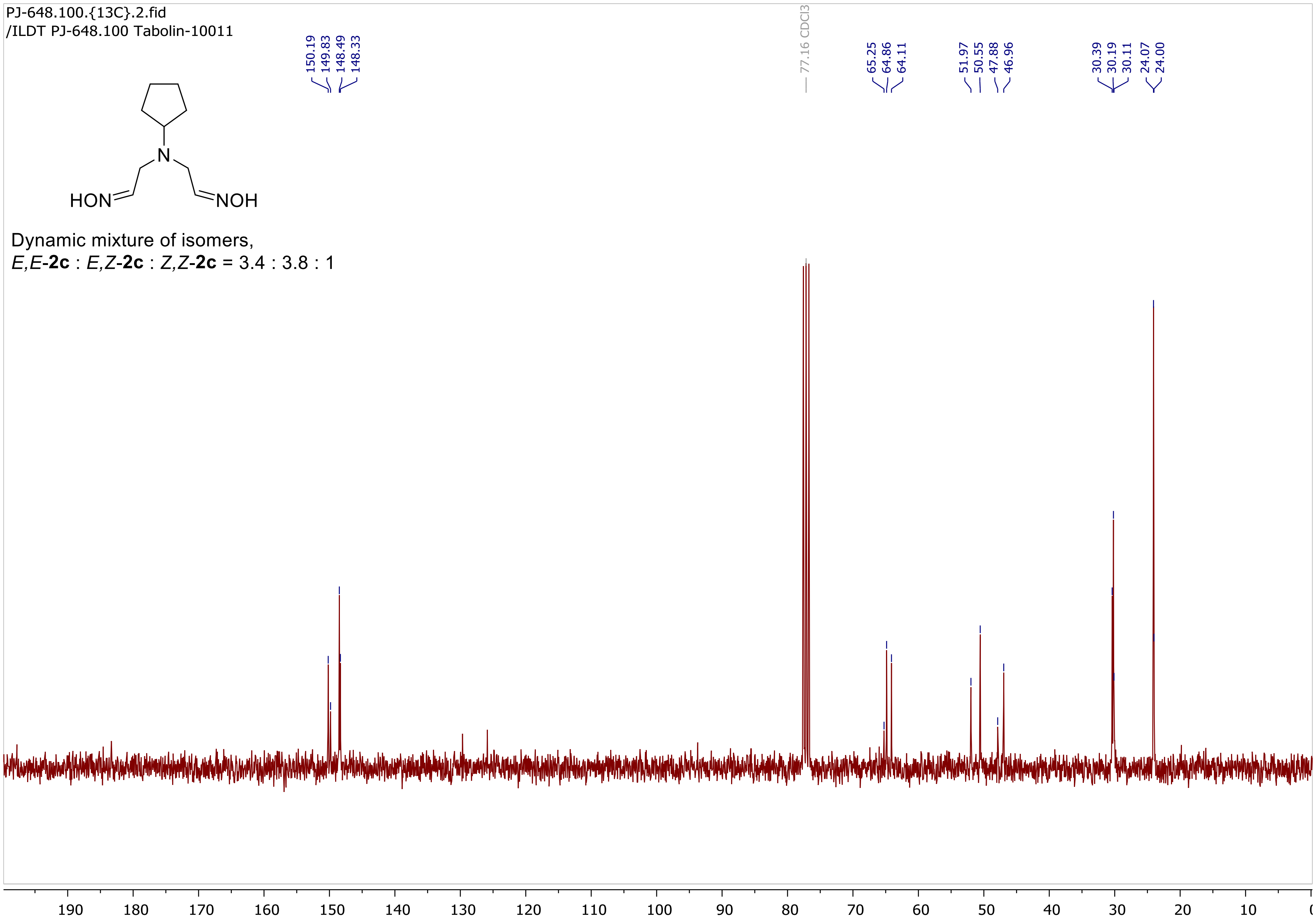
PJ-648.100.{13C}.2.fid
/ILDT PJ-648.100 Tabolin-10011
150.19
149.83
148.49
148.33
77.16 CDCl3
65.25
64.86
64.11
51.97
50.55
47.88
46.96
30.39
30.19
30.11
24.07
24.00
HON
N
NOH
Dynamic mixture of isomers,
E,E-**2c** : *E,Z*-**2c** : *Z,Z*-**2c** = 3.4 : 3.8 : 1
190
180
170
160
150
140
130
120
110
100
90
80
70
60
50
40
30
20
10

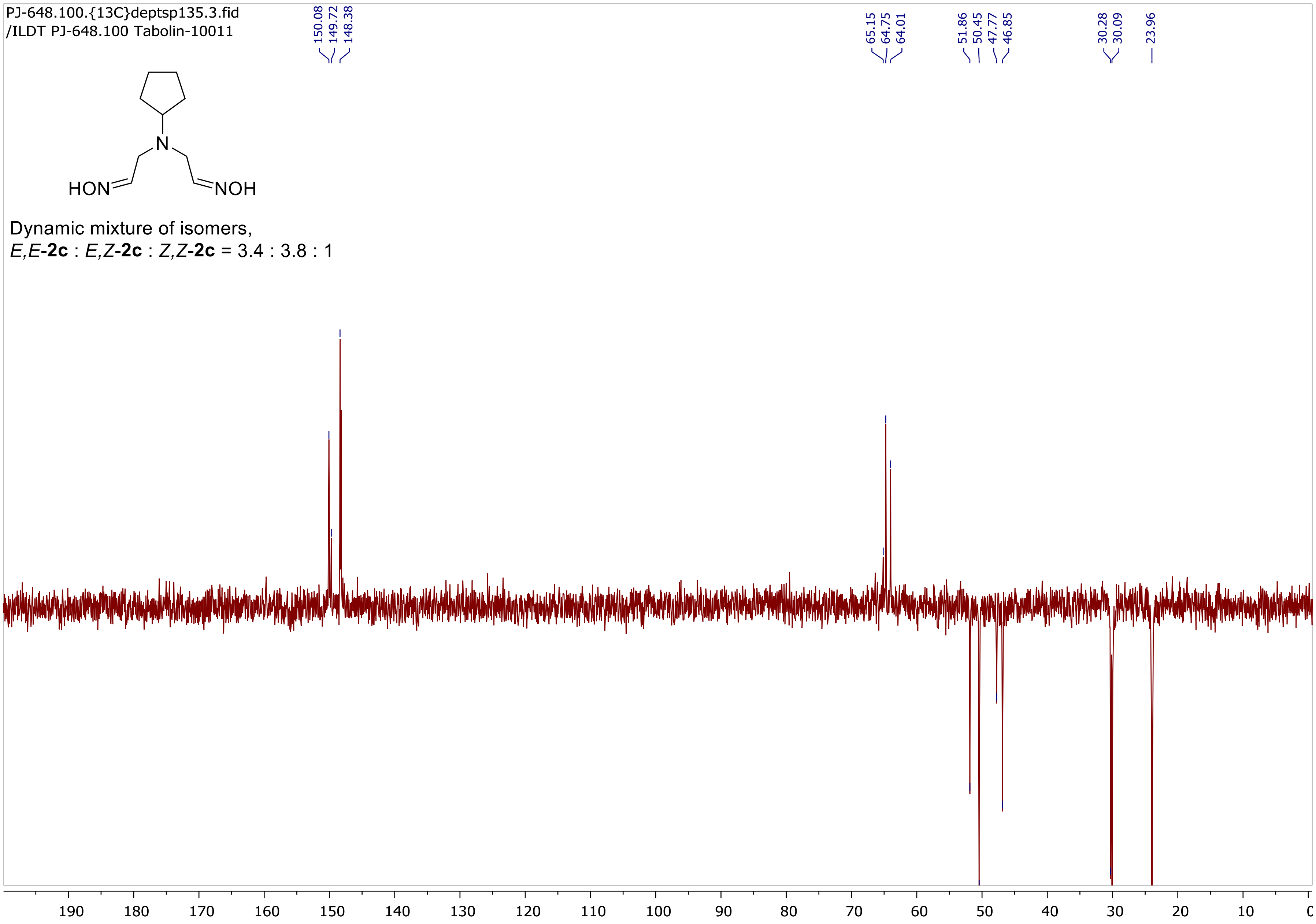
PJ-648.100.{13C}deptsp135.3.fid
/ILDT PJ-648.100 Tabolin-10011
150.08
149.72
148.38
65.15
64.75
64.01
51.86
50.45
47.77
46.85
30.28
30.09
23.96
HON
N
NOH
Dynamic mixture of isomers,
E,*E*-**2c** : *E*,*Z*-**2c** : *Z*,*Z*-**2c** = 3.4 : 3.8 : 1
190
180
170
160
150
140
130
120
110
100
90
80
70
60
50
40
30
20
10
0

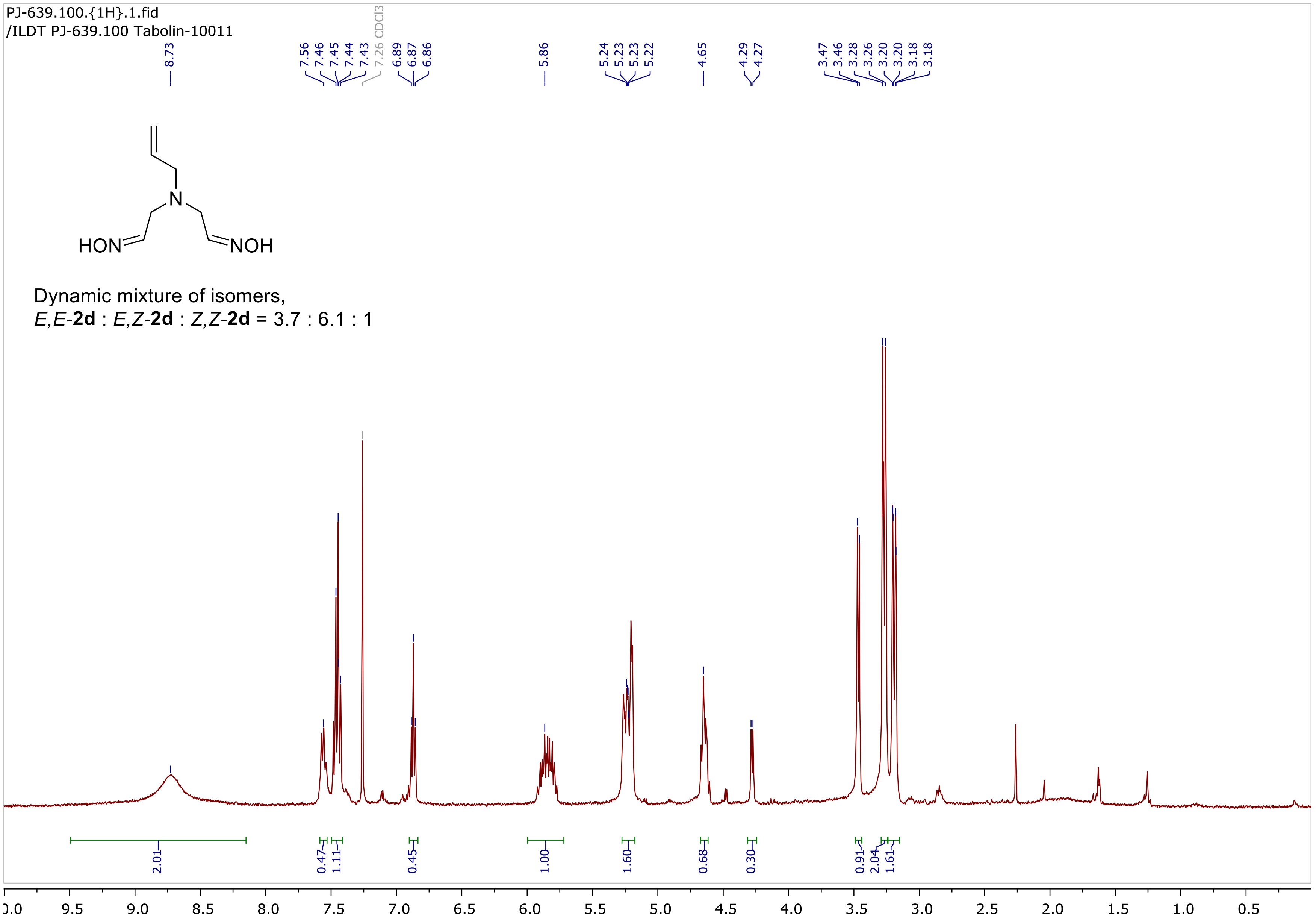
PJ-639.100.{1H}.1.fid
/ILDT PJ-639.100 Tabolin-10011
8.73
7.56
7.46
7.45
7.44
7.43
7.26 CDCl3
6.89
6.87
6.86
5.86
5.24
5.23
5.23
5.22
4.65
4.29
4.27
3.47
3.46
3.28
3.26
3.20
3.20
3.18
3.18
HON
N
NOH
Dynamic mixture of isomers,
E,E-**2d** : *E,Z*-**2d** : *Z,Z*-**2d** = 3.7 : 6.1 : 1
2.01
0.47
1.11
0.45
1.00
1.60
0.68
0.30
0.91
2.04
1.61
9.5
9.0
8.5
8.0
7.5
7.0
6.5
6.0
5.5
5.0
4.5
4.0
3.5
3.0
2.5
2.0
1.5
1.0
0.5

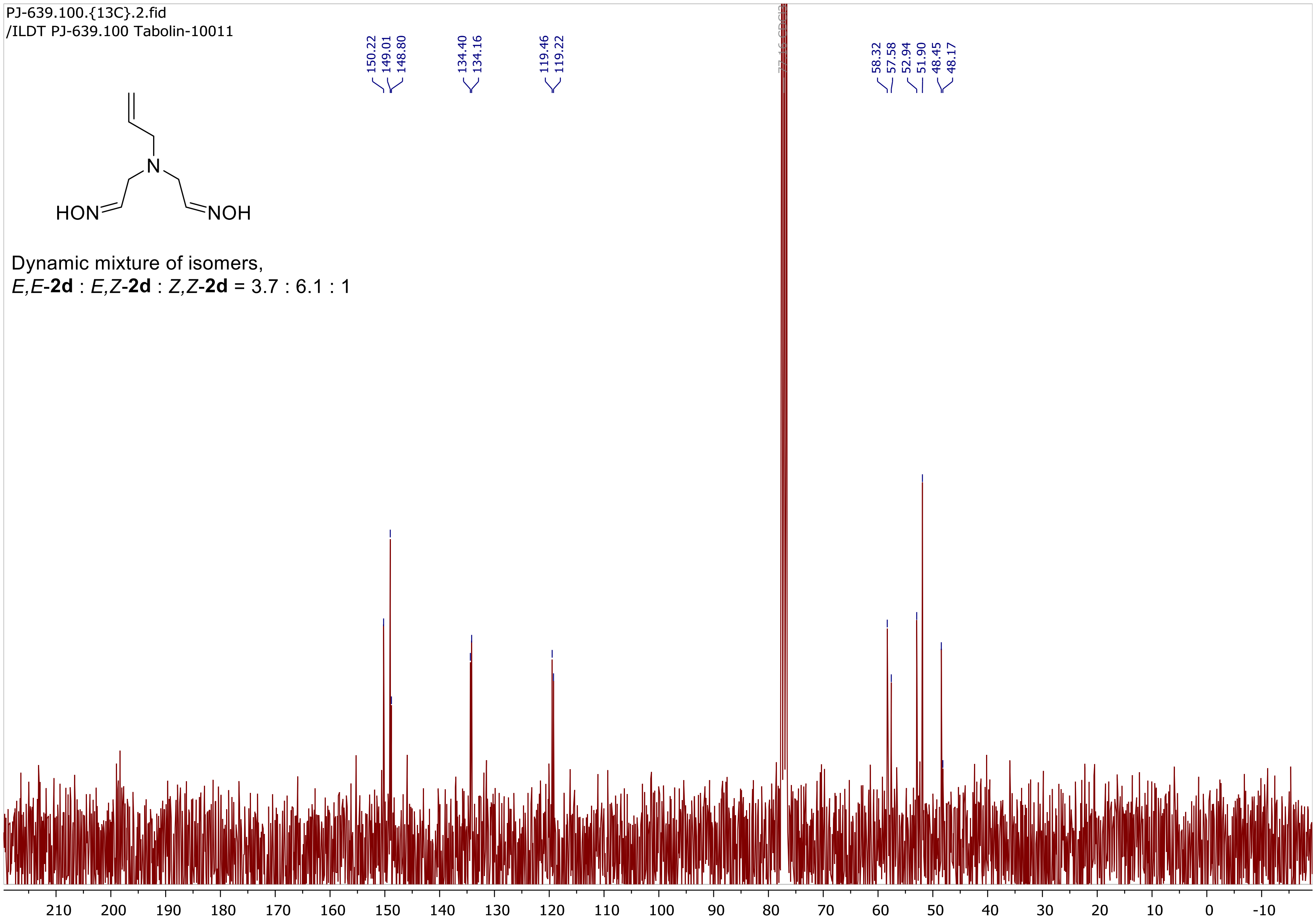

PJ-639.100.{13C}.2.fid
/ILDT PJ-639.100 Tabolin-10011
150.22
149.01
148.80
134.40
134.16
119.46
119.22
58.32
57.58
52.94
51.90
48.45
48.17
HON
N
NOH
Dynamic mixture of isomers,
E,E-**2d** : *E,Z*-**2d** : *Z,Z*-**2d** = 3.7 : 6.1 : 1
210
200
190
180
170
160
150
140
130
120
110
100
90
80
70
60
50
40
30
20
10
0
-10

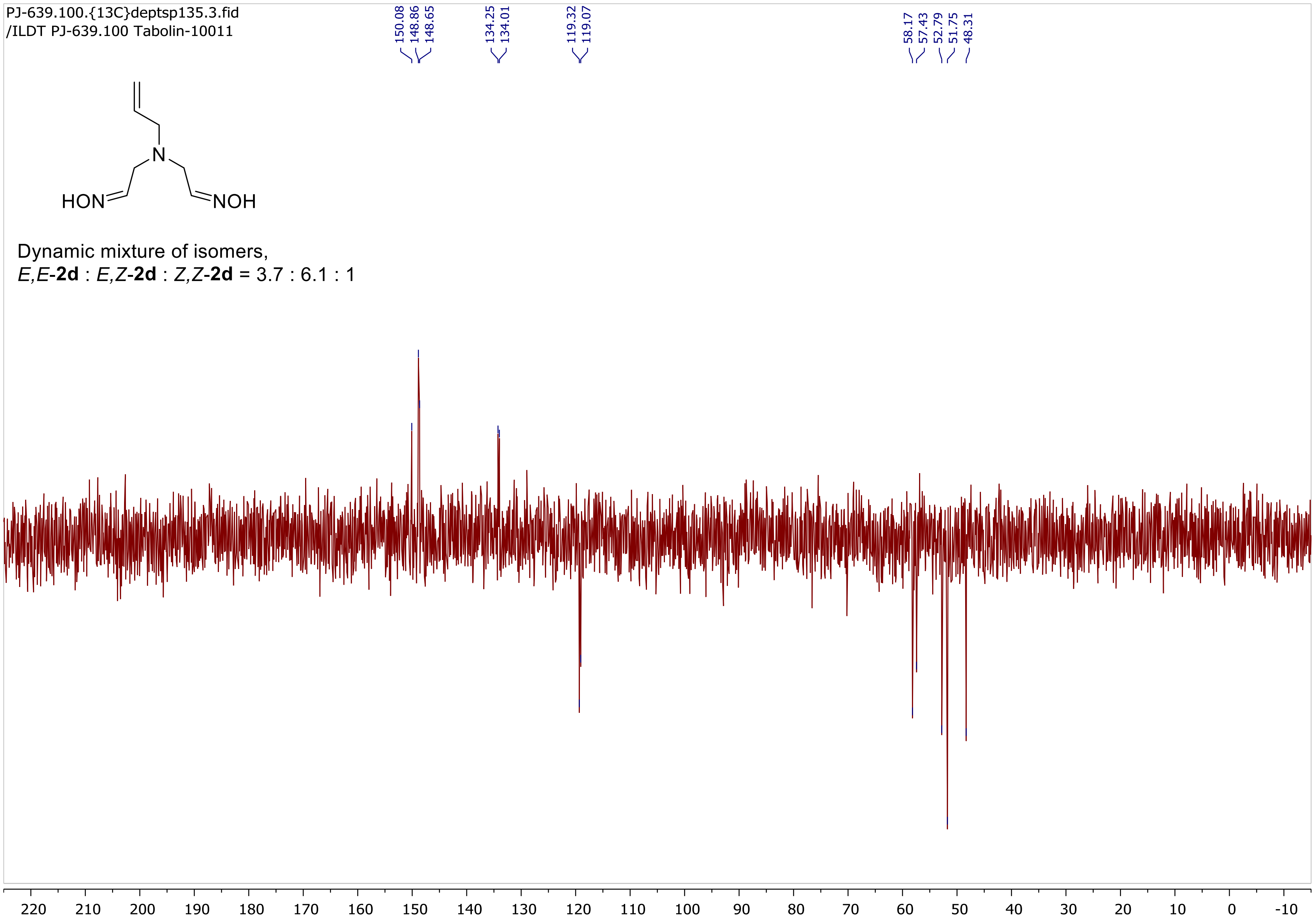
PJ-639.100.{13C}deptsp135.3.fid
/ILDT PJ-639.100 Tabolin-10011
150.08
148.86
148.65
134.25
134.01
119.32
119.07
58.17
57.43
52.79
51.75
48.31
HON
N
NOH
Dynamic mixture of isomers,
E,E-**2d** : *E,Z*-**2d** : *Z,Z*-**2d** = 3.7 : 6.1 : 1
220 210 200 190 180 170 160 150 140 130 120 110 100 90 80 70 60 50 40 30 20 10 0 -10

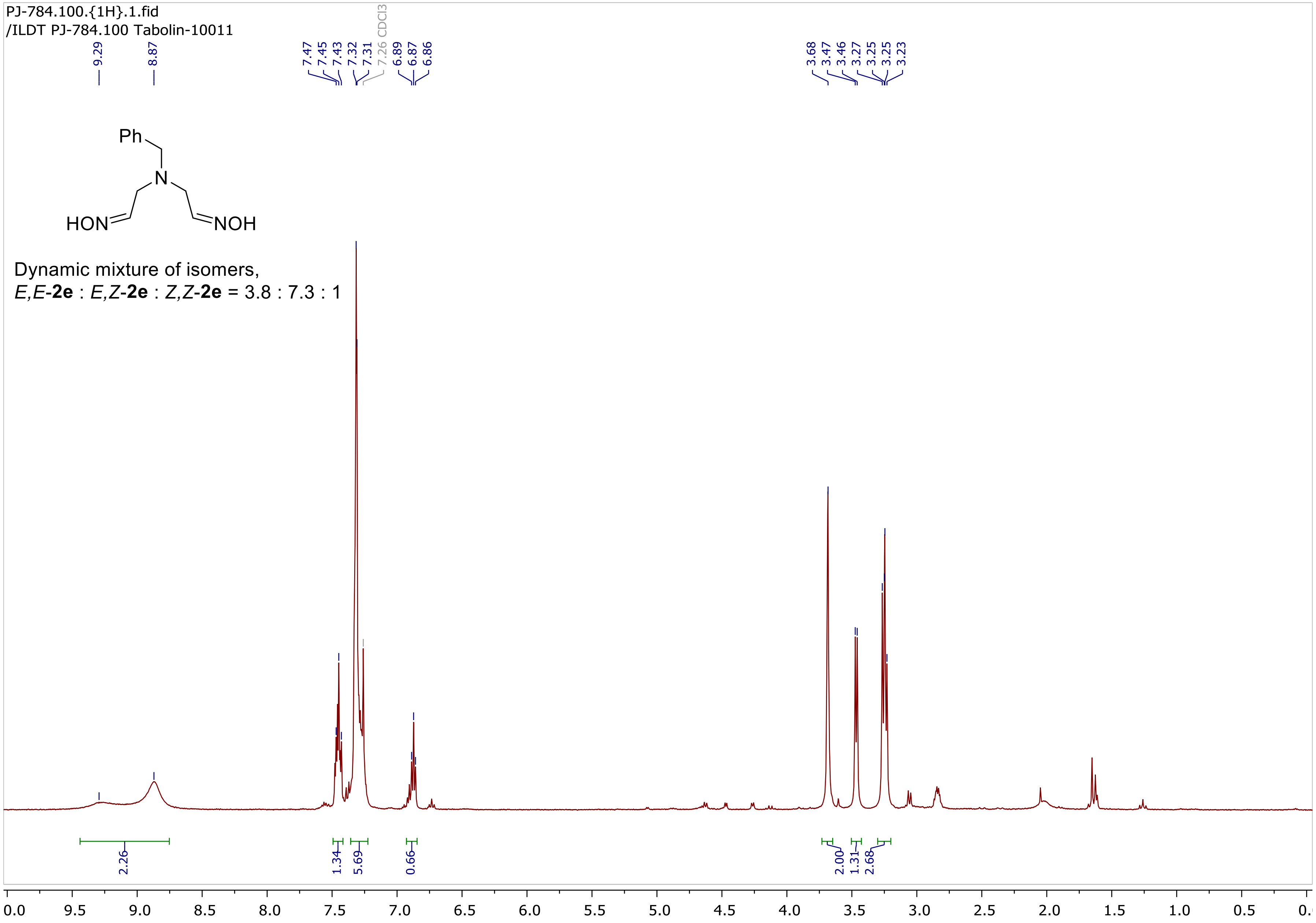
PJ-784.100.{1H}.1.fid
/ILDT PJ-784.100 Tabolin-10011
9.29
8.87
7.47
7.45
7.43
7.32
7.31
7.26 CDCl3
6.89
6.87
6.86
3.68
3.47
3.46
3.27
3.25
3.25
3.23
Ph
N
HON
NOH
Dynamic mixture of isomers,
E,*E*-**2e** : *E*,*Z*-**2e** : *Z*,*Z*-**2e** = 3.8 : 7.3 : 1
2.26
1.34
5.69
0.66
2.00
1.31
2.68
0.0
9.5
9.0
8.5
8.0
7.5
7.0
6.5
6.0
5.5
5.0
4.5
4.0
3.5
3.0
2.5
2.0
1.5
1.0
0.5

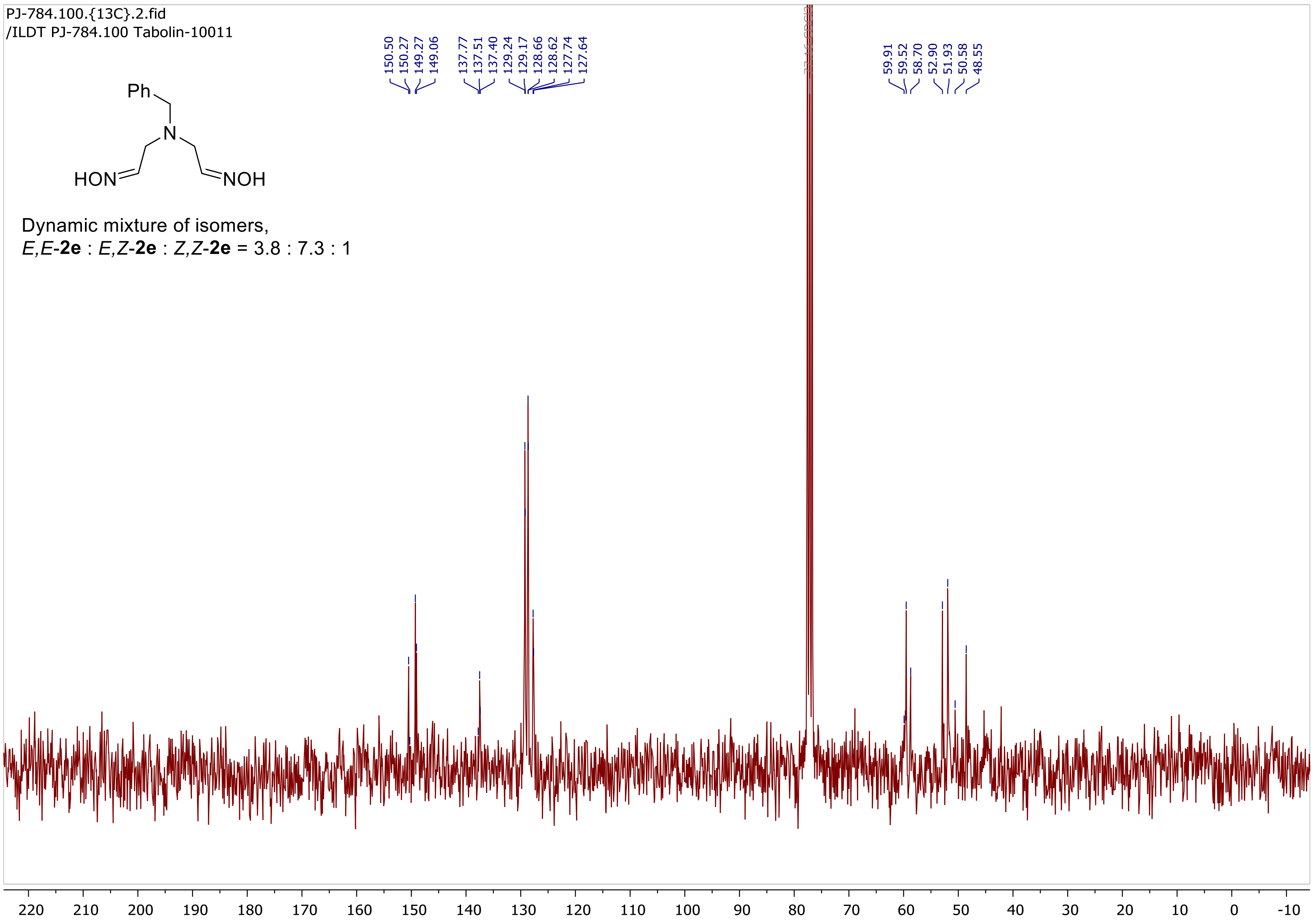
PJ-784.100.{13C}.2.fid
/ILDT PJ-784.100 Tabolin-10011
150.50
150.27
149.27
149.06
137.77
137.51
137.40
129.24
129.17
128.66
128.62
127.74
127.64
59.91
59.52
58.70
52.90
51.93
50.58
48.55
Ph
N
HON
NOH
Dynamic mixture of isomers,
E,*E*-**2e** : *E*,*Z*-**2e** : *Z*,*Z*-**2e** = 3.8 : 7.3 : 1
220 210 200 190 180 170 160 150 140 130 120 110 100 90 80 70 60 50 40 30 20 10 0 -10

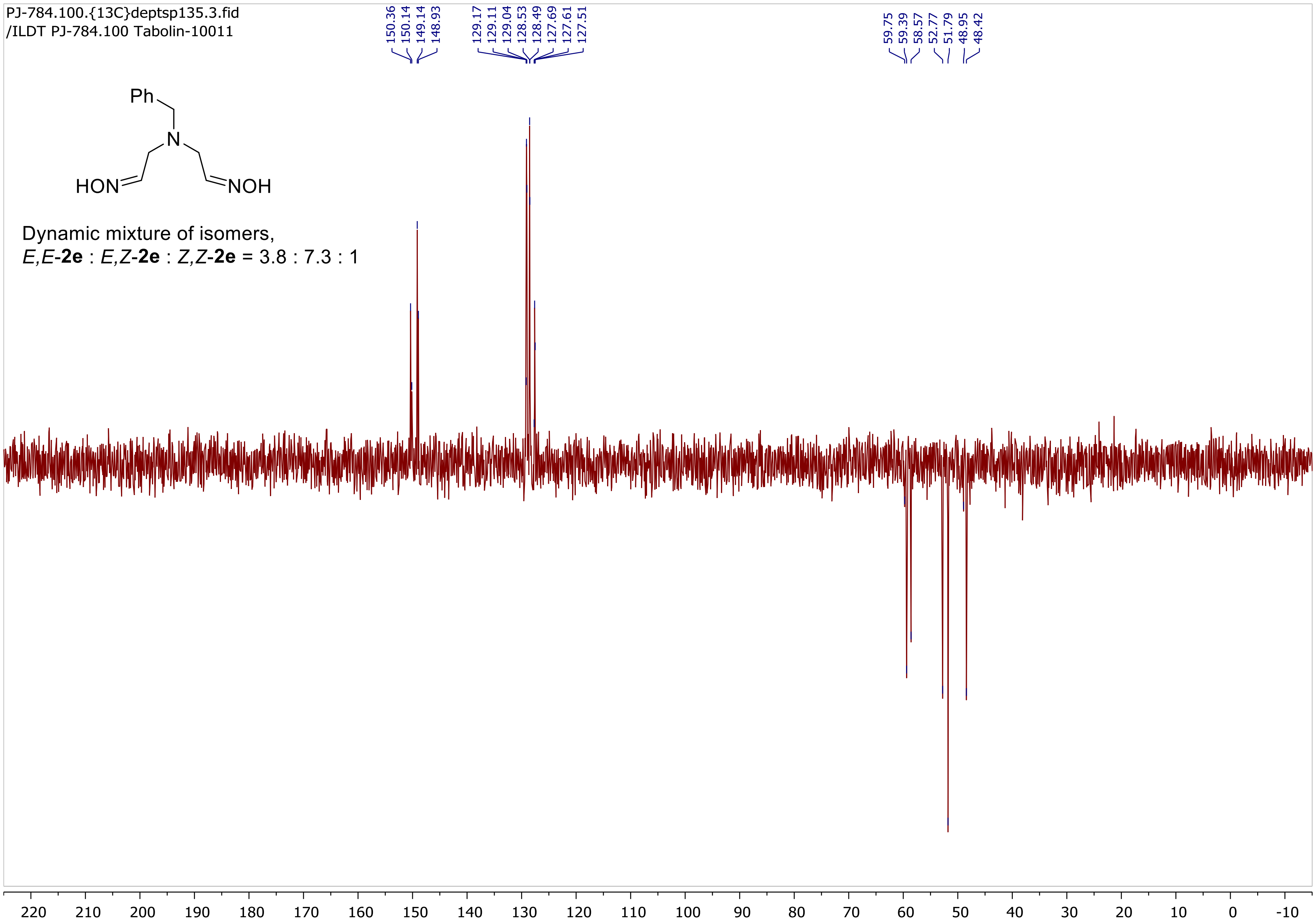
PJ-784.100.{13C}deptsp135.3.fid
/ILDT PJ-784.100 Tabolin-10011
150.36
150.14
149.14
148.93
129.17
129.11
129.04
128.53
128.49
127.69
127.61
127.51
59.75
59.39
58.57
52.77
51.79
48.95
48.42
Ph
N
HON
NOH
Dynamic mixture of isomers,
E,E-2e : E,Z-2e : Z,Z-2e = 3.8 : 7.3 : 1
220 210 200 190 180 170 160 150 140 130 120 110 100 90 80 70 60 50 40 30 20 10 0 -10

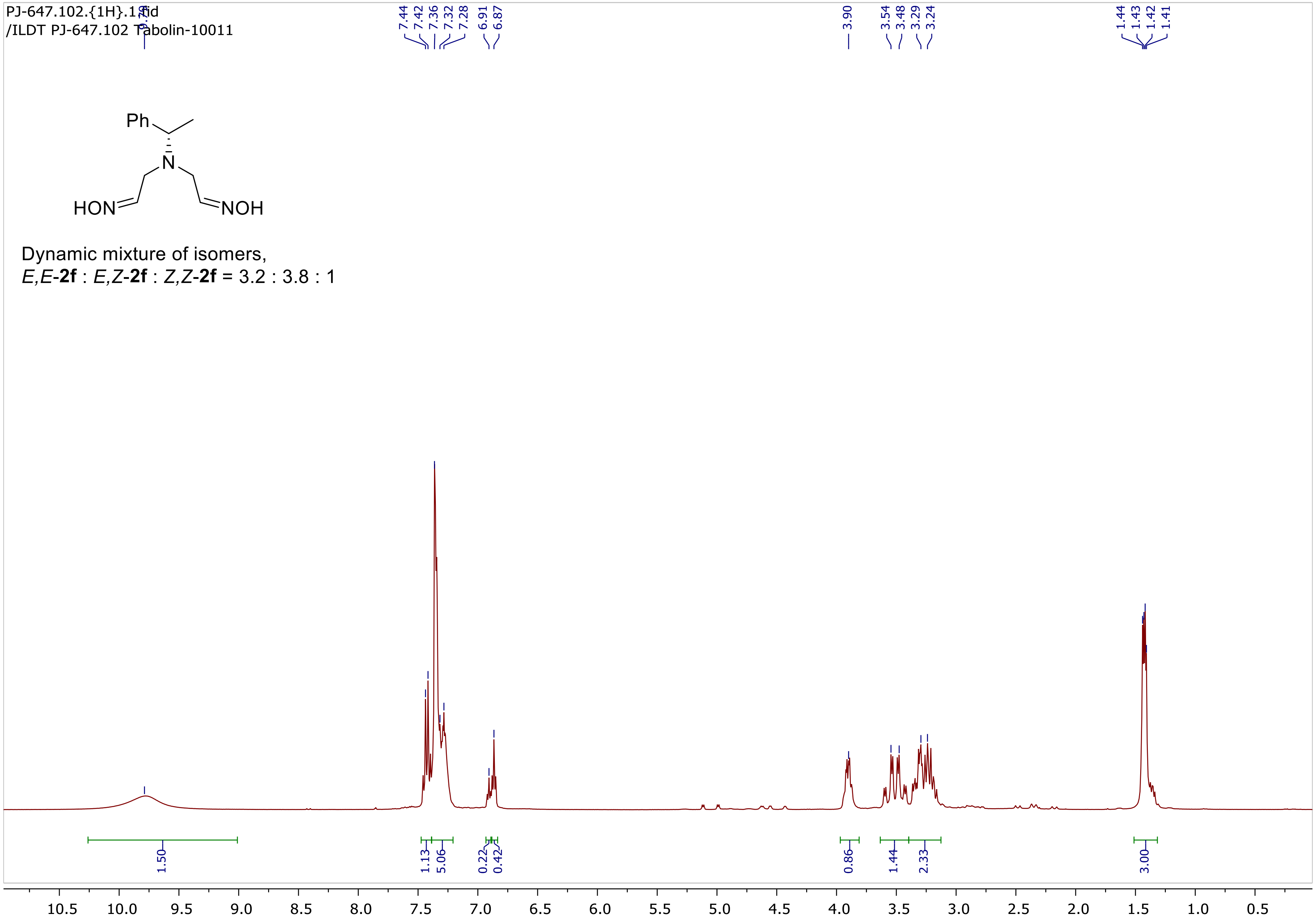
PJ-647.102.{1H}.1.fid
/ILDT PJ-647.102 Tabolin-10011
Ph
N
HON
NOH
Dynamic mixture of isomers,
E,E-**2f** : *E,Z*-**2f** : *Z,Z*-**2f** = 3.2 : 3.8 : 1
9.78
7.44
7.42
7.36
7.32
7.28
6.91
6.87
3.90
3.54
3.48
3.29
3.24
1.44
1.43
1.42
1.41
1.50
1.13
5.06
0.22
0.42
0.86
1.44
2.33
3.00
10.5
10.0
9.5
9.0
8.5
8.0
7.5
7.0
6.5
6.0
5.5
5.0
4.5
4.0
3.5
3.0
2.5
2.0
1.5
1.0
0.5

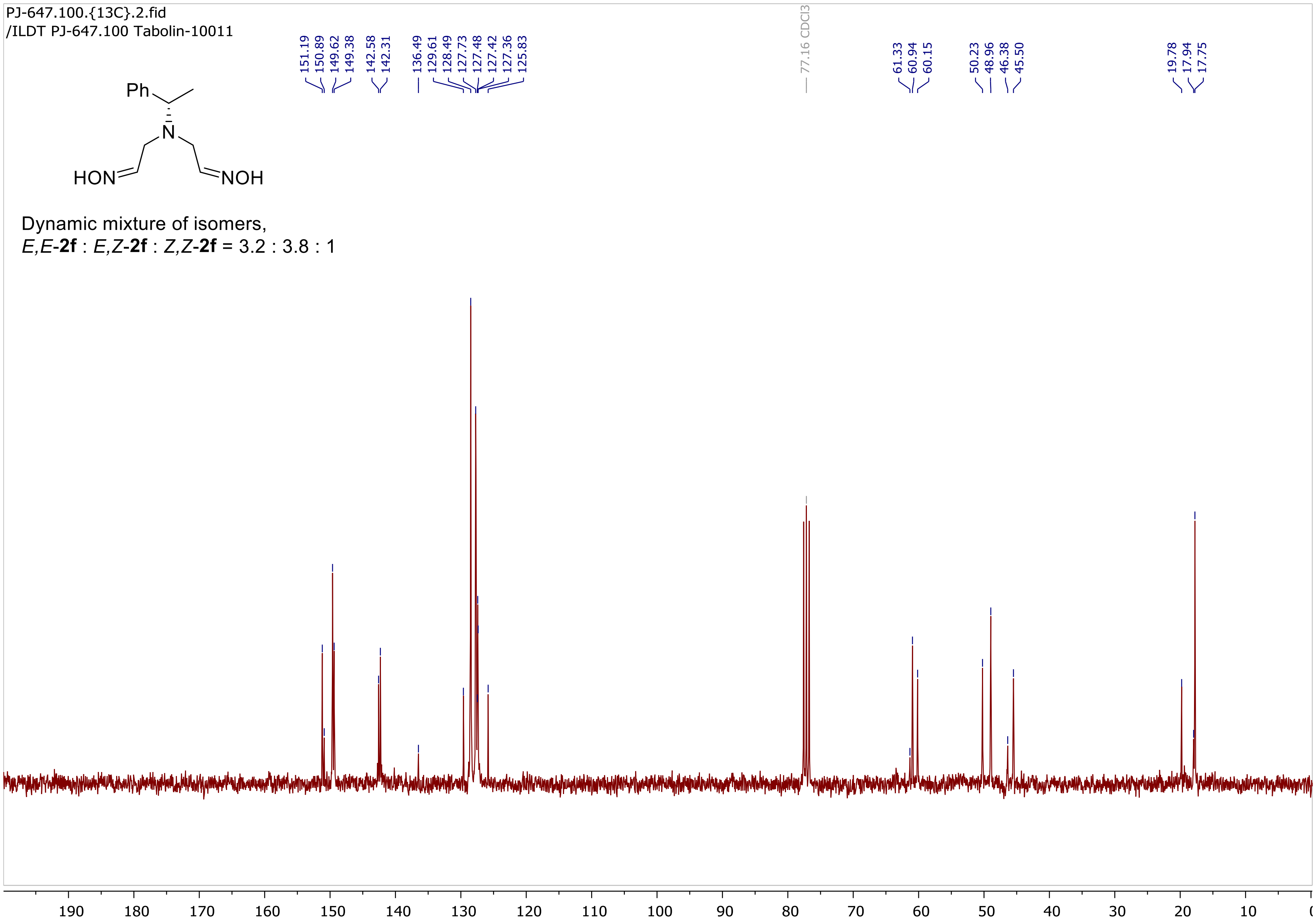
PJ-647.100.{13C}.2.fid
/ILDT PJ-647.100 Tabolin-10011
151.19
150.89
149.62
149.38
142.58
142.31
136.49
129.61
128.49
127.73
127.48
127.42
127.36
125.83
77.16 CDCl3
61.33
60.94
60.15
50.23
48.96
46.38
45.50
19.78
17.94
17.75
Ph
N
HON
NOH
Dynamic mixture of isomers,
E,*E*-**2f** : *E*,*Z*-**2f** : *Z*,*Z*-**2f** = 3.2 : 3.8 : 1
190
180
170
160
150
140
130
120
110
100
90
80
70
60
50
40
30
20
10

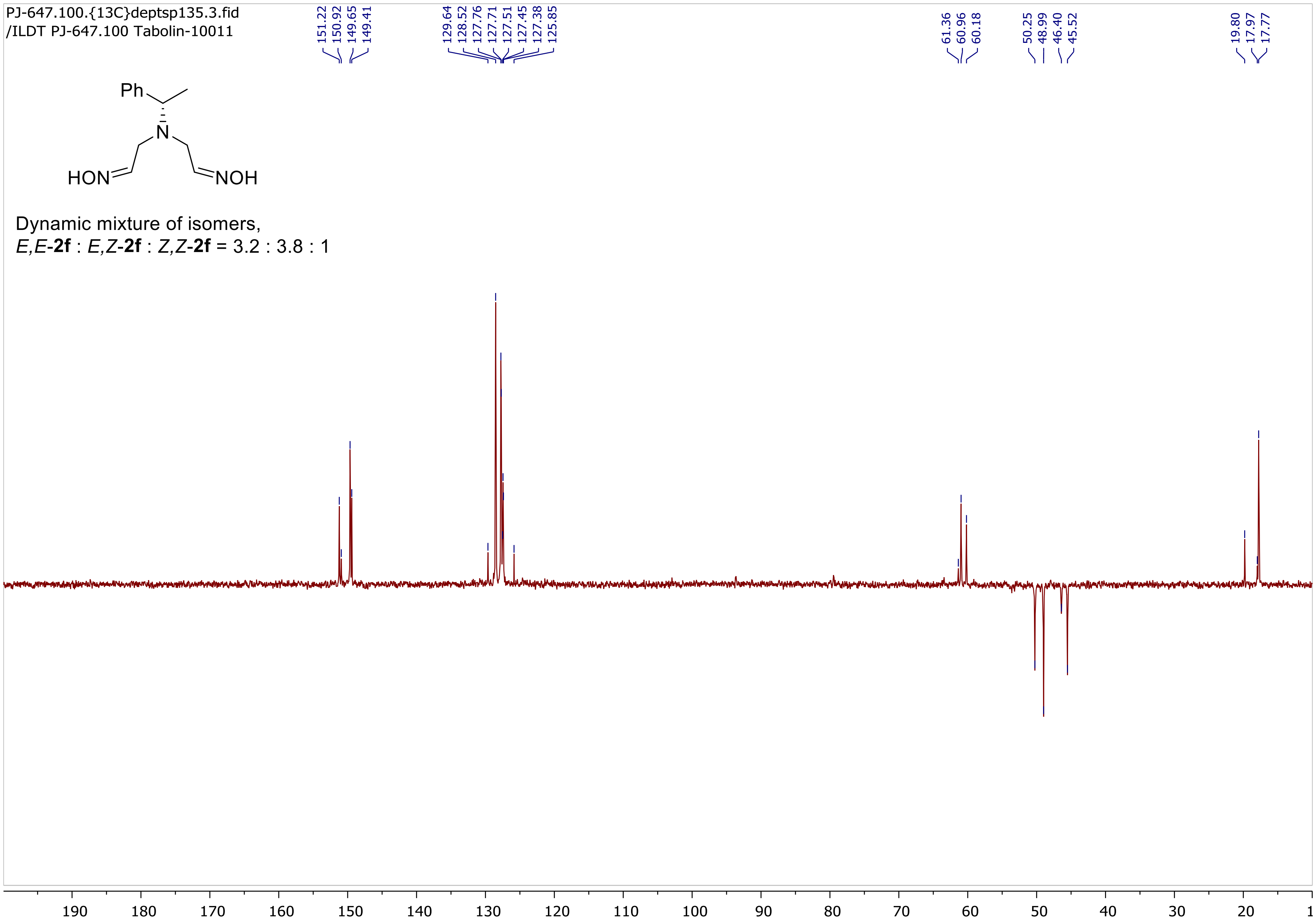
PJ-647.100.{13C}deptsp135.3.fid
/ILDT PJ-647.100 Tabolin-10011
151.22
150.92
149.65
149.41
129.64
128.52
127.76
127.71
127.51
127.45
127.38
125.85
61.36
60.96
60.18
50.25
48.99
46.40
45.52
19.80
17.97
17.77
Ph
N
HON
NOH
Dynamic mixture of isomers,
E,E-**2f** : *E,Z*-**2f** : *Z,Z*-**2f** = 3.2 : 3.8 : 1
190
180
170
160
150
140
130
120
110
100
90
80
70
60
50
40
30
20
1

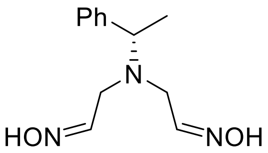

Dynamic mixture of isomers,
E,*E*-**2f** : *E*,*Z*-**2f** : *Z*,*Z*-**2f** = 3.2 : 3.8 : 1

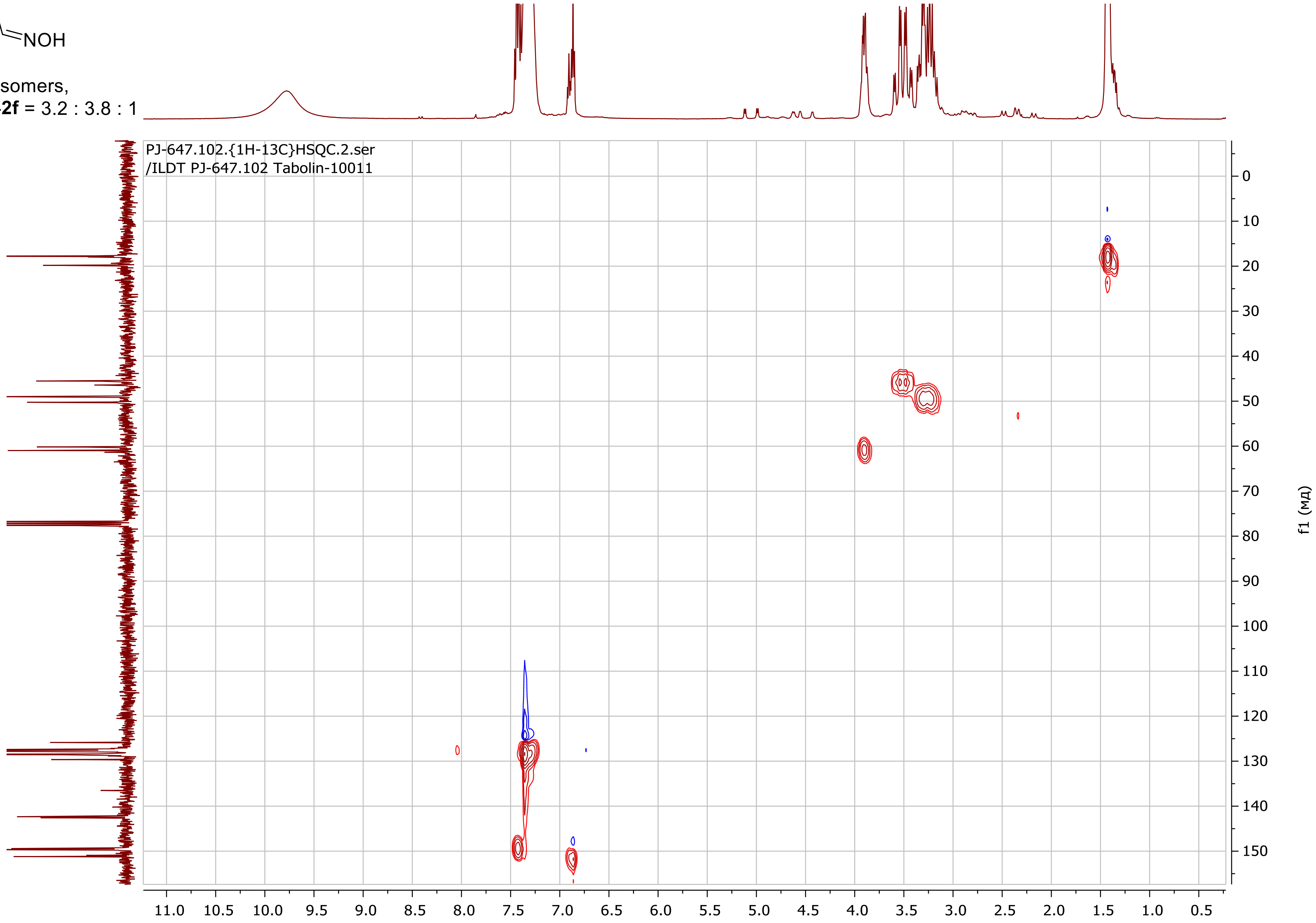

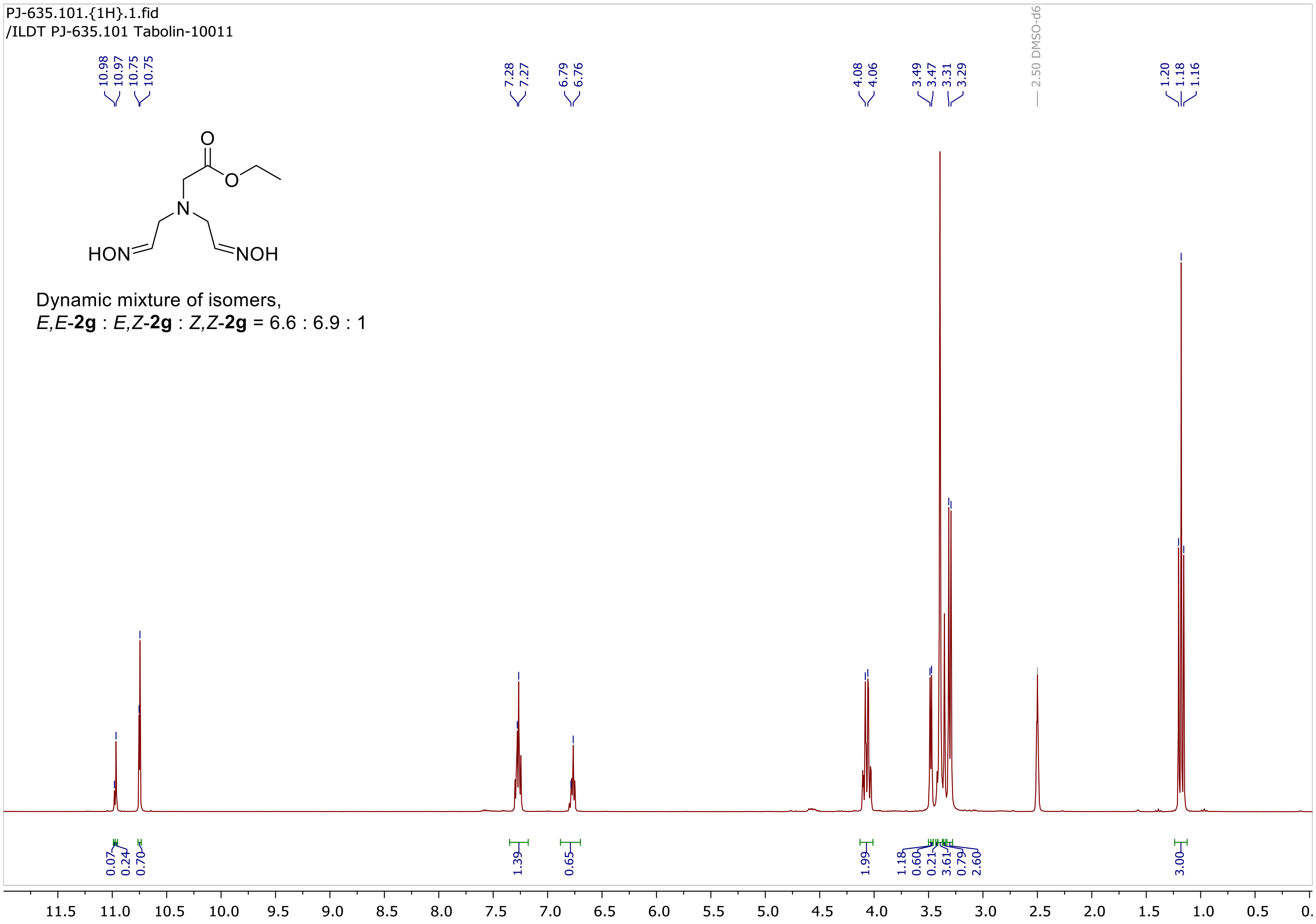
PJ-635.101.{1H}.1.fid
/ILDT PJ-635.101 Tabolin-10011
10.98
10.97
10.75
10.75
7.28
7.27
6.79
6.76
4.08
4.06
3.49
3.47
3.31
3.29
2.50 DMSO-d6
1.20
1.18
1.16
O
O
N
HON
NOH
Dynamic mixture of isomers,
E,E-2g : E,Z-2g : Z,Z-2g = 6.6 : 6.9 : 1
0.07
0.24
0.70
1.39
0.65
1.99
1.18
0.60
0.21
3.61
0.79
2.60
3.00
11.5
11.0
10.5
10.0
9.5
9.0
8.5
8.0
7.5
7.0
6.5
6.0
5.5
5.0
4.5
4.0
3.5
3.0
2.5
2.0
1.5
1.0
0.5
0.

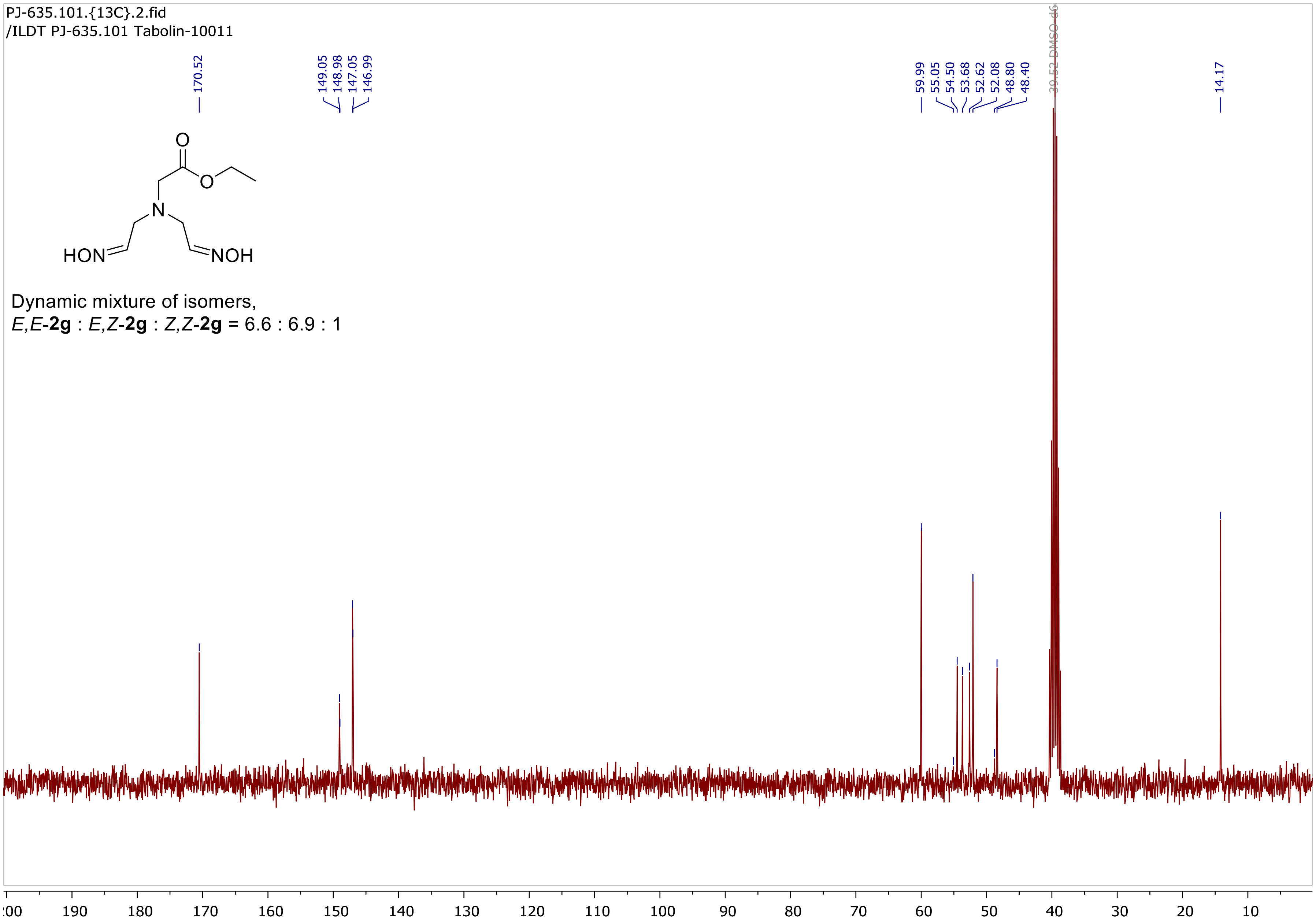
PJ-635.101.{13C}.2.fid
/ILDT PJ-635.101 Tabolin-10011
170.52
149.05
148.98
147.05
146.99
59.99
55.05
54.50
53.68
52.62
52.08
48.80
48.40
39.52 DMSO-d6
14.17
O
O
N
HON
NOH
Dynamic mixture of isomers,
E,*E*-**2g** : *E*,*Z*-**2g** : *Z*,*Z*-**2g** = 6.6 : 6.9 : 1
190
180
170
160
150
140
130
120
110
100
90
80
70
60
50
40
30
20
10

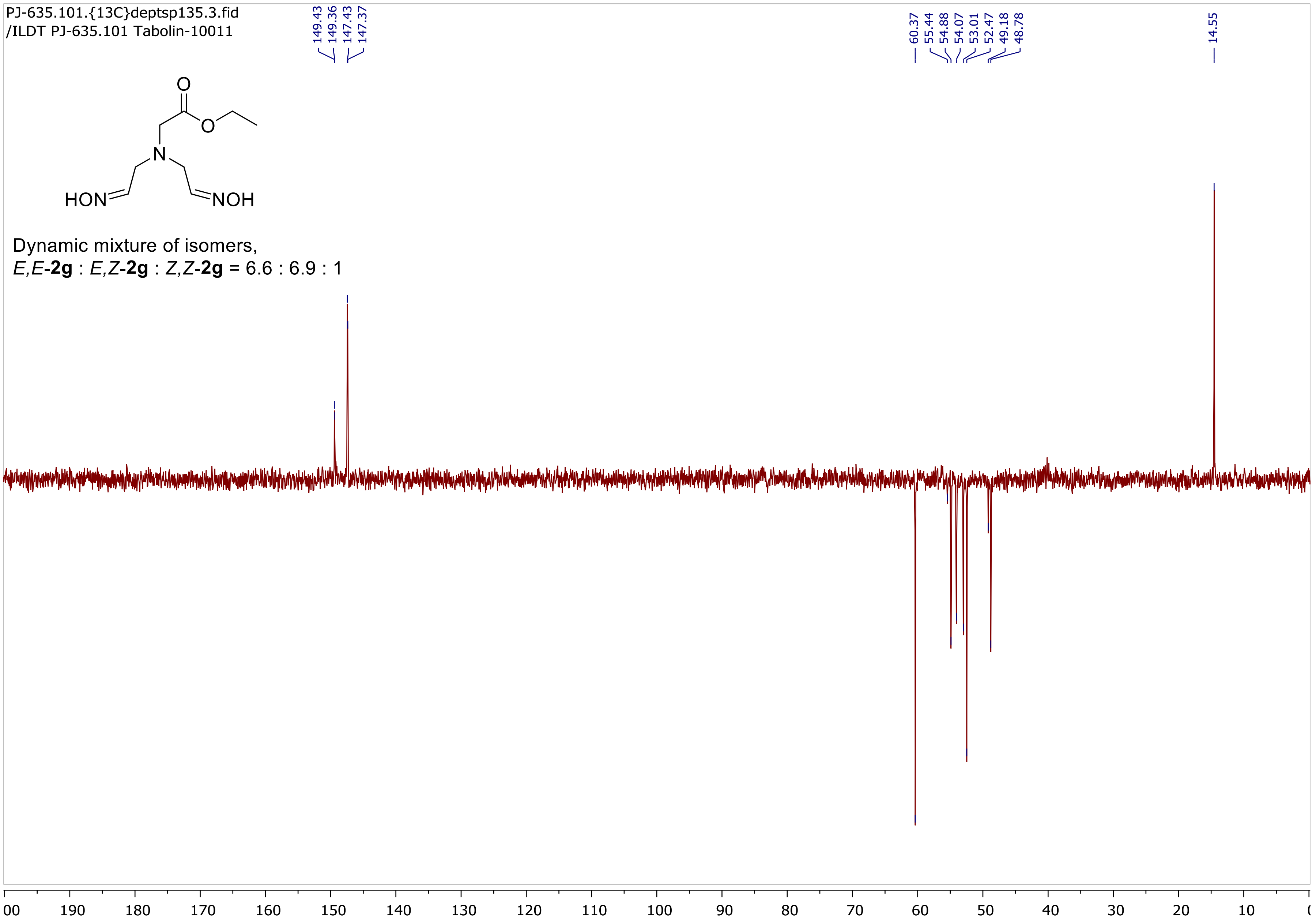
PJ-635.101.{13C}deptsp135.3.fid
/ILDT PJ-635.101 Tabolin-10011
149.43
149.36
147.43
147.37
60.37
55.44
54.88
54.07
53.01
52.47
49.18
48.78
14.55
O
O
N
HON
NOH
Dynamic mixture of isomers,
E,E-**2g** : *E,Z*-**2g** : *Z,Z*-**2g** = 6.6 : 6.9 : 1
00
190
180
170
160
150
140
130
120
110
100
90
80
70
60
50
40
30
20
10

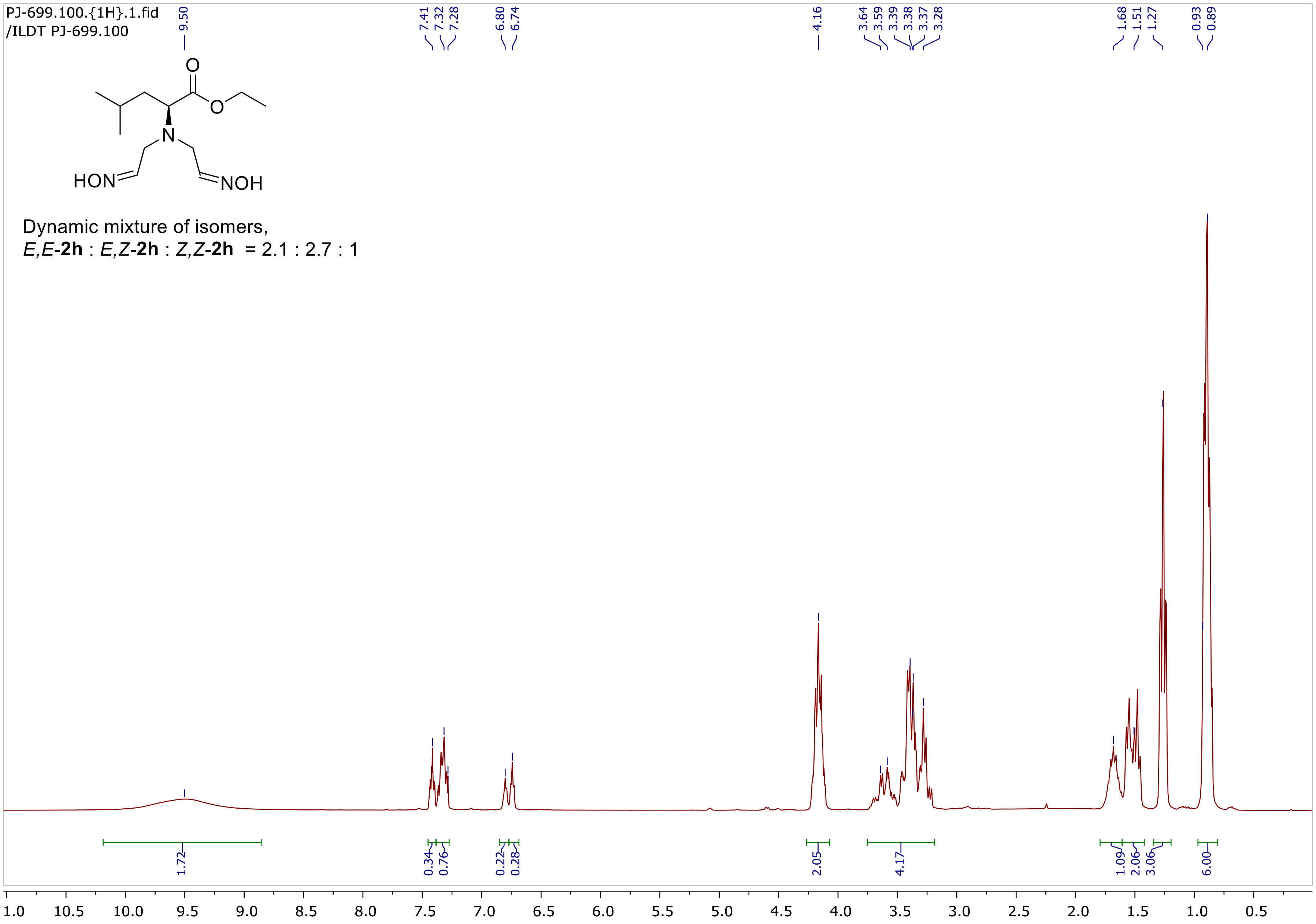
PJ-699.100.{1H}.1.fid
/ILDT PJ-699.100
9.50
7.41
7.32
7.28
6.80
6.74
4.16
3.64
3.59
3.39
3.38
3.37
3.28
1.68
1.51
1.27
0.93
0.89
O
O
N
HON
NOH
Dynamic mixture of isomers,
E,E-**2h** : *E,Z*-**2h** : *Z,Z*-**2h** = 2.1 : 2.7 : 1
1.72
0.34
0.76
0.22
0.28
2.05
4.17
1.09
2.06
3.06
6.00
1.0
10.5
10.0
9.5
9.0
8.5
8.0
7.5
7.0
6.5
6.0
5.5
5.0
4.5
4.0
3.5
3.0
2.5
2.0
1.5
1.0
0.5

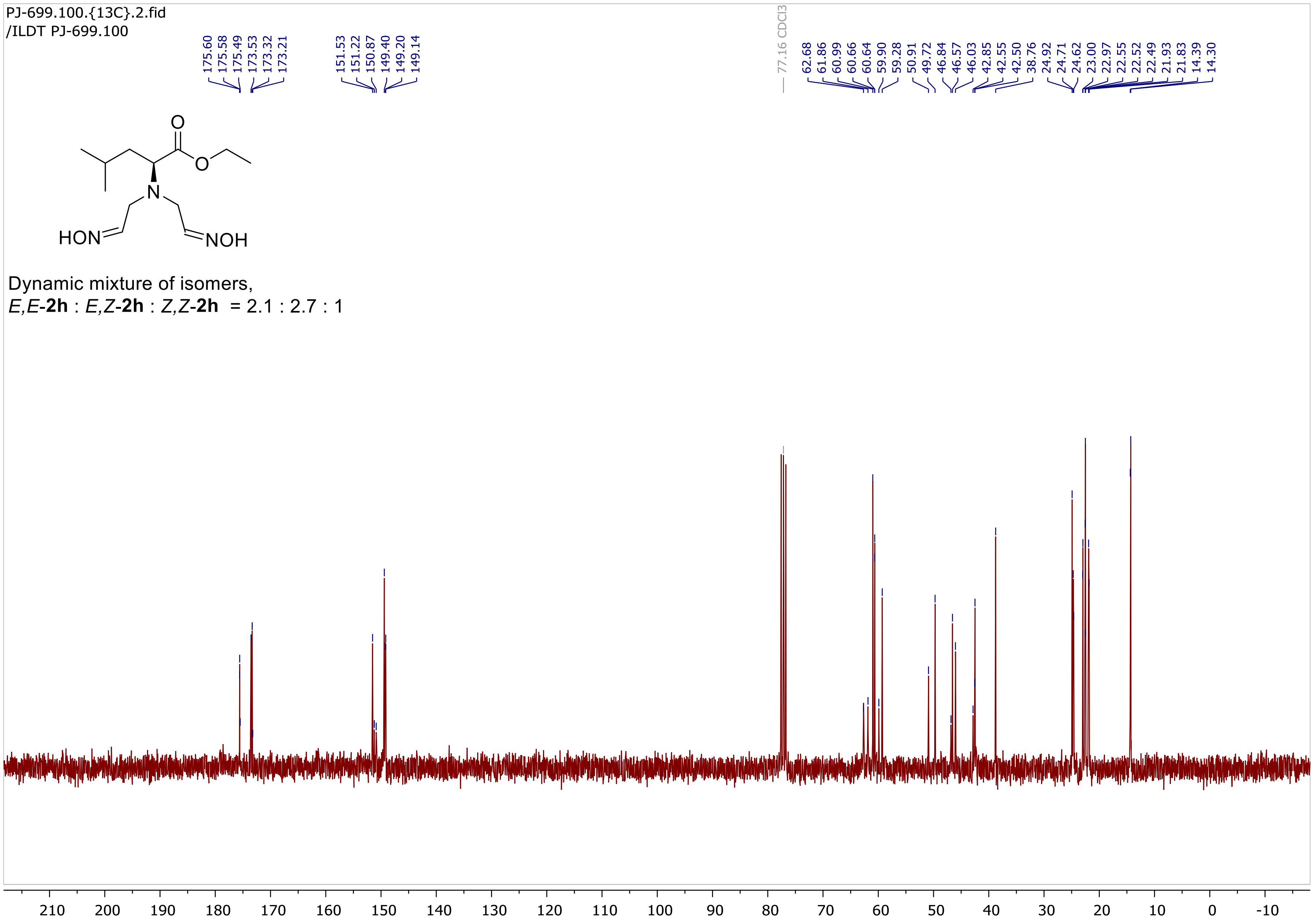
PJ-699.100.{13C}.2.fid
/ILDT PJ-699.100
175.60
175.58
175.49
173.53
173.32
173.21
151.53
151.22
150.87
149.40
149.20
149.14
77.16 CDCl3
62.68
61.86
60.99
60.66
60.64
59.90
59.28
50.91
49.72
46.84
46.57
46.03
42.85
42.55
42.50
38.76
24.92
24.71
24.62
23.00
22.97
22.55
22.52
22.49
21.93
21.83
14.39
14.30
O
O
N
HON
NOH
Dynamic mixture of isomers,
E,E-2h : E,Z-2h : Z,Z-2h = 2.1 : 2.7 : 1
210
200
190
180
170
160
150
140
130
120
110
100
90
80
70
60
50
40
30
20
10
0
-10

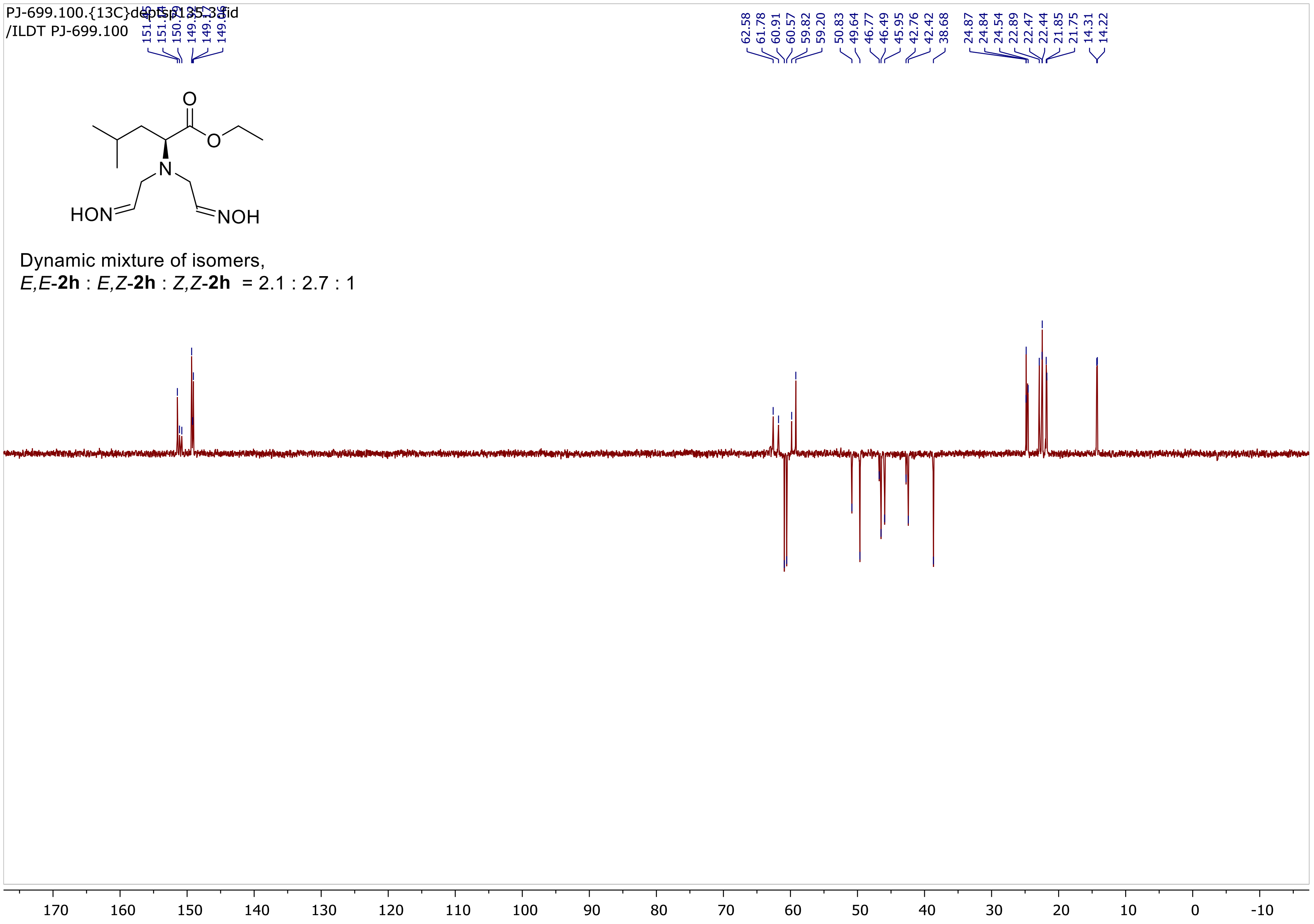
PJ-699.100.{13C}deptsp135.3.fid
/ILDT PJ-699.100
151.45
151.14
150.79
149.32
149.17
149.06
62.58
61.78
60.91
60.57
59.82
59.20
50.83
49.64
46.77
46.49
45.95
42.76
42.42
38.68
24.87
24.84
24.54
22.89
22.47
22.44
21.85
21.75
14.31
14.22
O
O
N
HON
NOH
Dynamic mixture of isomers,
E,E-**2h** : *E,Z*-**2h** : *Z,Z*-**2h** = 2.1 : 2.7 : 1
170
160
150
140
130
120
110
100
90
80
70
60
50
40
30
20
10
0
-10

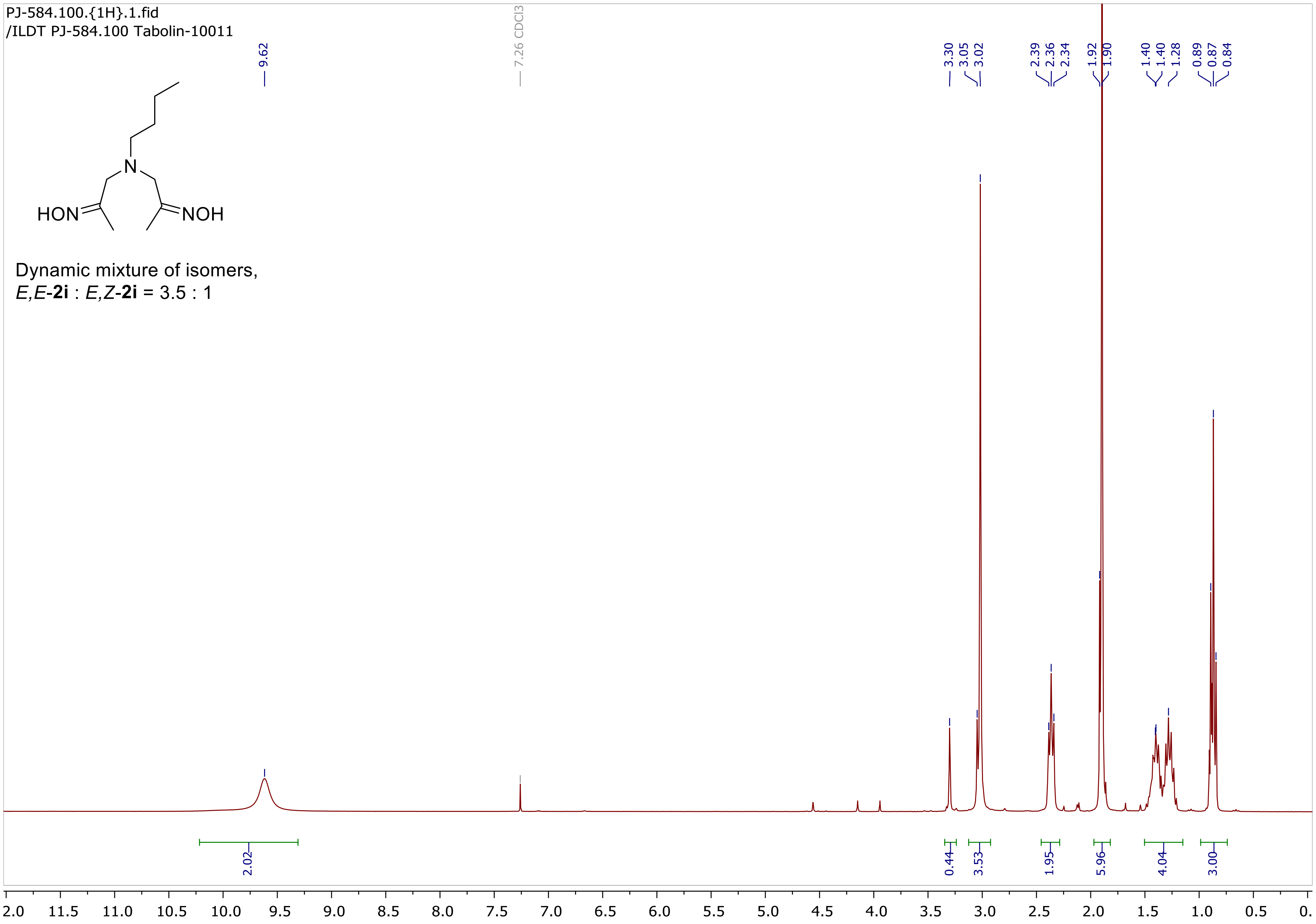

PJ-584.100.{1H}.1.fid
/ILDT PJ-584.100 Tabolin-10011
HON
N
NOH
Dynamic mixture of isomers,
E,*E*-**2i** : *E*,*Z*-**2i** = 3.5 : 1
9.62
7.26 CDCl3
3.30
3.05
3.02
2.39
2.36
2.34
1.92
1.90
1.40
1.40
1.28
0.89
0.87
0.84
2.02
0.44
3.53
1.95
5.96
4.04
3.00

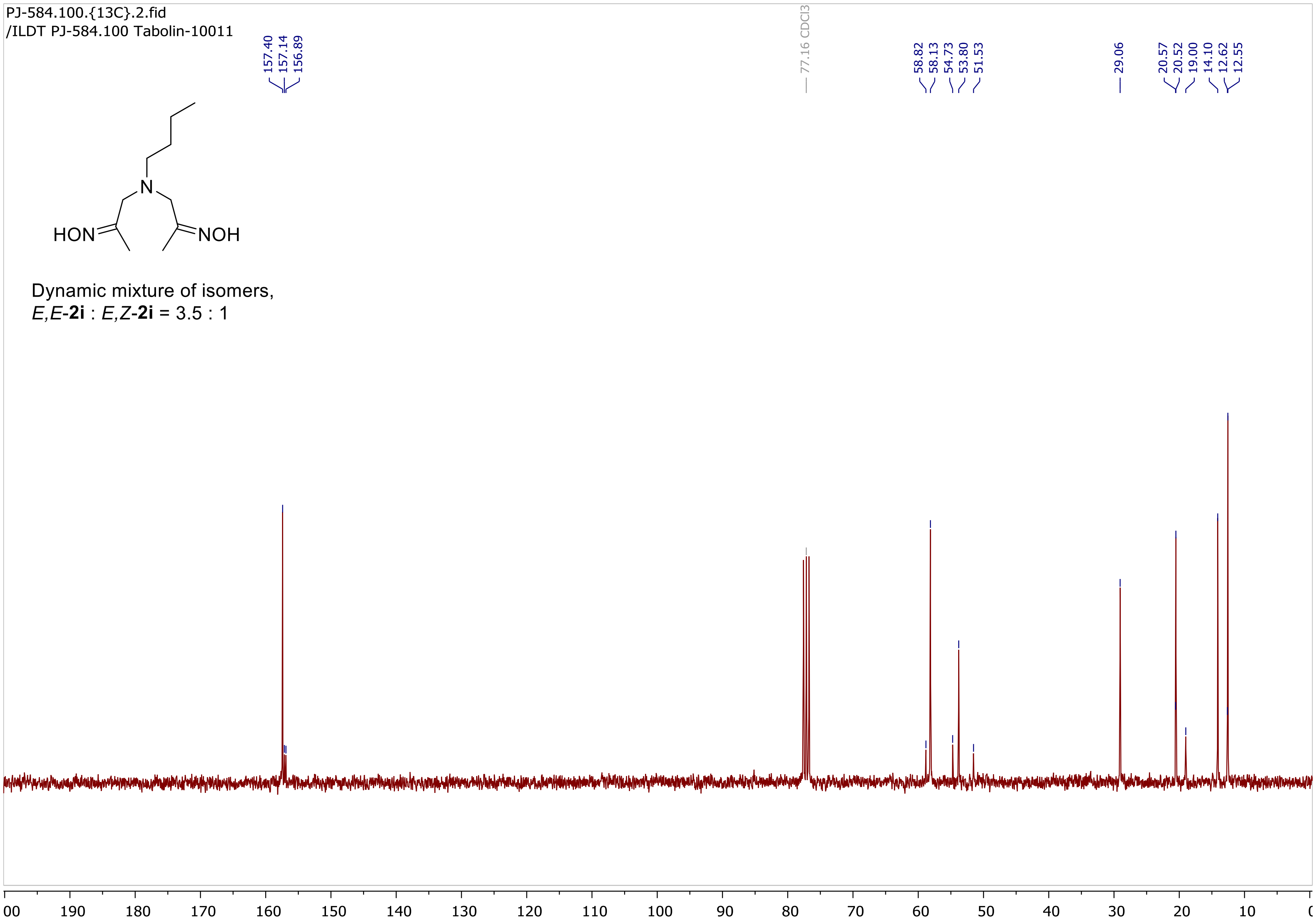
PJ-584.100.{13C}.2.fid
/ILDT PJ-584.100 Tabolin-10011
157.40
157.14
156.89
77.16 CDCl3
58.82
58.13
54.73
53.80
51.53
29.06
20.57
20.52
19.00
14.10
12.62
12.55
N
HON
NOH
Dynamic mixture of isomers,
E,*E*-**2i** : *E*,*Z*-**2i** = 3.5 : 1
190
180
170
160
150
140
130
120
110
100
90
80
70
60
50
40
30
20
10

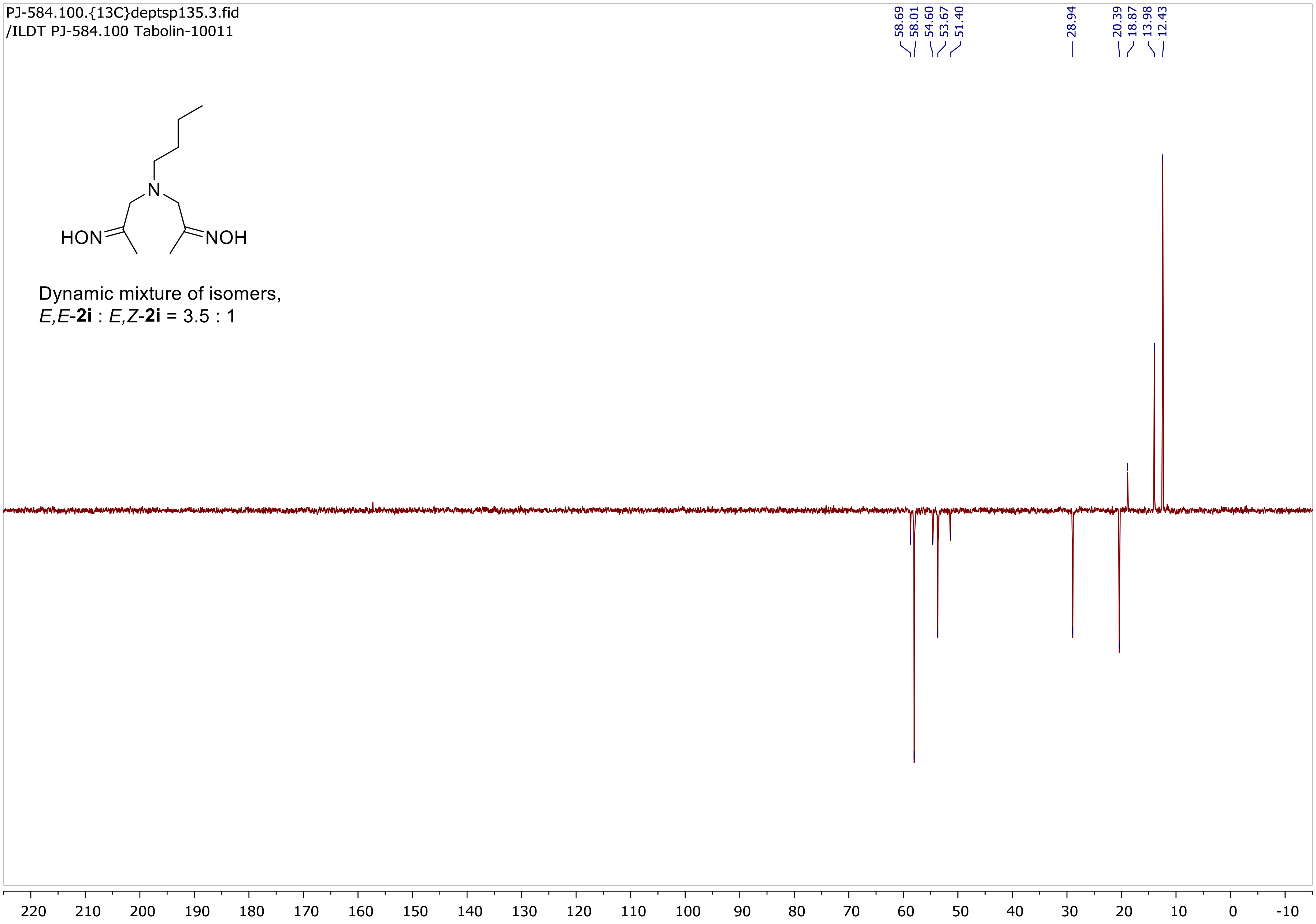
PJ-584.100.{13C}deptsp135.3.fid
/ILDT PJ-584.100 Tabolin-10011
58.69
58.01
54.60
53.67
51.40
28.94
20.39
18.87
13.98
12.43
HON
N
NOH
Dynamic mixture of isomers,
E,*E*-**2i** : *E*,*Z*-**2i** = 3.5 : 1
220 210 200 190 180 170 160 150 140 130 120 110 100 90 80 70 60 50 40 30 20 10 0 -10

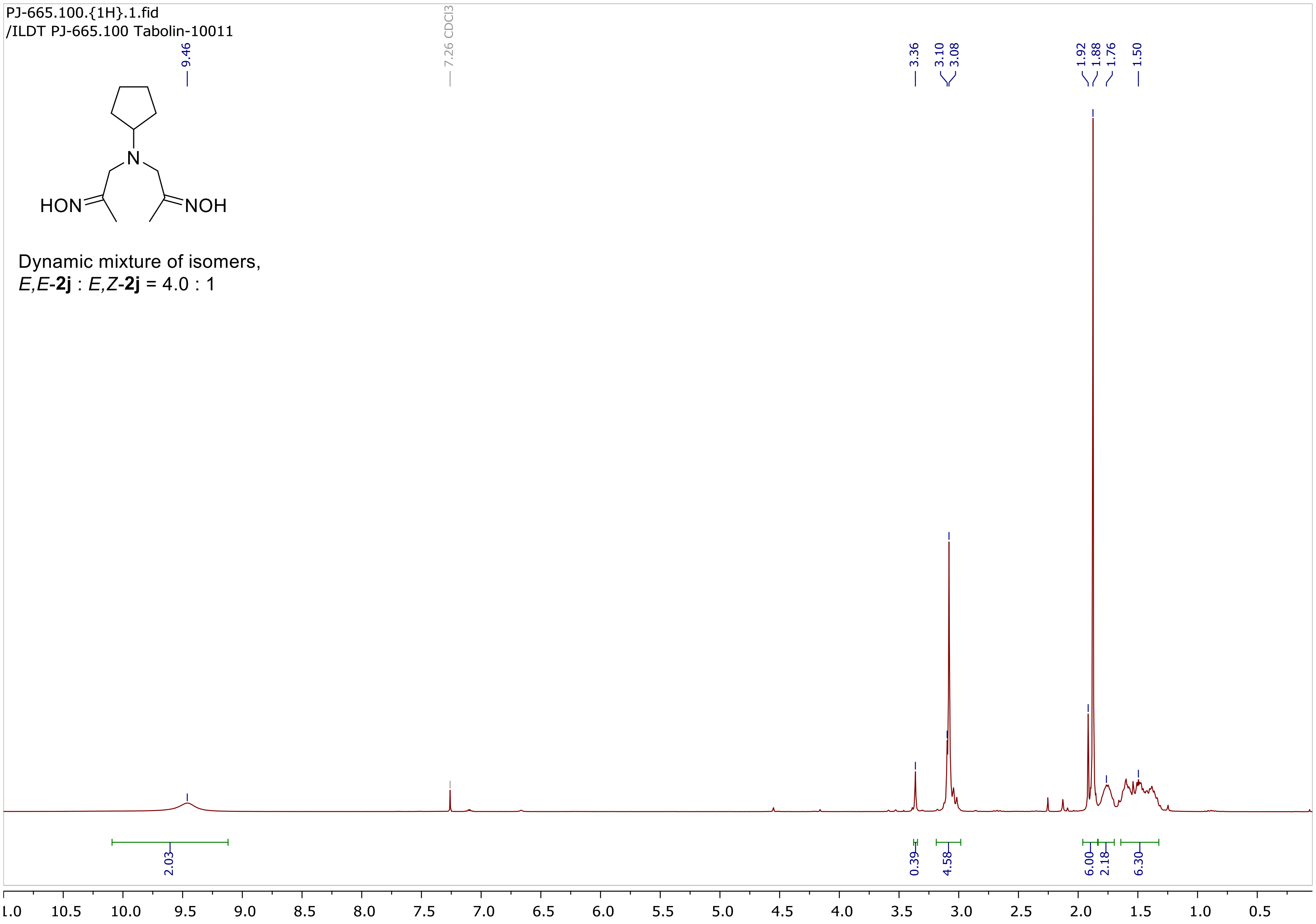
PJ-665.100.{1H}.1.fid
/ILDT PJ-665.100 Tabolin-10011
HON
N
NOH
Dynamic mixture of isomers,
E,E-2j : E,Z-2j = 4.0 : 1
9.46
7.26 CDCl3
3.36
3.10
3.08
1.92
1.88
1.76
1.50
2.03
0.39
4.58
6.00
2.18
6.30
10.5
10.0
9.5
9.0
8.5
8.0
7.5
7.0
6.5
6.0
5.5
5.0
4.5
4.0
3.5
3.0
2.5
2.0
1.5
1.0
0.5

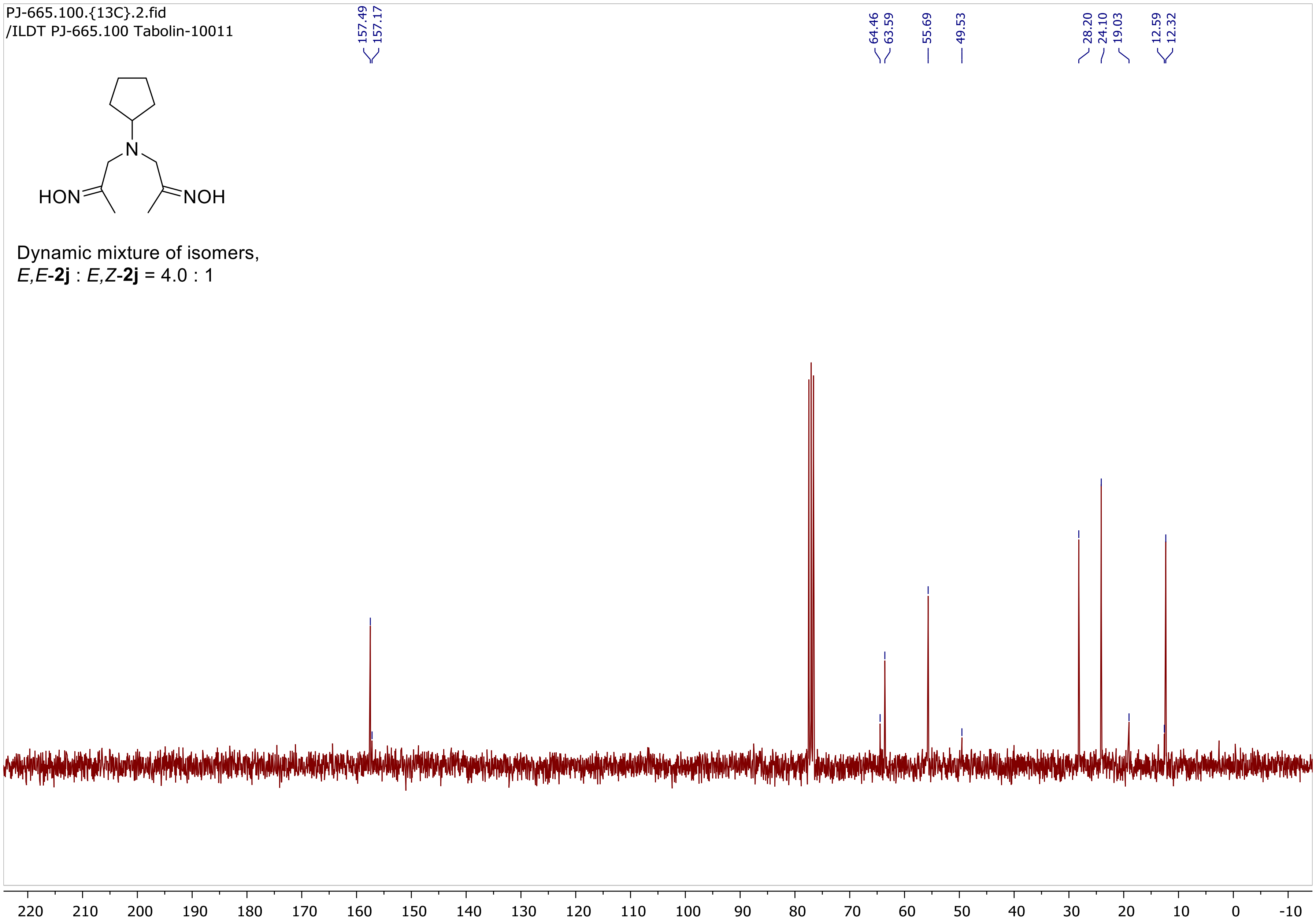
PJ-665.100.{13C}.2.fid
/ILDT PJ-665.100 Tabolin-10011
HON
N
NOH
Dynamic mixture of isomers,
E,*E*-**2j** : *E*,*Z*-**2j** = 4.0 : 1
157.49
157.17
64.46
63.59
55.69
49.53
28.20
24.10
19.03
12.59
12.32
220 210 200 190 180 170 160 150 140 130 120 110 100 90 80 70 60 50 40 30 20 10 0 -10

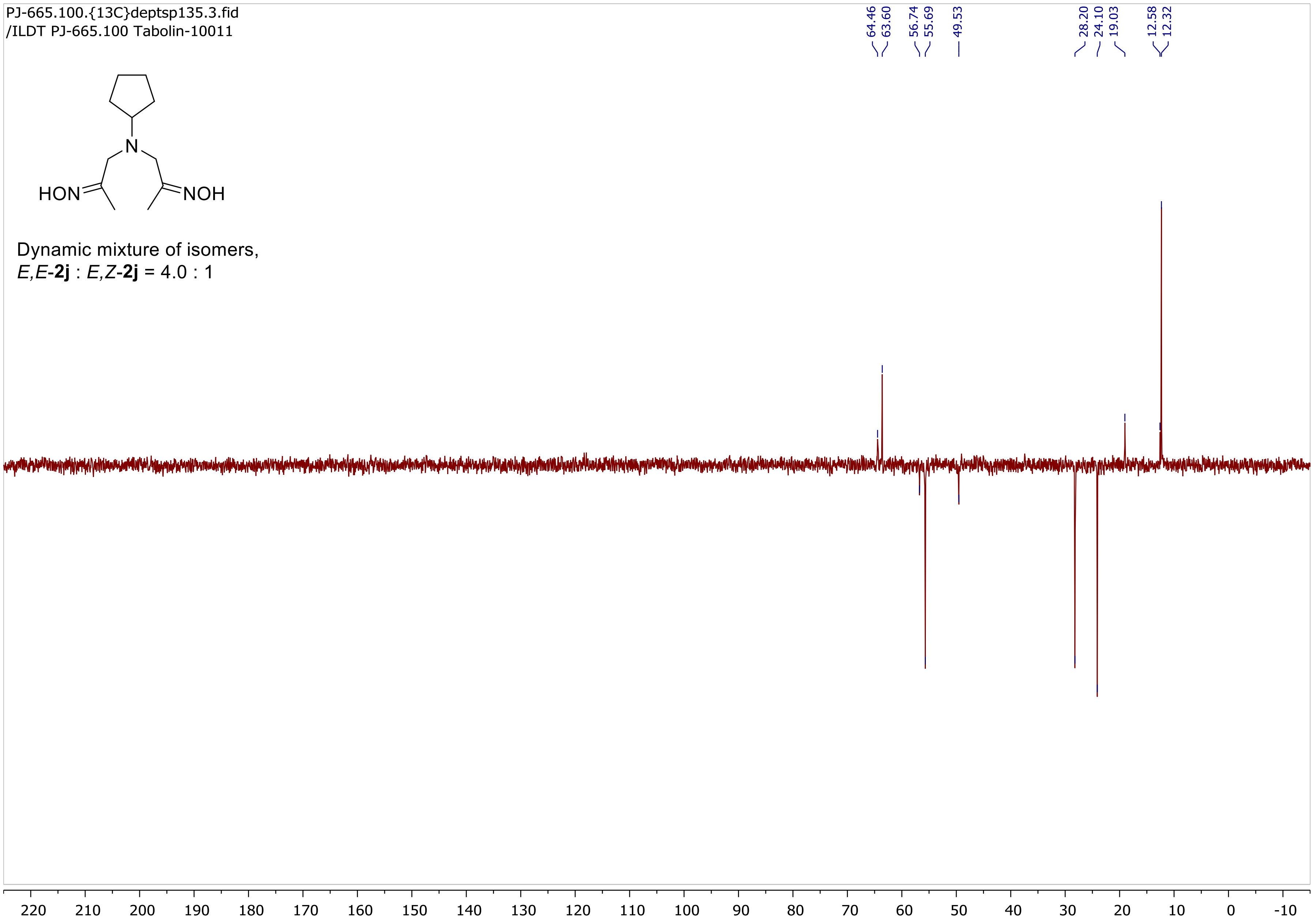
PJ-665.100.{13C}deptsp135.3.fid
/ILDT PJ-665.100 Tabolin-10011
HON
N
NOH
Dynamic mixture of isomers,
E,*E*-**2j** : *E*,*Z*-**2j** = 4.0 : 1
64.46
63.60
56.74
55.69
49.53
28.20
24.10
19.03
12.58
12.32
220 210 200 190 180 170 160 150 140 130 120 110 100 90 80 70 60 50 40 30 20 10 0 -10

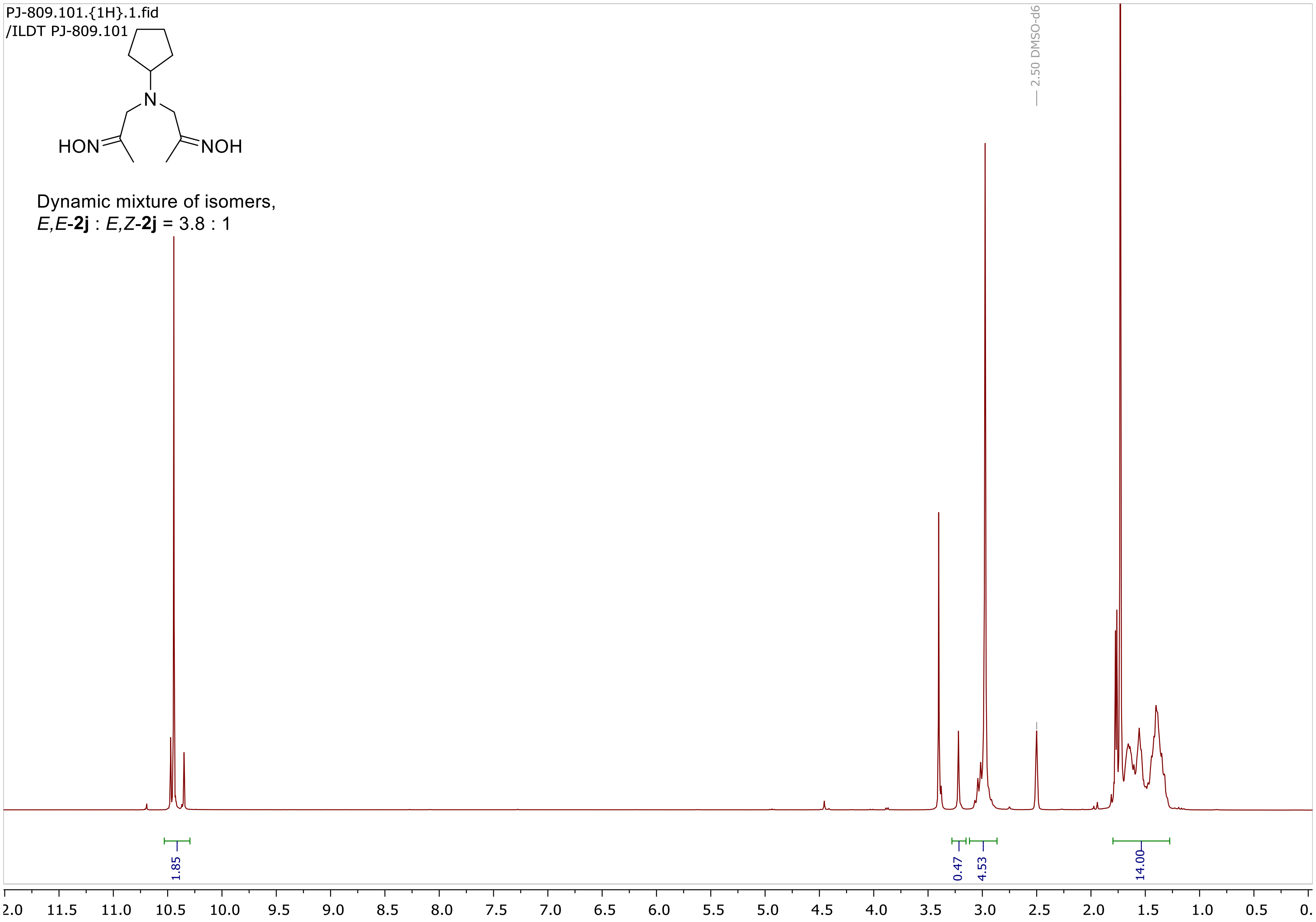

PJ-809.101.{1H}.1.fid
/ILDT PJ-809.101
N
HON
NOH
Dynamic mixture of isomers,
E,*E*-**2j** : *E*,*Z*-**2j** = 3.8 : 1
2.50 DMSO-d6
1.85
0.47
4.53
14.00
2.0 11.5 11.0 10.5 10.0 9.5 9.0 8.5 8.0 7.5 7.0 6.5 6.0 5.5 5.0 4.5 4.0 3.5 3.0 2.5 2.0 1.5 1.0 0.5 0.

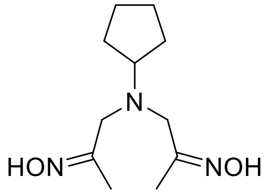

Dynamic mixture of isomers,
E,*E*-**2j** : *E*,*Z*-**2j** = 3.8 : 1

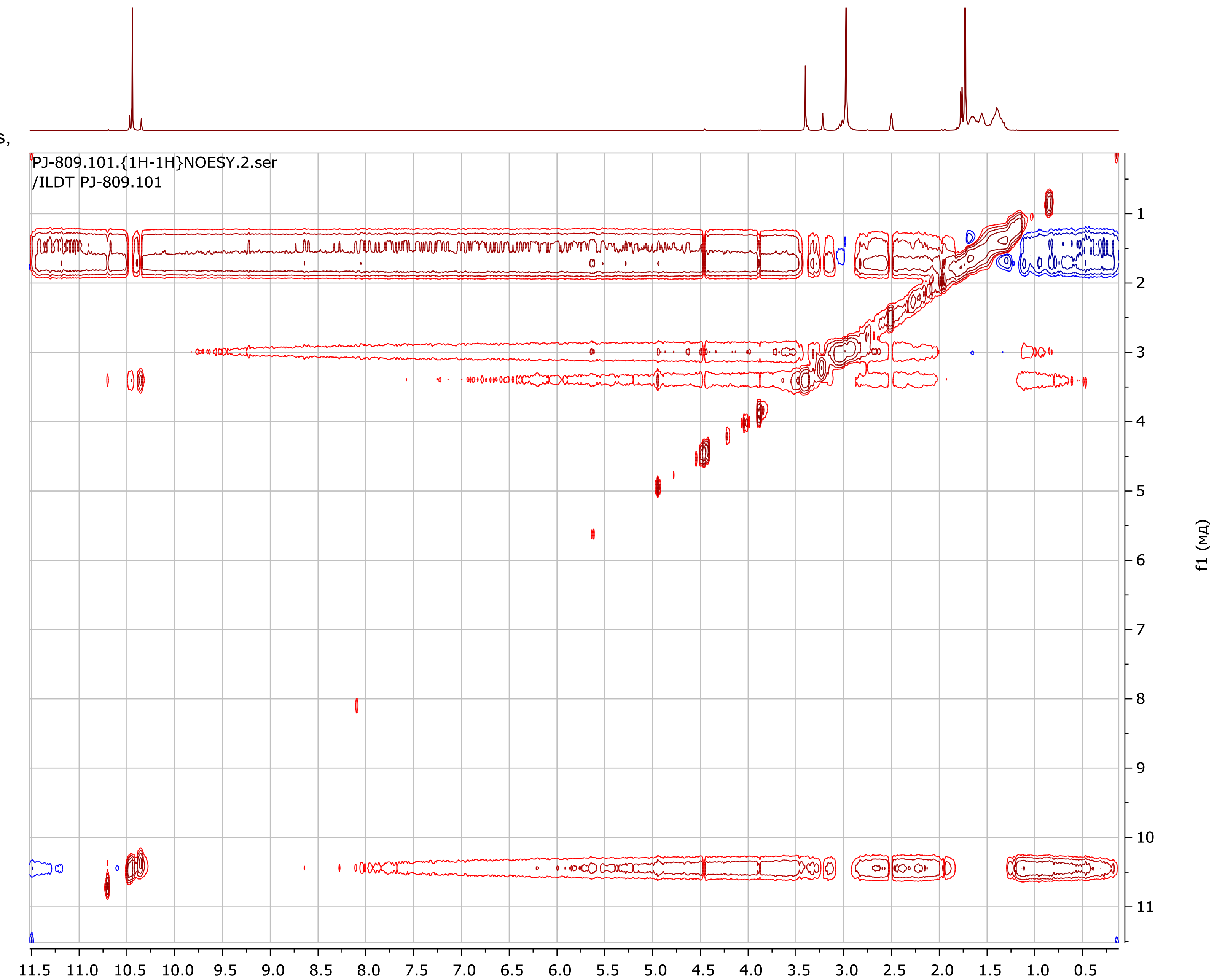

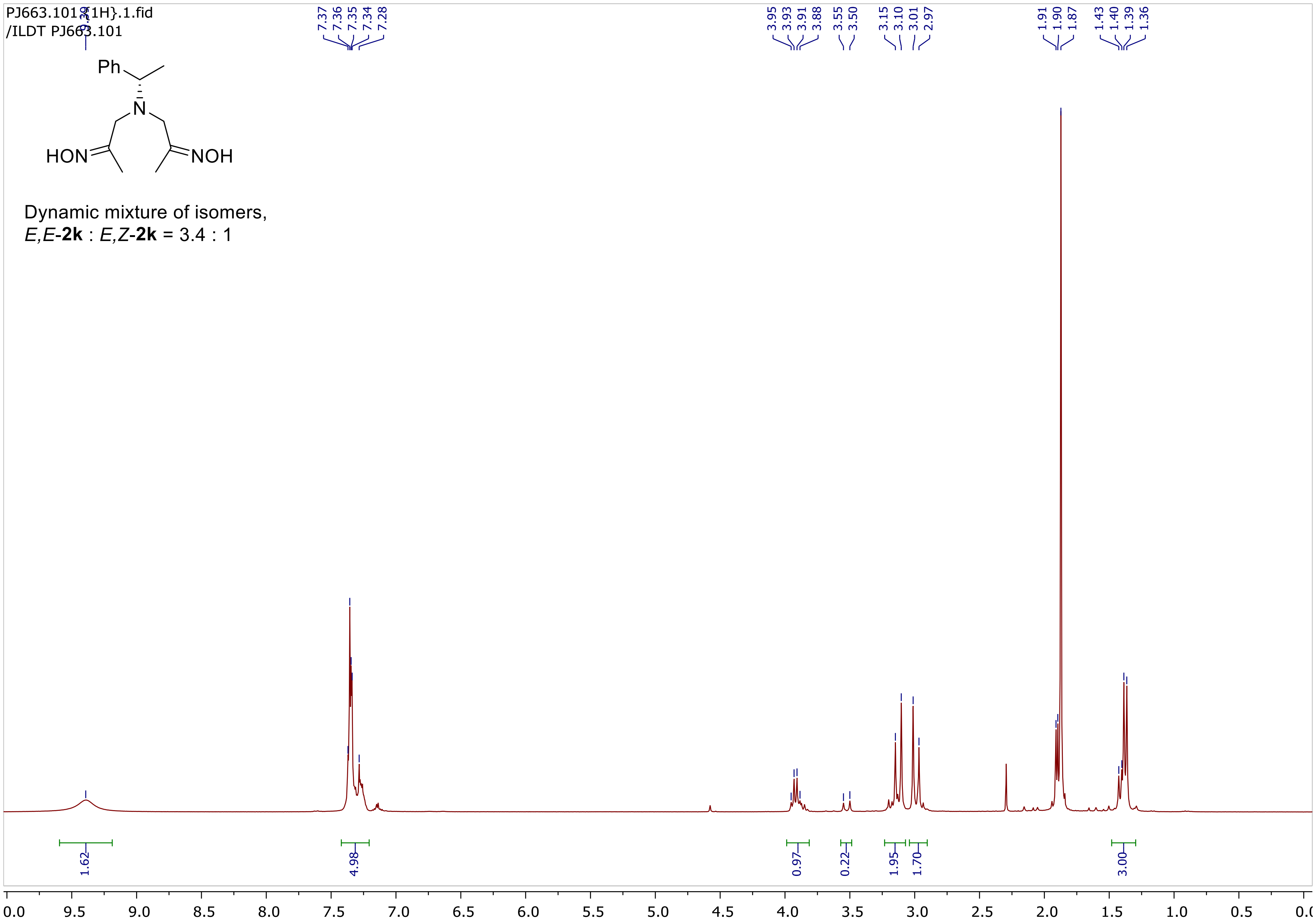
PJ663.101{1H}.1.fid
/ILDT PJ663.101
Ph
N
HON
NOH
Dynamic mixture of isomers,
E,*E*-**2k** : *E*,*Z*-**2k** = 3.4 : 1
7.37 7.36 7.35 7.34 7.28
3.95 3.93 3.91 3.88 3.55 3.50 3.15 3.10 3.01 2.97
1.91 1.90 1.87 1.43 1.40 1.39 1.36
1.62 4.98 0.97 0.22 1.95 1.70 3.00
0.0 9.5 9.0 8.5 8.0 7.5 7.0 6.5 6.0 5.5 5.0 4.5 4.0 3.5 3.0 2.5 2.0 1.5 1.0 0.5

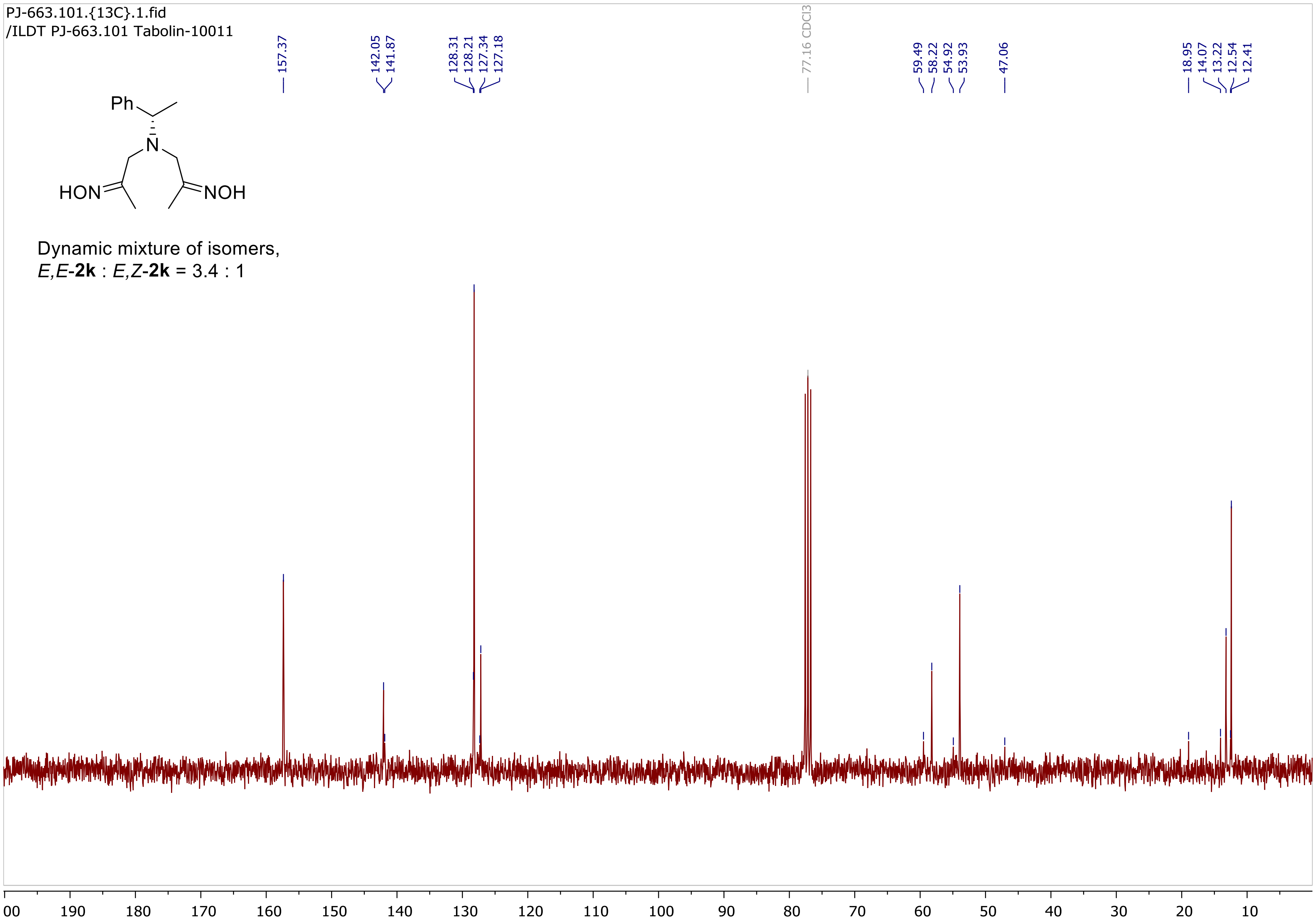
PJ-663.101.{13C}.1.fid
/ILDT PJ-663.101 Tabolin-10011
157.37
142.05
141.87
128.31
128.21
127.34
127.18
77.16 CDCl3
59.49
58.22
54.92
53.93
47.06
18.95
14.07
13.22
12.54
12.41
Ph
N
HON
NOH
Dynamic mixture of isomers,
E,*E*-**2k** : *E*,*Z*-**2k** = 3.4 : 1
00
190
180
170
160
150
140
130
120
110
100
90
80
70
60
50
40
30
20
10

PJ-663.101.{13C}deptsp135.2.fid
/ILDT PJ-663.101 Tabolin-10011

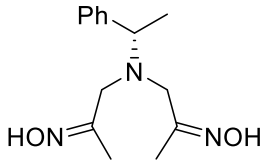

Dynamic mixture of isomers,
E,*E*-**2k** : *E*,*Z*-**2k** = 3.4 : 1

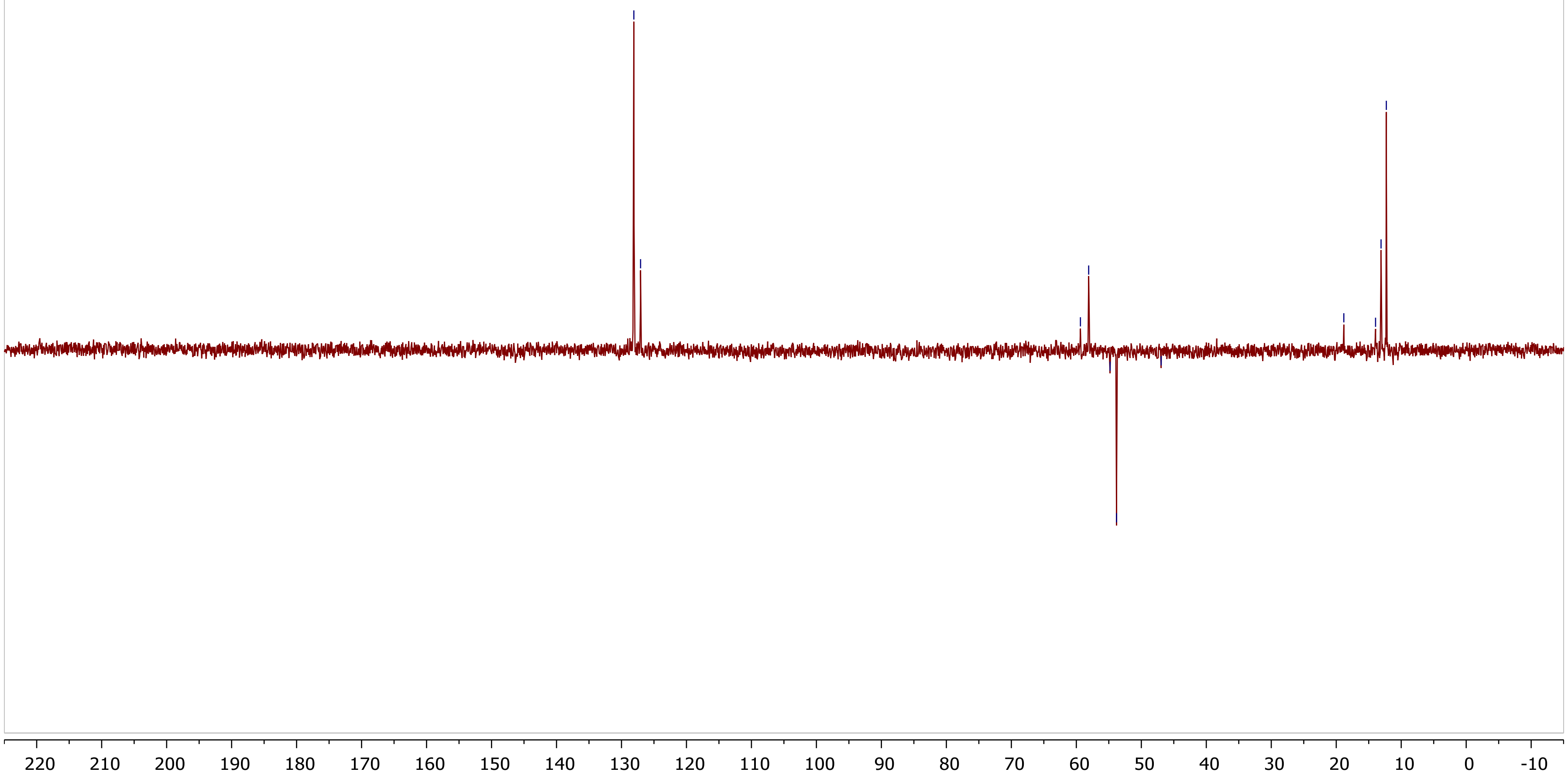

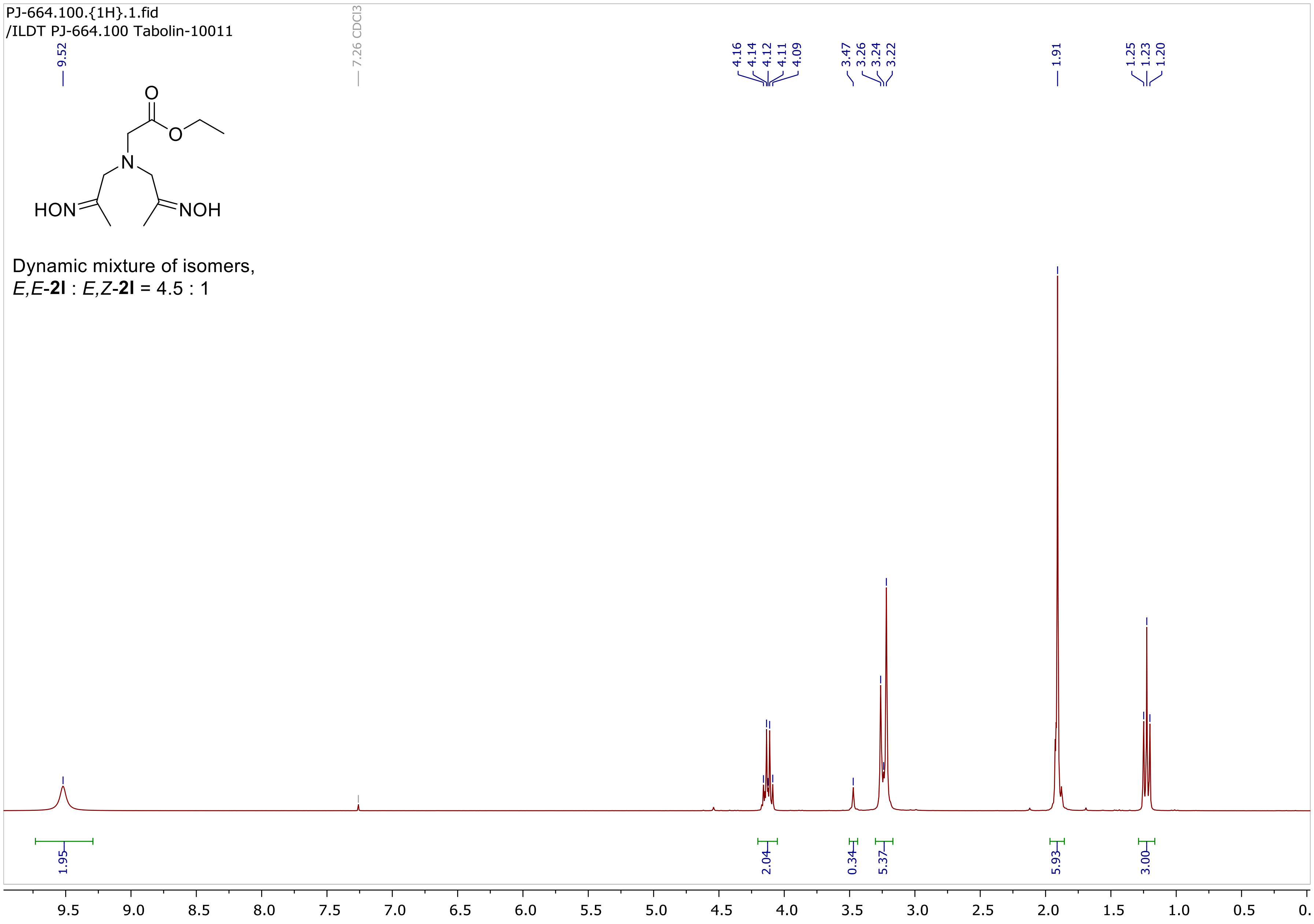
PJ-664.100.{1H}.1.fid
/ILDT PJ-664.100 Tabolin-10011
9.52
7.26 CDCl3
4.16
4.14
4.12
4.11
4.09
3.47
3.26
3.24
3.22
1.91
1.25
1.23
1.20
HON
NOH
N
O
O
Dynamic mixture of isomers,
E,E-**2l** : *E,Z*-**2l** = 4.5 : 1
1.95
2.04
0.34
5.37
5.93
3.00
9.5
9.0
8.5
8.0
7.5
7.0
6.5
6.0
5.5
5.0
4.5
4.0
3.5
3.0
2.5
2.0
1.5
1.0
0.5
0.

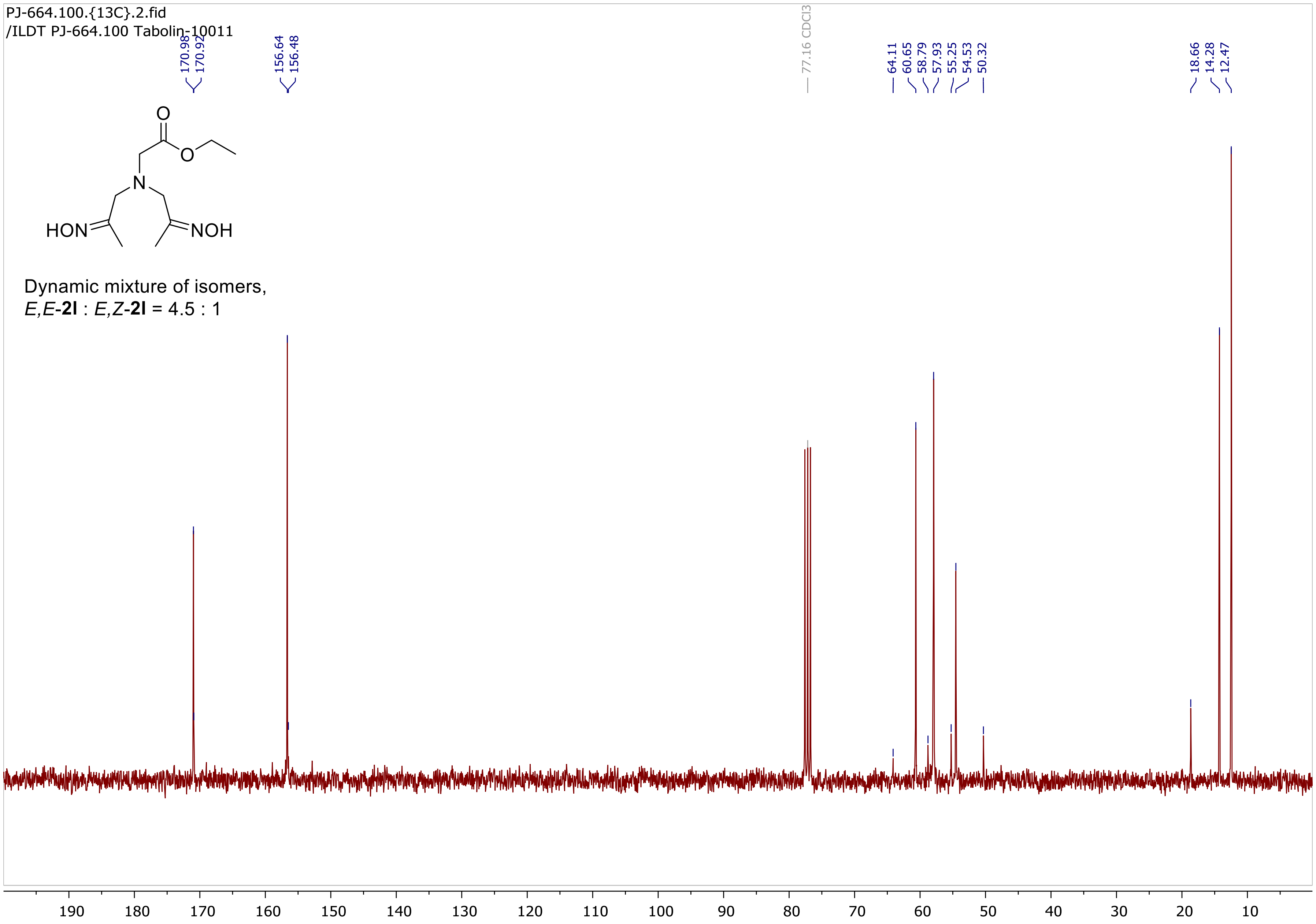
PJ-664.100.{13C}.2.fid
/ILDT PJ-664.100 Tabolin-10011
170.98
170.92
156.64
156.48
77.16 CDCl3
64.11
60.65
58.79
57.93
55.25
54.53
50.32
18.66
14.28
12.47
O
O
N
HON
NOH
Dynamic mixture of isomers,
E,E-2l : E,Z-2l = 4.5 : 1
190
180
170
160
150
140
130
120
110
100
90
80
70
60
50
40
30
20
10

PJ-664.100.{13C}deptsp135.3.fid
/ILDT PJ-664.100 Tabolin-10011

Dynamic mixture of isomers,
E,E-**2l** : *E,Z*-**2l** = 4.5 : 1

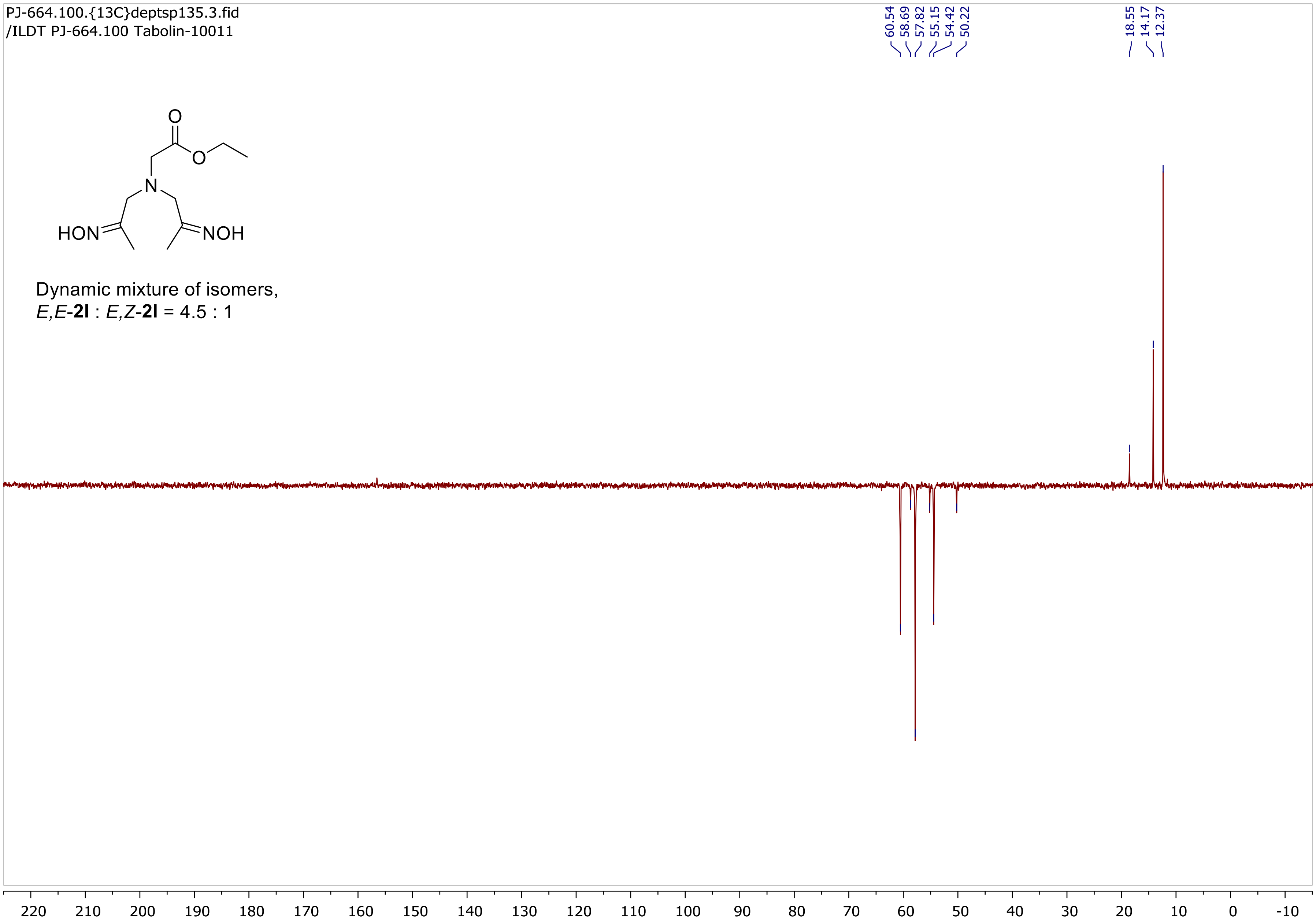

KVA-20.1.{1H}.1.fid
/ILDT KVA-20.1 Tabolin-10011

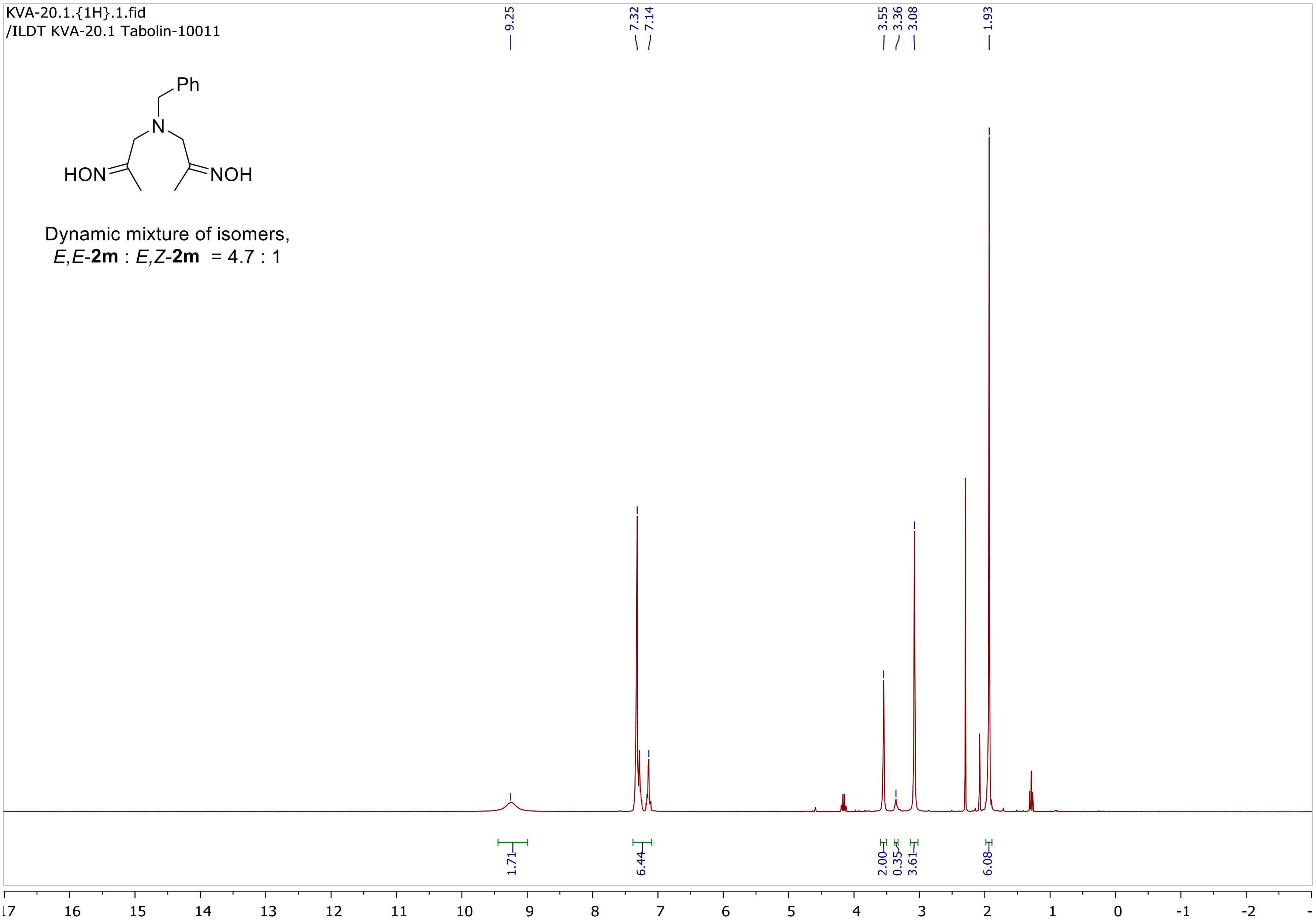

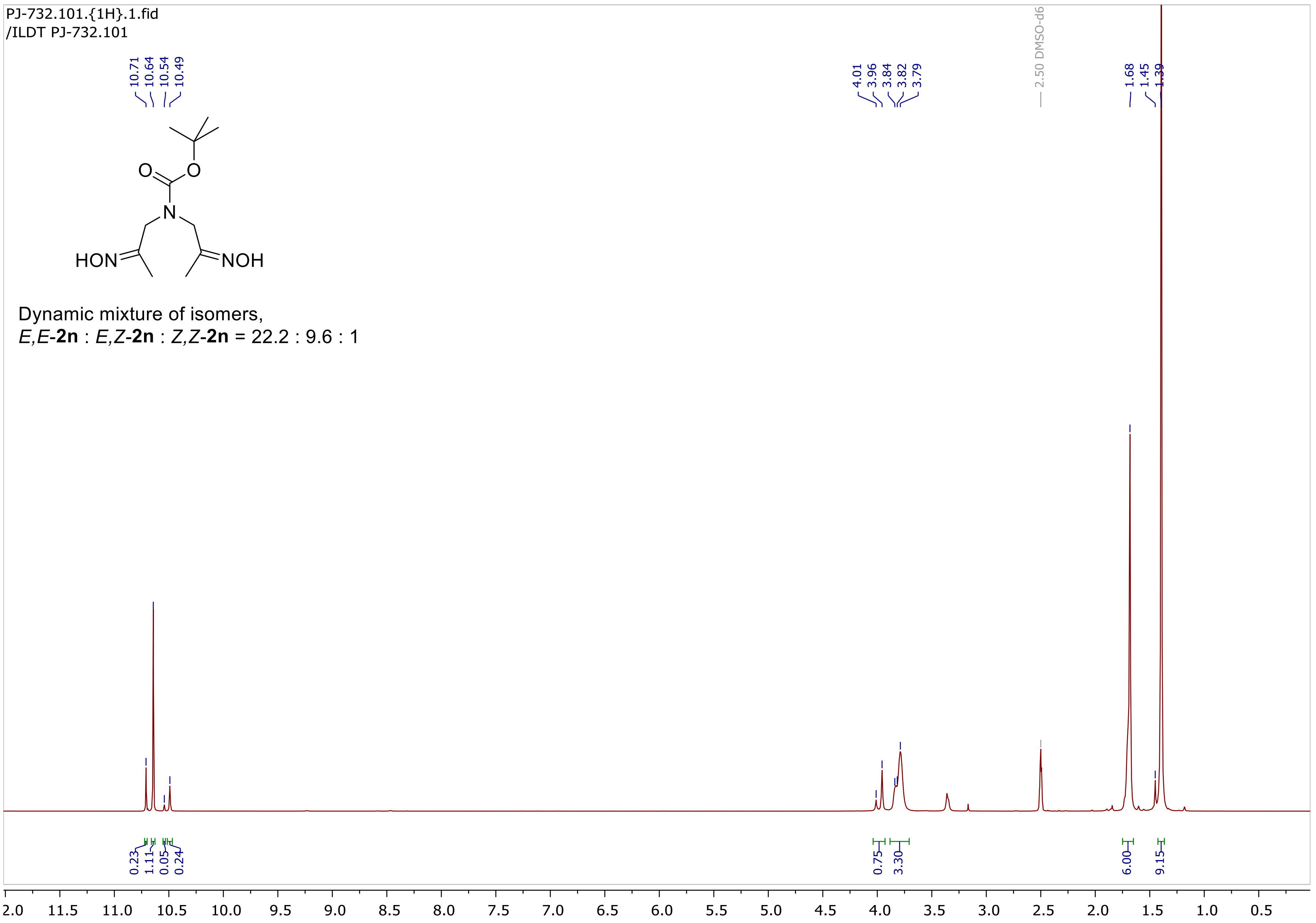
PJ-732.101.{1H}.1.fid
/ILDT PJ-732.101
10.71
10.64
10.54
10.49
4.01
3.96
3.84
3.82
3.79
2.50 DMSO-d6
1.68
1.45
1.39
HON
NOH
N
O
O
Dynamic mixture of isomers,
E,*E*-**2n** : *E*,*Z*-**2n** : *Z*,*Z*-**2n** = 22.2 : 9.6 : 1
0.23
1.11
0.05
0.24
0.75
3.30
6.00
9.15
2.0
11.5
11.0
10.5
10.0
9.5
9.0
8.5
8.0
7.5
7.0
6.5
6.0
5.5
5.0
4.5
4.0
3.5
3.0
2.5
2.0
1.5
1.0
0.5

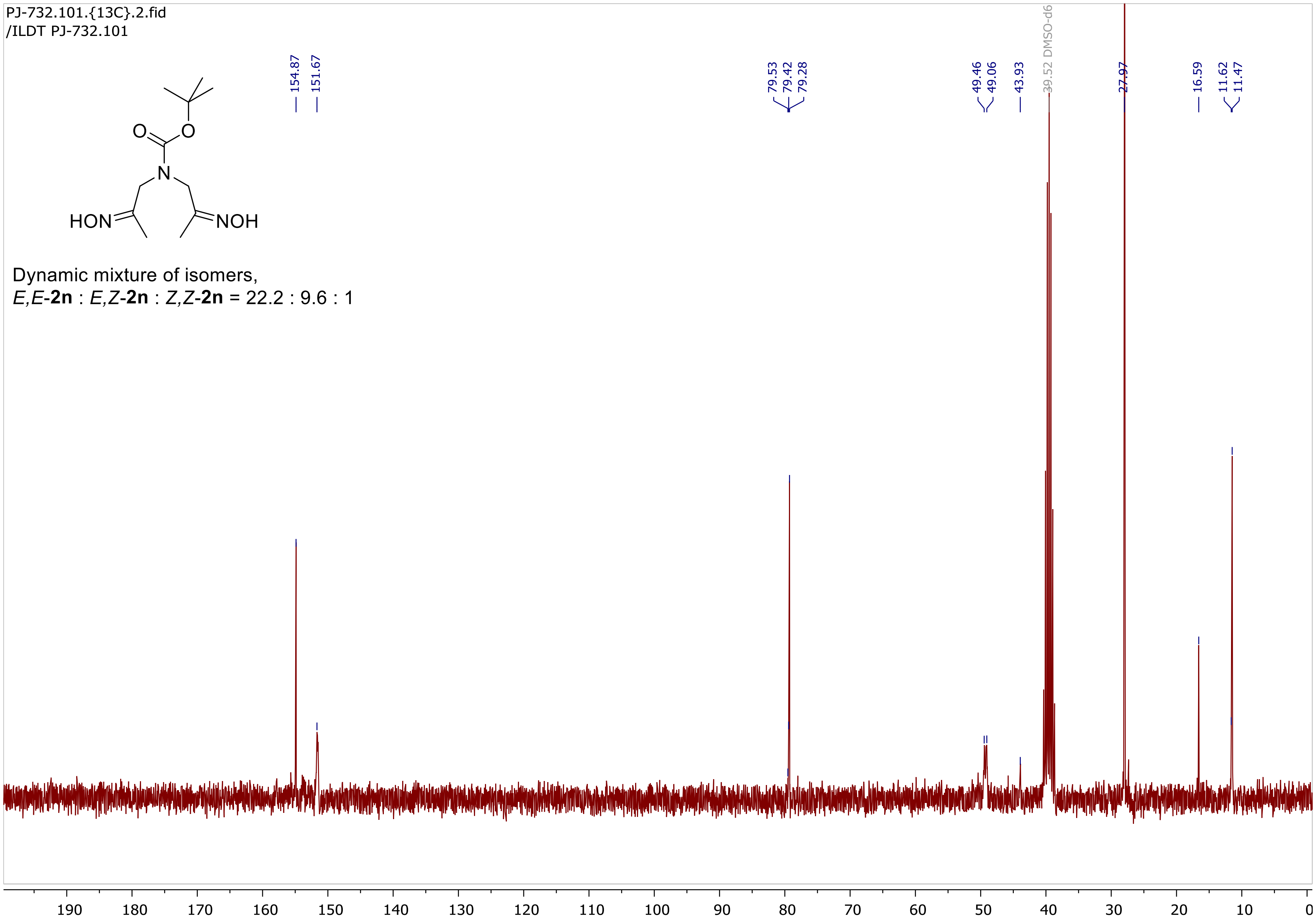
PJ-732.101.{13C}.2.fid
/ILDT PJ-732.101
HON
NOH
O
O
N
Dynamic mixture of isomers,
E,*E*-**2n** : *E*,*Z*-**2n** : *Z*,*Z*-**2n** = 22.2 : 9.6 : 1
154.87
151.67
79.53
79.42
79.28
49.46
49.06
43.93
39.52 DMSO-d6
27.97
16.59
11.62
11.47
190
180
170
160
150
140
130
120
110
100
90
80
70
60
50
40
30
20
10
0

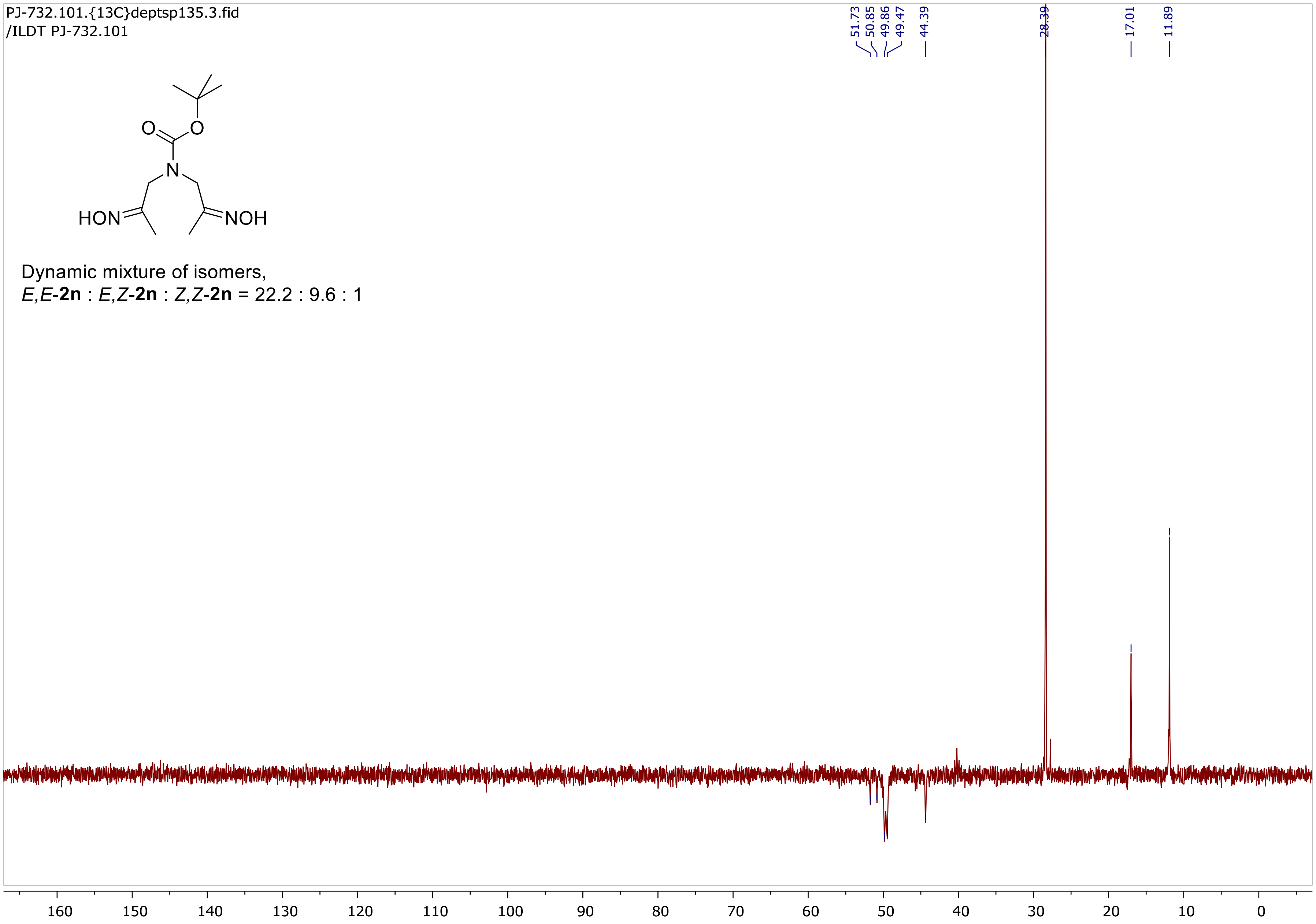
PJ-732.101.{13C}deptsp135.3.fid
/ILDT PJ-732.101
HON
NOH
O
O
N
Dynamic mixture of isomers,
E,*E*-**2n** : *E*,*Z*-**2n** : *Z*,*Z*-**2n** = 22.2 : 9.6 : 1
51.73
50.85
49.86
49.47
44.39
28.39
17.01
11.89
160
150
140
130
120
110
100
90
80
70
60
50
40
30
20
10
0

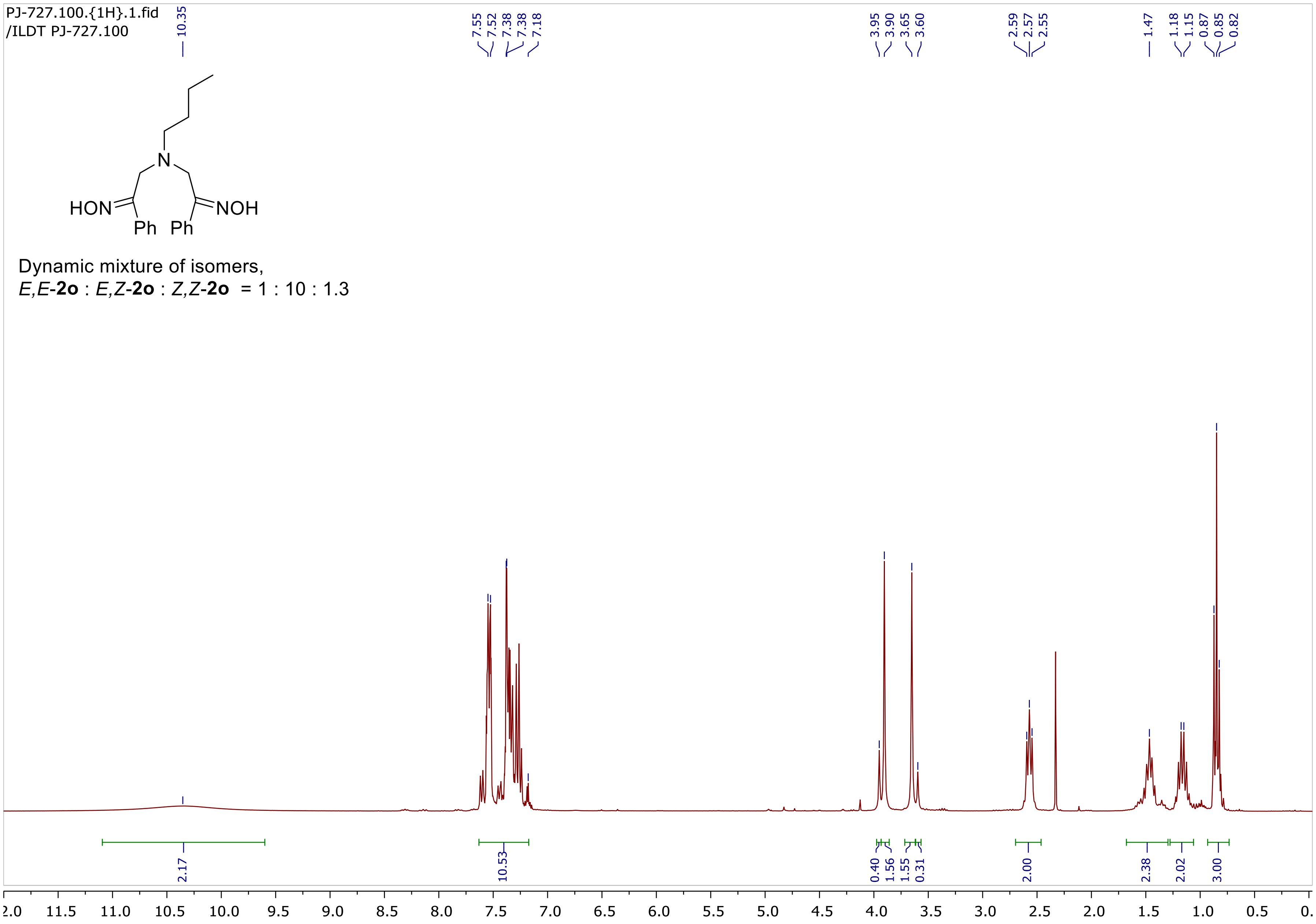
PJ-727.100.{1H}.1.fid
/ILDT PJ-727.100
HON
NOH
N
Ph Ph
Dynamic mixture of isomers,
E,E-**2o** : *E,Z*-**2o** : *Z,Z*-**2o** = 1 : 10 : 1.3
10.35
7.55
7.52
7.38
7.38
7.18
3.95
3.90
3.65
3.60
2.59
2.57
2.55
1.47
1.18
1.15
0.87
0.85
0.82
2.17
10.53
0.40
1.56
1.55
0.31
2.00
2.38
2.02
3.00

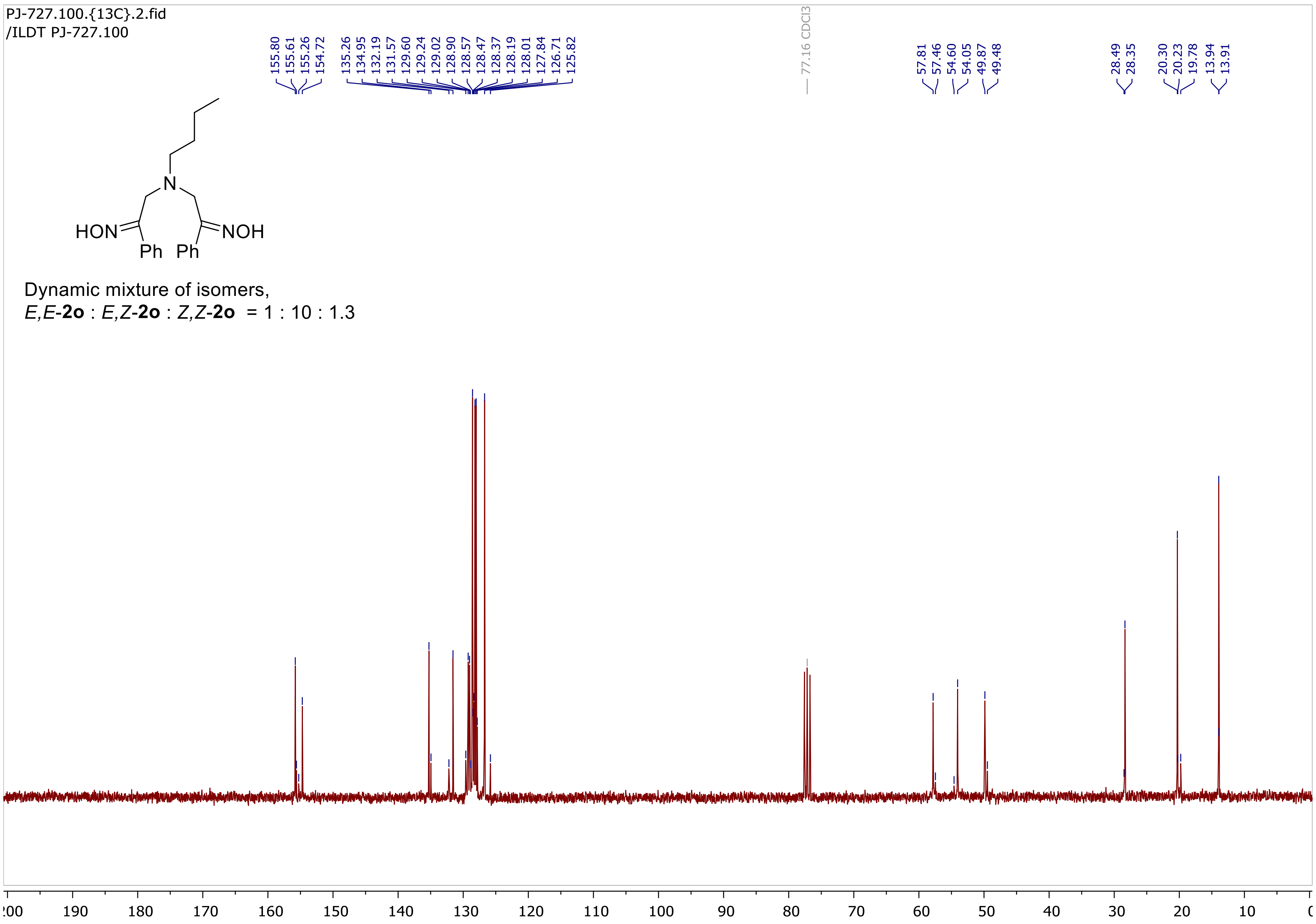
PJ-727.100.{13C}.2.fid
/ILDT PJ-727.100
155.80
155.61
155.26
154.72
135.26
134.95
132.19
131.57
129.60
129.24
129.02
128.90
128.57
128.47
128.37
128.19
128.01
127.84
126.71
125.82
77.16 CDCl3
57.81
57.46
54.60
54.05
49.87
49.48
28.49
28.35
20.30
20.23
19.78
13.94
13.91
N
HON
NOH
Ph
Ph
Dynamic mixture of isomers,
E,E-2o : E,Z-2o : Z,Z-2o = 1 : 10 : 1.3
190
180
170
160
150
140
130
120
110
100
90
80
70
60
50
40
30
20
10

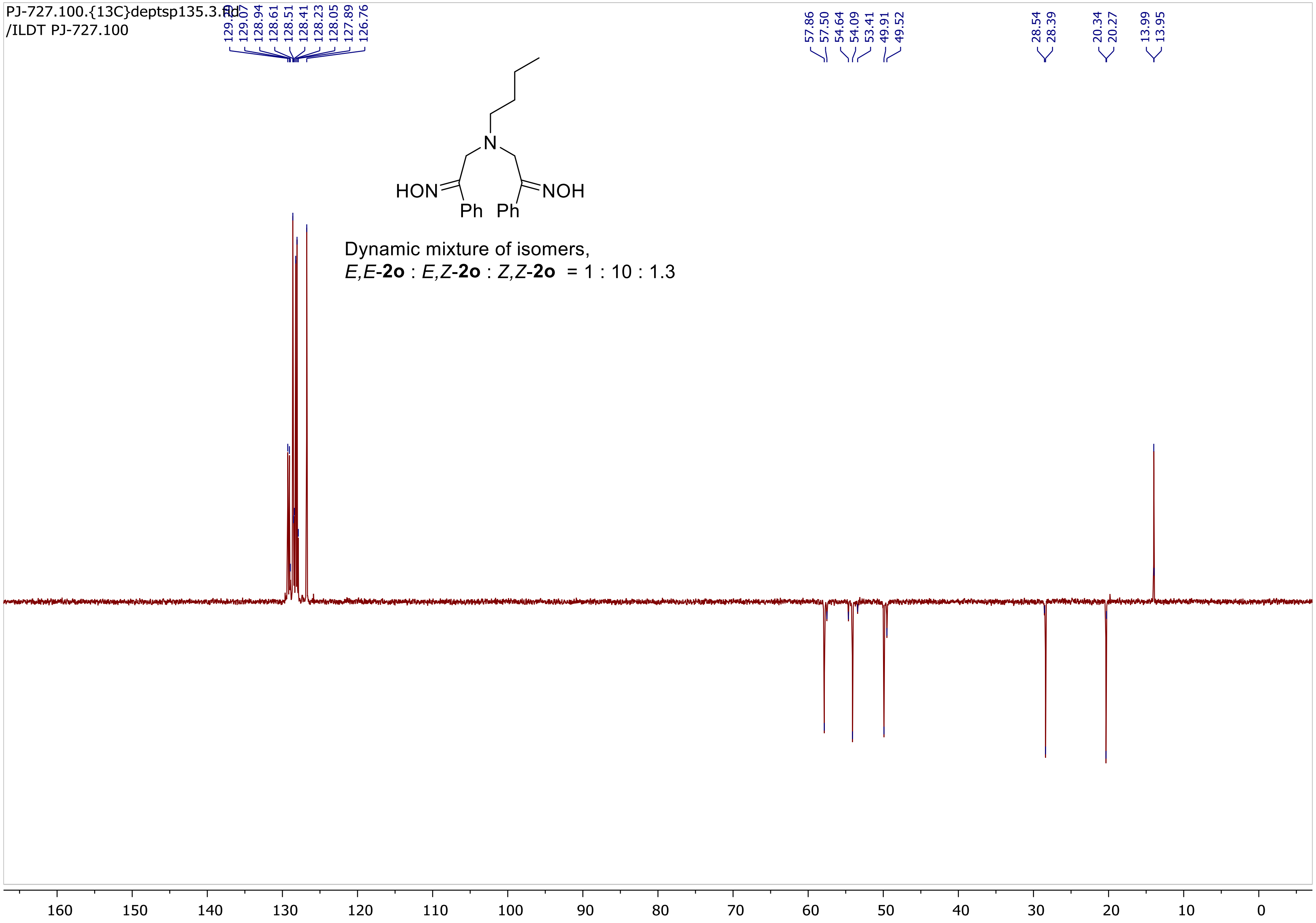
PJ-727.100.{13C}deptsp135.3.fid
/ILDT PJ-727.100
129.07
128.94
128.61
128.51
128.41
128.23
128.05
127.89
126.76
57.86
57.50
54.64
54.09
53.41
49.91
49.52
28.54
28.39
20.34
20.27
13.99
13.95
N
HON
NOH
Ph
Ph
Dynamic mixture of isomers,
E,E-**2o** : *E,Z*-**2o** : *Z,Z*-**2o** = 1 : 10 : 1.3
160
150
140
130
120
110
100
90
80
70
60
50
40
30
20
10
0

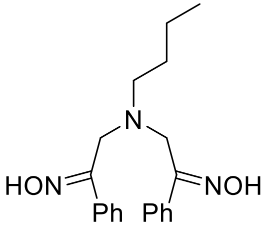

Dynamic mixture of isomers,
E,*E*-**2o** : *E*,*Z*-**2o** : *Z*,*Z*-**2o** =
1 : 10 : 1.3

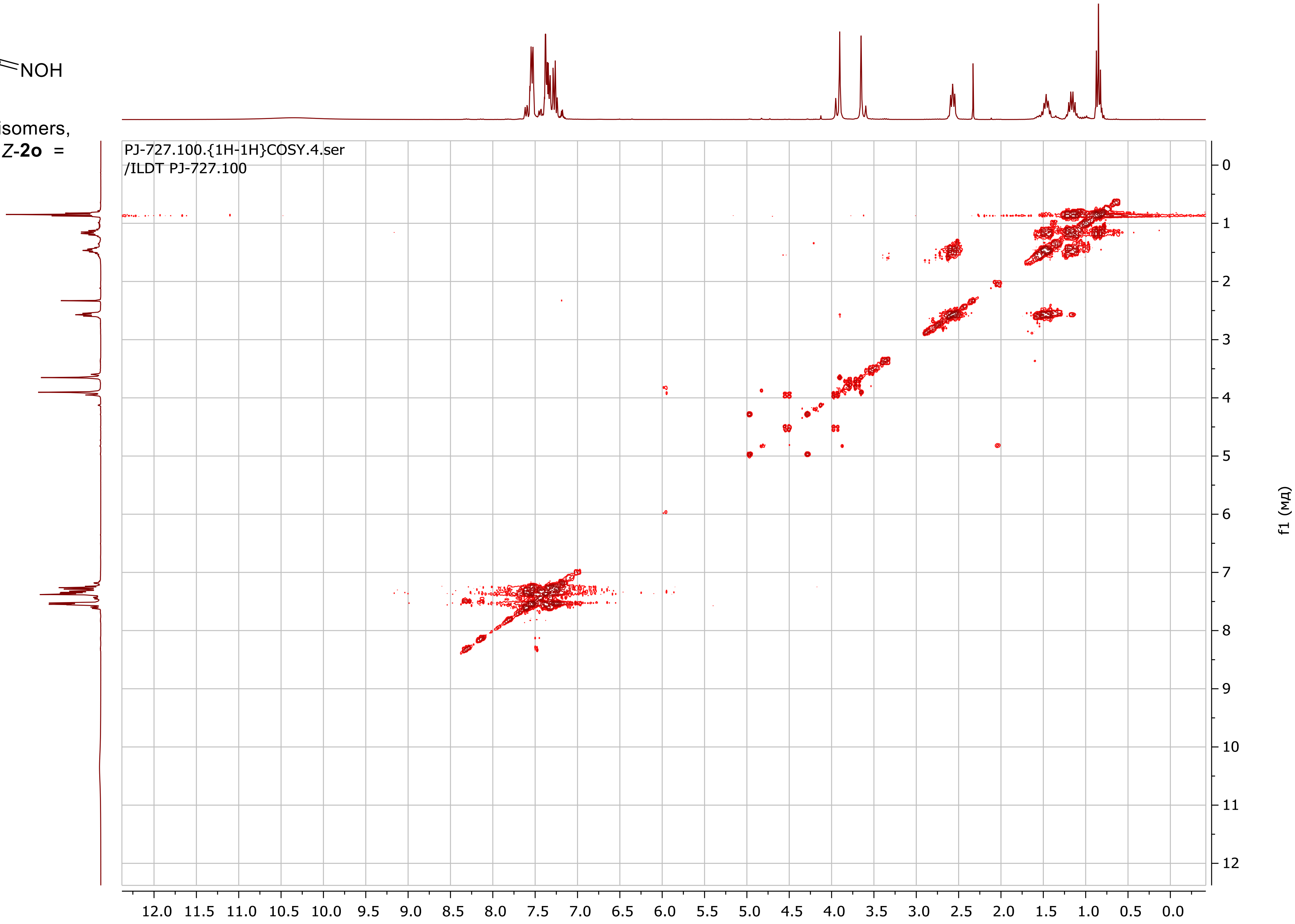

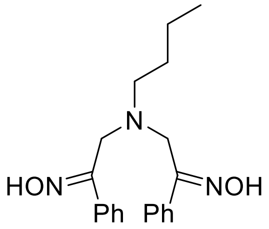

Dynamic mixture of isomers,
E,*E*-**2o** : *E*,*Z*-**2o** : *Z*,*Z*-**2o** = 1 : 10 : 1.3

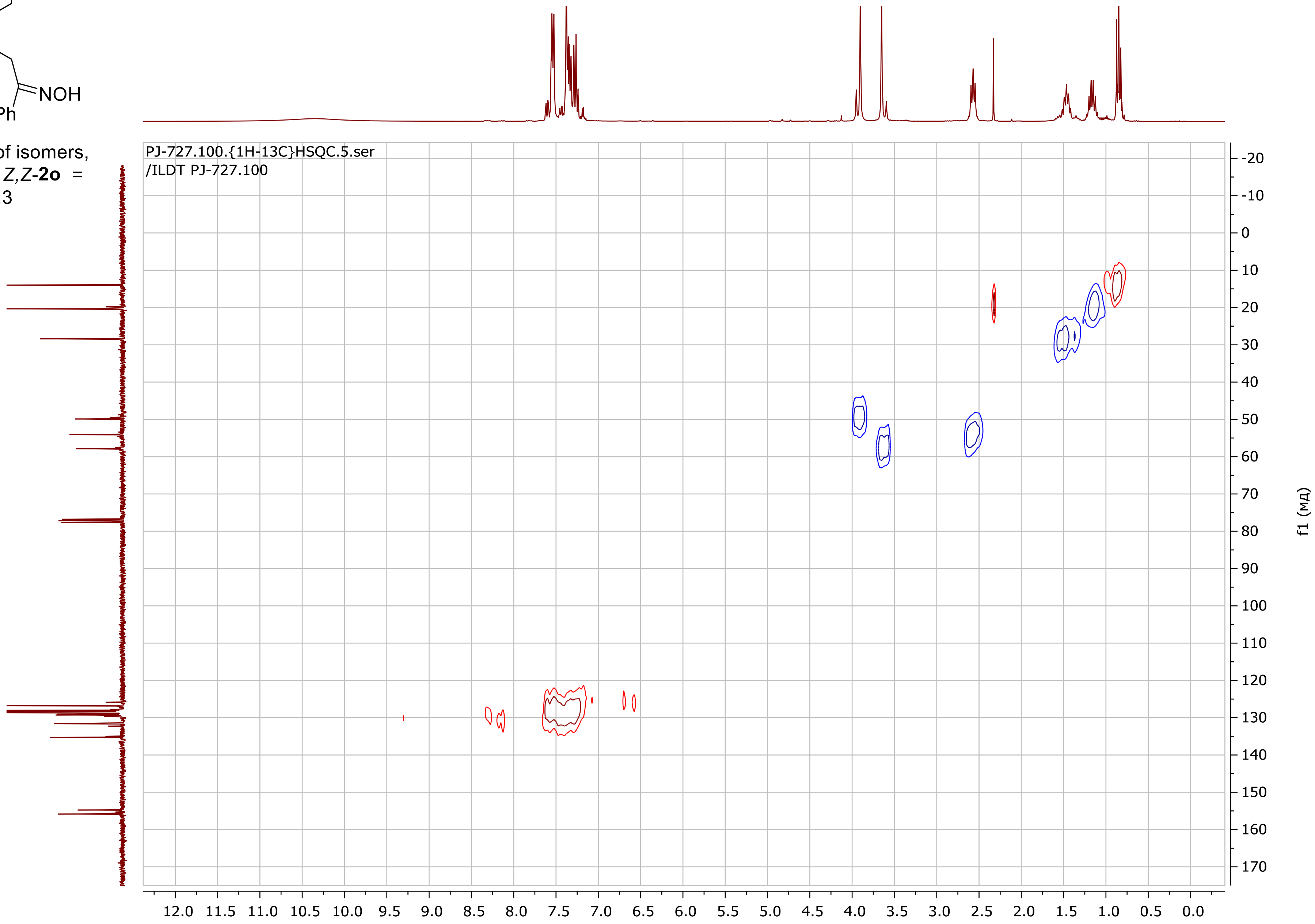

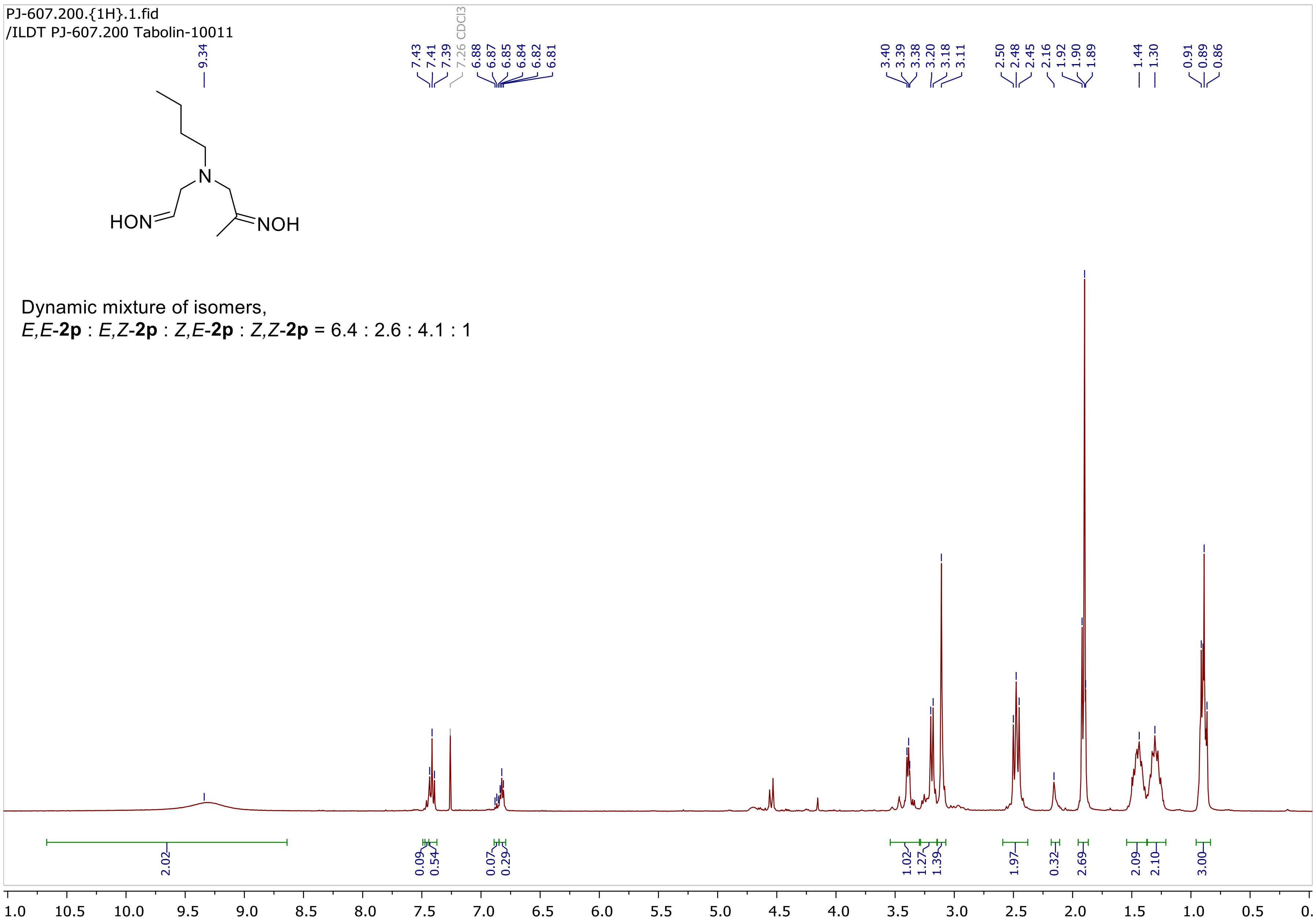
PJ-607.200.{1H}.1.fid
/ILDT PJ-607.200 Tabolin-10011
9.34
7.43
7.41
7.39
7.26 CDCl3
6.88
6.87
6.85
6.84
6.82
6.81
3.40
3.39
3.38
3.20
3.18
3.11
2.50
2.48
2.45
2.16
1.92
1.90
1.89
1.44
1.30
0.91
0.89
0.86
HON
N
NOH
Dynamic mixture of isomers,
E,E-**2p** : *E,Z*-**2p** : *Z,E*-**2p** : *Z,Z*-**2p** = 6.4 : 2.6 : 4.1 : 1
2.02
0.09
0.54
0.07
0.29
1.02
1.27
1.39
1.97
0.32
2.69
2.09
2.10
3.00
1.0
10.5
10.0
9.5
9.0
8.5
8.0
7.5
7.0
6.5
6.0
5.5
5.0
4.5
4.0
3.5
3.0
2.5
2.0
1.5
1.0
0.5
0.

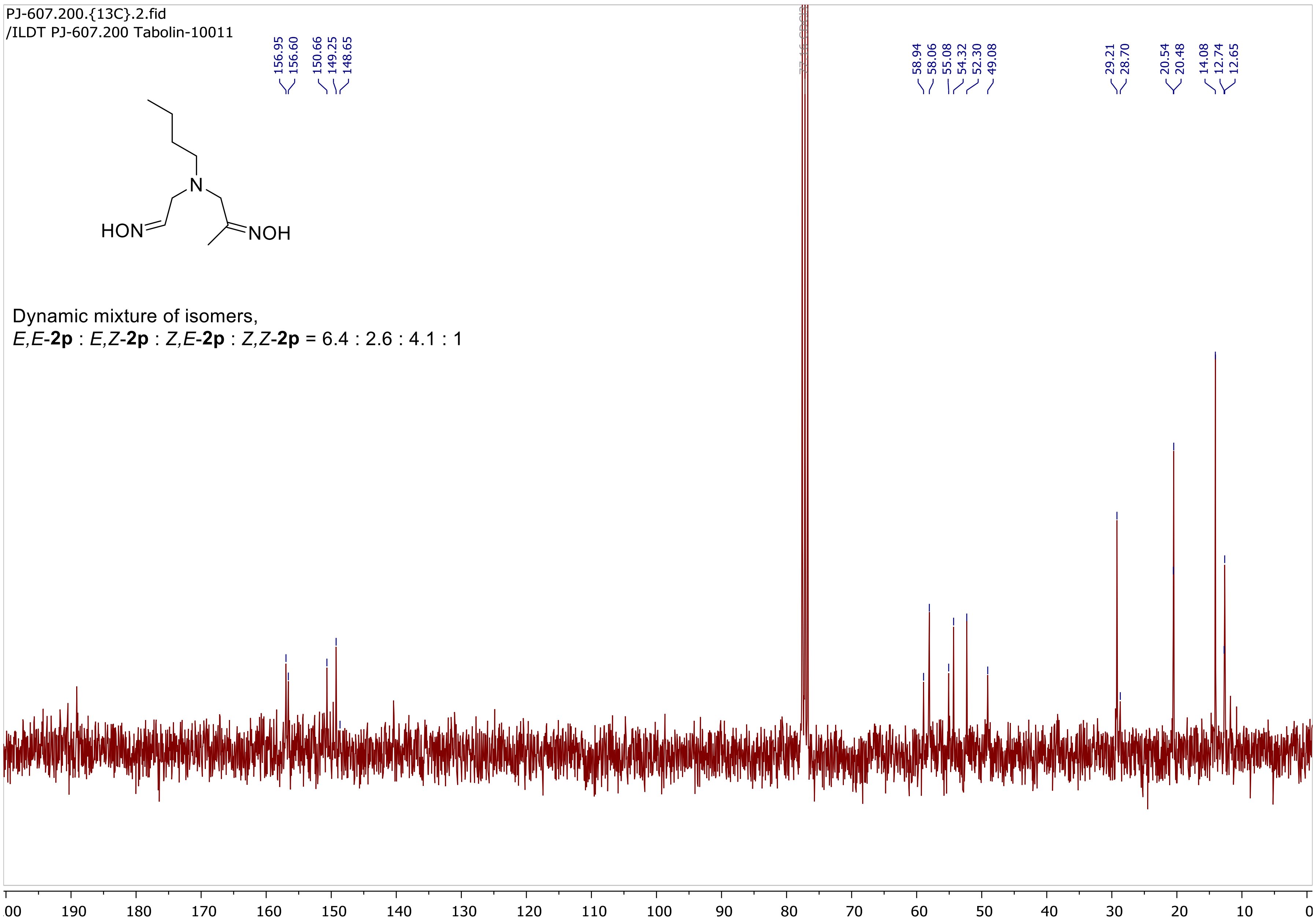
PJ-607.200.{13C}.2.fid
/ILDT PJ-607.200 Tabolin-10011
156.95
156.60
150.66
149.25
148.65
58.94
58.06
55.08
54.32
52.30
49.08
29.21
28.70
20.54
20.48
14.08
12.74
12.65
HON
N
NOH
Dynamic mixture of isomers,
E,E-**2p** : *E,Z*-**2p** : *Z,E*-**2p** : *Z,Z*-**2p** = 6.4 : 2.6 : 4.1 : 1
00
190
180
170
160
150
140
130
120
110
100
90
80
70
60
50
40
30
20
10
0

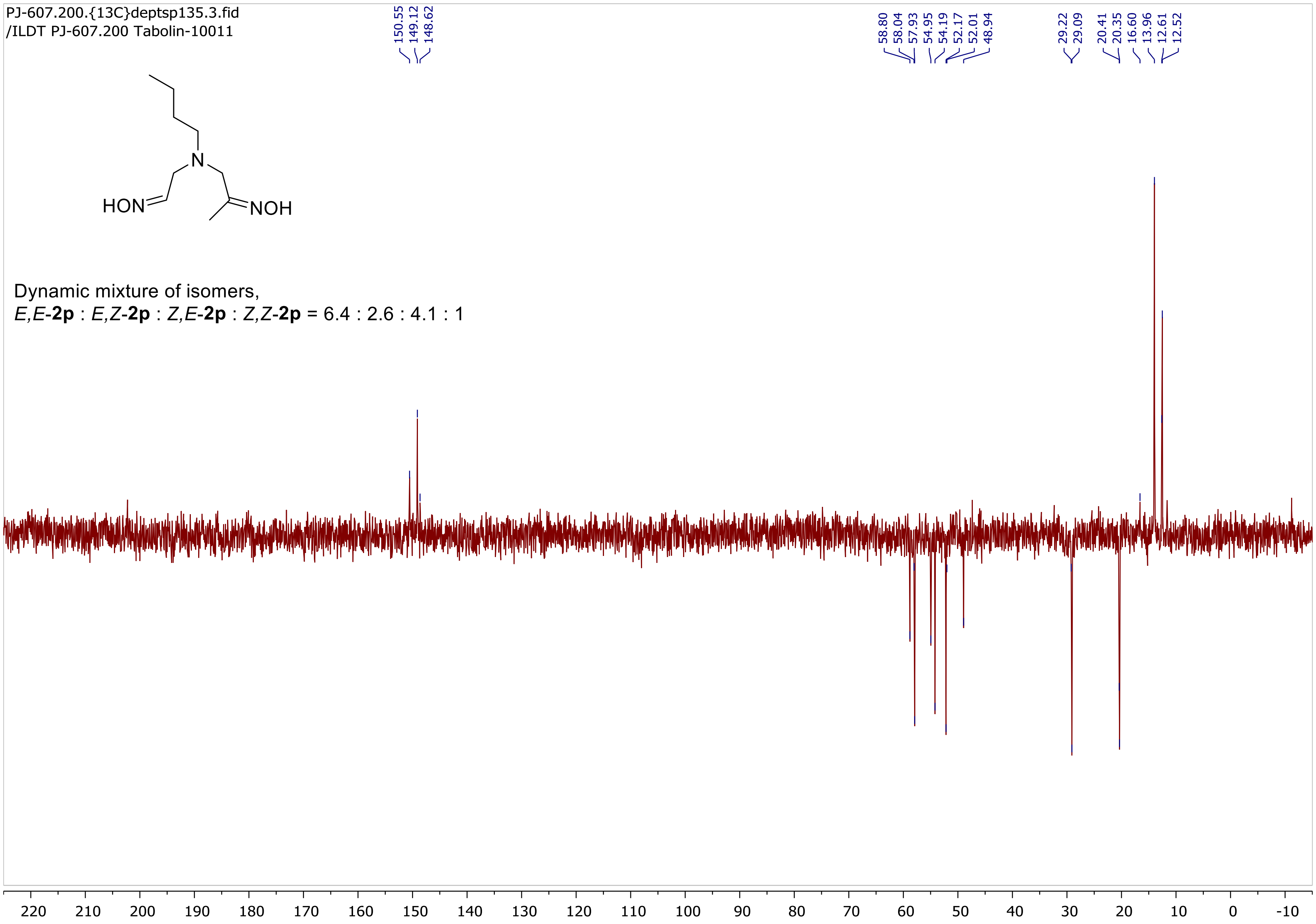
PJ-607.200.{13C}deptsp135.3.fid
/ILDT PJ-607.200 Tabolin-10011
150.55
149.12
148.62
58.80
58.04
57.93
54.95
54.19
52.17
52.01
48.94
29.22
29.09
20.41
20.35
16.60
13.96
12.61
12.52
HON
N
NOH
Dynamic mixture of isomers,
E,*E*-**2p** : *E*,*Z*-**2p** : *Z*,*E*-**2p** : *Z*,*Z*-**2p** = 6.4 : 2.6 : 4.1 : 1
220 210 200 190 180 170 160 150 140 130 120 110 100 90 80 70 60 50 40 30 20 10 0 -10

PJ-674.103.{1H}.1.fid
/ILDT PJ-674.103

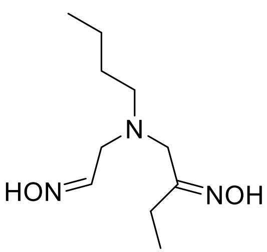

Dynamic mixture of isomers,
E,*E*-**2q** : *E*,*Z*-**2q** : *Z*,*E*-**2q** : *Z*,*Z*-**2q** = 1.6 : 1.5 : 1.3 : 1

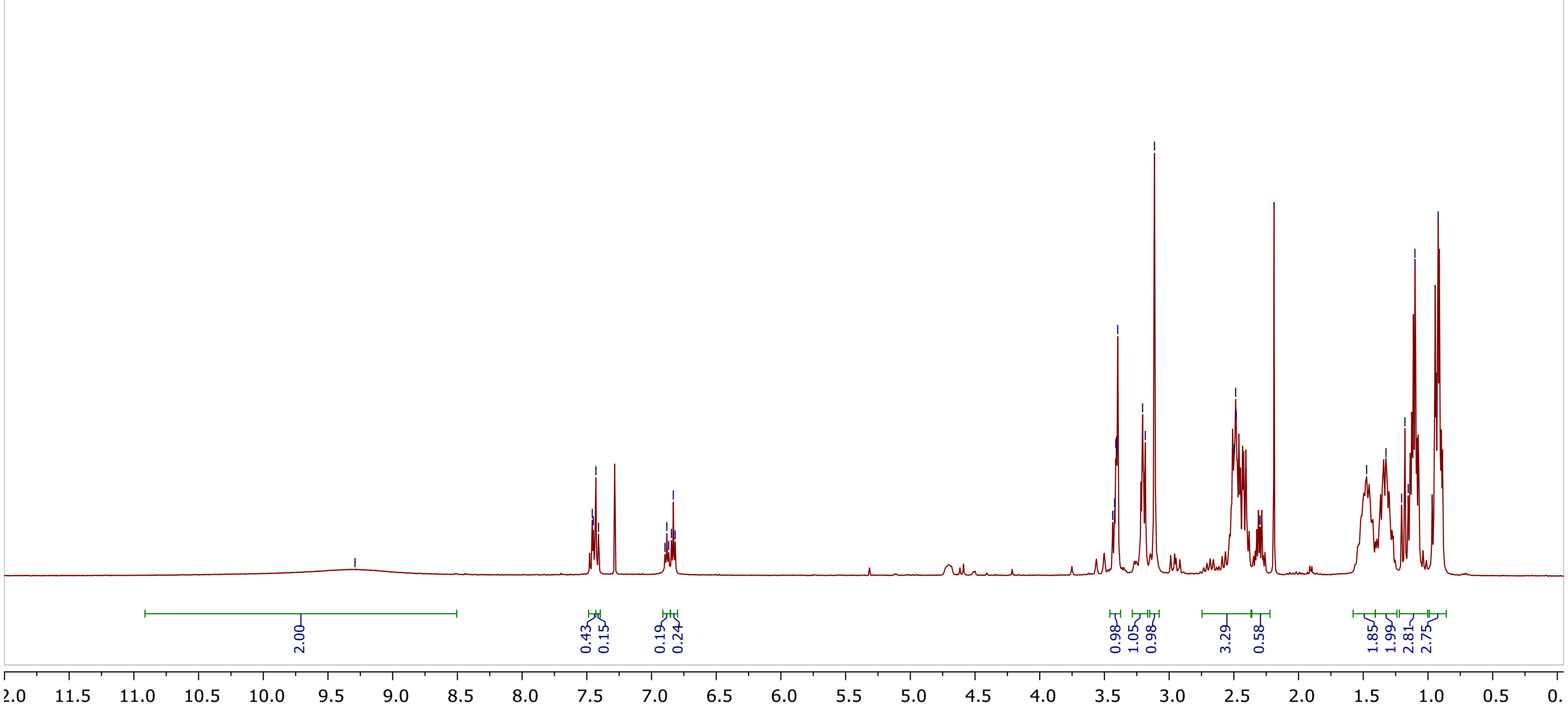

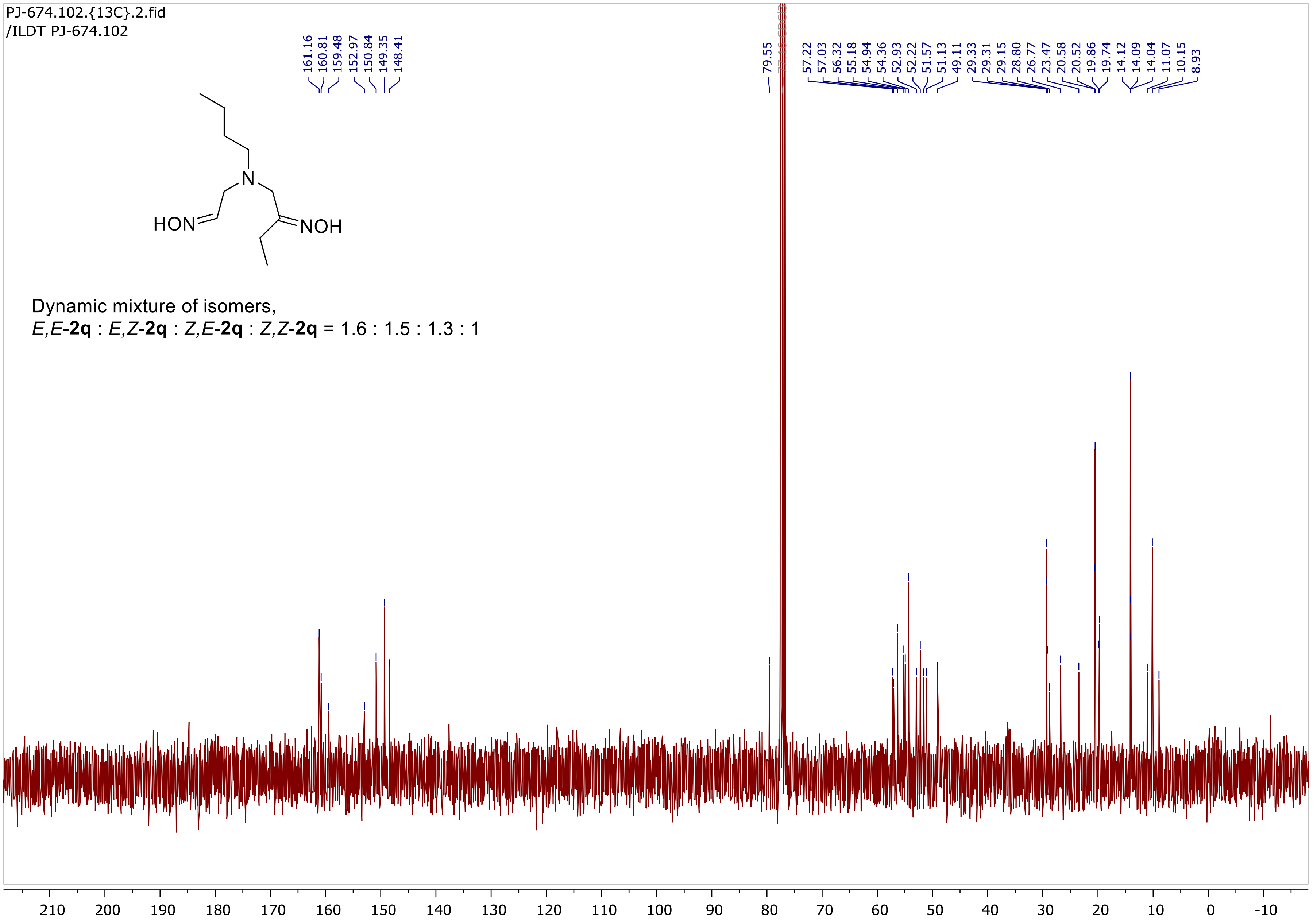

PJ-674.102.{13C}.2.fid
/ILDT PJ-674.102
161.16
160.81
159.48
152.97
150.84
149.35
148.41
79.55
57.22
57.03
56.32
55.18
54.94
54.36
52.93
52.22
51.57
51.13
49.11
29.33
29.31
29.15
28.80
26.77
23.47
20.58
20.52
19.86
19.74
14.12
14.09
14.04
11.07
10.15
8.93
N
HON
NOH
Dynamic mixture of isomers,
E,*E*-**2q** : *E*,*Z*-**2q** : *Z*,*E*-**2q** : *Z*,*Z*-**2q** = 1.6 : 1.5 : 1.3 : 1
210 200 190 180 170 160 150 140 130 120 110 100 90 80 70 60 50 40 30 20 10 0 -10

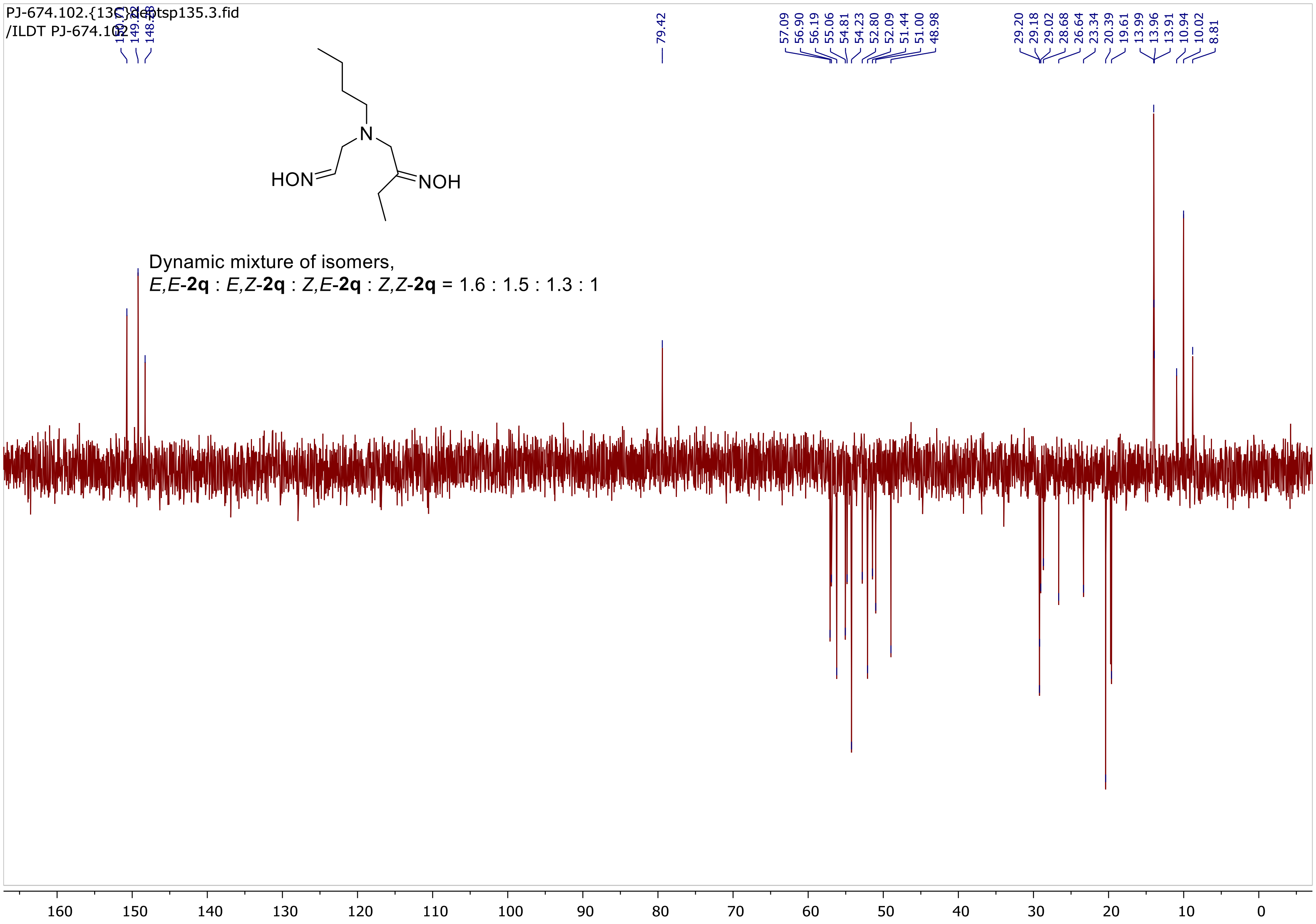
PJ-674.102.{13C}deptsp135.3.fid
/ILDT PJ-674.102
150.71
149.22
148.28
79.42
57.09
56.90
56.19
55.06
54.81
54.23
52.80
52.09
51.44
51.00
48.98
29.20
29.18
29.02
28.68
26.64
23.34
20.39
19.61
13.99
13.96
13.91
10.94
10.02
8.81
HON
N
NOH
Dynamic mixture of isomers,
E,*E*-**2q** : *E*,*Z*-**2q** : *Z*,*E*-**2q** : *Z*,*Z*-**2q** = 1.6 : 1.5 : 1.3 : 1
160
150
140
130
120
110
100
90
80
70
60
50
40
30
20
10
0

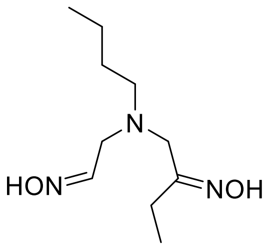

Dynamic mixture of isomers,
E,*E*-**2q** : *E*,*Z*-**2q** : *Z*,*E*-**2q** : *Z*,*Z*-**2q** =
1.6 : 1.5 : 1.3 : 1

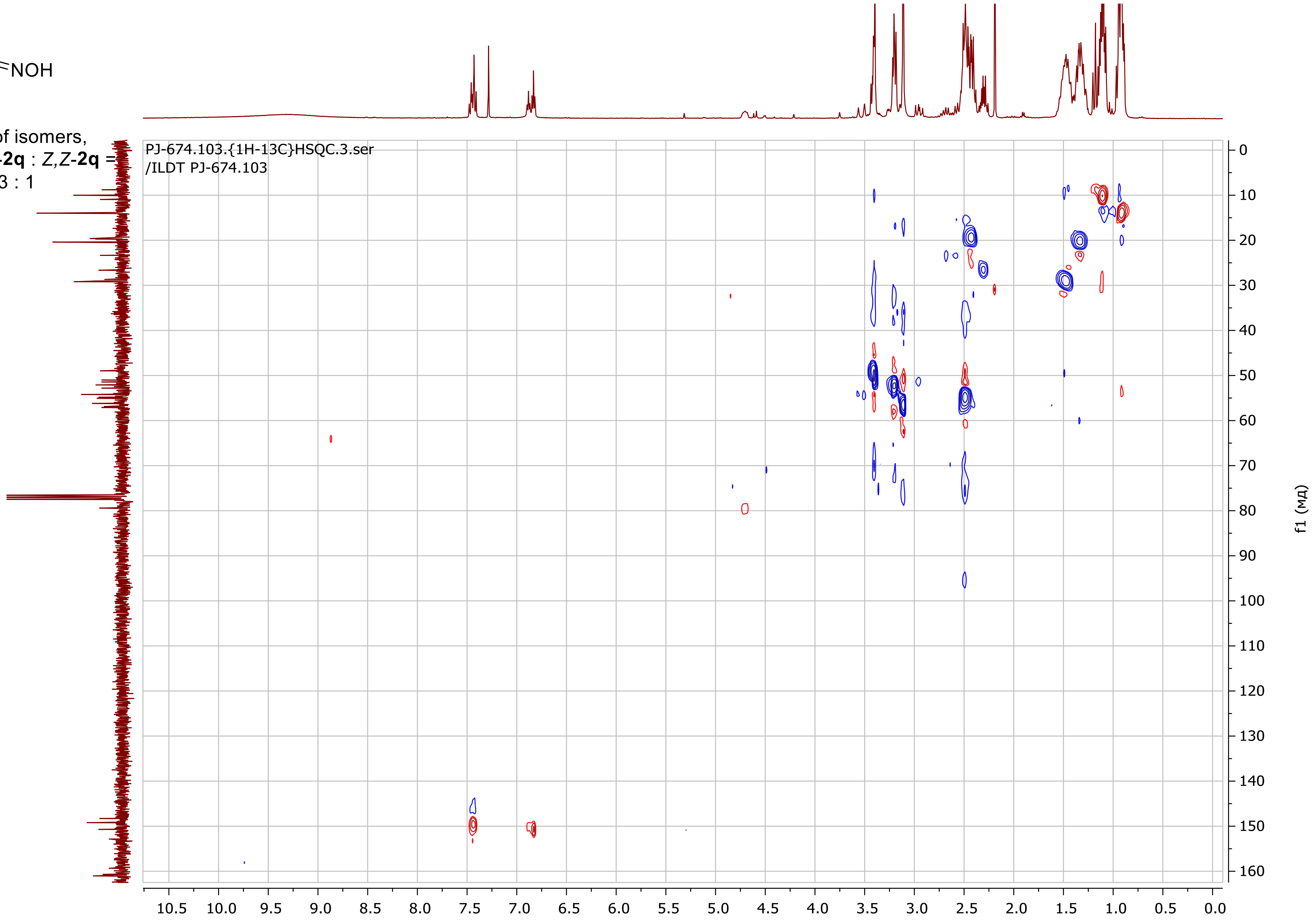

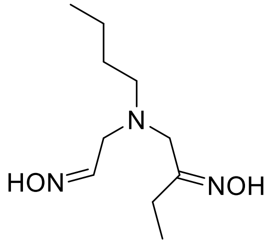

Dynamic mixture of isomers,
E,*E*-**2q** : *E*,*Z*-**2q** : *Z*,*E*-**2q** : *Z*,*Z*-**2q** =
1.6 : 1.5 : 1.3 : 1

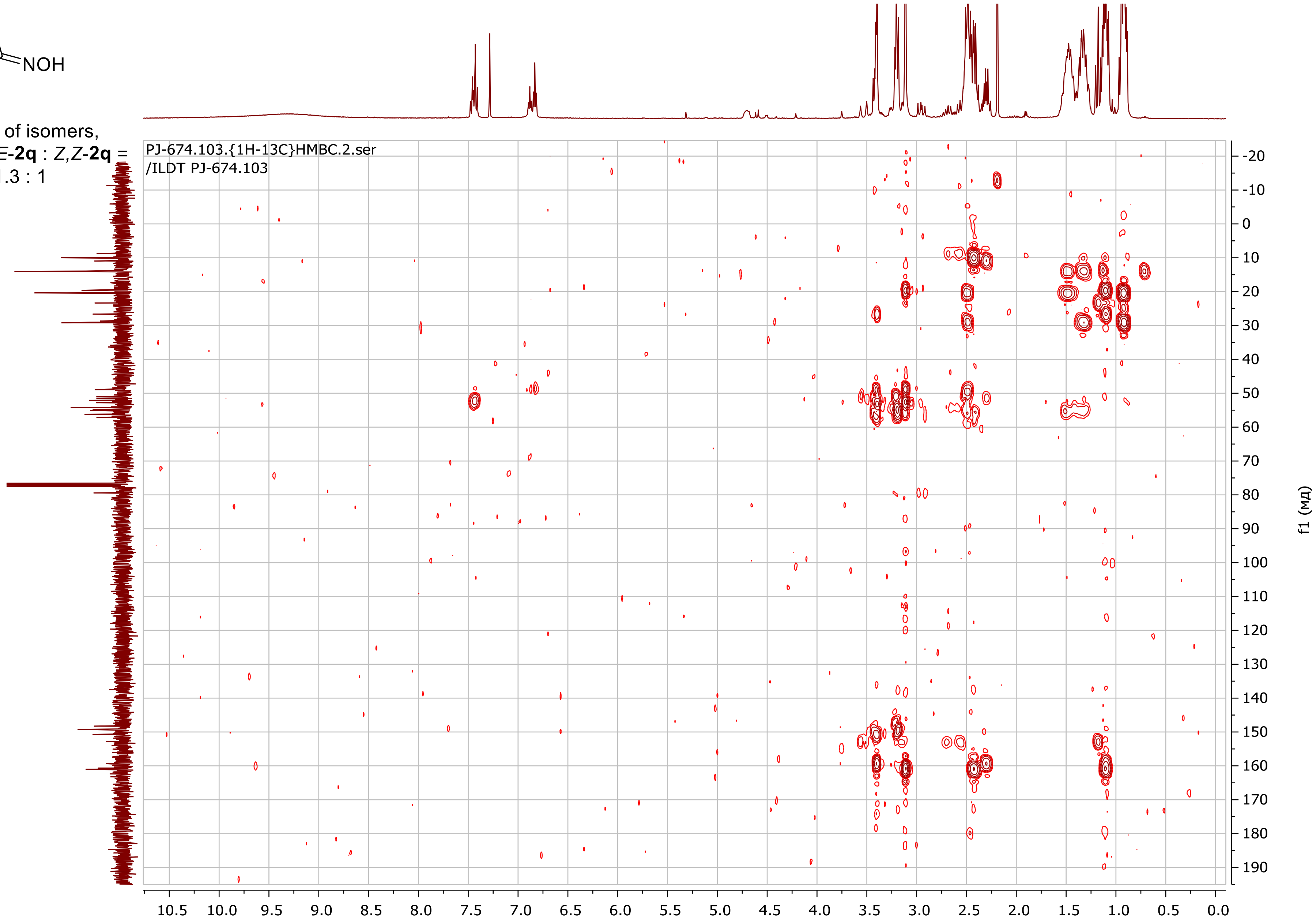

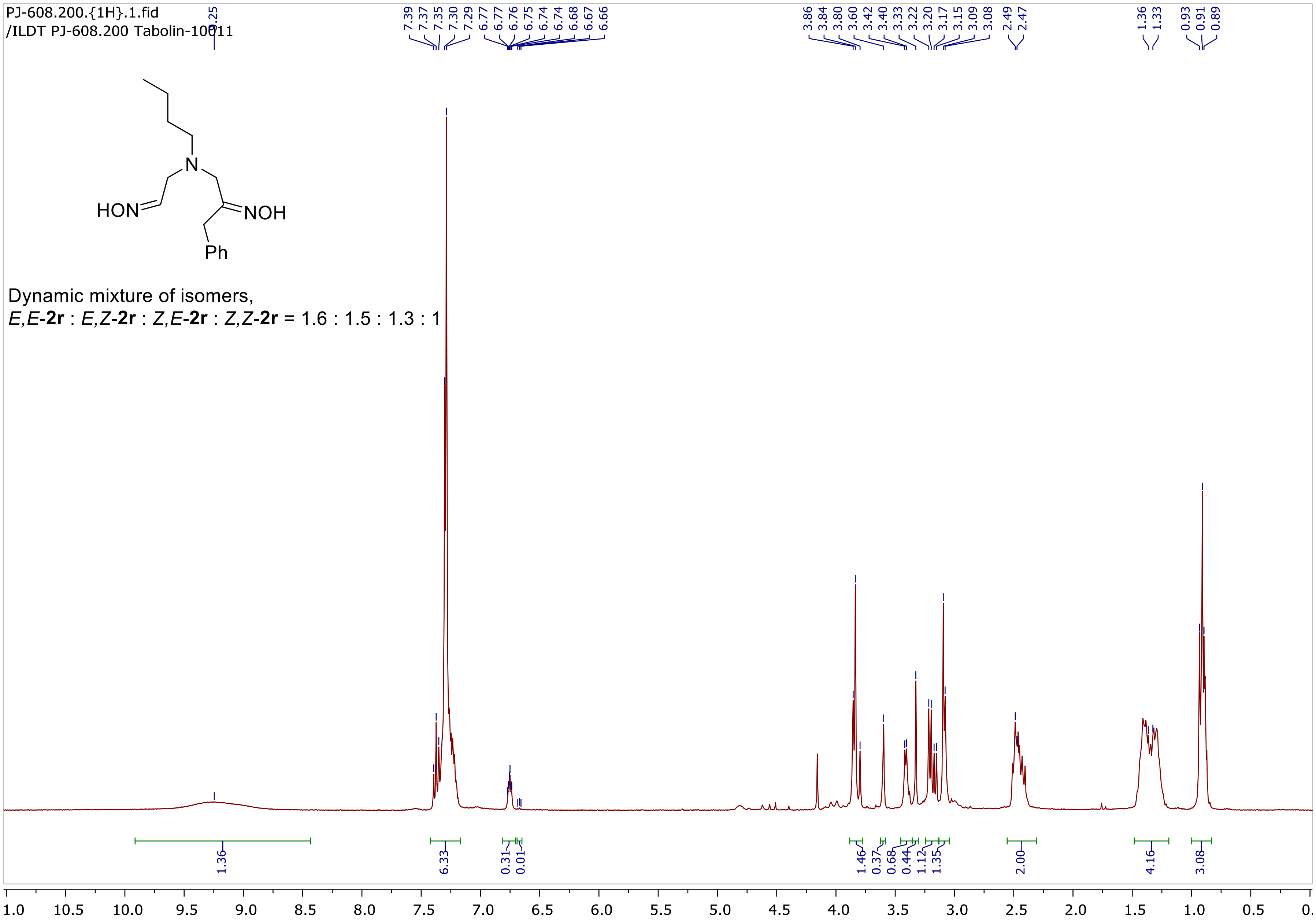
PJ-608.200.{1H}.1.fid
/ILDT PJ-608.200 Tabolin-10011
HON
N
NOH
Ph
Dynamic mixture of isomers,
E,E-**2r** : *E,Z*-**2r** : *Z,E*-**2r** : *Z,Z*-**2r** = 1.6 : 1.5 : 1.3 : 1
9.25
7.39 7.37 7.35 7.30 7.29 6.77 6.77 6.76 6.75 6.74 6.74 6.68 6.67 6.66
3.86 3.84 3.80 3.60 3.42 3.40 3.33 3.22 3.20 3.17 3.15 3.09 3.08 2.49 2.47
1.36 1.33 0.93 0.91 0.89
1.36 6.33 0.31 0.01 1.46 0.37 0.68 0.44 1.12 1.35 2.00 4.16 3.08

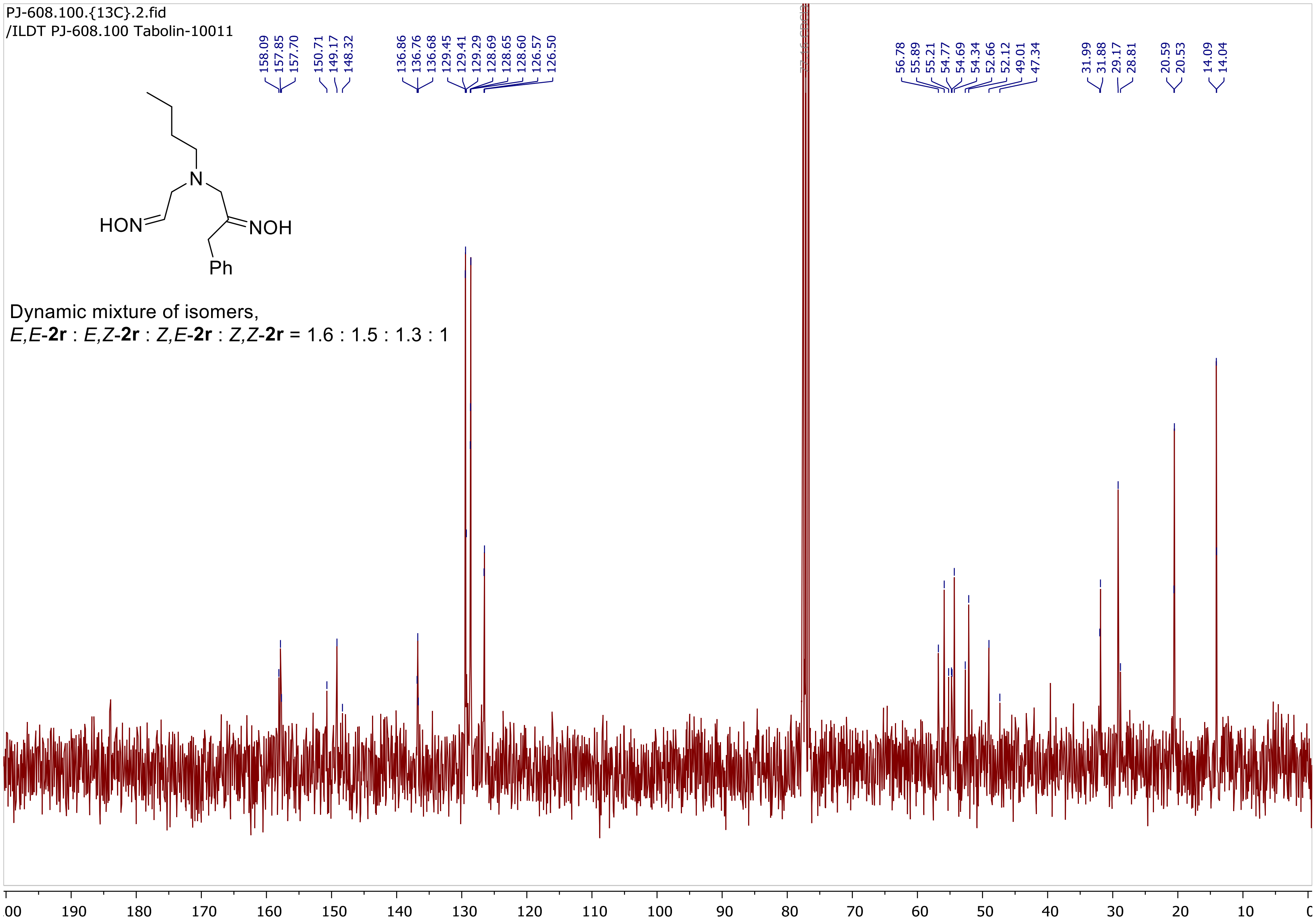
PJ-608.100.{13C}.2.fid
/ILDT PJ-608.100 Tabolin-10011
158.09
157.85
157.70
150.71
149.17
148.32
136.86
136.76
136.68
129.45
129.41
129.29
128.69
128.65
128.60
126.57
126.50
56.78
55.89
55.21
54.77
54.69
54.34
52.66
52.12
49.01
47.34
31.99
31.88
29.17
28.81
20.59
20.53
14.09
14.04
N
HON
NOH
Ph
Dynamic mixture of isomers,
E,E-**2r** : *E,Z*-**2r** : *Z,E*-**2r** : *Z,Z*-**2r** = 1.6 : 1.5 : 1.3 : 1
190
180
170
160
150
140
130
120
110
100
90
80
70
60
50
40
30
20
10

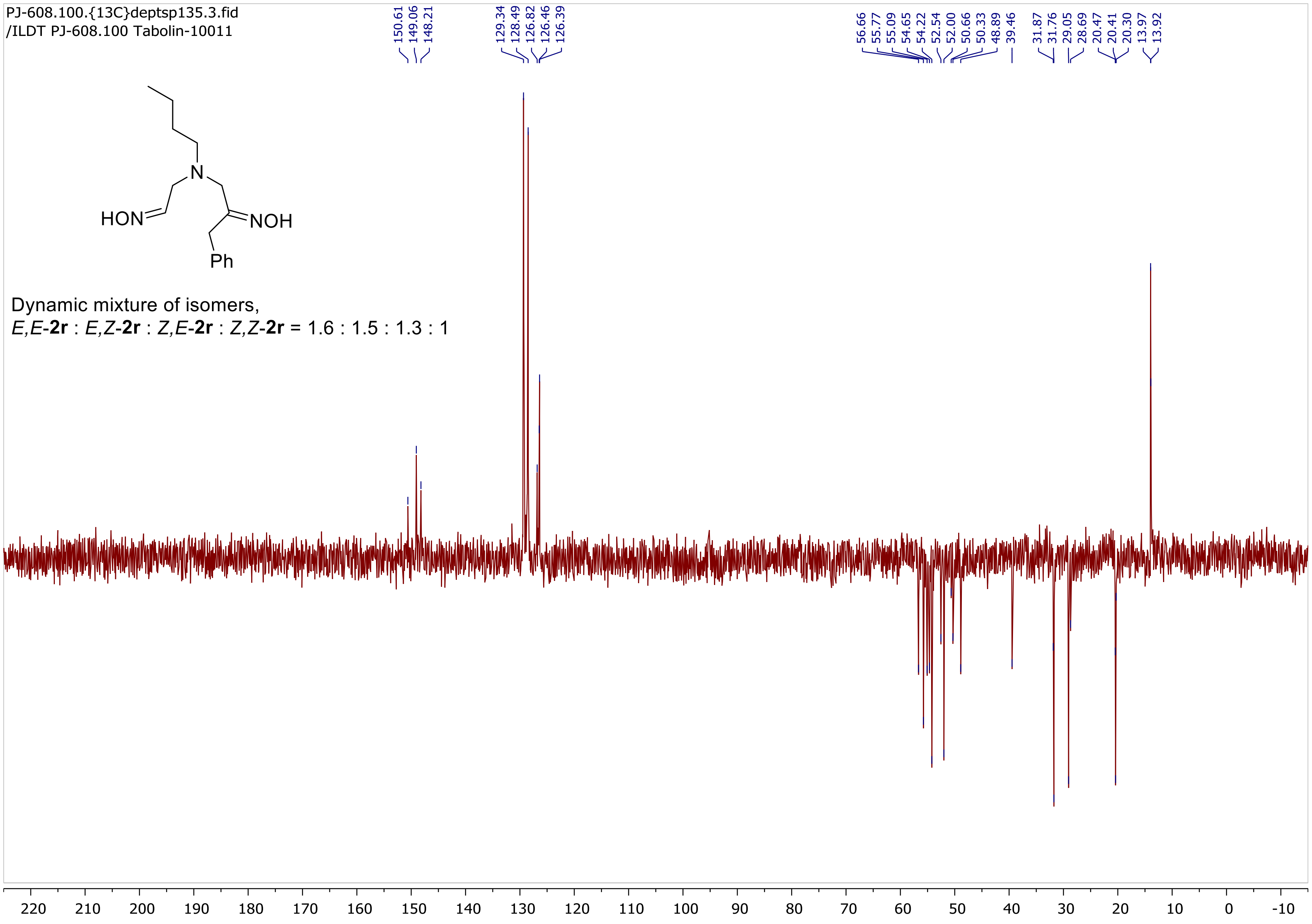
PJ-608.100.{13C}deptsp135.3.fid
/ILDT PJ-608.100 Tabolin-10011
150.61
149.06
148.21
129.34
128.49
126.82
126.46
126.39
56.66
55.77
55.09
54.65
54.22
52.54
52.00
50.66
50.33
48.89
39.46
31.87
31.76
29.05
28.69
20.47
20.41
20.30
13.97
13.92
HON
N
NOH
Ph
Dynamic mixture of isomers,
E,*E*-**2r** : *E*,*Z*-**2r** : *Z*,*E*-**2r** : *Z*,*Z*-**2r** = 1.6 : 1.5 : 1.3 : 1
220 210 200 190 180 170 160 150 140 130 120 110 100 90 80 70 60 50 40 30 20 10 0 -10

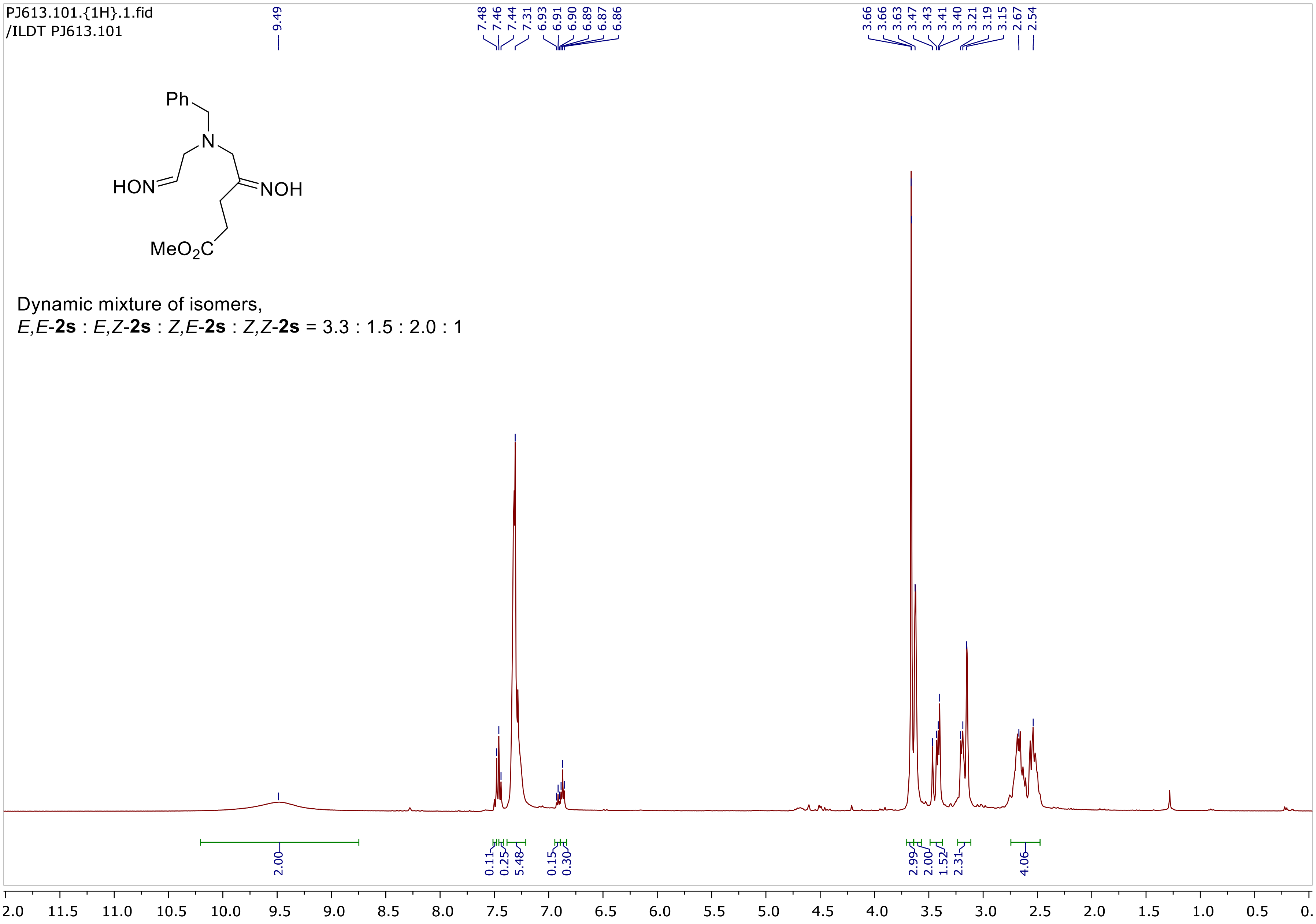
PJ613.101.{1H}.1.fid
/ILDT PJ613.101
Ph
N
HON
NOH
MeO2C
Dynamic mixture of isomers,
E,E-2s : E,Z-2s : Z,E-2s : Z,Z-2s = 3.3 : 1.5 : 2.0 : 1
9.49
7.48
7.46
7.44
7.31
6.93
6.91
6.90
6.89
6.87
6.86
3.66
3.66
3.63
3.47
3.43
3.41
3.40
3.21
3.19
3.15
2.67
2.54
2.00
0.11
0.25
5.48
0.15
0.30
2.99
2.00
1.52
2.31
4.06
2.0
11.5
11.0
10.5
10.0
9.5
9.0
8.5
8.0
7.5
7.0
6.5
6.0
5.5
5.0
4.5
4.0
3.5
3.0
2.5
2.0
1.5
1.0
0.5
0.

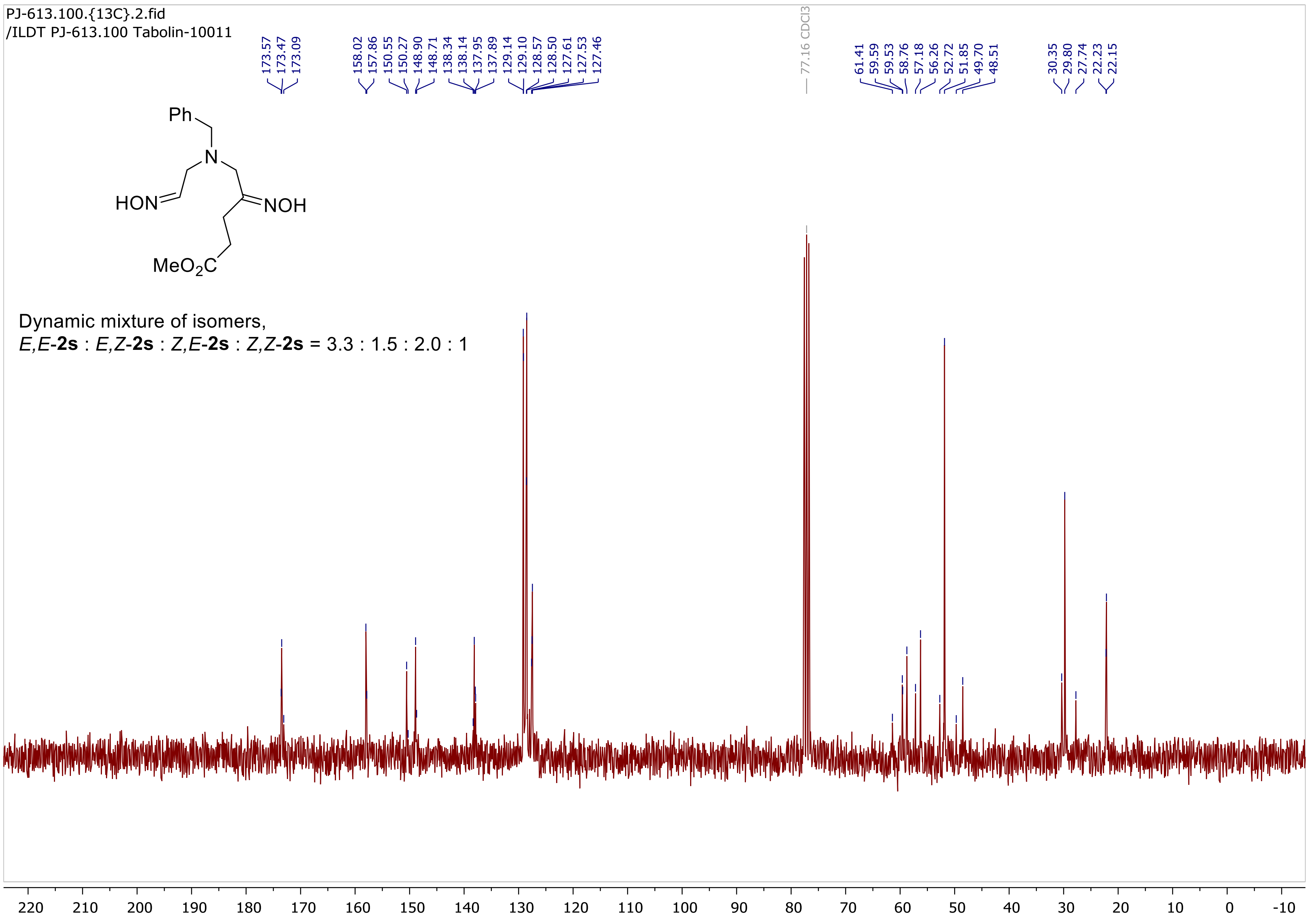
PJ-613.100.{13C}.2.fid
/ILDT PJ-613.100 Tabolin-10011
173.57
173.47
173.09
158.02
157.86
150.55
150.27
148.90
148.71
138.34
138.14
137.95
137.89
129.14
129.10
128.57
128.50
127.61
127.53
127.46
77.16 CDCl3
61.41
59.59
59.53
58.76
57.18
56.26
52.72
51.85
49.70
48.51
30.35
29.80
27.74
22.23
22.15
Ph
N
HON
NOH
MeO2C
Dynamic mixture of isomers,
E,E-2s : E,Z-2s : Z,E-2s : Z,Z-2s = 3.3 : 1.5 : 2.0 : 1
220 210 200 190 180 170 160 150 140 130 120 110 100 90 80 70 60 50 40 30 20 10 0 -10

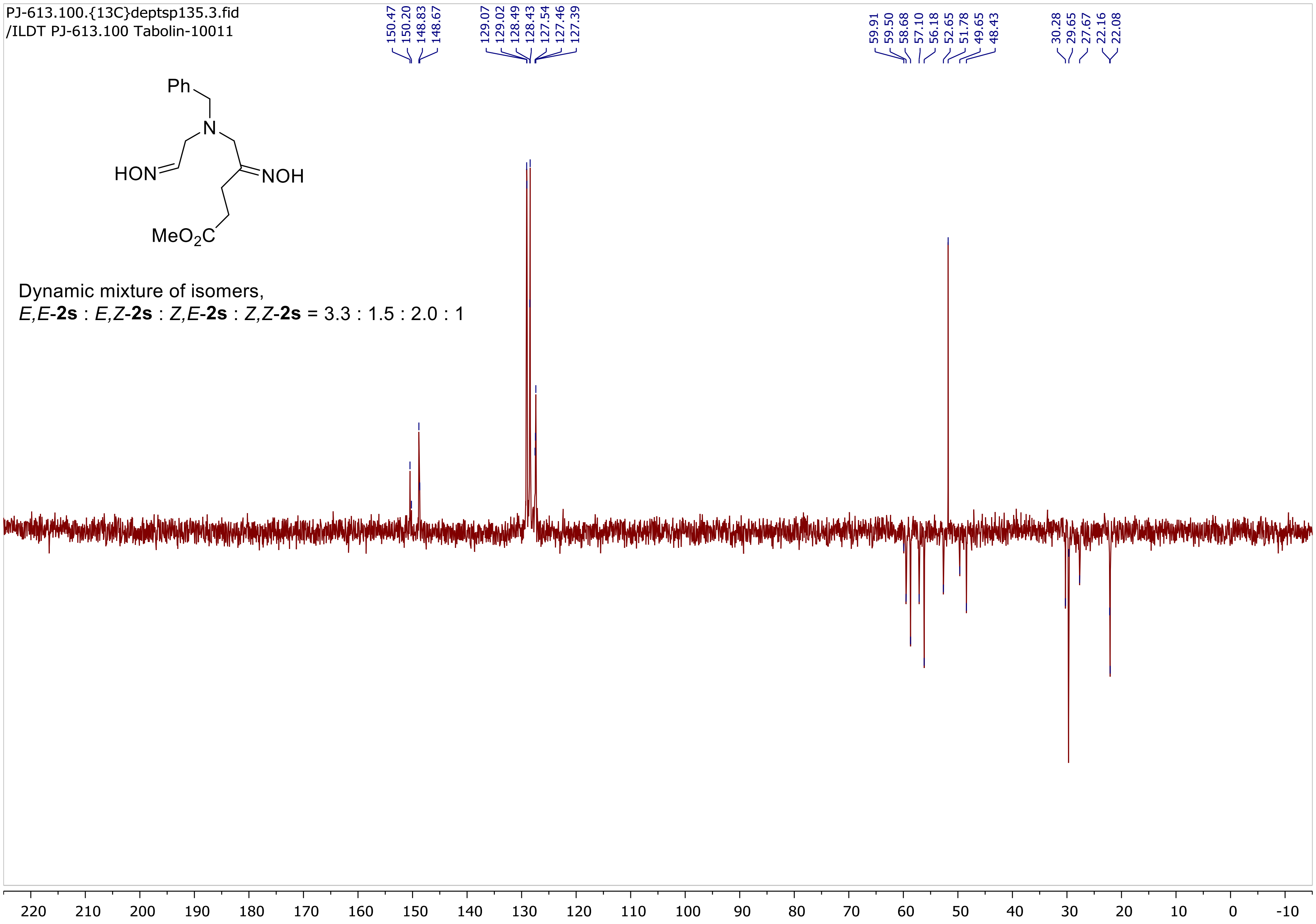

Dynamic mixture of isomers,
E,*E*-**2s** : *E*,*Z*-**2s** : *Z*,*E*-**2s** : *Z*,*Z*-**2s** = 3.3 : 1.5 : 2.0 : 1

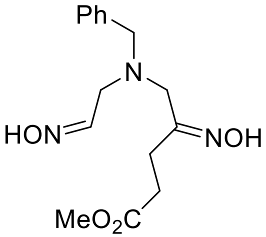

Dynamic mixture of isomers,
E,*E*-**2s** : *E*,*Z*-**2s** : *Z*,*E*-**2s** : *Z*,*Z*-**2s** =
3.3 : 1.5 : 2.0 : 1

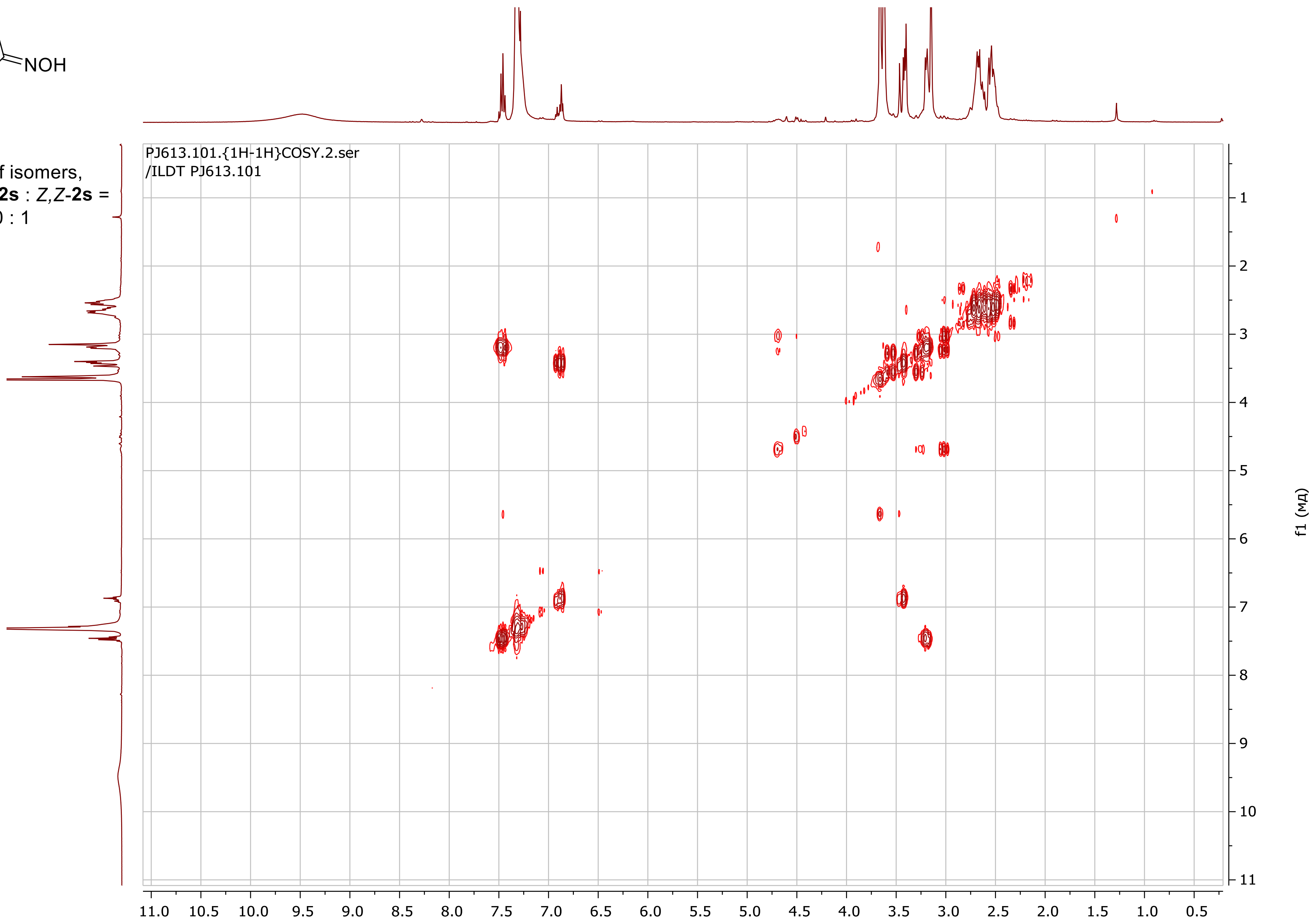

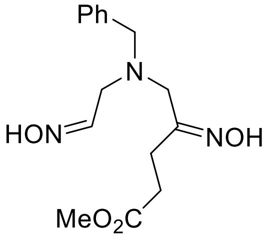

Dynamic mixture of isomers,
E,*E*-**2s** : *E*,*Z*-**2s** : *Z*,*E*-**2s** : *Z*,*Z*-**2s** =
3.3 : 1.5 : 2.0 : 1

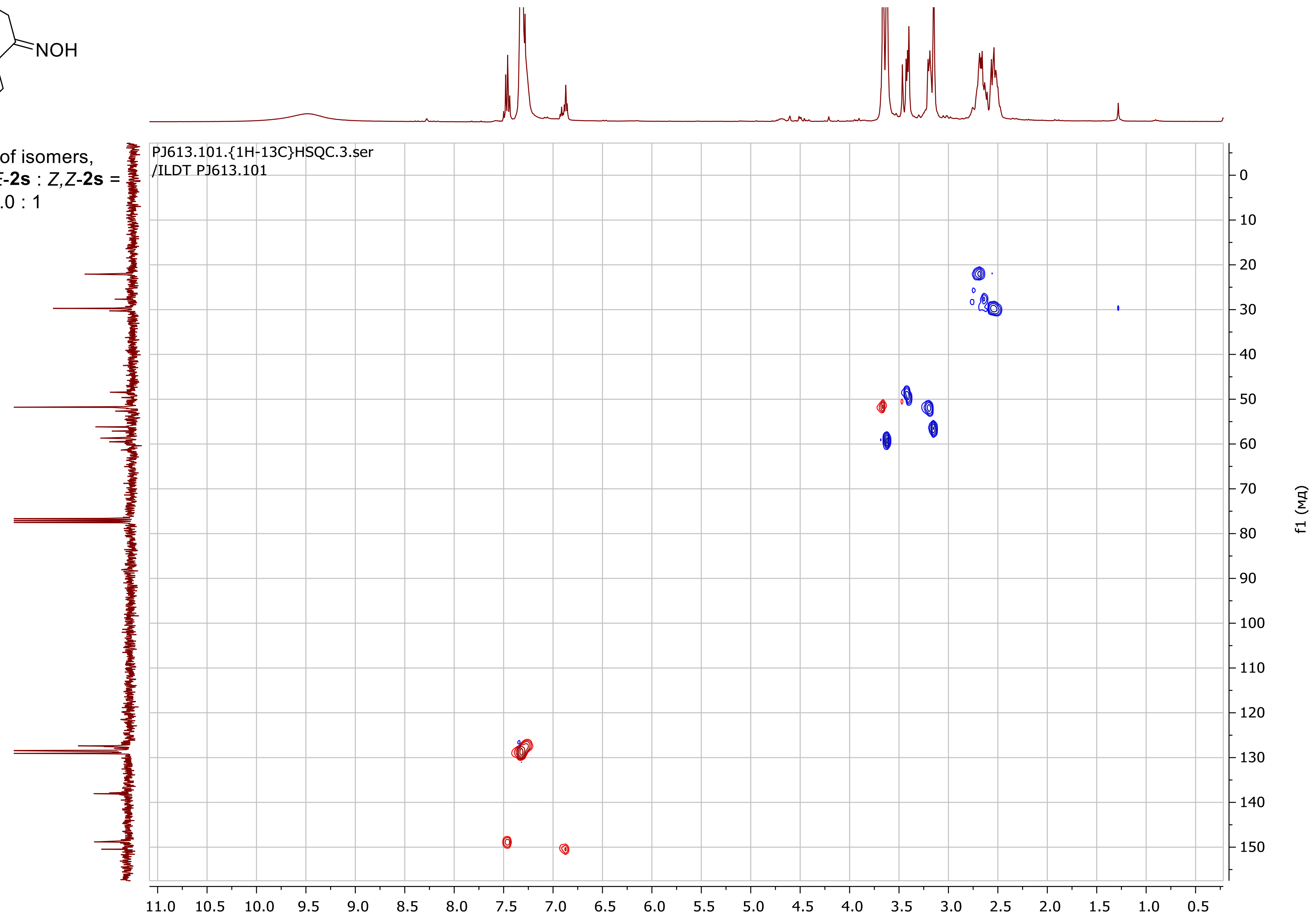

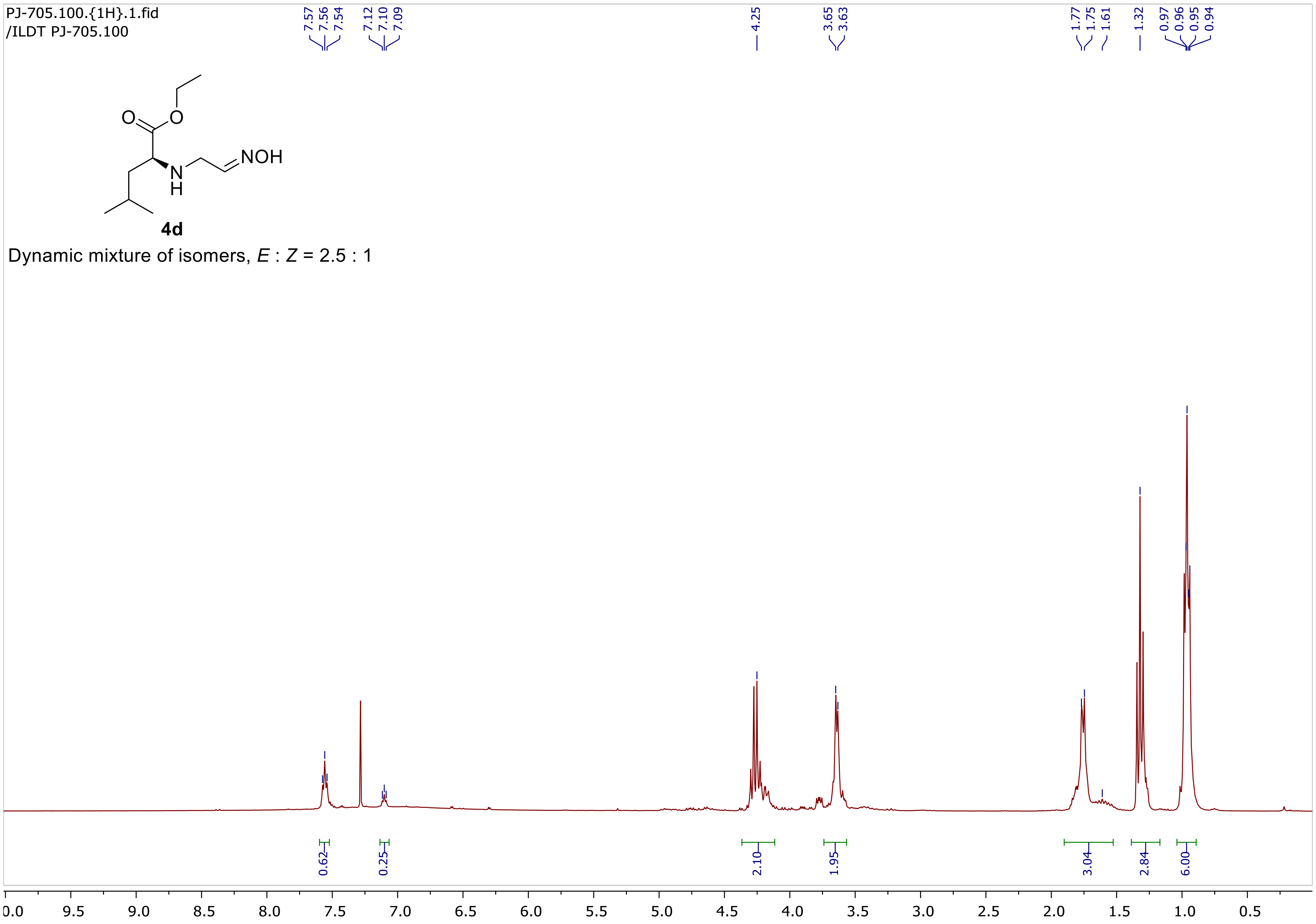
PJ-705.100.{1H}.1.fid
/ILDT PJ-705.100
7.57
7.56
7.54
7.12
7.10
7.09
4.25
3.65
3.63
1.77
1.75
1.61
1.32
0.97
0.96
0.95
0.94
O
O
NOH
N
H
4d
Dynamic mixture of isomers, E : Z = 2.5 : 1
0.62
0.25
2.10
1.95
3.04
2.84
6.00
0.0
9.5
9.0
8.5
8.0
7.5
7.0
6.5
6.0
5.5
5.0
4.5
4.0
3.5
3.0
2.5
2.0
1.5
1.0
0.5

PJ-705.100.{13C}.4.fid
/ILDT PJ-705.100

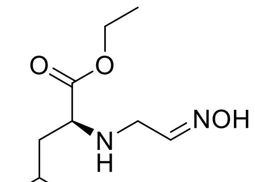

4d

Dynamic mixture of isomers, *E* : *Z* = 2.5 : 1

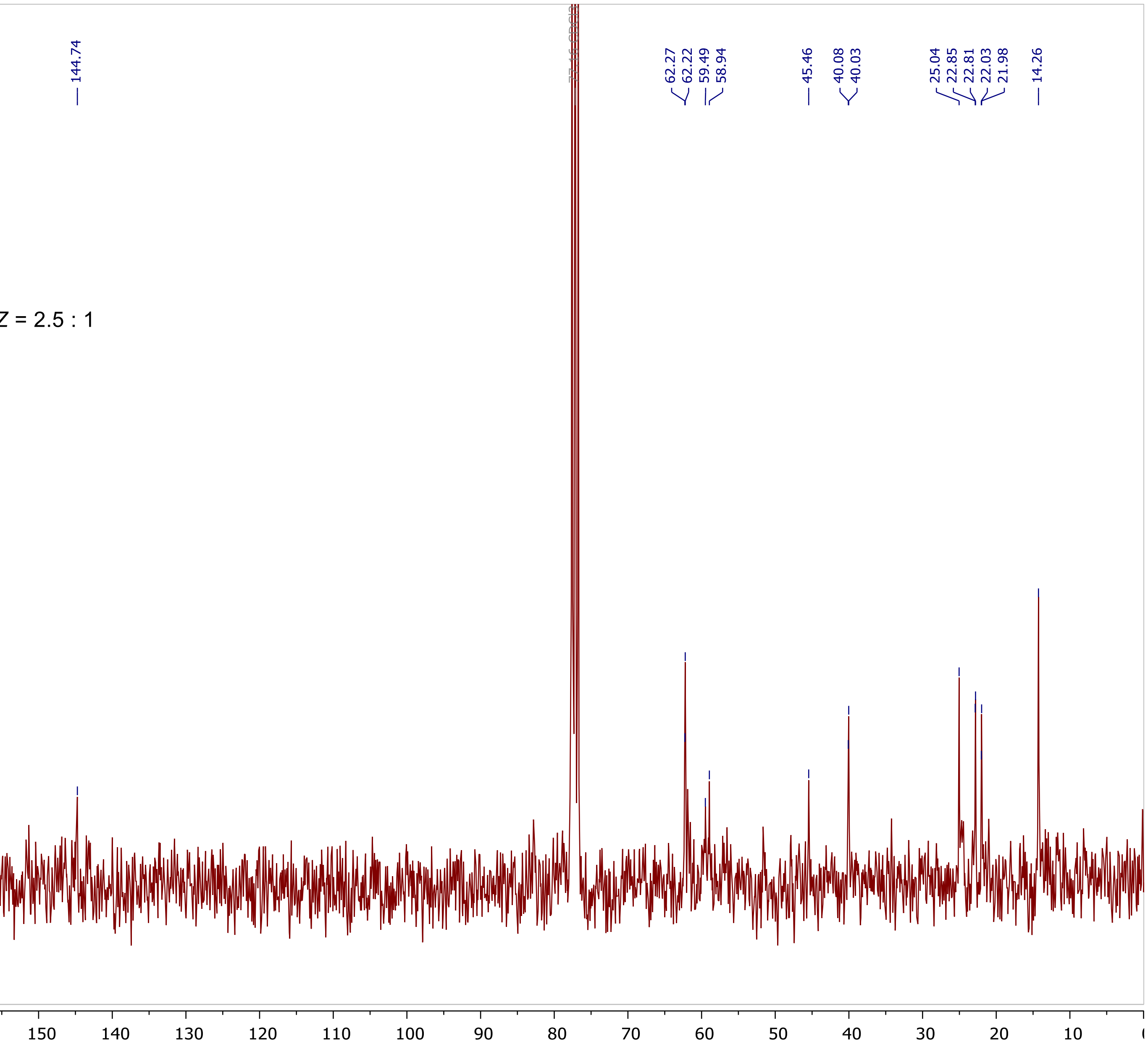

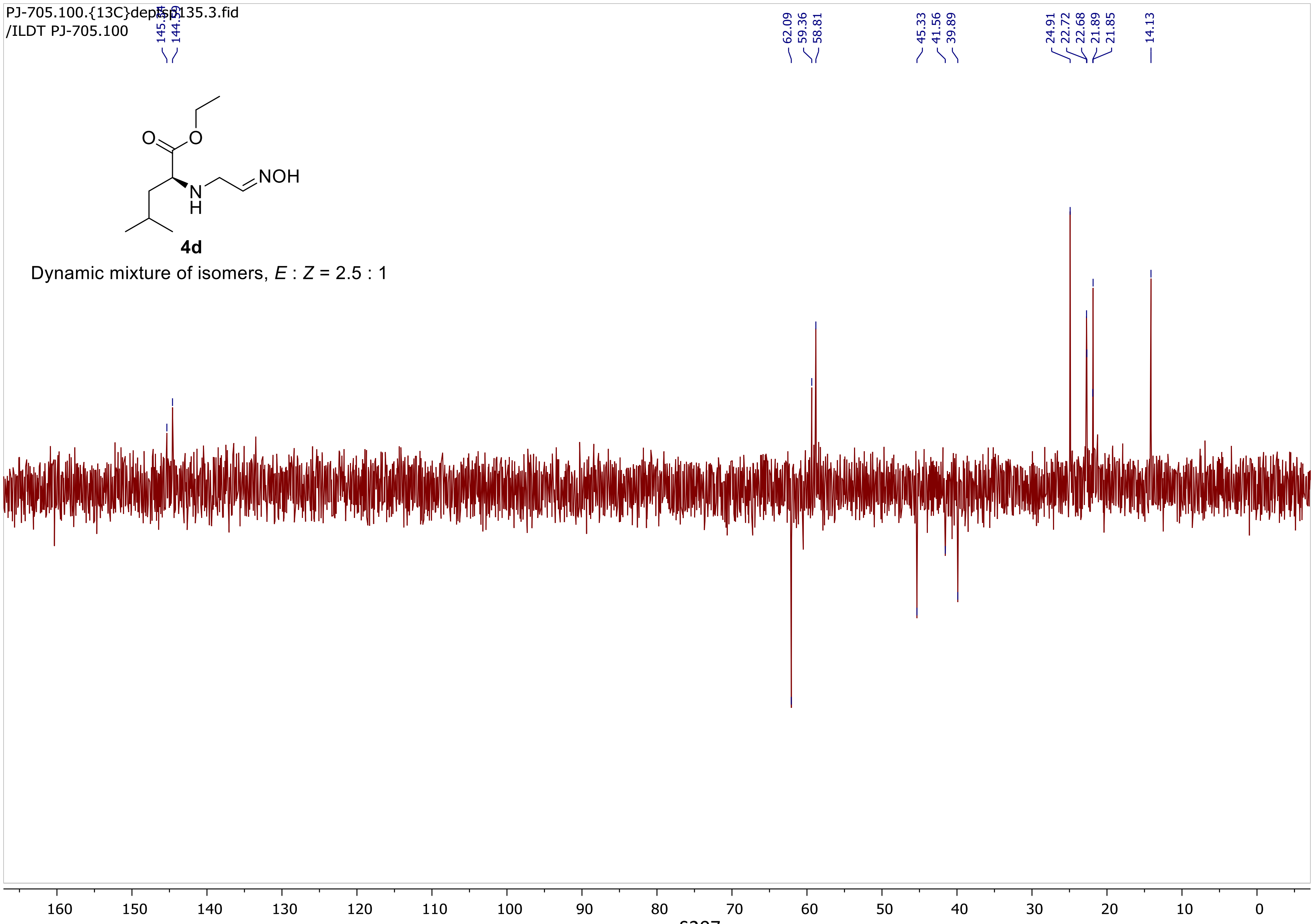

PJ-705.100.{13C}deptsp135.3.fid
/ILDT PJ-705.100
145.34
144.59
62.09
59.36
58.81
45.33
41.56
39.89
24.91
22.72
22.68
21.89
21.85
14.13
O
O
NOH
N
H
4d
Dynamic mixture of isomers, E : Z = 2.5 : 1
160
150
140
130
120
110
100
90
80
70
60
50
40
30
20
10
0

5. References

[1] A. D. Dilman, A. A. Tishkov, I. M. Lyapkalo, S. L. Ioffe, Y. A. Strelenko, V. A. Tartakovsky, *Synthesis* **1998**, *1998*, 181-185.

[2] A. D. Dilman, S. L. Ioffe, H. Mayr, *J. Org. Chem.* **2001**, *66*, 3196-3200.

[3] Y. A. Naumovich, S. L. Ioffe, A. Y. Sukhorukov, *J. Org. Chem.* **2019**, *84*, 7244-7254.

[4] A. Y. Sukhorokov, I. V. Bliznets, A. V. Lesiv, Y. A. Khomutova, Y. A. Strelenko, S. L. Ioffe, *Synthesis* **2005**, *2005*, 1077-1082.

[5] A. D. Dilman, A. A. Tishkov, I. M. Lyapkalo, S. L. Ioffe, V. V. Kachala, Y. A. Strelenko, V. A. Tartakovsky, *J. Chem. Soc., Perkin Trans. 1* **2000**, 2926-2929.

[6] M. C. Walton, Y. Yang, X. Hong, K. N. Houk, L. E. Overman, *Org. Lett.* **2015**, *17*, 6166-6169.

[7] L. Biancalana, M. Bortoluzzi, E. Ferretti, M. Hayatifar, F. Marchetti, G. Pampaloni, S. Zacchini, *RSC Adv.*, **2017**, *7*, 10158-10174.

[8] J. D. Firth, P. O'Brien, L. Ferris, *J. Am. Chem. Soc.* **2016**, *138*, 651-659.

[9] N. Thamban Chandrika, S. K. Shrestha, H. X. Ngo, S. Garneau-Tsodikova, *Bioorg. Med. Chem.* **2016**, *24*, 3680-3686.

[10] V. Arutla, J. Leal, X. Liu, S. Sokalingam, M. Raleigh, A. Adaralegbe, L. Liu, P. R. Pentel, S. M. Hecht, Y. Chang, *ACS Comb. Sci.* **2017**, *19*, 286-298.

[11] J. K. Johnson, E. M. Skoda, J. Zhou, E. Parrinello, D. Wang, K. O'Malley, B. R. Eyer, M. Kazancioglu, K. Eisermann, P. A. Johnston, J. B. Nelson, Z. Wang, P. Wipf, *ACS Med. Chem. Lett.* **2016**, *7*, 785–790.

[12] D. Manetti, C. Ghelardini, A. Bartolini, C. Bellucci, S. Dei, N. Galeotti, F. Gualtieri, M. N. Romanelli, S. Scapecchi, E. Teodori, *J. Med. Chem.* **2000**, *43*, 1969-1974.

[13] A. N. Semakin, A. Yu. Sukhorukov, S. L. Ioffe, V. A. Tartakovsky, *Synthesis* **2011**, *9*, 1403-1412.

[14] L. Toma, G. Cignarella, D. Barlocco, F. Ronchetti, *Tetrahedron* **1992**, *48*, 159-166.

[15] S. Crosignani, J. Gonzalez, D. Swinnen, *Org. Lett.* **2004**, *6*, 4579-4582.

[16] T. Wang, J. F. Kadow, Z. Zhang, Z. Yin, Q. Gao, D. Wu, D. D. Parker, Z. Yang, L. Zadjura, B. A. Robinson, Y.-F. Gong, W. S. Blair, P.-Y. Shi, G. Yamanaka, P.-F. Lin, N. A. Meanwell, *Bioorg. Med. Chem. Lett.* **2009**, *19*, 5140-5145.

[17] R. Wodtke, J. Steinberg, M. Köckerling, R. Löser, C. Mamat, *RSC Adv.* **2018**, *8*, 40921-40933.

[18] Maurer, A., Hoevelmann, S., Martin, E., Hentsch, B., Gassen, M., Kraus, J., Krauss, R. and Vincek, A.S., Novel compounds as histone deacetylase inhibitors, 2005, *U.S. Patent Application* 10/624,571 (4SC AG and G2M Cancer Drugs AG).

[19] M. Hesse, H. Meier, B. Zeeh, Spektroskopische Methoden in der Organischen Chemie, Georg Thieme Verlag, Stuttgart, 1995, p. 200.

[20] R. R. Fraser, M. Bresse, *Can. J. Chem*. **1983**, *61*, 576-578.
